# Decarboxylative 1,4-carbocyanation of 1,3-enynes to access tetra-substituted allenes *via* copper/photoredox dual catalysis†

**DOI:** 10.1039/d1sc02896k

**Published:** 2021-07-21

**Authors:** Ya Chen, Junjie Wang, Yixin Lu

**Affiliations:** Department of Chemistry, National University of Singapore 3 Science Drive 3 Singapore 117543 Singapore chmlyx@nus.edu.sg; Joint School of National University of Singapore and Tianjin University, International Campus of Tianjin University Binhai New City Fuzhou Fujian 350207 China

## Abstract

We disclose herein the first example of merging photoredox catalysis and copper catalysis for radical 1,4-carbocyanations of 1,3-enynes. Alkyl *N*-hydroxyphthalimide esters are utilized as radical precursors, and the reported mild and redox-neutral protocol has broad substrate scope and remarkable functional group tolerance. This strategy allows for the synthesis of diverse multi-substituted allenes with high chemo- and regio-selectivities, also permitting late stage allenylation of natural products and drug molecules.

## Abstract 2

An efficient synthesis of multi-substituted allenes by metallaphotoredox-catalyzed decarboxylative 1,4-carbocyanation of 1,3-enynes is described.

## Graphical abstract

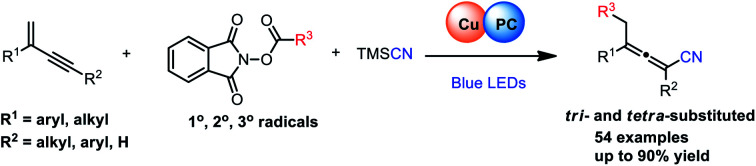

## Introduction

Owing to their unique structural characteristics and properties, allenes represent an important structural unit that is commonly present in a wide range of natural products and bioactive molecules, and they are also versatile building blocks in organic synthesis.^1^ Classical synthetic methods for allene preparation predominantly rely on functionalized alkynes.^2^ In recent years, 1,3-enynes have drawn much attention and perceived as desirable precursors for the efficient synthesis of multi-substituted allenes; both organo- and transition metal-catalyzed 1,4-difunctionalizations of 1,3-enynes were successfully developed.^3–6^ Despite rapid advance of this research field, some problems persist, *e.g.* the necessity of using activated 1,3-enynes, the requirement of sensitive organometallic reagents, and the difficulty to access tetrasubstituted allenes, among others. As part of our long term interest in utilizing allenes in organic synthesis, we were intrigued to tackle these synthetic challenges.

**Fig. 1:**
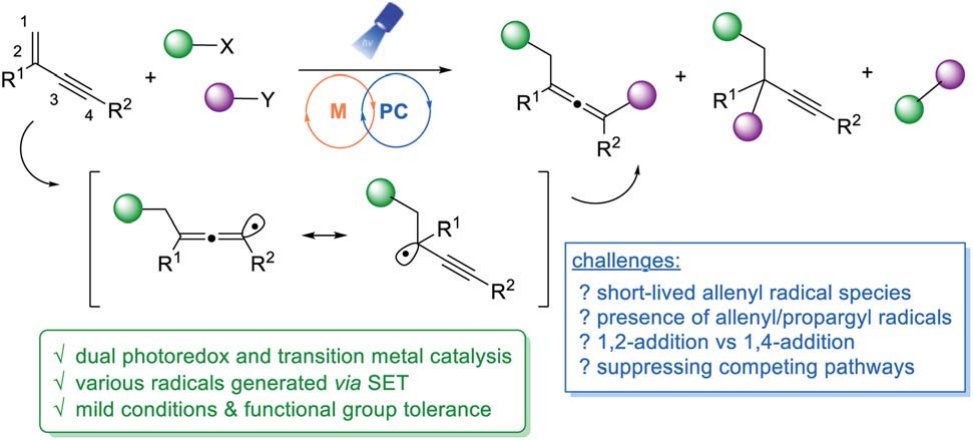
1,4-Difunctionalizations of 1,3-enynes *via* photoredox/transition metal dual catalysis.

Visible light photoredox and transition metal dual catalysis has recently emerged as a powerful synthetic strategy, offering a new paradigm for the effective construction of molecular architectures under mild conditions, often in a unique reaction pattern.^7^ In this context, 1,2-difunctionalizations of alkenes and alkynes *via* metallaphotoredox catalysis have recently attracted extensive research efforts because of simultaneous functionalizations at two sequential chemical bonding sites in one synthetic step.^8–10^ Despite the wide utilization of conventional alkenes or alkynes, activation of 1,3-enynes *via* metallaphotoredox catalysis still remains less explored to date.^11^ When the functionalizations of 1,3-enynes are concerned, there are a number of key challenges to be addressed. The allenyl radicals generated are short-lived,^12^ whereas allenes are excellent radical acceptors.^1*a*,13^ Controlling regioselectivity of the reaction is another challenge. When the radical addition to 1,3-enynes occurs, propargyl radicals and allenyl radicals are generated, and thus both 1,2-addition^14^ and 1,4-addition products may be formed.^15^ Very recently, the Liu group disclosed a divergent synthesis of CF_3_-substituted allenyl nitriles by using ligand-controlled radical 1,2- and 1,4- additions to 1,3-enynes.^16^ Shortly after, the Bao group reported a copper-catalyzed 1,4-difunctionalization of 1,3-enynes using diacyl peroxides as radical precursors.^17^ We hypothesized that merging visible light photoredox catalysis and transition metal catalysis may offer a general strategy through 1,4-difunctionalizations of 1,3-enynes to access a range of structurally diverse multi-substituted allenes. A variety of different radical precursors that can be activated under photoredox conditions may be utilized, and the employment of different transition metals in the proposed dual catalysis pathways will add in an extra dimension. Apparently, it is very challenging to gain good control of chemo- and regio-selectivities of a multi-component catalytic system in a radical process (Fig. 1).

To test our hypothesis, we chose *N*-hydroxyphthalimide (NHP) esters as a radical precursor, which are readily derived from abundant and inexpensive alkyl carboxylic acids and have been used extensively in radical decarboxylation processes.^18^ The addition of visible-light-induced radicals to 1,3-enynes to generate allenyl radical species is well anticipated. For the metal catalysis, we reasoned if copper(ii) is utilized, it may intercept the advanced allenyl radical to form copper(iii) species;^19^ the reductive elimination of which can lead to the creation of multi-substituted allenes. Herein, we disclose the first metallaphotoredox catalyzed radical 1,4-difunctionalization of 1,3-enynes, furnishing a wide range of tetra-substituted allenes under mild conditions.

## Results and discussion

We initiated our investigations by examining the reaction of 1,3-enynes **1a** with NHP ester **2a** and TMSCN using photoredox/copper dual catalytic systems (Table 1). After extensive screening (see the ESI, Tables S1 & S2†), we obtained 1,4-carbocyanation product **3a** in good yield, using a catalytic system consisting of Ir(ppy)_3_ and Cu(CH_3_CN)_4_PF_6_/bpy in DMF under irradiation with blue LEDs. A solvent screening was subsequently carried out (see the ESI, Table S3†) and dimethylacetamide (DMA) was identified as the solvent of choice. In the presence of 2.5 mol% copper catalyst and 1.0 mol% photocatalyst in DMA, allene **3a** was obtained in an isolated yield of 82% (entry 1). The reaction is regiospecific, as no 1,2-addition product was detected. It is noteworthy that the molar ratios of the three reactants had great influence on the chemoselectivity of the reaction. Reducing the molar equivalence of TMSCN or increasing the amount of radical precursor NHP ester led to the decreased yield of **3a**, with the formation of Heck-type byproducts^20^ from **1a** and **2a** observed. (entries 2 and 3). Further experiments demonstrated that the Cu(CH_3_CN)_4_PF_6_/bpy system, iridium photocatalyst, and visible light are indispensable for obtaining the desired product (entries 4–7). Finally, we showed that the reaction is sensitive towards both air and moisture (entries 8 and 9).

**Table 1:** Optimization of reaction conditions^a^

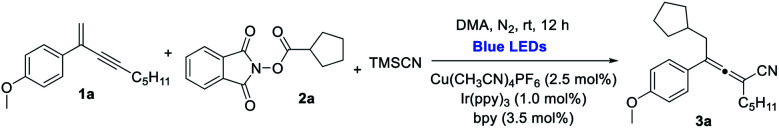		
Entry	Variation from the standard conditions	Yield^b^ (%)
1	None	88 (82)
2	1.1 equiv. TMSCN	65
3	1.5 equiv. **2a**	77
4	No Cu(CH_3_CN)_4_PF_6_	<5
5	No Ir(ppy)_3_	<5
6	No ligand	<5
7	In the dark	<5
8	In air	<5
9	10 μL H_2_O was added	46

a Reaction conditions: **1a** (0.2 mmol), **2a** (0.2 mmol) and TMSCN (0.4 mmol) in DMA (1.0 mL), Cu(CH_3_CN)_4_PF_6_ (2.5 mol%), bpy (3.5 mol%), Ir(ppy)_3_ (1.0 mol%), at room temperature, 30 W blue LEDs, 12 h.

b Determined by ^1^H NMR analysis of the crude product with CH_2_Br_2_ as an internal standard. Yield of the isolated product given in parentheses.

**Scheme 1:**
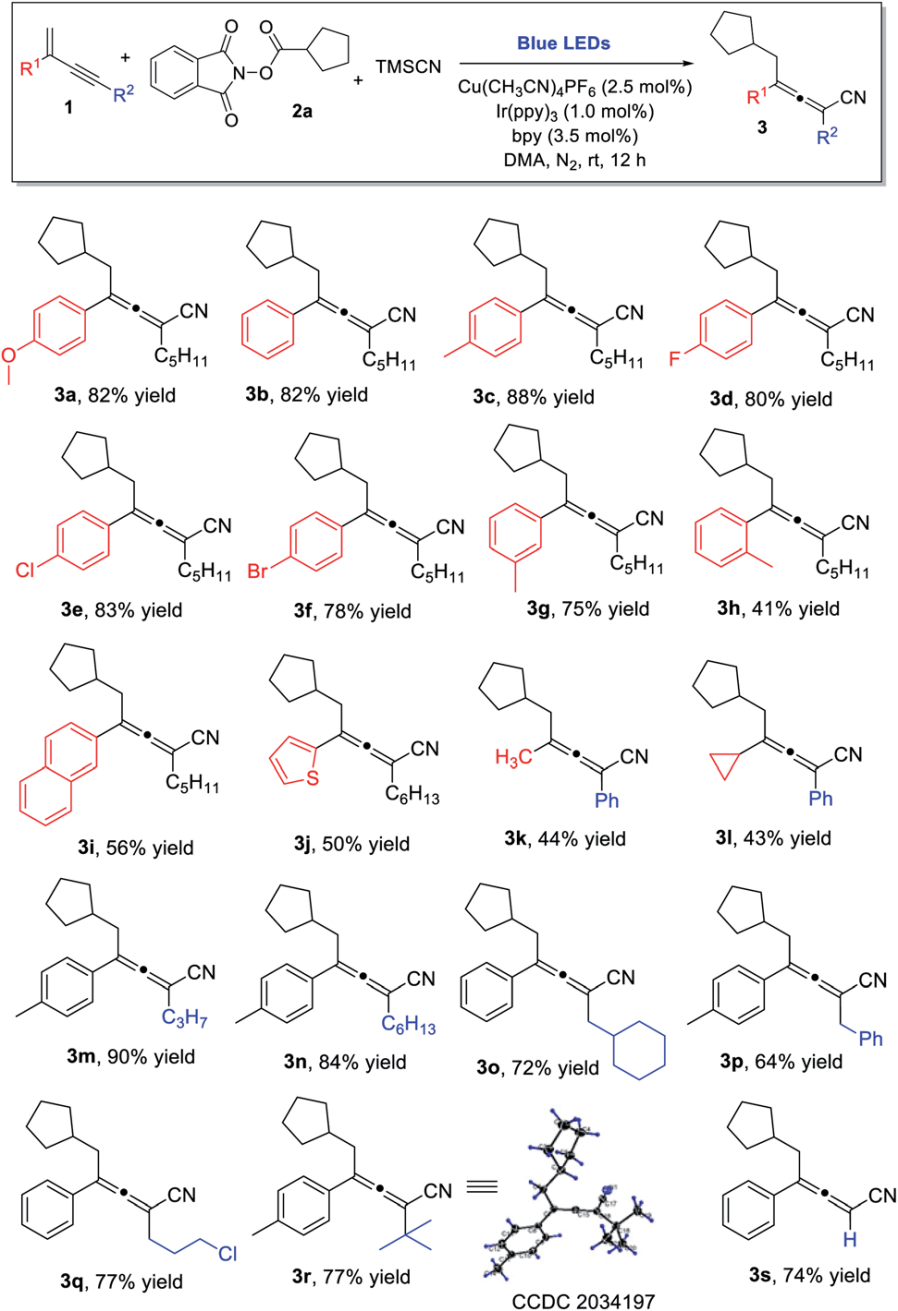
1,3-Enyne scope. ^*a*^ Reaction conditions: **1** (0.2 mmol), **2a** (0.2 mmol) and TMSCN (0.4 mmol) in DMA (1.0 mL), Cu(CH_3_CN)_4_PF_6_ (2.5 mol%), bpy (3.5 mol%), Ir(ppy)_3_ (1.0 mol%), at room temperature, 30 W blue LEDs, 12 h. Yields given refer to isolated yields.

With the established optimal reaction conditions, we proceeded to examine the reaction scope (Scheme 1). A wide range of 1,3-enynes could be utilized. For the C2-aryl substituent, both electron-donating and electron-withdrawing groups on the aromatic ring were well tolerated, and the corresponding tetra-substituted allenes were obtained in good yields (**3a–3f**). While the enyne bearing a *meta*-methyl phenyl substituent was found to be a good substrate (**3g**), the enynes containing an *ortho*-methyl phenyl or a naphthyl group at the C2-position led to much decreased yields (**3h** and **3i**). The reaction was also applicable to 1,3-enynes bearing a thiophene, methyl, or cyclopropyl substituent at the 2-position, although the yields of allenes were only modest (**3j**, **3k**, **3l**). The alkynyl moiety in the 1,3-enyne structures could also be varied; linear alkyl chains of different lengths (**3m**, **3n**), as well as the branched alkyl group (**3o**) and benzyl substituent (**3p**) were all found to be suitable. Moreover, the 1,3-enynes with a chlorine-containing alkyl chain (**3q**), a sterically hindered *tert*-butyl group (**3r**), or without a C4 substituent (**3s**) could all be utilized for the reaction, and the allene products were obtained in consistently good yields.

**Scheme 2:**
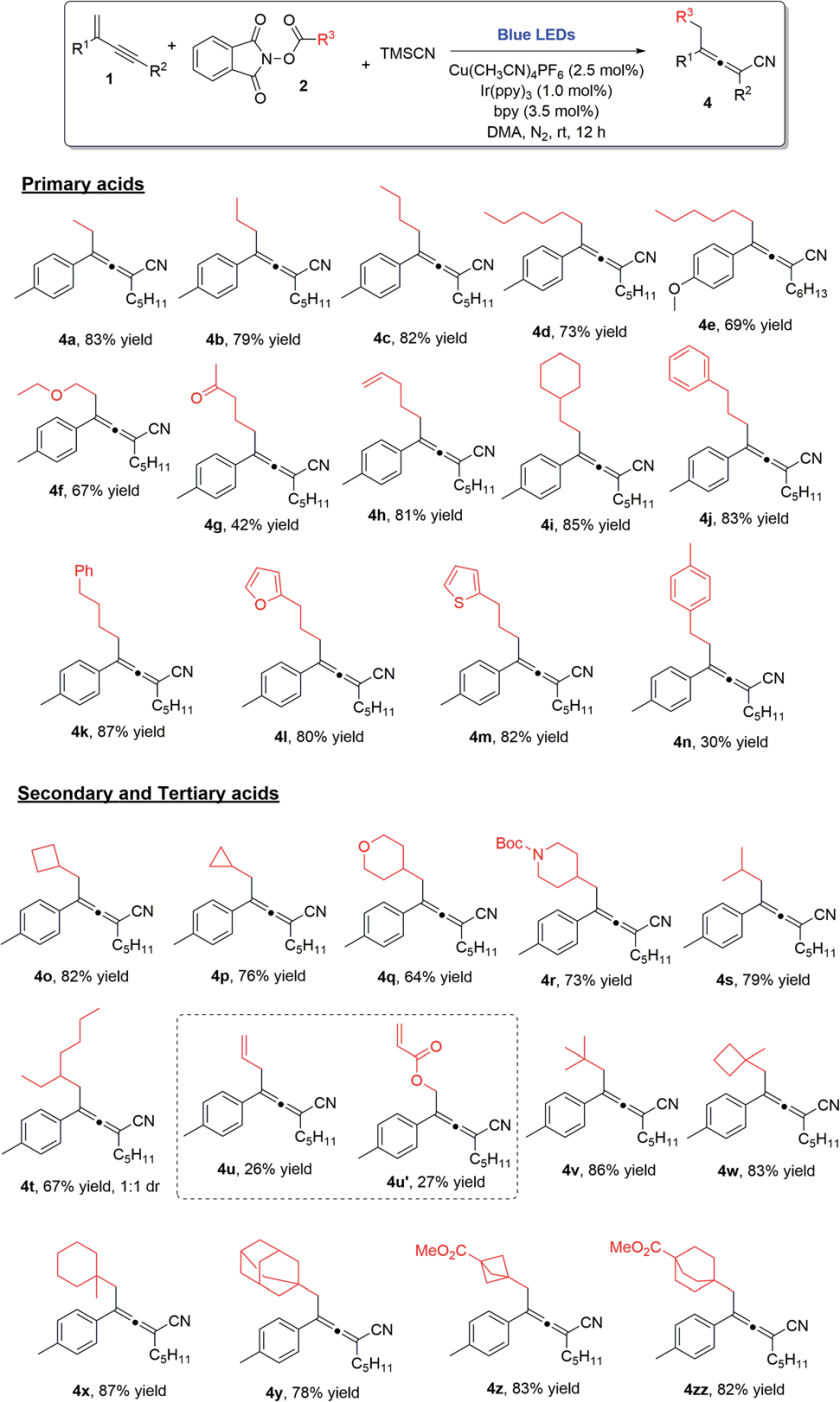
NHP ester scope. ^*a*^ Reaction conditions: **1** (0.2 mmol), **2** (0.2 mmol) and TMSCN (0.4 mmol) in DMA (1.0 mL), Cu(CH_3_CN)_4_PF_6_ (2.5 mol%), bpy (3.5 mol%), Ir(ppy)_3_ (1.0 mol%), at room temperature, 30 W blue LEDs, 12 h. Yields given refer to isolated yields.

Alkyl carboxylic acids are readily available and inexpensive, and thus their NHP esters are desirable starting materials in chemical transformations. The employment of different NHP esters in this decarboxylative 1,4-carbocyanation of 1,3-enynes allows for the incorporation of a group of diverse alkyl moieties into the tetra-substituted allene products. The substrate scope with regard to redox-active alkyl esters is remarkably broad (Scheme 2). Simple linear alkyl groups of various chain lengths (**4a–4e**), as well as alkyl substituents bearing an ether (**4f**), a ketone (**4g**), an alkene (**4h**), or a cyclohexyl (**4i**) moiety at the terminal position, were all found to be compatible with the reaction conditions, leading to the formation of the corresponding allenes in good yields. Moreover, the reaction was also applicable to linear alkyls containing a terminal phenyl, furan, or thiophene (**4j–4m**). Notably, when benzyl NHP ester was employed, the allene product was obtained in a lower yield (**4n**), due to the formation of a two-component cross-coupling byproduct.^21^ Significantly, secondary alkyl carboxylic acid derived NHP esters were amenable to the copper/photoredox catalytic process; cyclobutyl (**4o**), cyclopropyl (**4p**), pyran (**4q**), piperidine (**4r**), as well as simply branched alkyl chains (**4s** and **4t**) could all be readily incorporated into tetra-substituted allenes. Interestingly, when vinyl NHP ester was used, the anticipated 1,4-carbocyanation product (**4u**) was formed, along with a non-decarboxylative product (**4u′**). Lastly, we also examined tertiary alkyl NHP esters in this 1,4-difunctionalization process; remarkably, the desired allenes with highly sterically hindered alkyl groups incorporated were obtained in high yields and with excellent chemo- and regio-selectivities (**4v–4zz**).

**Scheme 3:**
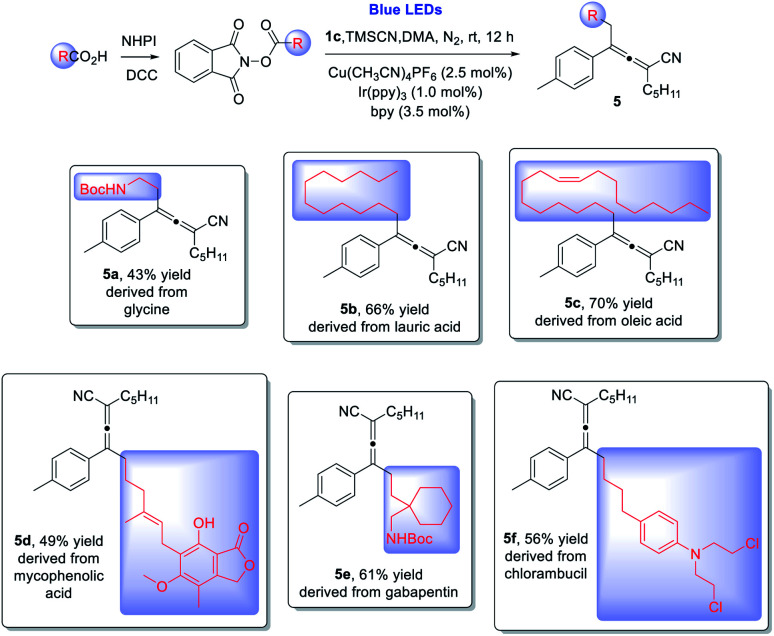
Late-stage functionalization of natural products and drugs. ^*a*^ Reaction conditions: **1c** (0.2 mmol), **2** (0.2 mmol) and TMSCN (0.4 mmol) in DMA (1.0 mL), Cu(CH_3_CN)_4_PF_6_ (2.5 mol%), bpy (3.5 mol%), Ir(ppy)_3_ (1.0 mol%), at room temperature, 30 W blue LEDs, 12 h. Yields given refer to isolated yields.

This copper/photoredox dual catalytic strategy enables the synthesis of allenes at room temperature under redox-neutral conditions without requiring the utilization of external oxidizing reagents, thus providing a unique entry to access complex allene structures. We next explored late stage allenylation of natural products and drug molecules: from glycine (**5a**) to fatty acids, *e.g.* lauric acid (**5b**) and oleic acid (**5c**), and to drugs, *e.g.* mycophenolic acid (**5d**), gabapentin (**5e**), and chlorambucil (**5f**). Remarkably, all the allenylations *via* 1,4-difunctionalization of enynes proceeded in reasonably good yields, displaying impressive chemo- and regio-selectivities and excellent functional group compatibility (Scheme 3).

**Scheme 4:**
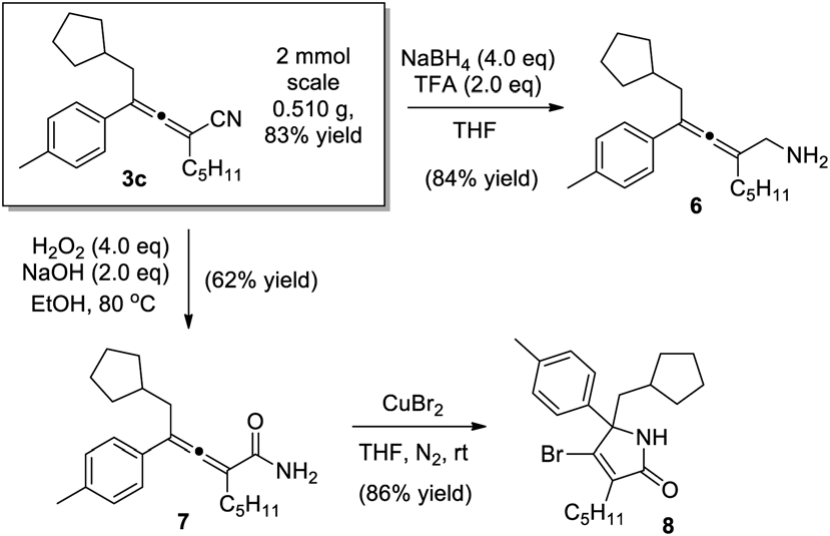
Synthetic manipulations of the allene product.

Synthetic manipulations of the 1,4-carbocyanation allene products were demonstrated (Scheme 4). The reaction could be scaled up smoothly, virtually without compromising the chemical yield. The α-allenyl amine **6**, an important skeleton found in a wide range of bioactive compounds,^1^ was readily prepared in 84% yield, through the reduction of allenenitrile **3c**. Alternatively, when **3c** was subjected to hydrolysis, allenamide **7** was obtained, and the subsequent cyclization of which led to highly functionalized lactam **8**.^22^

**Scheme 5:**
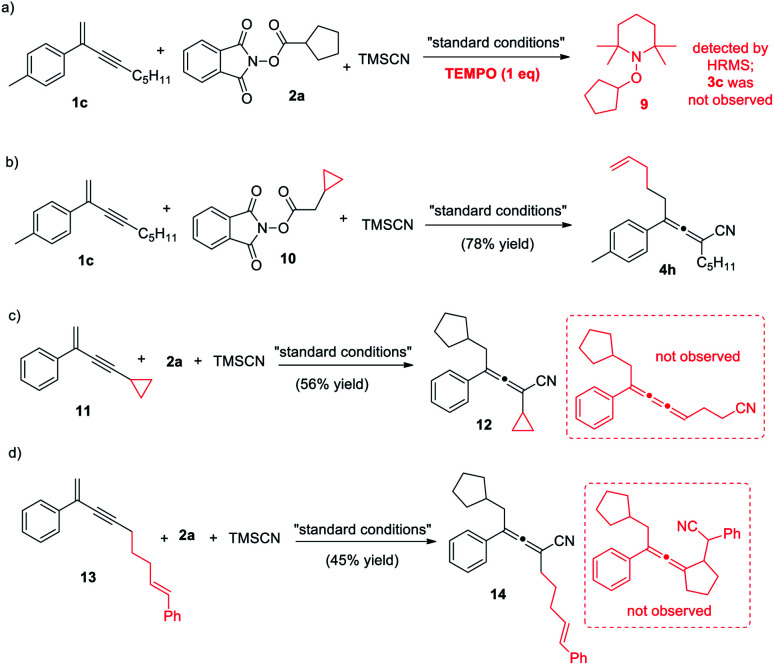
Control experiments.

To gain mechanistic insights into the reaction, we conducted a few control experiments (Scheme 5). When one molar equivalence of the radical scavenger TEMPO was added, the decarboxylative 1,4-carbocyanation was completely inhibited, and the TEMPO-captured **9** was detected, confirming the presence of an alkyl radical species during the reaction (eqn (a)). Several radical clock studies were also performed. The reaction of NHP ester derived from cyclopropylacetic acid produced exclusively the ring-opening product **4h** in 78% yield, strongly supporting that the reaction is a radical process. When substrates **11** and **13** were treated with NHP ester **2a**, the corresponding allenes were obtained in moderate yields. No ring-opening product from **11** or ring-closing product from **13** was detected, suggesting that the coupling of the allenyl radical with the Cu(ii) complex proceeds much faster.^17*b*^

**Fig. 2:**
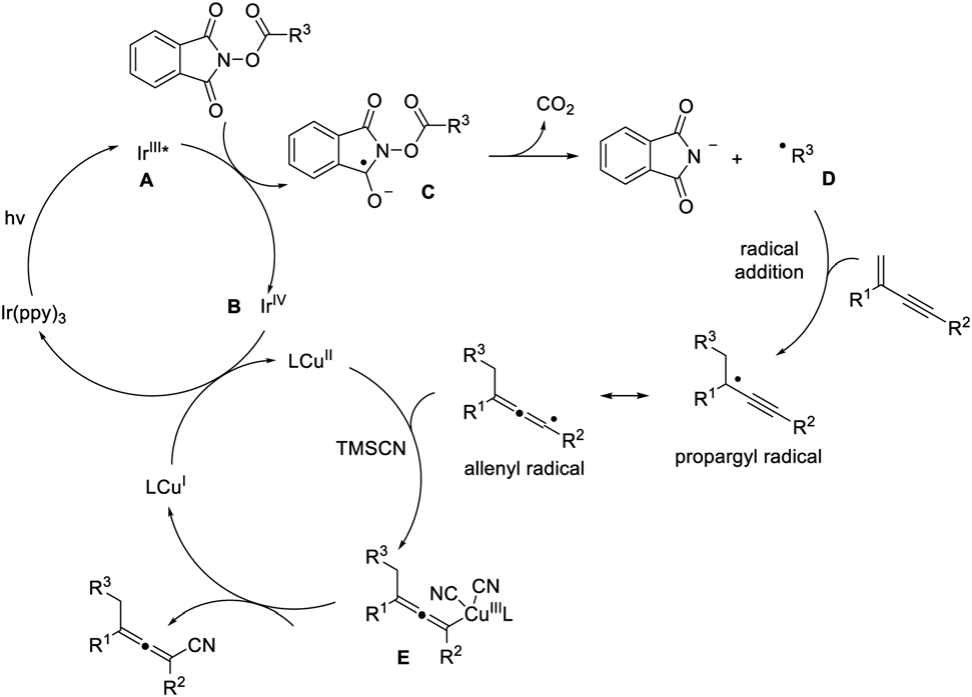
Proposed catalytic cycle.

On the basis of the above results as well as related literature reports,^16,17^ a plausible mechanism is proposed (Fig. 2). Excitation of photocatalyst Ir(ppy)_3_ leads to the active state (**A**) that undergoes single electron transfer with the NHP ester to produce the corresponding ester radical anion (**C**). The alkyl radical **D** is formed upon the extrusion of CO_2_. The subsequent addition of the alkyl radical to 1,3-enyne generates the propargyl radical, and the resonance form of which is an allenyl radical. The allenyl radical plays a key role in intercepting the copper(ii) complex and creates a copper(iii) species (**E**), which undergoes reductive elimination readily to furnish a tetra-substituted allene product, regenerating the copper catalyst at the same time. In this reaction cycle, the iridium photocatalyst is crucial in decarboxylative creation of the alkyl radical, as well as in facilitating the formation of copper(ii) species.

## Conclusions

In summary, we developed the first copper/photoredox dual catalysis enabled decarboxylative 1,4-carbocyanation of 1,3-enynes, for the synthesis of tetra-substituted allenes. The reaction is conducted under mild reaction conditions, displaying excellent chemoselectivity and regioselectivity, as well as remarkable functional group compatibility. The scope of the reaction is very broad, applicable to a wide range of primary, secondary, and tertiary NHP esters. Furthermore, the late-stage allenylation of natural products and drugs has also been demonstrated. We believe our reported method will have great conceptual implications, and may lead to the discovery of a range of general and versatile multi-functionalizations of 1,3-enynes and related compounds. We are currently working in these directions, and our findings will be reported in due course.

## Data availability

All experimental procedures, characterization, copies of NMR spectra for all new compounds related to this article can be found in the ESI.†

## Author contributions

Y. C. designed and performed the experiments, prepared the experimental part and the first draft of the manuscript. J. W. synthesized the NHP esters. Y. L. supervised the work, finalized the manuscript and coordinated the overall project.

## Conflicts of interest

There are no conflicts to declare.

## Supplementary Material

SC-012-D1SC02896K-s001

SC-012-D1SC02896K-s002
