# Supplementary material for: Decarboxylative 1,4-carbocyanation of 1,3-enynes to access tetra-substituted allenes via copper/photoredox dual catalysis
Source: Chem Sci. 2021 Jul 21;12(34):11316–21. doi: 10.1039/d1sc02896k (PMC8447876; doi:10.1039/d1sc02896k)
Supplement: SC-012-D1SC02896K-s001 [file SC-012-D1SC02896K-s001.pdf]

Electronic Supplementary Material (ESI) for Chemical Science.
This journal is © The Royal Society of Chemistry 2021

Supporting Information for

Decarboxylative 1,4-Carbocyanation of 1,3-Enynes to Access Tetra-substituted Allenes via Copper/Photoredox Dual Catalysis

Ya Chen,[a] Junjie Wang,[a,b] and Yixin Lu*,[a,b]

[a] Department of Chemistry, National University of Singapore, 3 Science Drive 3, Singapore, 117543

[b] Joint School of National University of Singapore and Tianjin University, International Campus of Tianjin University, Binhai New City, Fuzhou, Fujian, PR China, 350207

E-mail: chmlyx@nus.edu.sg

Contents

1. General information ---S2
2. Optimization of reaction conditions ---S2
3. General procedure for the synthesis of 1,3-enynes ----------------------S5
4. General procedure for 1,4-carbocyanation of 1,3-enynes--------------------------S10
5. Scale-up syntheses and further transformations-----------------------------------S30
6. Control experiments ---S32
7. X-ray crystallography of **3r** ---S37
8. References --S37
9. Copy of NMR spectra---S38

1. General information

Unless otherwise noted, all experiments were carried out under an atmosphere of nitrogen and anhydrous conditions. ^{1}H NMR and ^{13}C NMR spectra were recorded on Bruker AMX 400 Spectrometer (^{1}H 400 MHz and ^{13}C 100 MHz, respectively) and Bruker AMX 500 Spectrometer (^{1}H 500 MHz and ^{13}C 125 MHz, respectively). Chemical shifts (δ) were given in ppm and were referenced to residual solvent or TMS peaks. All high resolution mass spectra were obtained on a Bruker micrOTOFQ II (ESI). All the solvents were purified according to the standard procedures. Substrates of NHP ester were synthesized according to the modified literature methods.[1] All other chemicals which are commercially available were employed without further purification.

2. Optimization of reaction conditions

Table S1. Screening of copper catalysts [a]

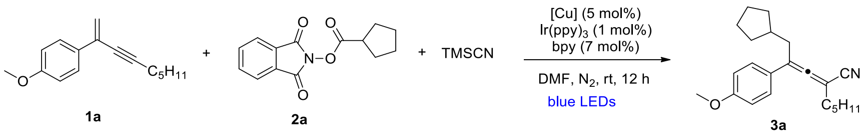

Entry	[Cu]	Yield (%)[b]
**1**	**$Cu(CH_3CN)_4PF_6$**	**74**
2	CuOAc	73
3	CuCl	50
4	CuBr	32
5	CuI	66
6	CuCN	68
7	$Cu(OTf)_2$	70
8	$CuCl_2$	16

[a] Reaction conditions: **1a** (0.2 mmol), **2a** (0.2 mmol) and TMSCN (0.4 mmol) in 1.0 mL DMF, [Cu] (5 mol%), bpy (7 mol%), $Ir(ppy)_3$ (1 mol%), at room temperature, 30 W blue LEDs, 12 h. [b] Determined by ^{1}H NMR analysis of crude product with CH_2Br_2 as an internal standard.

Table S2. Screening of ligands[a]

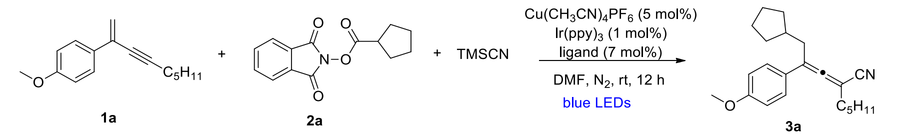

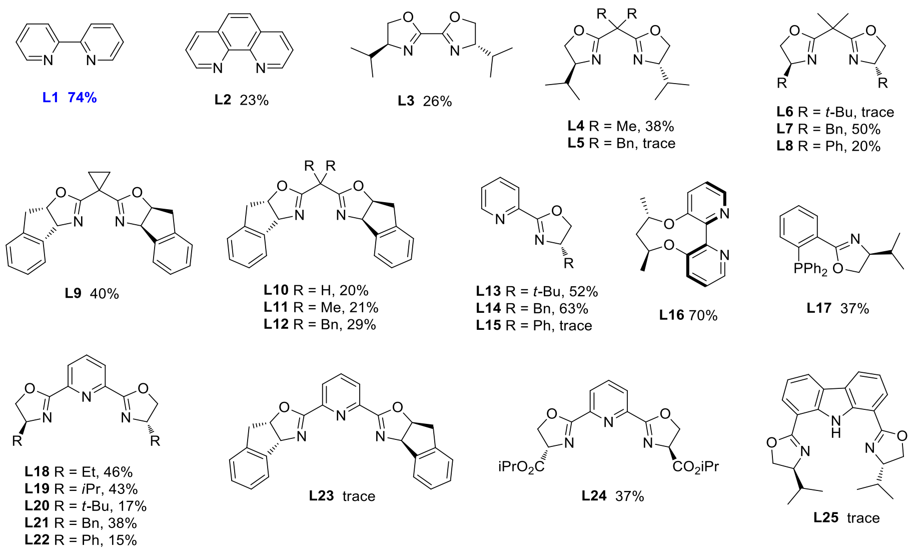

[a] Reaction conditions: **1a** (0.2 mmol), **2a** (0.2 mmol) and TMSCN (0.4 mmol) in 1.0 mL DMF, $Cu(CH_3CN)_4PF_6$ (5 mol%), ligand (7 mol%), $Ir(ppy)_3$ (1 mol%), at room temperature, 30 W blue LEDs, 12 h. [b] Determined by ^1H NMR analysis of crude product with CH_2Br_2 as an internal standard.

Table S3. Screening of solvents[a]

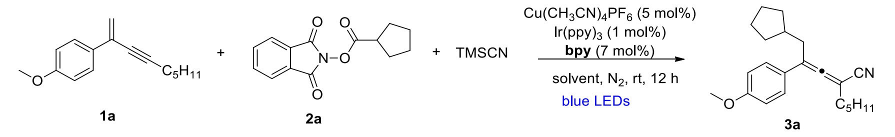

Entry	Solvent	Yield (%)[b]
1	DMF	74
2	DMSO	70
3	DMA	86
4	NMP	81
5	CH_3CN	trace
6	MeOH	trace

7	THF	15
8	EA	trace
9	DCM	trace
10	PhCl	18
**11**[c]	**DMA**	**88 (82)**

[a] Reaction conditions: **1a** (0.2 mmol), **2a** (0.2 mmol) and TMSCN (0.4 mmol) in 1.0 mL solvent, $Cu(CH_3CN)_4PF_6$ (5 mol%), bpy (7 mol%), $Ir(ppy)_3$ (1 mol%), at room temperature, 30 W blue LEDs, 12 h. [b] Determined by ^1H NMR analysis of crude product with CH_2Br_2 as an internal standard. Yield of the isolated product given within parentheses. [c] $Cu(CH_3CN)_4PF_6$ (2.5 mol%), bpy (3.5 mol%).

3. General procedure for the synthesis of 1,3-enynes[2]

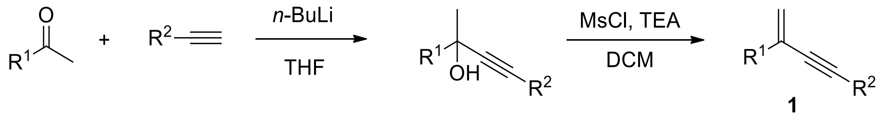

Under nitrogen atmosphere, *n*-BuLi (2.0 M in hexane, 5 mmol, 2.5 mL) was added dropwise to a solution of alkyne (5 mmol) in anhydrous THF (20 mL) at -78 °C. After addition, the resulting solution was stirred at room temperature for one hour. Then, cooled to -78 °C again, ketone (5 mmol) in THF (10 mL) was added dropwise. The reaction mixture was allowed to warm to room temperature and was monitored by TLC for completion. Once completion the reaction was quenched with saturated aqueous NH_4Cl and extracted with EtOAc three times. The combined organic layer was dried over Na_2SO_4 and concentrated under reduced pressure to afford the crude propargyl alcohol.

The resulting propargyl alcohol was dissolved in DCM (30 mL), and the mixture was cooled to 0 °C. TEA (25 mmol, 5 equiv) was added to this solution and methylsulfonyl chloride (12.5 mmol, 2.5 equiv) sequentially. After one hour the reaction was monitored by TLC for completion. Once completion the reaction was quenched with saturated aqueous NH_4Cl. The aqueous layer was extracted with DCM and the combined organic layers were washed with brine, dried over Na_2SO_4, filtered, and concentrated under reduced pressure. The crude material was purified by flash chromatography to yield the 1,3-enyne.

1b[2a], **1j**[2d], **1k**[2b], **1l**[2d], **1m**[2b], **1n**[2c], **1o**[2d], **1p**[2c], **1r**[2e], **1t**[2f], **11**[2c], **13**[2c] are known compounds in the references.

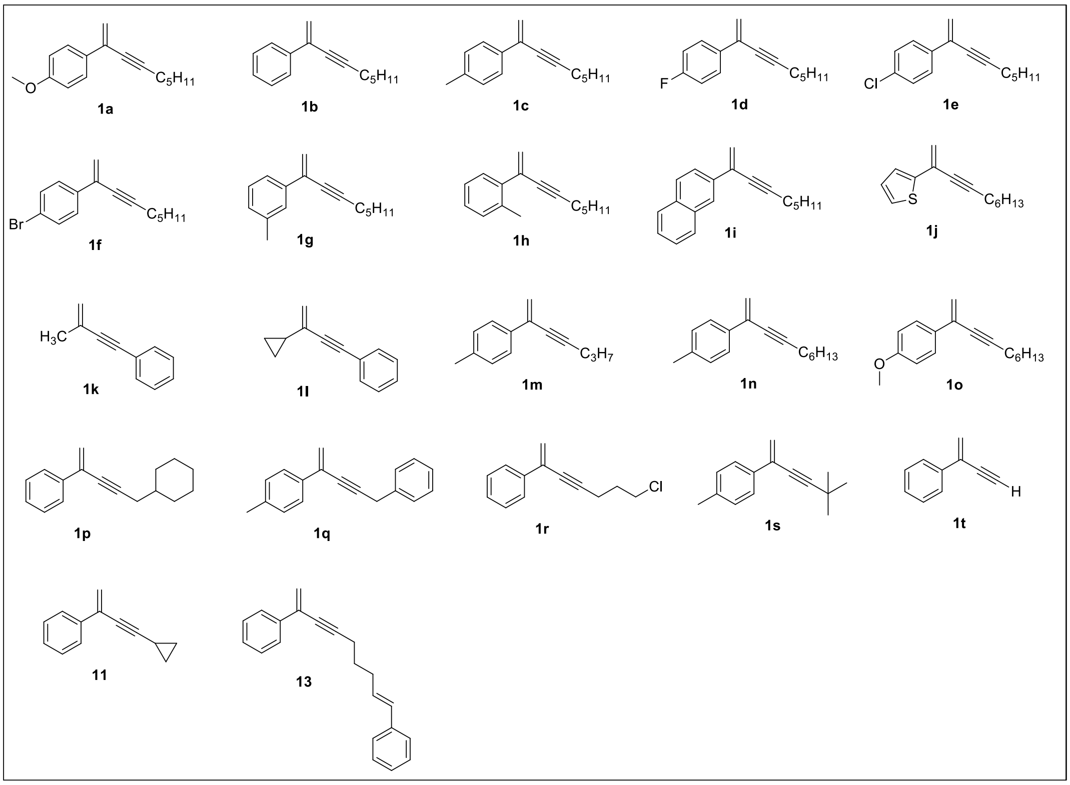

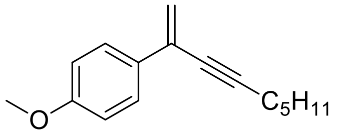

1a

1-methoxy-4-(non-1-en-3-yn-2-yl)benzene (1a): Pale yellow oil, isolated yield 50%. ^{1}H NMR (400 MHz, $CDCl_3$) δ 7.52 (d, *J* = 9.2 Hz, 2H), 6.79 (d, *J* = 8.8 Hz, 2H), 5.65 (d, *J* = 1.2 Hz, 1H), 5.40 (d, *J* = 1.2 Hz, 1H), 3.73 (s, 3H), 2.32 (t, *J* = 7.0 Hz, 2H), 1.56 – 1.49 (m, 2H), 1.40 – 1.25 (m, 4H), 0.85 (t, *J* = 7.2 Hz, 3H); ^{13}C NMR (100 MHz, $CDCl_3$) δ 159.7, 130.6, 130.4, 127.4, 117.5, 113.7, 91.9, 80.1, 55.4, 31.3, 28.6, 22.4, 19.5, 14.1.

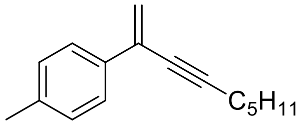

1c

1-methyl-4-(non-1-en-3-yn-2-yl)benzene (1c): Pale yellow oil, isolated yield 50%. ^{1}H NMR (400 MHz, $CDCl_3$) δ 7.56 (d, *J* = 8.0 Hz, 2H), 7.17-7.15 (m, 2H), 5.81 (d, *J* = 1.2 Hz, 1H), 5.54 (d, *J* = 1.2 Hz, 1H), 2.42 (t, *J* = 7.2 Hz, 2H), 2.37 (s, 3H), 1.65 – 1.56 (m, 2H), 1.49 – 1.34 (m, 4H), 0.94 (t, *J* = 7.2 Hz, 3H); ^{13}C NMR (100 MHz, $CDCl_3$) δ 138.1, 135.2, 130.9, 129.1, 126.1, 118.5, 92.0, 80.0, 31.3, 28.6, 22.4, 21.3, 19.5, 14.2.

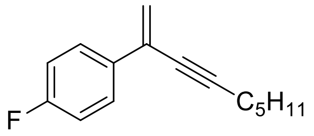

1d

1-fluoro-4-(non-1-en-3-yn-2-yl)benzene (1d): Pale yellow oil, isolated yield 59%. ^{1}H NMR (400 MHz, $CDCl_3$) δ 7.64 – 7.61 (m, 2H), 7.05 – 7.00 (m, 2H), 5.77 (d, *J* = 1.2 Hz, 1H), 5.56 – 5.55 (m, 1H), 2.41 (t, *J* = 7.0 Hz, 2H), 1.64 – 1.58 (m, 2H), 1.46 – 1.33 (m, 4H), 0.93 (t, *J* = 7.0 Hz, 3H); ^{13}C NMR (100 MHz, $CDCl_3$) δ 162.9 (d, J_{C-F} = 246.0 Hz), 134.1 (d, J_{C-F} = 3.0 Hz), 130.1, 127.9 (d, J_{C-F} = 8.0 Hz), 119.2 (d, J_{C-F} = 1.0 Hz), 115.2 (d, J_{C-F} = 22.0 Hz), 92.5, 79.7, 31.3, 28.6, 22.4, 19.5, 14.2.

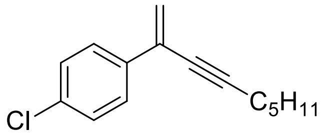

1e

1-chloro-4-(non-1-en-3-yn-2-yl)benzene (1e): Pale yellow oil, isolated yield 68%. ^{1}H NMR (400 MHz, $CDCl_3$) δ 7.54 (d, *J* = 8.4 Hz, 2H), 7.27 (d, *J* = 8.4 Hz, 2H), 5.77 (d, *J* = 0.8 Hz, 1H), 5.55 (d, *J* = 0.8 Hz, 1H), 2.37 (t, *J* = 7.2 Hz, 2H), 1.59 – 1.52 (m, 2H), 1.43 – 1.29 (m, 4H), 0.89 (t, *J* = 7.2 Hz, 3H); ^{13}C NMR (100 MHz, $CDCl_3$) δ 136.5, 134.1, 130.1, 128.5, 127.5, 119.8, 92.7, 79.5, 31.3, 28.5, 22.4, 19.5, 14.1.

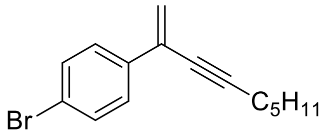

1f

1-bromo-4-(non-1-en-3-yn-2-yl)benzene (1f): Pale yellow oil, isolated yield 49%. ^{1}H NMR (400 MHz, $CDCl_3$) δ 7.53 – 7.50 (m, 2H), 7.48 – 7.45 (m, 2H), 5.82 (d, *J* = 0.8 Hz, 1H), 5.59 (m, 1H), 2.40 (t, *J* = 7.2 Hz, 2H), 1.63 – 1.58 (m, 2H), 1.45 – 1.33 (m, 4H), 0.93 (t, *J* = 7.2 Hz, 3H); ^{13}C NMR (100 MHz, $CDCl_3$) δ 136.9, 131.5, 130.2, 127.9, 122.3, 119.9, 92.7, 79.4, 31.3, 28.5, 22.4, 19.5, 14.2.

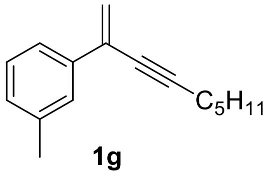

1g

1-methyl-3-(non-1-en-3-yn-2-yl)benzene (1g): Pale yellow oil, isolated yield 55%. ^{1}H NMR (500 MHz, $CDCl_3$) δ 7.51 – 7.48 (m, 2H), 7.28 – 7.24 (m, 1H), 7.15 – 7.13 (m, 1H), 5.85 (d, *J* = 1.5 Hz, 1H), 5.59 (d, *J* = 1.0 Hz, 1H), 2.44 (t, *J* = 7.0 Hz,

2H), 2.40 (s, 3H), 1.68 – 1.62 (m, 2H), 1.51 – 1.45 (m, 2H), 1.42 – 1.37 (m, 2H), 0.96 (t, *J* = 7.3 Hz, 3H); ^{13}C NMR (125 MHz, $CDCl_3$) δ 137.9, 131.2, 129.0, 128.3, 127.0, 123.3, 119.3, 92.1, 80.0, 31.3, 28.6, 22.4, 21.6, 19.5, 14.2.

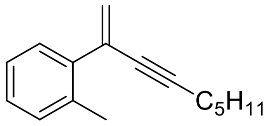

1h

1-methyl-2-(non-1-en-3-yn-2-yl)benzene (1h): Pale yellow oil, isolated yield 46%. ^{1}H NMR (400 MHz, $CDCl_3$) δ 7.29-7.26 (m, 1H), 7.23-7.17 (m, 3H), 5.71-5.70 (m, 1H), 5.41-5.40 (m, 1H), 2.46-2.45 (m, 3H), 2.37-2.32 (m, 2H), 1.59 – 1.53 (m, 2H), 1.42 – 1.31 (m, 4H), 0.95 – 0.90 (m, 3H); ^{13}C NMR (100 MHz, $CDCl_3$) δ 140.1, 135.6, 132.4, 130.4, 128.8, 127.8, 125.9, 123.9, 92.1, 80.8, 31.3, 28.5, 22.3, 20.3, 19.6, 14.1.

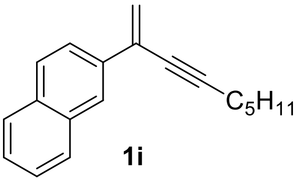

1i

2-(non-1-en-3-yn-2-yl)naphthalene (1i): Pale yellow oil, isolated yield 40%. ^{1}H NMR (400 MHz, $CDCl_3$) δ 8.18 (m, 1H), 7.90-7.78 (m, 4H), 7.52-7.47 (m, 2H), 6.01 (d, *J* = 0.8 Hz, 1H), 5.71 (d, *J* = 1.2 Hz, 1H), 2.50 (t, *J* = 7.0 Hz, 3H), 1.73-1.66 (m, 2H), 1.56 – 1.49 (m, 2H), 1.47 – 1.39 (m, 2H), 0.98 (t, *J* = 7.2 Hz, 3H); ^{13}C NMR (100 MHz, $CDCl_3$) δ 135.2, 133.4, 133.3, 131.1, 128.5, 128.0, 127.7, 126.4, 126.3, 126.1, 123.6, 119.8, 92.4, 79.9, 31.4, 28.6, 22.4, 19.6, 14.2.

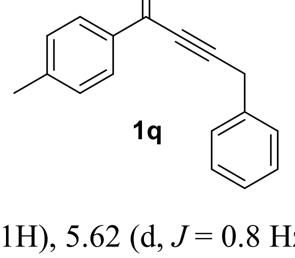
1q

1-methyl-4-(5-phenylpent-1-en-3-yn-2-yl)benzene (1q): Pale yellow oil, isolated yield 45%. ^{1}H NMR (400 MHz, $CDCl_3$) δ 7.58 (d, *J* = 8.4 Hz, 2H), 7.44-7.41 (m, 2H), 7.38-7.34 (m, 2H), 7.29-7.25 (m, 1H), 7.18-7.16 (m, 2H), 5.87 (d, *J* = 1.2 Hz, 1H), 5.62 (d, *J* = 0.8 Hz, 1H), 3.86 (s, 2H), 2.37 (s, 3H); ^{13}C NMR (100 MHz, $CDCl_3$) δ 138.2, 136.9, 135.0, 130.7, 129.1, 128.7, 128.1, 126.8, 126.1, 119.3, 88.9, 82.2, 25.9, 21.3.

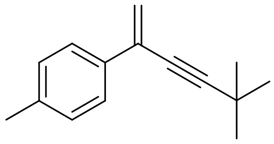

1s

1-(5,5-dimethylhex-1-en-3-yn-2-yl)-4-methylbenzene (1s): Pale yellow oil, isolated yield 80%. ^{1}H NMR (400 MHz, $CDCl_3$) δ 7.55 (d, *J* = 8.0 Hz, 2H), 7.17-7.15 (m, 2H), 5.79 (d, *J* = 1.2 Hz, 1H), 5.52 (d, *J* = 1.6 Hz, 1H), 2.36 (s, 3H), 1.33 (s, 9H); ^{13}C NMR (100 MHz, $CDCl_3$) δ 138.1, 135.3, 130.8, 129.1, 126.1, 118.4, 100.0, 78.5, 31.2, 28.2, 21.3.

4. General procedure for 1,4-carbocyanation of 1,3-enynes

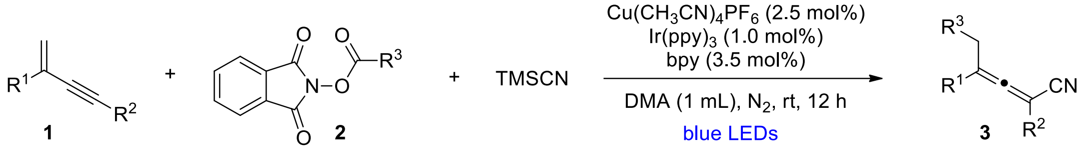

General procedure : In a 10 mL sealed tube, $Cu(CH_3CN)_4PF_6$ (0.005 mmol, 2.5 mol%), bpy (0.007 mmol, 3.5 mol%), $Ir(ppy)_3$ (0.002 mmol, 1.0 mol%) and NHP ester (0.2 mmol, 1.0 equiv) were added in degassed DMA under a nitrogen atmosphere, and the mixture was stirred at room temperature for 30 minutes. Then 1,3-enyne (0.2 mmol, 1.0 equiv) and TMSCN (0.4 mmol, 2.0 equiv) were sequentially added. The reaction mixture was stirred at room temperature under 30 W blue LEDs irradiation for 12 hours. After the reaction completion, the reaction mixture was diluted with EtOAc, then washed with brine, dried over Na_2SO_4, filtered, and concentrated under reduced pressure. The residue was purified by column chromatography on silica gel (hexane/EtOAc) to afford the corresponding products.

The analytical data of the products are summarized below. **4e** is known compound in the references.[2d]

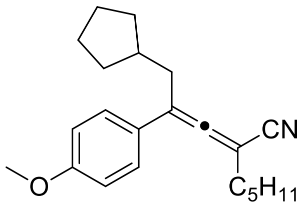

3a

2-(3-cyclopentyl-2-(4-methoxyphenyl)prop-1-en-1-ylidene)heptanenitrile (3a): Pale yellow oil, 52.8 mg, isolated yield 82%. ^{1}H NMR (400 MHz, $CDCl_3$) δ 7.27 (d, *J* = 9.2 Hz, 2H), 6.89 (d, *J* = 9.2 Hz, 2H), 3.82 (s, 3H), 2.49 (d, *J* = 7.2 Hz, 2H), 2.29 – 2.26 (m, 2H), 2.06 – 2.02 (m, 1H), 1.84 – 1.79 (m, 2H), 1.66 – 1.51 (m, 6H), 1.35 – 1.32 (m, 4H), 1.27 – 1.19 (m, 2H), 0.90 – 0.87 (m, 3H); ^{13}C NMR (100 MHz, $CDCl_3$) δ 212.8, 159.8, 128.0, 126.0, 116.0, 114.3, 111.2, 84.5, 55.5, 38.2, 37.2, 32.9, 32.1, 31.1, 27.7, 25.4, 25.4, 22.4, 14.1. HRMS (ESI): m/z calcd. for $C_{22}H_{29}NONa^+$($[M+Na]^+$) = 346.2165, found = 346.2159.

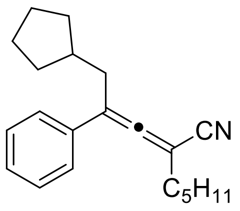

3b

2-(3-cyclopentyl-2-phenylprop-1-en-1-ylidene)heptanenitrile (3b): Colorless oil, 48.1 mg, isolated yield 82%. ^{1}H NMR (400 MHz, $CDCl_3$) δ 7.39 – 7.28 (m, 5H), 2.53 (d, *J* = 7.2 Hz, 2H), 2.31 – 2.27 (m, 2H), 2.05 – 2.02 (m, 1H), 1.87 – 1.79 (m, 2H), 1.67 – 1.51 (m, 6H), 1.38 – 1.18 (m, 6H), 0.91 – 0.86 (m, 3H); ^{13}C NMR (100 MHz, $CDCl_3$) δ 212.8, 134.0, 128.9, 128.4, 126.8, 115.9, 111.6, 84.7, 38.2, 37.1, 32.9, 32.9, 32.0, 31.1, 27.6, 25.4, 25.4, 22.4, 14.1. HRMS (ESI): m/z calcd. for $C_{21}H_{27}NNa^+$([M+Na]$^+$) = 316.2036, found = 316.2038.

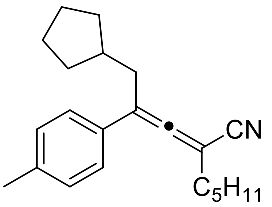

3c

2-(3-cyclopentyl-2-(*p*-tolyl)prop-1-en-1-ylidene)heptanenitrile (3c): Colorless oil, 54.0 mg, isolated yield 88%. ^{1}H NMR (400 MHz, $CDCl_3$) δ 7.24 (d, *J* = 6.8 Hz, 2H), 7.17 (d, *J* = 8.0 Hz, 2H), 2.50 (d, *J* = 7.6 Hz, 2H), 2.36 (s, 3H), 2.30 – 2.26 (m, 2H), 2.06 – 2.01 (m, 1H), 1.86 – 1.79 (m, 2H), 1.67 – 1.50 (m, 6H), 1.37 – 1.29 (m, 4H), 1.27 – 1.19 (m, 2H), 0.90 – 0.87 (m, 3H); ^{13}C NMR (100 MHz, $CDCl_3$) δ 212.8, 138.4, 130.9, 129.6, 126.7, 116.0, 111.4, 84.5, 38.2, 37.1, 32.9, 32.0, 31.1, 27.6, 25.4, 25.4, 22.4, 21.3, 14.1. HRMS (ESI): m/z calcd. for $C_{22}H_{29}NNa^+$([M+Na]$^+$) = 330.2192, found = 330.2201.

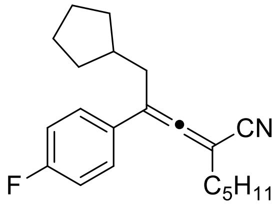

3d

2-(3-cyclopentyl-2-(4-fluorophenyl)prop-1-en-1-ylidene)heptanenitrile (3d): Pale yellow oil, 49.9 mg, isolated yield 80%. ^{1}H NMR (400 MHz, $CDCl_3$) δ 7.33 – 7.29 (m, 2H), 7.07 – 7.03 (m, 2H), 2.49 (d, *J* = 7.2 Hz, 2H), 2.31 – 2.27 (m, 2H), 2.05 – 1.99 (m, 1H), 1.86 – 1.78 (m, 2H), 1.67 – 1.52 (m, 6H), 1.35 – 1.31 (m, 4H), 1.26 – 1.19 (m, 2H), 0.90 – 0.86 (m, 3H); ^{13}C NMR (100 MHz, $CDCl_3$) δ 212.6 (d, J_{C-F} = 2.0 Hz), 162.7 (d, J_{C-F} = 247.0 Hz), 130.0 (d, J_{C-F} = 4.0 Hz), 128.5 (d, J_{C-F} = 8.0 Hz), 115.9 (d, J_{C-F} = 21.0 Hz), 115.7, 110.8, 84.9, 38.1, 37.3, 32.9, 32.9, 32.0, 31.1, 27.6, 25.4, 25.4, 22.4, 14.1. HRMS (ESI): m/z calcd. for $C_{21}H_{26}FNNa^+$([M+Na]$^+$) = 334.1941, found = 334.1951.

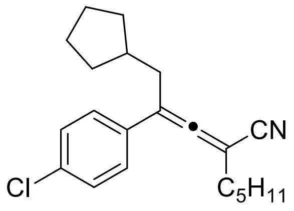

3e

2-(2-(4-chlorophenyl)-(3-cyclopentylprop-1-en-1-ylidene)heptanenitrile (3e): Pale yellow oil, 54.2 mg, isolated yield 83%. ^{1}H NMR (400 MHz, $CDCl_3$) δ 7.34 – 7.31 (m, 2H), 7.28 – 7.25 (m, 2H), 2.49 (d, J = 7.2 Hz, 2H), 2.31 – 2.27 (m, 2H), 2.06 – 2.00 (m, 1H), 1.86 – 1.76 (m, 2H), 1.65 – 1.53 (m, 6H), 1.37 – 1.29 (m, 4H), 1.26 – 1.17 (m, 2H), 0.90 – 0.86 (m, 3H); ^{13}C NMR (100 MHz, $CDCl_3$) δ 212.6, 134.3, 132.5, 129.1, 128.1, 115.6, 110.8, 85.2, 38.1, 37.1, 32.9, 32.9, 31.9, 31.1, 27.6, 25.4, 25.4, 22.4, 14.1. HRMS (ESI): m/z calcd. for $C_{21}H_{26}ClNNa^{+}$($[M+Na]^{+}$) = 350.1646, found = 350.1645.

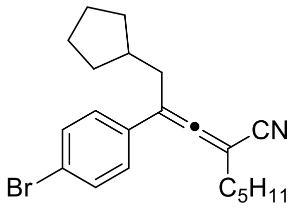

3f

2-(2-(4-bromophenyl)-(3-cyclopentylprop-1-en-1-ylidene)heptanenitrile (3f): Pale yellow oil, 57.9 mg, isolated yield 78%. ^{1}H NMR (400 MHz, $CDCl_3$) δ 7.48 (d, J = 8.4 Hz, 2H), 7.20 (d, J = 8.4 Hz, 2H), 2.49 (d, J = 7.6 Hz, 2H), 2.31 – 2.27 (m, 2H), 2.06 – 1.98 (m, 1H), 1.85 – 1.78 (m, 2H), 1.67 – 1.51 (m, 6H), 1.35– 1.31 (m, 4H), 1.24 – 1.18 (m, 2H), 0.88 (t, J = 7.0 Hz, 3H); ^{13}C NMR (100 MHz, $CDCl_3$) δ 212.6, 133.0, 132.0, 128.4, 122.4, 115.5, 110.9, 85.2, 38.1, 37.0, 32.9, 32.9, 31.9, 31.1, 27.6, 25.4, 25.4, 22.4, 14.1. HRMS (ESI): m/z calcd. for $C_{21}H_{26}BrNNa^{+}$($[M+Na]^{+}$) = 394.1141, found = 394.1150.

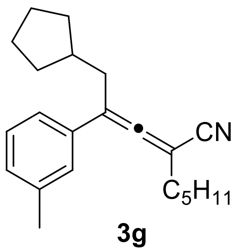

3g

2-(3-cyclopentyl-2-(*m*-tolyl)prop-1-en-1-ylidene)heptanenitrile (3g): Pale yellow oil, 45.8 mg, isolated yield 75%. ^{1}H NMR (400 MHz, $CDCl_3$) δ 7.25 – 7.22 (m, 1H), 7.15 – 7.09 (m, 3H), 2.50 (d, J = 7.2 Hz, 2H), 2.36 (s, 3H), 2.28 (t, J = 7.6 Hz, 2H), 2.07 – 2.00 (m, 1H), 1.88 – 1.76 (m, 2H), 1.65 – 1.50 (m, 6H), 1.38 – 1.17 (m, 6H), 0.89 – 0.86 (m, 3H); ^{13}C NMR (100 MHz, $CDCl_3$) δ 212.8, 138.5, 133.9, 129.2, 128.7, 127.5, 123.9, 115.9, 111.6, 84.5, 38.2, 37.2, 32.9, 32.9, 32.0, 31.1, 27.6, 25.4, 25.4, 22.4, 21.6, 14.1. HRMS (ESI): m/z calcd. for

$C_{22}H_{29}NNa^{+}$([M+Na]$^{+}$) = 330.2192, found = 330.2189.

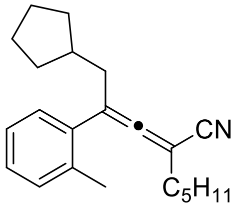

3h

2-(3-cyclopentyl-2-(*o*-tolyl)prop-1-en-1-ylidene)heptanenitrile (3h): Pale yellow oil, 25.0 mg, isolated yield 41%. ^{1}H NMR (400 MHz, $CDCl_3$) δ 7.22 – 7.17 (m, 4H), 2.42 – 2.39 (m, 2H), 2.36 (s, 3H), 2.22 –2.18 (m, 2H), 1.98 – 1.92 (m, 1H), 1.85 – 1.78 (m, 2H), 1.65 – 1.60 (m, 2H), 1.59 – 1.51 (m, 4H), 1.33 – 1.26 (m, 4H), 1.25 – 1.17 (m, 2H), 0.88 – 0.84 (m, 3H); ^{13}C NMR (100 MHz, $CDCl_3$) δ 210.2, 135.9, 134.9, 130.9, 128.1, 128.0, 126.2, 116.1, 110.5, 82.0, 40.9, 38.0, 32.9, 32.9, 31.6, 31.0, 27.5, 25.5, 25.5, 22.4, 20.4, 14.1. HRMS (ESI): m/z calcd. for $C_{22}H_{29}NNa^{+}$([M+Na]$^{+}$) = 330.2192, found = 330.2194.

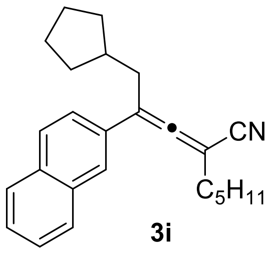

3i

2-(3-cyclopentyl-2-(naphthalen-2-yl)prop-1-en-1-ylidene)heptanenitrile (3i): Pale yellow oil, 38.2 mg, isolated yield 56%. ^{1}H NMR (400 MHz, $CDCl_3$) δ 7.85 – 7.78 (m, 4H), 7.52 – 7.44 (m, 3H), 2.66 (d, *J* = 7.2 Hz, 2H), 2.36 – 2.32 (m, 2H), 2.17 – 2.09 (m, 1H), 1.88 – 1.84 (m, 2H), 1.70 – 1.55 (m, 6H), 1.42 – 1.24 (m, 6H), 0.89 (t, *J* = 7.0 Hz, 3H); ^{13}C NMR (100 MHz, $CDCl_3$) δ 213.4, 133.5, 133.2, 131.2, 128.5, 128.3, 127.8, 126.7, 126.6, 125.3, 125.1, 115.9, 111.8, 85.0, 38.3, 37.1, 33.0, 32.1, 31.1, 27.6, 25.4, 25.4, 22.4, 14.1. HRMS (ESI): m/z calcd. for $C_{25}H_{29}NNa^{+}$([M+Na]$^{+}$) = 366.2192, found = 366.2196.

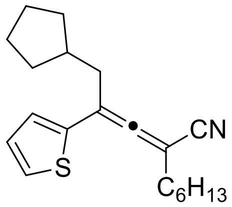

3j

2-(3-cyclopentyl)-2-(thiophen-2-yl)-prop-1-en-1-ylidene)octanenitrile (3j): Pale yellow oil, 31.4 mg, isolated yield 50%. ^{1}H NMR (500 MHz, $CDCl_3$) δ 7.28 (dd, *J* = 5.0, 1.5 Hz, 1H), 7.03 – 7.00 (m, 2H), 2.52 – 2.50 (m, 2H), 2.31 – 2.28 (m, 2H), 2.14 – 2.08 (m, 1H), 1.86 – 1.82 (m, 2H), 1.66 – 1.53 (m, 6H), 1.41 – 1.19 (m, 8H), 0.89 – 0.87 (m, 3H); ^{13}C NMR (125 MHz, $CDCl_3$) δ 212.4, 137.9,

127.8, 126.3, 125.3, 115.3, 107.1, 85.5, 38.4, 38.2, 32.9, 32.9, 32.3, 31.6, 28.6, 27.9, 25.4, 22.7, 14.2. HRMS (ESI): m/z calcd. for $C_{20}H_{27}NNaS^+$([M+Na]$^+$) = 336.1756, found = 336.1759.

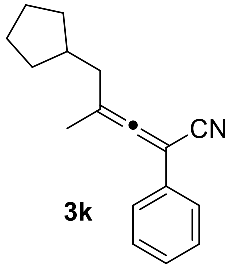

3k

5-cyclopentyl-4-methyl-2-phenylpenta-2,3-dienenitrile (3k): Pale yellow oil, 21.0 mg, isolated yield 44%. ^{1}H NMR (400 MHz, $CDCl_3$) δ 7.47 – 7.44 (m, 2H), 7.41 – 7.36 (m, 2H), 7.32 – 7.28 (m, 1H), 2.21 (t, *J* = 7.6 Hz, 2H), 2.07 – 2.00 (m, 1H), 1.92 (s, 3H), 1.88 –1.83 (m, 1H), 1.79 – 1.74 (m, 1H), 1.63 – 1.49 (m, 6H); ^{13}C NMR (100 MHz, $CDCl_3$) δ 209.7, 130.8, 129.0, 128.4, 125.7, 115.1, 110.3, 85.2, 40.8, 37.8, 33.0, 25.5, 18.4. HRMS (ESI): m/z calcd. for $C_{17}H_{19}NNa^+$([M+Na]$^+$) = 260.1410, found = 260.1412.

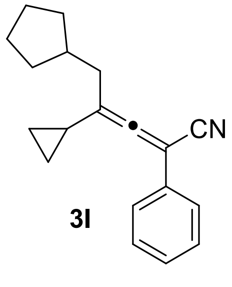

3l

5-cyclopentyl-4-cyclopropyl-2-phenylpenta-2,3-dienenitrile (3l): Colorless oil, 22.8 mg, isolated yield 43%. ^{1}H NMR (500 MHz, $CDCl_3$) δ 7.44 – 7.41 (m, 2H), 7.39 – 7.36 (m, 2H), 7.32 – 7.29 (m, 1H), 2.34 – 2.25 (m, 2H), 2.12 – 2.06 (m, 1H), 1.89 – 1.83 (m, 1H), 1.80 – 1.74 (m, 1H), 1.63 – 1.57 (m, 2H), 1.55 – 1.51 (m, 2H), 1.35 – 1.30 (m, 1H), 1.21 – 1.15 (m, 2H), 0.86 – 0.82 (m, 2H), 0.57 – 0.51 (m, 2H); ^{13}C NMR (100 MHz, $CDCl_3$) δ 209.5, 130.7, 129.1, 128.5, 125.5, 119.0, 114.9, 87.7, 39.6, 38.1, 33.1, 33.0, 25.5, 12.7, 7.6, 7.5. HRMS (ESI): m/z calcd. for $C_{19}H_{21}NNa^+$([M+Na]$^+$) = 286.1566, found = 286.1573.

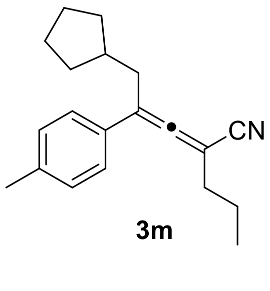

3m

5-cyclopentyl-2-propyl-4-(*p*-tolyl)penta-2,3-dienenitrile (3m): Colorless oil, 50.2 mg, isolated yield 90%. ^{1}H NMR (500 MHz, $CDCl_3$) δ 7.24 (d, *J* = 8.5 Hz, 2H), 7.17 (d, *J* = 8.0 Hz, 2H), 2.51 (d, *J* = 7.0 Hz, 2H), 2.36 (s, 3H), 2.29 – 2.26 (m, 2H), 2.08 – 2.02 (m, 1H), 1.86 – 1.78 (m, 2H), 1.65 – 1.53 (m, 6H), 1.26 – 1.19 (m, 2H), 0.99 (t, *J* = 7.5 Hz, 3H); ^{13}C NMR (125 MHz, $CDCl_3$) δ 212.9,

138.4, 130.9, 129.6, 126.7, 115.9, 111.4, 84.3, 38.2, 37.1, 34.0, 32.9, 25.4, 25.4, 21.3, 21.3, 13.5. HRMS (ESI): m/z calcd. for $C_{20}H_{25}NNa^+$([M+Na]$^+$) = 302.1879, found = 302.1886.

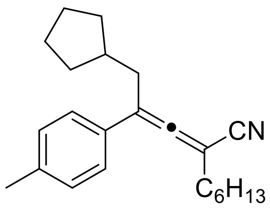

3n

2-(3-cyclopentyl-2-(*p*-tolyl)prop-1-en-1-ylidene)octanenitrile (3n): Colorless oil, 54.0 mg, isolated yield 84%. ^{1}H NMR (400 MHz, $CDCl_3$) δ 7.16 (d, *J* = 8.4 Hz, 2H), 7.09 (d, *J* = 8.0 Hz, 2H), 2.43 (d, *J* = 7.2 Hz, 2H), 2.28 (s, 3H), 2.22 – 2.19 (m, 2H), 2.01 – 1.93 (m, 1H), 1.75 – 1.69 (m, 2H), 1.59 – 1.44 (m, 6H), 1.30 – 1.12 (m, 8H), 0.82 – 0.78 (m, 3H); ^{13}C NMR (100 MHz, $CDCl_3$) δ 212.8, 138.4, 131.0, 129.6, 126.7, 116.0, 111.5, 84.5, 38.2, 37.1, 32.9, 32.1, 31.6, 28.6, 27.9, 25.4, 25.4, 22.7, 21.3, 14.1. HRMS (ESI): m/z calcd. for $C_{23}H_{31}NNa^+$([M+Na]$^+$) = 344.2349, found = 344.2351.

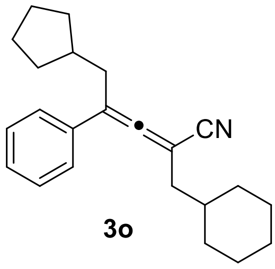

3o

2-(cyclohexylmethyl)-5-cyclopentyl-4-phenylpenta-2,3-dienenitrile (3o): Colorless oil, 45.9 mg, isolated yield 72%. ^{1}H NMR (500 MHz, $CDCl_3$) δ 7.38 – 7.33 (m, 4H), 7.31 – 7.28 (m, 1H), 2.52 (d, *J* = 7.5 Hz, 2H), 2.17 (dd, *J* = 7.3, 1.8 Hz, 2H), 2.08 – 2.02 (m, 1H), 1.85 – 1.79 (m, 4H), 1.74 – 1.53 (m, 8H), 1.32 – 1.13 (m, 5H), 1.00 – 0.92 (m, 2H); ^{13}C NMR (125 MHz, $CDCl_3$) δ 213.4, 134.0, 128.9, 128.3, 126.9, 116.0, 110.9, 83.3, 39.7, 38.2, 37.1, 36.7, 33.0, 32.9, 32.9, 32.9, 26.4, 26.1, 25.4, 25.3. HRMS (ESI): m/z calcd. for $C_{23}H_{29}NNa^+$([M+Na]$^+$) = 342.2192, found = 342.2200.

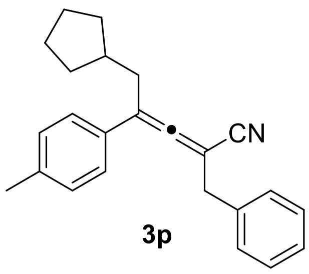

3p

2-benzyl-5-cyclopentyl-4-(*p*-tolyl)penta-2,3-dienenitrile (3p): Colorless oil, 42.0 mg, isolated yield 64%. ^{1}H NMR (400 MHz, $CDCl_3$) δ 7.35 – 7.31 (m, 2H), 7.29 – 7.25 (m, 3H), 7.19 – 7.14 (m, 4H), 3.59 (s, 2H), 2.47 (d, *J* = 7.2 Hz, 2H), 2.36 (s, 3H), 2.02 – 1.94 (m, 1H), 1.81 – 1.74 (m, 2H),

1.64 – 1.60 (m, 2H), 1.53 – 1.49 (m, 2H), 1.21 – 1.14 (m, 2H); ^{13}C NMR (100 MHz, $CDCl_3$) δ 213.4, 138.6, 136.7, 130.6, 129.6, 129.0, 128.9, 127.4, 126.8, 115.7, 112.3, 84.3, 38.6, 38.2, 37.1, 33.0, 32.8, 25.4, 25.3, 21.3. HRMS (ESI): m/z calcd. for $C_{24}H_{25}NNa^+$($[M+Na]^+$) = 350.1879, found = 350.1873.

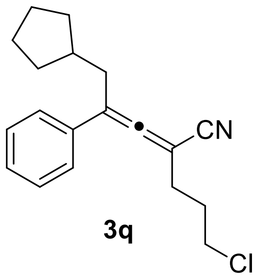

3q

2-(3-chloropropyl)-5-cyclopentyl-4-phenylpenta-2,3-dienenitrile (3q): Colorless oil, 46.0 mg, isolated yield 77%. ^{1}H NMR (500 MHz, $CDCl_3$) δ 7.40 – 7.30 (m, 5H), 3.59 (t, J = 6.3 Hz, 2H), 2.55 (d, J = 7.5 Hz, 2H), 2.51 – 2.48 (m, 2H), 2.08 – 2.03 (m, 3H), 1.84 – 1.83 (m, 2H), 1.66 – 1.63 (m, 2H), 1.59 – 1.53 (m, 2H), 1.27 – 1.21 (m, 2H); ^{13}C NMR (125 MHz, $CDCl_3$) δ 213.0, 133.6, 129.0 , 128.6, 126.8, 115.4, 112.5, 83.2, 43.6, 38.1, 37.2, 32.9, 30.5, 29.2, 25.4, 25.4. HRMS (ESI): m/z calcd. for $C_{19}H_{22}ClNNa^+$($[M+Na]^+$) = 322.1333, found = 322.1341.

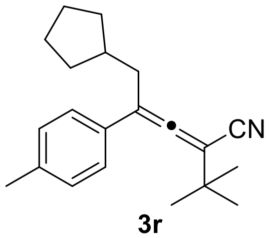

3r

2-(*tert*-butyl)-5-cyclopentyl-4-(*p*-tolyl)penta-2,3-dienenitrile (3r): White solid, 45.1 mg, isolated yield 77%. ^{1}H NMR (400 MHz, $CDCl_3$) δ 7.24 (d, J = 8.4 Hz, 2H), 7.18 – 7.16 (m, 2H), 2.52 (dd, J = 7.2, 4.4 Hz, 2H), 2.36 (s, 3H), 2.06 – 2.00 (m, 1H), 1.87 – 1.81 (m, 2H), 1.67 – 1.60 (m, 2H), 1.59 – 1.51 (m, 2H), 1.23 (s, 11H); ^{13}C NMR (100 MHz, $CDCl_3$) δ 210.3, 138.3, 131.1, 129.6, 126.5, 115.1, 112.8, 95.8, 38.3, 37.7, 35.3, 33.2, 33.0, 29.1, 25.5, 25.3, 21.3. HRMS (ESI): m/z calcd. for $C_{21}H_{27}NNa^+$($[M+Na]^+$) = 316.2036, found = 316.2044.

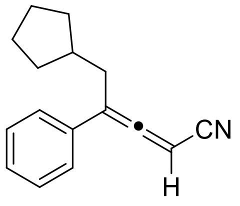

3s

5-cyclopentyl-4-phenylpenta-2,3-dienenitrile (3s): Pale yellow oil, 33.1 mg, isolated yield 74%. ^{1}H NMR (400 MHz, $CDCl_3$) δ 7.41 – 7.30 (m, 5H), 5.55 (t, J = 3.0 Hz, 1H), 2.57 – 2.53 (m, 2H), 2.11 – 2.04 (m, 1H), 1.87 – 1.81 (m, 2H), 1.67 – 1.53 (m, 4H), 1.26 – 1.21 (m, 2H); ^{13}C NMR (100 MHz, $CDCl_3$) δ 217.3, 132.8, 129.0, 128.7, 126.9, 113.6, 112.2, 69.7, 38.1, 36.8, 32.9, 32.8, 25.4, 25.4. HRMS (ESI):

m/z calcd. for $C_{16}H_{17}NNa^+([M+Na]^+)$ = 246.1253, found = 246.1258.

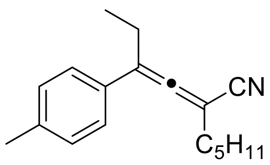

4a

2-(2-(*p*-tolyl)but-1-en-1-ylidene)heptanenitrile (4a): Pale yellow oil, 42.0 mg, isolated yield 83%. ^1H NMR (500 MHz, $CDCl_3$) δ 7.25 – 7.23 (m, 2H), 7.18 – 7.16 (m, 2H), 2.53 (q, *J* = 7.3 Hz, 2H), 2.36 (s, 3H), 2.29 (t, *J* = 7.8 Hz, 2H), 1.61 – 1.55 (m, 2H), 1.37 – 1.31 (m, 4H), 1.16 (t, *J* = 7.3 Hz, 3H), 0.88 (t, *J* = 7.0 Hz, 3H); ^{13}C NMR (125 MHz, $CDCl_3$) δ 212.1, 138.4, 130.9, 129.6, 126.6, 116.1, 113.8, 85.7, 32.0, 31.1, 27.6, 23.5, 22.4, 21.3, 14.1, 12.3. HRMS (ESI): m/z calcd. for $C_{18}H_{23}NNa^+([M+Na]^+)$ = 276.1723, found = 276.1726.

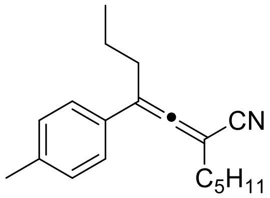

4b

2-pentyl-4-(*p*-tolyl)hepta-2,3-dienenitrile (4b): Pale yellow oil, 42.1 mg, isolated yield 79%. ^1H NMR (400 MHz, $CDCl_3$) δ 7.25 – 7.22 (m, 2H), 7.18 – 7.16 (m, 2H), 2.48 (t, *J* = 7.6 Hz, 2H), 2.36 (s, 3H), 2.28 (t, *J* = 7.6 Hz, 2H), 1.60 – 1.54 (m, 4H), 1.36 – 1.31 (m, 4H), 1.01 (t, *J* = 7.4 Hz, 3H), 0.88 (t, *J* = 7.2 Hz, 3H); ^{13}C NMR (100 MHz, $CDCl_3$) δ 212.3, 138.4, 130.8, 129.6, 126.7, 116.0, 111.8, 84.9, 32.4, 32.0, 31.1, 27.6, 22.4, 21.3, 21.0, 14.1, 14.0. HRMS (ESI): m/z calcd. for $C_{19}H_{25}NNa^+([M+Na]^+)$ = 290.1879, found = 290.1878.

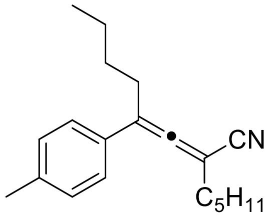

4c

2-pentyl-4-(*p*-tolyl)octa-2,3-dienenitrile (4c): Pale yellow oil, 46.1 mg, isolated yield 82%. ^1H NMR (400 MHz, $CDCl_3$) δ 7.23 (d, *J* = 8.0 Hz, 2H), 7.17 (d, *J* = 8.0 Hz, 2H), 2.50 (t, *J* = 7.4 Hz, 2H), 2.36 (s, 3H), 2.30 – 2.26 (m, 2H), 1.60 – 1.50 (m, 4H), 1.45 – 1.39 (m, 2H), 1.36 – 1.31 (m, 4H), 0.94 (t, *J* = 7.4 Hz, 3H), 0.88 (t, *J* = 7.0 Hz, 3H); ^{13}C NMR (100 MHz, $CDCl_3$) δ 212.3, 138.4, 130.9, 129.6, 126.7, 116.0, 112.0, 84.9, 32.0, 31.1, 30.0, 29.9, 27.6, 22.5, 22.4, 21.3, 14.1, 14.0. HRMS (ESI): m/z calcd. for $C_{20}H_{27}NNa^+([M+Na]^+)$ = 304.2036, found = 304.2042.

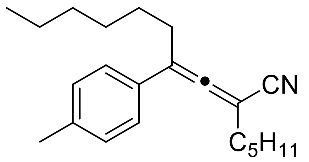

4d

2-pentyl-4-(*p*-tolyl)deca-2,3-dienenitrile (4d): Pale yellow oil, 45.1 mg, isolated yield 73%. ^{1}H NMR (400 MHz, $CDCl_3$) δ 7.23 (d, *J* = 8.4 Hz, 2H), 7.17 (d, *J* = 7.6 Hz, 2H), 2.49 (t, *J* = 7.4 Hz, 2H), 2.36 (s, 3H), 2.28 (t, *J* = 7.6 Hz, 2H), 1.60 – 1.50 (m, 4H), 1.41 – 1.29 (m, 10H), 0.91 – 0.86 (m, 6H); ^{13}C NMR (100 MHz, $CDCl_3$) δ 212.3, 138.4, 130.9, 129.6, 126.7, 116.0, 112.0, 84.9, 32.0, 31.8, 31.1, 30.3, 29.1, 27.7, 27.6, 22.8, 22.5, 21.3, 14.2, 14.1. HRMS (ESI): m/z calcd. for $C_{22}H_{31}NNa^+$([M+Na]$^+$) = 332.2349, found = 332.2348.

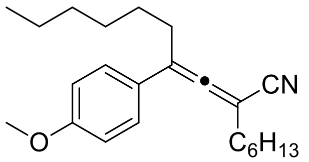

4e

2-hexyl-4-(4-methoxyphenyl)deca-2,3-dienenitrile (4e): Colorless oil, 46.7 mg, isolated yield 69%. ^{1}H NMR (400 MHz, $CDCl_3$) δ 7.27 (d, *J* = 8.8 Hz, 2H), 6.89 (d, *J* = 8.8 Hz, 2H), 3.82 (s, 3H), 2.48 (t, *J* = 7.4 Hz, 2H), 2.28 (t, *J* = 7.4 Hz, 2H), 1.60 – 1.52 (m, 4H), 1.39 – 1.26 (m, 12H), 0.92 – 0.86 (m, 6H); ^{13}C NMR (100 MHz, $CDCl_3$) δ 212.2, 159.8, 128.0, 125.9, 116.1, 114.3, 111.7, 84.9, 55.5, 32.1, 31.8, 31.6, 30.4, 29.1, 28.6, 27.9, 27.7, 22.8, 22.7, 14.2, 14.1. These data matches with reported values.[2d]

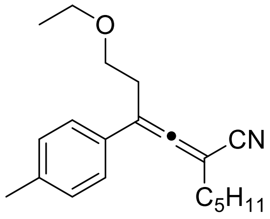

4f

2-(4-ethoxy-2-(*p*-tolyl)but-1-en-1-ylidene)heptanenitrile (4f): Pale yellow oil, 39.8 mg, isolated yield 67%. ^{1}H NMR (400 MHz, $CDCl_3$) δ 7.24 (d, *J* = 8.4 Hz, 2H), 7.17 (d, *J* = 8.0 Hz, 2H), 3.60 (t, *J* = 6.8 Hz, 2H), 3.50 (q, *J* = 7.1 Hz, 2H), 2.78 (t, *J* = 6.6 Hz, 2H), 2.35 (s, 3H), 2.29 (t, *J* = 7.6 Hz, 2H), 1.61 – 1.57 (m, 2H), 1.34 – 1.32 (m, 4H), 1.21 (t, *J* = 7.0 Hz, 3H), 0.90 – 0.86 (m, 3H); ^{13}C NMR (100 MHz, $CDCl_3$) δ 212.3 , 138.5, 130.5, 129.6, 126.6, 115.8, 108.9, 85.3, 68.3, 66.6, 32.0, 31.1, 30.7, 27.5, 22.4, 21.3, 15.3, 14.1. HRMS (ESI): m/z calcd. for $C_{20}H_{27}NONa^+$([M+Na]$^+$) = 320.1985, found = 320.1992.

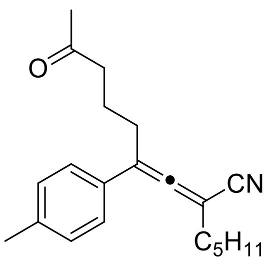

4g

8-oxo-2-pentyl-4-(*p*-tolyl)nona-2,3-dienenitrile (4g): Colorless oil, 26.0 mg, isolated yield 42%. ^{1}H NMR (400 MHz, $CDCl_3$) δ 7.24 – 7.21 (m, 2H), 7.18 – 7.16 (m, 2H), 2.55 – 2.51 (m, 4H), 2.35 (s, 3H), 2.30 – 2.26 (m, 2H), 2.16 (s, 3H), 1.86 – 1.78 (m, 2H), 1.60 – 1.55 (m, 2H), 1.35 – 1.31 (m, 4H), 0.89 – 0.86 (m, 3H); ^{13}C NMR (100 MHz, $CDCl_3$) δ 212.1, 208.2, 138.6, 130.3, 129.7, 126.7, 115.8, 111.4, 85.4, 42.6, 32.0, 31.1, 30.2, 29.5, 27.6, 22.4, 21.6, 21.3, 14.1. HRMS (ESI): m/z calcd. for $C_{21}H_{27}NONa^+$($[M+Na]^+$) = 332.1985, found = 332.1988.

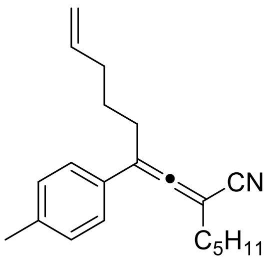

4h

2-pentyl-4-(*p*-tolyl)nona-2,3,8-trienenitrile (4h): Colorless oil, 47.6 mg, isolated yield 81%. ^{1}H NMR (400 MHz, $CDCl_3$) δ 7.23 (d, J = 8.4 Hz, 2H), 7.17 (d, J = 8.0 Hz, 2H), 5.87 – 5.76 (m, 1H), 5.08 – 4.99 (m, 2H), 2.53 – 2.50 (m, 2H), 2.36 (s, 3H), 2.30 – 2.26 (m, 2H), 2.19 – 2.13 (m, 2H), 1.68 – 1.54 (m, 4H), 1.37 – 1.29 (m, 4H), 0.90 – 0.87 (m, 3H); ^{13}C NMR (100 MHz, $CDCl_3$) δ 212.2, 138.5, 138.0, 130.8, 129.6, 126.7, 115.9, 115.4, 111.8, 85.1, 33.4, 32.0, 31.1, 29.6, 27.6, 26.9, 22.4, 21.3, 14.1. HRMS (ESI): m/z calcd. for $C_{21}H_{27}NNa^+$($[M+Na]^+$) = 316.2036, found = 316.2024.

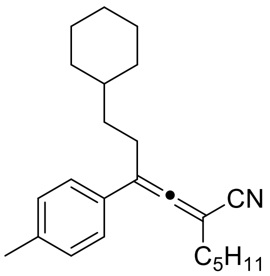

4i

2-(4-cyclohexyl-2-(*p*-tolyl)but-1-en-1-ylidene)heptanenitrile (4i): Pale yellow oil, 57.0 mg, isolated yield 85%. ^{1}H NMR (400 MHz, $CDCl_3$) δ 7.23 (d, J = 8.4 Hz, 2H), 7.17 (d, J = 8.0 Hz, 2H), 2.51 (t, J = 7.8 Hz, 2H), 2.36 (s, 3H), 2.28 (t, J = 7.6 Hz, 2H), 1.75 – 1.69 (m, 5H), 1.60 – 1.56 (m, 2H), 1.43 – 1.38 (m, 2H), 1.37 – 1.14 (m, 8H), 0.98 – 0.87 (m, 5H); ^{13}C NMR (100 MHz, $CDCl_3$) δ 212.3, 138.4, 130.9, 129.6, 126.6, 116.0, 112.2, 84.9, 37.4, 35.3, 33.4, 33.4, 32.0, 31.1, 27.7, 27.6, 26.7, 26.5, 22.5, 21.3, 14.1. HRMS (ESI): m/z calcd. for $C_{24}H_{33}NNa^+$($[M+Na]^+$) = 358.2505, found = 358.2508.

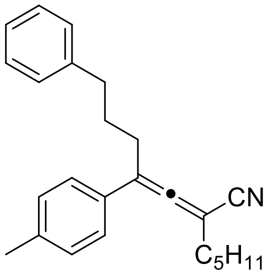

4j

2-pentyl-7-phenyl-4-(*p*-tolyl)hepta-2,3-dienenitrile (4j): Pale yellow oil, 57.0 mg, isolated yield 83%. ^{1}H NMR (400 MHz, $CDCl_3$) δ 7.33 – 7.29 (m, 2H), 7.23 – 7.16 (m, 7H), 2.73 (t, *J* = 7.6 Hz, 2H), 2.54 (t, *J* = 8.2 Hz, 2H), 2.36 (s, 3H), 2.29 (t, *J* = 7.6 Hz, 2H), 1.93 – 1.84 (m, 2H), 1.61 – 1.55 (m, 2H), 1.35 – 1.30 (m, 4H), 0.88 (t, *J* = 7.0 Hz, 3H); ^{13}C NMR (100 MHz, $CDCl_3$) δ 212.2, 141.7, 138.5, 130.7, 129.6, 128.6, 128.6, 126.6, 126.1, 115.9, 111.8, 85.2, 35.5, 32.0, 31.1, 29.7, 29.3, 27.6, 22.4, 21.3, 14.1. HRMS (ESI): m/z calcd. for $C_{25}H_{29}NNa^+$($[M+Na]^+$) = 366.2192, found = 366.2199.

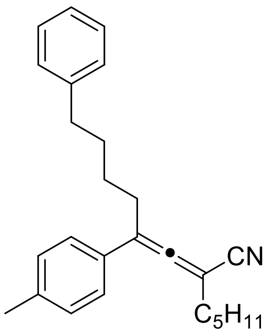

4k

2-pentyl-8-phenyl-4-(*p*-tolyl)octa-2,3-dienenitrile (4k): Pale yellow oil, 62.1 mg, isolated yield 87%. ^{1}H NMR (400 MHz, $CDCl_3$) δ 7.23 – 7.08 (m, 9H), 2.58 (t, *J* = 7.6 Hz, 2H), 2.45 (t, *J* = 7.4 Hz, 2H), 2.28 (s, 3H), 2.18 (t, *J* = 7.6 Hz, 2H), 1.69 – 1.62 (m, 2H), 1.55 – 1.44 (m, 4H), 1.27 – 1.19 (m, 4H), 0.81 (t, *J* = 7.0 Hz, 3H); ^{13}C NMR (100 MHz, $CDCl_3$) δ 212.1, 142.3, 138.4, 130.8, 129.6, 128.5, 128.4, 126.6, 125.9, 115.9, 111.9, 85.1, 35.8, 32.0, 31.1, 31.1, 30.1, 27.6, 27.3, 22.4, 21.3, 14.1. HRMS (ESI): m/z calcd. for $C_{26}H_{31}NNa^+$($[M+Na]^+$) = 380.2349, found = 380.2356.

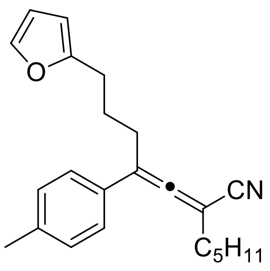

4l

7-(furan-2-yl)-2-pentyl-4-(*p*-tolyl)hepta-2,3-dienenitrile (4l): Pale yellow oil, 53.2 mg, isolated yield 80%. ^{1}H NMR (500 MHz, $CDCl_3$) δ 7.32 – 7.31 (m, 1H), 7.23 – 7.21 (m, 2H), 7.17 (d, *J* = 8.5 Hz, 2H), 6.30– 6.29 (m, 1H), 6.03 – 6.02 (m, 1H), 2.75 – 2.72 (m, 2H), 2.57 – 2.53 (m, 2H), 2.36 (s, 3H), 2.30 – 2.27 (m, 2H), 1.91 – 1.87 (m, 2H), 1.60 – 1.55 (m, 2H), 1.35 – 1.29 (m, 4H), 0.88 (t, *J* = 7.3 Hz, 3H); ^{13}C NMR (125 MHz, $CDCl_3$) δ 212.1, 155.3, 141.2, 138.5, 130.6, 129.6, 126.6, 115.9, 111.6, 110.3, 105.4, 85.3, 32.0, 31.1, 29.6,

27.6, 27.5, 26.2, 22.4, 21.3, 14.0. HRMS (ESI): m/z calcd. for $C_{23}H_{27}NONa^+$([M+Na]$^+$) = 356.1985, found = 356.1986.

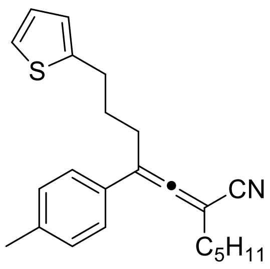

4m

2-pentyl-7-(thiophen-2-yl)- 4-(*p*-tolyl)hepta-2,3-dienenitrile (4m): Pale yellow oil, 57.2 mg, isolated yield 82%. ^{1}H NMR (500 MHz, $CDCl_3$) δ 7.23 – 7.21 (m, 2H), 7.19 – 7.17 (m, 2H), 7.14 (dd, *J* = 5.0, 1.0 Hz, 1H), 6.94 (dd, *J* = 5.0, 3.5 Hz, 1H), 6.83 – 6.82 (m, 1H), 2.97 – 2.93 (m, 2H), 2.58 (t, *J* = 7.5 Hz, 2H), 2.37 (s, 3H), 2.31 – 2.28 (m, 2H), 1.97 – 1.90 (m, 2H), 1.61 – 1.56 (m, 2H), 1.37 – 1.30 (m, 4H), 0.88 (t, *J* = 7.0 Hz, 3H); ^{13}C NMR (125 MHz, $CDCl_3$) δ 212.1, 144.4, 138.5, 130.6, 129.6, 126.9, 126.6, 124.6, 123.3, 115.9, 111.5, 85.3, 32.0, 31.1, 29.7, 29.4, 27.6, 22.4, 21.3, 14.0. HRMS (ESI): m/z calcd. for $C_{23}H_{27}NSNa^+$([M+Na]$^+$) = 372.1756, found = 372.1766.

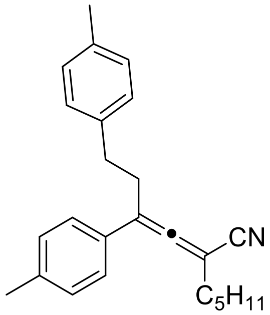

4n

2-(2,4-di-*p*-tolybut-1-en-1-ylidene)heptanenitrile (4n): Pale yellow oil, 20.6 mg, isolated yield 30%. ^{1}H NMR (400 MHz, $CDCl_3$) δ 7.25 – 7.23 (m, 2H), 7.19 – 7.17 (m, 2H), 7.13 – 7.07 (m, 4H), 2.85 – 2.76 (m, 4H), 2.37 (s, 3H), 2.33 (s, 3H), 2.16 – 2.12 (m, 2H), 1.51 – 1.44 (m, 2H), 1.31 – 1.27 (m, 4H), 0.90 – 0.86 (m, 3H); ^{13}C NMR (100 MHz, $CDCl_3$) δ 212.2, 138.5, 137.9, 135.9, 130.6, 129.7, 129.3, 128.4, 126.7, 115.8, 111.3, 85.3, 33.4, 32.1, 31.9, 31.1, 27.6, 22.4, 21.3, 21.2, 14.1. HRMS (ESI): m/z calcd. for $C_{25}H_{29}NNa^+$([M+Na]$^+$) = 366.2192, found = 366.2199.

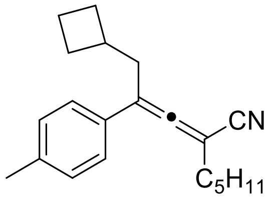

4o

2-(3-cyclobutyl-2-(*p*-tolyl)prop-1-en-1-ylidene)heptanenitrile (4o): Colorless oil, 48.0 mg, isolated yield 82%. ^{1}H NMR (500 MHz, $CDCl_3$) δ 7.22 (d, *J* = 8.0 Hz, 2H), 7.16 (d, *J* = 8.0 Hz, 2H), 2.61 – 2.59 (m, 2H), 2.54 – 2.47 (m, 1H), 2.35 (s, 3H), 2.28 – 2.25 (m, 2H), 2.18 – 2.08 (m, 2H),

1.91 – 1.86 (m, 2H), 1.74 – 1.69 (m, 2H), 1.61 – 1.55 (m, 2H), 1.36 – 1.30 (m, 4H), 0.89 (t, *J* = 7.0 Hz, 3H); ^{13}C NMR (125 MHz, $CDCl_3$) δ 212.5, 138.4, 130.9, 129.6, 126.6, 116.0, 110.4, 84.8, 37.5, 34.1, 32.1, 31.2, 28.5, 28.4, 27.6, 22.4, 21.3, 18.5, 14.1. HRMS (ESI): m/z calcd. for $C_{21}H_{27}NNa^+$([M+Na]$^+$) = 316.2036, found = 316.2034.

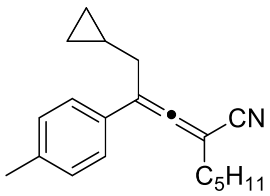

4p

2-（3-cyclopropyl-2-(*p*-tolyl)prop-1-en-1-ylidene）heptanenitrile (4p): Colorless oil, 42.6 mg, isolated yield 76%. ^{1}H NMR (500 MHz, $CDCl_3$) δ 7.23 (d, *J* = 8.0 Hz, 2H), 7.17 (d, *J* = 8.0 Hz, 2H), 2.43 – 2.41 (m, 2H), 2.36 (s, 3H), 2.30 (t, *J* = 7.8 Hz, 2H), 1.61 – 1.58 (m, 2H), 1.36 – 1.32 (m, 4H), 0.94 – 0.91 (m, 1H), 0.88 (t, *J* = 7.0 Hz, 3H), 0.59 – 0.53 (m, 2H), 0.24 – 0.16 (m, 2H); ^{13}C NMR (100 MHz, $CDCl_3$) δ 212.9, 138.4, 130.9, 129.6, 126.6, 116.1, 112.1, 85.3, 35.5, 32.1, 31.2, 27.6, 22.4, 21.3, 14.1, 9.4, 5.2, 5.1. HRMS (ESI): m/z calcd. for $C_{20}H_{25}NNa^+$([M+Na]$^+$) = 302.1879, found = 302.1878.

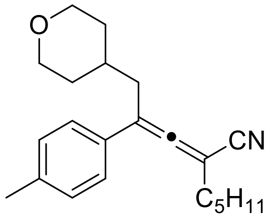

4q

2-(3-(tetrahydro-2*H*-pyran-4-yl)-2-(*p*-tolyl)prop-1-en-1-ylidene)heptanenitrile (4q): Colorless oil, 41.6 mg, isolated yield 64%. ^{1}H NMR (400 MHz, $CDCl_3$) δ 7.22 (d, *J* = 8.4 Hz, 2H), 7.18 (d, *J* = 8.0 Hz, 2H), 3.98 – 3.94 (m, 2H), 3.35 – 3.33 (m, 2H), 2.51 – 2.40 (m, 2H), 2.36 (s, 3H), 2.29 – 2.26 (m, 2H), 1.73 – 1.65 (m, 3H), 1.62 – 1.56 (m, 2H), 1.38 – 1.30 (m, 6H), 0.90 – 0.86 (m, 3H); ^{13}C NMR (100 MHz, $CDCl_3$) δ 212.4, 138.6, 130.5, 129.7, 126.7, 115.7, 109.3, 84.4, 68.0, 67.9, 37.9, 33.7, 33.3, 33.1, 32.0, 31.1, 27.6, 22.4, 21.3, 14.1. HRMS (ESI): m/z calcd. for $C_{22}H_{29}NONa^+$([M+Na]$^+$) = 346.2141, found = 346.2150.

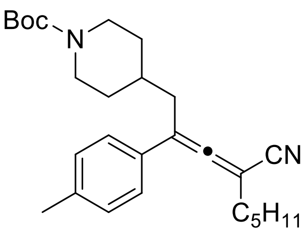

4r

***tert*-butyl 4-(4-cyano-2-(*p*-tolyl)nona-2,3-dien-1-yl)piperidine-1-carboxylate (4r):** Pale yellow oil, 61.8 mg, isolated yield 73%. ^{1}H NMR (400 MHz, $CDCl_3$) δ 7.22 – 7.16 (m, 4H), 4.14 – 4.09 (m, 2H), 2.67 – 2.61 (m, 2H), 2.46 – 2.41 (m, 2H), 2.36 (s, 3H), 2.29 – 2.25 (m, 2H), 1.75 – 1.70 (m, 2H), 1.65 – 1.55 (m, 3H), 1.45 (s, 9H), 1.35 – 1.31 (m, 4H), 1.21 – 1.13 (m, 2H), 0.90 – 0.86 (m, 3H); ^{13}C NMR (100 MHz, $CDCl_3$) δ 212.4, 154.9, 138.6, 130.4, 129.7, 126.7, 115.7, 109.4, 84.3, 79.5, 37.5, 34.7, 32.3, 32.2, 31.9, 31.0, 28.6, 27.6, 22.4, 21.3, 14.0. HRMS (ESI): m/z calcd. for $C_{27}H_{38}N_2O_2Na^+$([M+Na]$^+$) = 445.2825, found = 445.2819.

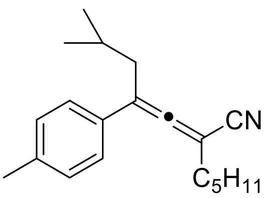

4s

6-methyl-2-pentyl-4-(*p*-tolyl)hepta-2,3-dienenitrile (4s): Colorless oil, 44.6 mg, isolated yield 79%. ^{1}H NMR (500 MHz, $CDCl_3$) δ 7.23 (d, J = 8.5 Hz, 2H), 7.17 (d, J = 7.5 Hz, 2H), 2.43 – 2.34 (m, 5H), 2.29 – 2.26 (m, 2H), 1.85 – 1.79 (m, 1H), 1.62 – 1.56 (m, 2H), 1.36 – 1.30 (m, 4H), 0.99 – 0.97 (m, 6H), 0.89 (t, J = 7.3 Hz, 3H); ^{13}C NMR (125 MHz, $CDCl_3$) δ 212.6, 138.4, 130.8, 129.6, 126.8, 115.9, 110.6, 84.1, 39.8, 32.0, 31.1, 27.6, 27.0, 22.8, 22.7, 22.4, 21.3, 14.1. HRMS (ESI): m/z calcd. for $C_{20}H_{27}NNa^+$([M+Na]$^+$) = 304.2036, found = 304.2040.

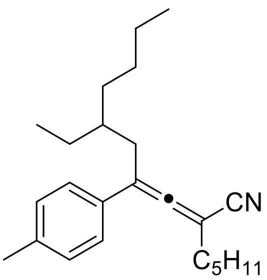

4t

6-ethyl-2-pentyl-4-(*p*-tolyl)deca-2,3-dienenitrile (4t): Colorless oil, 45.0 mg, isolated yield 67%, dr = 1:1. ^{1}H NMR (400 MHz, $CDCl_3$) δ 7.24 – 7.21 (m, 2H), 7.18 – 7.16 (m, 2H), 2.44 – 2.42 (m, 2H), 2.36 (s, 3H), 2.29 – 2.25 (m, 2H), 1.61 – 1.55 (m, 2H), 1.52 – 1.47 (m, 1H), 1.39 – 1.26 (m, 12H), 0.91 – 0.86 (m, 9H); ^{13}C NMR (100 MHz, $CDCl_3$) δ 212.6, 138.4, 131.0, 131.0, 129.6, 126.8, 115.9, 110.8, 110.8, 84.1, 84.0, 37.5, 37.5, 35.0, 35.0, 33.0, 32.8, 32.0, 31.1, 29.0, 28.9, 27.7, 26.1, 25.9, 23.3, 22.4, 21.3, 14.3, 14.1, 10.8, 10.7. HRMS (ESI): m/z calcd. for $C_{24}H_{35}NNa^+$([M+Na]$^+$) = 360.2662, found = 360.2662.

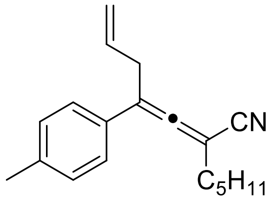

4u

2-pentyl-4-(*p*-tolyl)hepta-2,3,6-trienenitrile (4u): Pale yellow oil, 13.8 mg, isolated yield 26%. ^{1}H NMR (400 MHz, $CDCl_3$) δ 7.25 – 7.23 (m, 2H), 7.18 – 7.16 (m, 2H), 5.92 – 5.82 (m, 1H), 5.21 – 5.13 (m, 2H), 3.27 – 3.25 (m, 2H), 2.36 (s, 3H), 2.29 – 2.26 (m, 2H), 1.58 – 1.54 (m, 2H), 1.34 – 1.30 (m, 4H), 0.89 – 0.86 (m, 3H); ^{13}C NMR (100 MHz, $CDCl_3$) δ 212.5, 138.6, 134.0, 130.4, 129.6, 126.7, 117.8, 115.8, 110.6, 85.5, 34.7, 32.0, 31.0, 27.5, 22.4, 21.3, 14.1. HRMS (ESI): m/z calcd. for $C_{19}H_{24}N^+$($[M+H]^+$) = 266.1903, found = 266.1897.

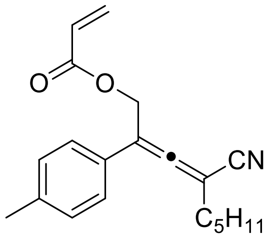

4u'

4-cyano-2-(*p*-tolyl)nona-2,3-dien-1-yl acrylate (4u'): Pale yellow oil, 16.7 mg, isolated yield 27%. ^{1}H NMR (400 MHz, $CDCl_3$) δ 7.24 – 7.18 (m, 4H), 6.45 (dd, *J* = 17.4, 1.4 Hz, 1H), 6.14 (dd, *J* = 17.2, 10.4 Hz, 1H), 5.89 (dd, *J* = 10.4, 1.2 Hz, 1H), 5.12 (q, *J* = 13.1 Hz, 2H), 2.37 (s, 3H), 2.30 (t, *J* = 7.6 Hz, 2H), 1.60 – 1.55 (m, 2H), 1.35 – 1.28 (m, 4H), 0.87 (t, *J* = 7.2 Hz, 3H); ^{13}C NMR (100 MHz, $CDCl_3$) δ 211.9, 165.5, 139.2, 131.9, 129.9, 128.1, 127.9, 126.7, 114.9, 108.2, 87.3, 61.6, 31.9, 31.0, 27.4, 22.4, 21.4, 14.0. HRMS (ESI): m/z calcd. for $C_{20}H_{23}NO_2Na^+$($[M+Na]^+$) = 332.1621, found = 332.1623.

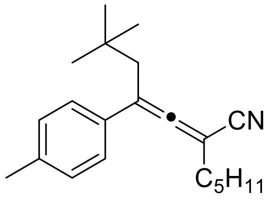

4v

6,6-dimethyl-2-pentyl-4-(*p*-tolyl)hepta-2,3-dienenitrile (4v): Pale yellow oil, 50.7 mg, isolated yield 86%. ^{1}H NMR (400 MHz, $CDCl_3$) δ 7.24 (d, *J* = 8.4 Hz, 2H), 7.15 (d, *J* = 8.0 Hz, 2H), 2.51 – 2.38 (m, 2H), 2.35 (s, 3H), 2.28 – 2.24 (m, 2H), 1.59 – 1.56 (m, 2H), 1.34 – 1.30 (m, 4H), 0.93 (s, 9H), 0.89 – 0.85 (m, 3H); ^{13}C NMR (100 MHz, $CDCl_3$) δ 213.8, 138.2, 132.3, 129.5, 127.0, 116.0, 109.6, 83.0, 43.9, 32.6, 31.9, 31.1, 30.0, 27.5, 22.4, 21.3, 14.1. HRMS (ESI): m/z calcd. for $C_{21}H_{29}NNa^+$($[M+Na]^+$) = 318.2192, found = 318.2198.

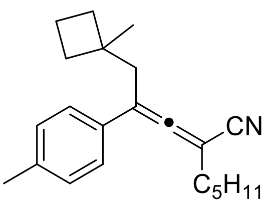

4w

2-(3-(1-methylcyclobutyl)-2-(*p*-tolyl)prop-1-en-1-ylidene)heptanenitrile (4w): Colorless oil, 50.8 mg, isolated yield 83%. ^{1}H NMR (400 MHz, $CDCl_3$) δ 7.23 (d, *J* = 8.4 Hz, 2H), 7.17 – 7.15 (m, 2H), 2.62 (d, *J* = 0.8 Hz, 2H), 2.35 (s, 3H), 2.27 – 2.23 (m, 2H), 1.93 – 1.69 (m, 6H), 1.61 – 1.55 (m, 2H), 1.34 – 1.31 (m, 4H), 1.18 (s, 3H), 0.89 – 0.86 (m, 3H); ^{13}C NMR (100 MHz, $CDCl_3$) δ 213.2, 138.3, 131.9, 129.5, 126.8, 115.9, 109.1, 84.3, 42.8, 38.9, 33.8, 33.8, 32.0, 31.2, 27.5, 26.3, 22.4, 21.3, 15.4, 14.1. HRMS (ESI): m/z calcd. for $C_{22}H_{29}NNa^{+}$($[M+Na]^{+}$) = 330.2192, found = 330.2196.

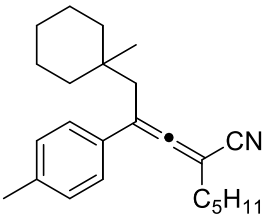

4x

2-（3-（1-methylcyclohexyl）-2-(*p*-tolyl)prop-1-en-1-ylidene）heptanenitrile (4x): Colorless oil, 58.1 mg, isolated yield 87%. ^{1}H NMR (500 MHz, $CDCl_3$) δ 7.26 – 7.23 (m, 2H), 7.15 (d, *J* = 8.0 Hz, 2H), 2.51 – 2.42 (m, 2H), 2.35 (s, 3H), 2.26 – 2.23 (m, 2H), 1.59 – 1.56 (m, 2H), 1.46 – 1.41 (m, 5H), 1.33 – 1.25 (m, 9H), 0.89 – 0.86 (m, 6H); ^{13}C NMR (125 MHz, $CDCl_3$) δ 213.7, 138.1, 132.7, 129.5, 127.0, 116.1, 109.0, 82.7, 42.6, 38.2, 38.1, 35.1, 31.9, 31.1, 27.5, 26.4, 25.3, 22.4, 22.2, 21.3, 14.1. HRMS (ESI): m/z calcd. for $C_{24}H_{33}NNa^{+}$($[M+Na]^{+}$) = 358.2505, found = 358.2504.

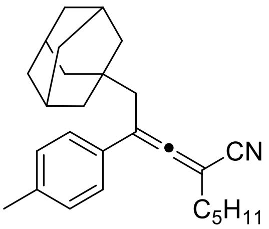

4y

2-(3-(adamantan-1-yl)-2-(*p*-tolyl)prop-1-en-1-ylidene)heptanenitrile (4y): Colorless oil, 58.0 mg, isolated yield 78%. ^{1}H NMR (400 MHz, $CDCl_3$) δ 7.26 – 7.24 (m, 2H), 7.15 (d, *J* = 8.0 Hz, 2H), 2.39 – 2.35 (m, 4H), 2.27 – 2.23 (m, 3H), 1.94 – 1.91 (m, 3H), 1.68 – 1.56 (m, 8H), 1.53 – 1.44 (m, 6H), 1.34 – 1.29 (m, 4H), 0.89 – 0.85 (m, 3H); ^{13}C NMR (100 MHz, $CDCl_3$) δ 213.7, 138.1, 132.4, 129.5, 127.0, 116.2, 108.2, 82.8, 44.6, 43.0, 37.0, 34.6, 32.0, 31.1, 28.8, 27.6, 22.4, 21.3, 14.1. HRMS (ESI): m/z calcd. for $C_{27}H_{35}NNa^{+}$($[M+Na]^{+}$) = 396.2662, found = 396.2666.

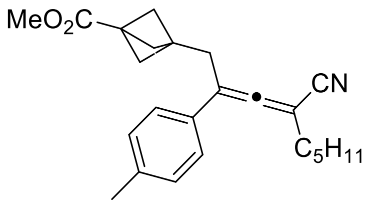

4z

Methyl 3-(4-cyano-2-(*p*-tolyl)nona-2,3-dien-1-yl)bicyclo[1.1.1]pentane-1-carboxylate (4z): Pale yellow oil, 60.2 mg, isolated yield 83%. ^{1}H NMR (400 MHz, $CDCl_3$) δ 7.20 – 7.14 (m, 4H), 3.63 (s, 3H), 2.73 (s, 2H), 2.35 (s, 3H), 2.30 – 2.26 (m, 2H), 1.93 (s, 6H), 1.62 – 1.54 (m, 2H), 1.35 – 1.32 (m, 4H), 0.90 – 0.86 (m, 3H); ^{13}C NMR (100 MHz, $CDCl_3$) δ 212.9, 170.3, 138.6, 130.3, 129.6, 126.7, 115.7, 108.8, 84.6, 52.4, 51.7, 38.9, 38.0, 32.9, 32.1, 31.1, 27.5, 22.4, 21.3, 14.1. HRMS (ESI): m/z calcd. for $C_{24}H_{29}NO_2Na^+$([M+Na]$^+$) = 386.2091, found = 386.2082.

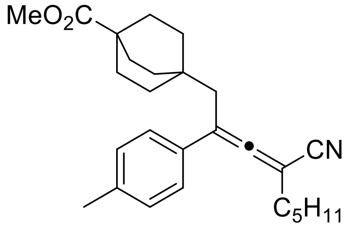

4zz

Methyl 4-(4-cyano-2-(*p*-tolyl)nona-2,3-dien-1-yl)bicyclo[2.2.2]octane-1-carboxylate (4zz): Pale yellow oil, 66.6 mg, isolated yield 82%. ^{1}H NMR (400 MHz, $CDCl_3$) δ 7.21 (d, *J* = 8.0 Hz, 2H), 7.16 – 7.14 (m, 2H), 3.61 (s, 3H), 2.44 – 2.30 (m, 5H), 2.26 – 2.23 (m, 2H), 1.76 – 1.72 (m, 6H), 1.58 – 1.54 (m, 2H), 1.44 – 1.39 (m, 6H), 1.33 – 1.28 (m, 4H), 0.89 – 0.85 (m, 3H); ^{13}C NMR (100 MHz, $CDCl_3$) δ 213.6, 178.3, 138.3, 131.9, 129.6, 127.0, 115.9, 108.6, 83.1, 51.7, 41.5, 38.9, 32.8, 32.0, 31.1, 31.0, 28.6, 27.5, 22.4, 21.3, 14.1. HRMS (ESI): m/z calcd. for $C_{27}H_{35}NO_2Na^+$([M+Na]$^+$) = 428.2560, found = 428.2565.

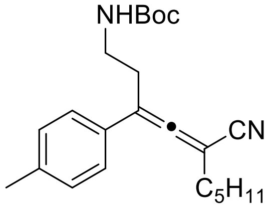

5a

***tert*-butyl(5-cyano-3-(*p*-tolyl)deca-3,4-dien-1-yl)carbamate (5a):** Pale yellow oil, 32.0 mg, isolated yield 43%. ^{1}H NMR (400 MHz, $CDCl_3$) δ 7.23 (d, *J* = 8.0 Hz, 2H), 7.18 (d, *J* = 8.0 Hz, 2H), 4.67 (br, s, 1H), 3.35 – 3.30 (m, 2H), 2.74 – 2.69 (m, 2H), 2.35 (s, 3H), 2.31 – 2.27 (m, 2H), 1.59 – 1.56 (m, 2H), 1.45 (s, 9H), 1.35 – 1.30 (m, 4H), 0.89 – 0.86 (m, 3H); ^{13}C NMR (100 MHz, $CDCl_3$) δ 212.0, 155.9, 138.8, 130.0, 129.8, 126.6, 115.6, 85.6, 39.0, 32.1, 31.1, 28.5, 27.6, 22.4,

21.3, 14.1. HRMS (ESI): m/z calcd. for $C_{23}H_{32}N_2O_2Na^+$([M+Na]$^+$) = 391.2356, found = 391.2355.

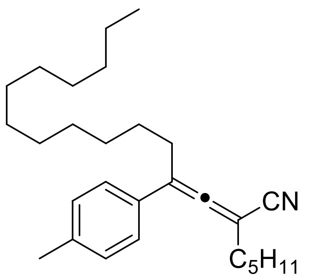

5b

2-pentyl-4-(*p*-tolyl)hexadeca-2,3-dienenitrile (5b): Pale yellow oil, 52.2 mg, isolated yield 66%. ^{1}H NMR (400 MHz, $CDCl_3$) δ 7.23 (d, *J* = 8.0 Hz, 2H), 7.17 (d, *J* = 8.0 Hz, 2H), 2.49 (t, *J* = 7.4 Hz, 2H), 2.36 (s, 3H), 2.28 (t, *J* = 7.6 Hz, 2H), 1.60 – 1.49 (m, 4H), 1.39 – 1.26 (m, 22H), 0.90 – 0.87 (m, 6H).; ^{13}C NMR (100 MHz, $CDCl_3$) δ 212.3, 138.4, 130.9, 129.6, 126.7, 116.0, 112.1, 84.9, 32.1, 32.0, 31.1, 30.3, 29.8, 29.8, 29.8, 29.6, 29.5, 29.5, 27.8, 27.6, 22.8, 22.5, 21.3, 14.3, 14.1. HRMS (ESI): m/z calcd. for $C_{28}H_{43}NNa^+$([M+Na]$^+$) = 416.3288, found = 416.3290.

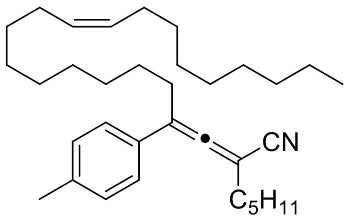

5c

(*Z*)-2-pentyl-4-(*p*-tolyl)docosa-2,3,13-trienenitrile (5c): Pale yellow oil, 66.4 mg, isolated yield 70%. ^{1}H NMR (400 MHz, $CDCl_3$) δ 7.23 (d, *J* = 8.0 Hz, 2H), 7.17 (d, *J* = 8.0 Hz, 2H), 5.36 – 5.34 (m, 2H), 2.49 (t, *J* = 7.4 Hz, 2H), 2.36 (s, 3H), 2.28 (t, *J* = 7.6 Hz, 2H), 2.04 – 1.99 (m, 4H), 1.60 – 1.50 (m, 4H), 1.35 – 1.26 (m, 25H), 0.90 – 0.87 (m, 6H); ^{13}C NMR (100 MHz, $CDCl_3$) δ 212.2, 138.4, 130.9, 130.1, 130.0, 129.6, 126.7, 116.0, 112.0, 84.9, 32.1, 32.0, 31.1, 30.3, 29.9, 29.9, 29.7, 29.7, 29.6, 29.5, 29.4, 27.8, 27.6, 27.4, 22.8, 22.5, 21.3, 14.3, 14.1. HRMS (ESI): m/z calcd. for $C_{34}H_{53}NNa^+$([M+Na]$^+$) = 498.4070, found = 498.4082.

(*E*)-10-(4-hydroxy-6-methoxy-7-methyl-3-oxo-1,3-dihydroisobenzofuran-5-yl)-8-

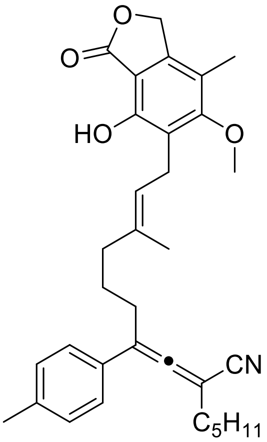

5d

methyl-2-pentyl-4-(*p*-tolyl)deca-2,3,8-trienenitrile (5d): Pale yellow oil, 50.6 mg, isolated yield 49%. ^{1}H NMR (400 MHz, $CDCl_3$) δ 7.68 (s, 1H), 7.19 – 7.13 (m, 4H), 5.25 – 5.19 (m, 3H), 3.77 (s, 3H), 3.40 (d, *J* = 6.8 Hz, 2H), 2.44 – 2.41 (m, 2H), 2.35 (s, 3H), 2.27 – 2.23 (m, 2H), 2.14 (s, 3H), 2.08 (t, *J* = 7.4 Hz, 2H), 1.78 (d, *J* = 1.2 Hz, 3H), 1.64 – 1.53 (m, 4H), 1.34 – 1.30 (m, 4H), 0.88 – 0.85 (m, 3H); ^{13}C NMR (100 MHz, $CDCl_3$) δ 212.2, 173.1, 163.8, 153.8, 144.1, 138.4, 135.1, 130.8, 129.6, 126.6, 122.8, 122.5, 116.9, 116.0, 111.9, 106.5, 85.0, 70.2, 61.1, 39.2, 32.0, 31.1, 29.6, 27.6, 25.8, 22.8, 22.4, 21.3, 16.2, 14.1, 11.7. HRMS (ESI): m/z calcd. for $C_{33}H_{39}NO_4Na^+$([M+Na]$^+$) = 536.2771, found = 536.2770.

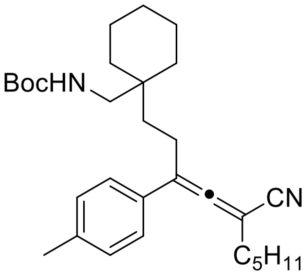

5e

***tert*-butyl((1-(5-cyano-3-(*p*-tolyl)deca-3,4-dien-1-yl)cyclohexyl)methyl)carbamate (5e):** Pale yellow oil, 56.8 mg, isolated yield 61%. ^{1}H NMR (500 MHz, $CDCl_3$) δ 7.23 (d, *J* = 8.0 Hz, 2H), 7.16(d, *J* = 8.0 Hz, 2H), 4.47 (br, s, 1H), 3.12 – 3.10 (m, 2H), 2.46 (t, *J* = 8.3 Hz, 2H), 2.35 (s, 3H), 2.28 (t, *J* = 7.8 Hz, 2H), 1.59 – 1.54 (m, 2H), 1.49 – 1.41 (m, 17H), 1.34 – 1.31 (m, 8H), 0.88 (t, *J* = 7.0 Hz, 3H); ^{13}C NMR (125 MHz, $CDCl_3$) δ 212.1, 156.3, 138.5, 130.8, 129.6, 126.7, 116.0, 112.8, 85.0, 79.4, 46.7, 36.4, 33.7, 33.6, 33.6, 32.1, 31.1, 28.5, 27.6, 26.3, 24.1, 22.5, 21.6, 21.3, 14.1. HRMS (ESI): m/z calcd. for $C_{30}H_{44}N_2O_2Na^+$([M+Na]$^+$) = 487.3295, found = 487.3286.

8-(4-(bis(2-chloroethyl)amino)phenyl)-2-pentyl-4-(*p*-tolyl)octa-2,3-dienenitrile

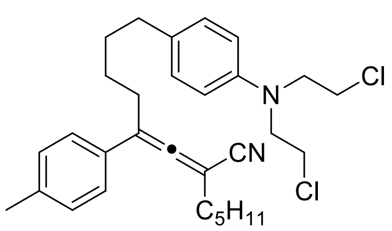

5f

(5f): Pale yellow oil, 56.0 mg, isolated yield 56%. ^{1}H NMR (400 MHz, $CDCl_3$) δ 7.22 (d, *J* = 8.4 Hz, 2H), 7.17 (d, *J* = 8.0 Hz, 2H), 7.07 (d, *J* = 8.4 Hz, 2H), 6.63 (d, *J* = 8.8 Hz, 2H), 3.72 – 3.68 (m, 4H), 3.64 – 3.60 (m, 4H), 2.58 – 2.50 (m, 4H), 2.36 (s, 3H), 2.28 – 2.24 (m, 2H), 1.71 – 1.65 (m, 2H), 1.62 – 1.53 (m, 4H), 1.35 – 1.31 (m, 4H), 0.88 (t, *J* = 7.0 Hz, 3H); ^{13}C NMR (100 MHz, $CDCl_3$) δ 212.2, 144.3, 138.4, 131.6, 130.8, 129.7, 129.6, 126.7, 116.0, 112.3, 111.9, 85.1, 53.8, 40.7, 34.6, 32.0, 31.4, 31.1, 30.1, 27.6, 27.3, 22.4, 21.3, 14.1. HRMS (ESI): m/z calcd. for $C_{30}H_{38}N_2Cl_2Na^+$($[M+Na]^+$) = 519.2304, found = 519.2306.

5. Scale-up syntheses and further transformations

5.1 Scale-up reaction

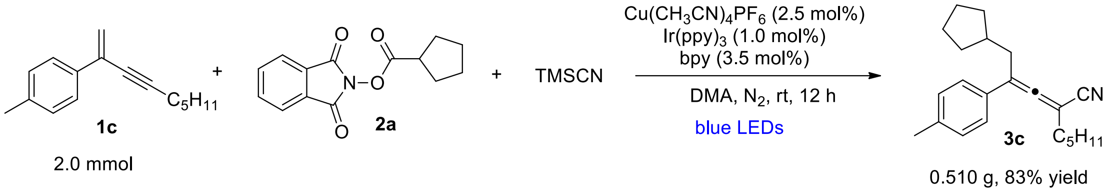

In a 100 mL sealed tube, $Cu(CH_3CN)_4PF_6$ (0.05 mmol, 2.5 mol%), bpy (0.07 mmol, 3.5 mol%), $Ir(ppy)_3$ (0.02 mmol, 1.0 mol%) and NHP ester **2a** (2.0 mmol, 1.0 equiv) were added in degassed DMA (10 mL) under a nitrogen atmosphere, and the mixture was stirred at room temperature for 30 minutes. Then 1,3-enyne **1c** (2.0 mmol, 1.0 equiv) and TMSCN (4.0 mmol, 2.0 equiv) were sequentially added. The reaction mixture was stirred at room temperature under 30 W blue LEDs irradiation for 12 hours. After the reaction completion, the reaction mixture was diluted with EtOAc, then washed with brine, dried over Na_2SO_4, filtered, and concentrated under reduced pressure. The residue was purified by column chromatography on silica gel (hexane/EtOAc) to afford the product **3c** (0.510 g, 83% yield).

5.2 Further transformations

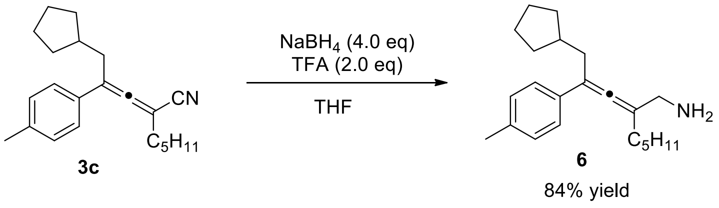

TFA (0.4 mmol, 30 μL) was added dropwise to a suspension of $NaBH_4$ (0.8 mmol, 30.4 mg) and **3c** (0.2 mmol, 61.4 mg) in dry THF (4 mL) at 0 °C. The reaction mixture was stirred at room temperature overnight. Then the mixture was cautiously added water at 0 °C and extracted with DCM. The combined organic layer was dried over Na_2SO_4, and concentrated under reduced pressure and then was purified by flash chromatography on silica gel (hexane/EtOAc) to afford the product **6** in 84% yield.

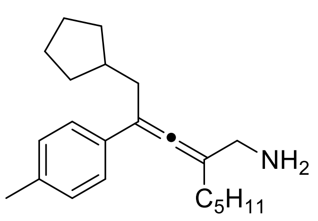

6

2-(3-cyclopentyl-2-(*p*-tolyl)prop-1-en-1-ylidene)heptan-1-amine (6): Colorless oil, 52.2 mg, isolated yield 84%. ^{1}H NMR (400 MHz, $CDCl_3$) δ 7.22 (d, *J* = 8.0 Hz, 2H), 7.14 (d, *J* = 8.0 Hz, 2H), 3.57 – 3.50 (br, m, 2H), 3.39 – 3.35 (m, 2H), 2.54 – 2.38 (m, 2H), 2.34 (s, 3H), 2.12 – 2.07 (m, 2H), 2.04 – 1.96 (m, 1H), 1.76 – 1.72 (m, 2H), 1.65 – 1.59 (m, 2H), 1.55 – 1.47 (m, 4H), 1.36 – 1.26 (m, 4H), 1.23 – 1.15 (m, 2H), 0.89 – 0.86 (m, 3H); ^{13}C NMR (100 MHz, $CDCl_3$) δ 198.1, 137.5, 133.4, 129.5, 126.1, 112.1, 105.5, 49.7, 38.4, 37.7, 33.2, 33.0, 31.8, 31.7, 27.5, 25.5, 25.4, 22.6, 21.2, 14.1. HRMS (ESI): m/z calcd. for $C_{22}H_{34}N^+$($[M+H]^+$) = 312.2686, found = 312.2689.

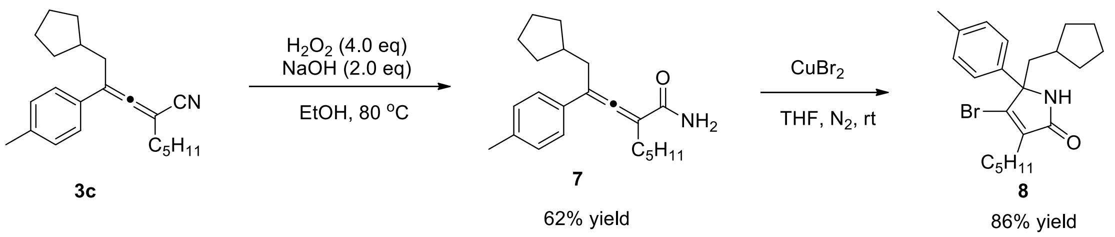

7[3a]: H_2O_2 (0.8 mmol, 30% aqueous solution) was added dropwise to a suspension of NaOH (0.4 mmol, 92 mg) and **3c** (0.2 mmol, 61.4 mg) in dry EtOH (2 mL) at room temperature. Then the reaction mixture was stirred at 80 °C for 4 h. After cooling to room temperature, the mixture was quenched with aqueous solution of sodium thiosulfate and extracted with ethyl acetate. The combined organic layer was dried over Na_2SO_4, and concentrated under reduced pressure and then was purified by flash chromatography on silica gel (hexane/EtOAc) to afford 40.3 mg product **7** in 62% yield.

8[3b]: Under nitrogen atmosphere, a mixture of **7** (0.12 mmol, 40.3 mg) and $CuBr_2$ (0.48 mmol, 107 mg) in dry THF (1 mL) was stirred at room temperature overnight. After removal of THF, an aqueous solution of NaOH (1 M, 2 mL) was added. Copper salts were removed by filtration through a Celite pad. The filtrate was extracted with ethyl acetate. The combined organic layer was dried over Na_2SO_4, and concentrated under reduced pressure and then was purified by flash chromatography on silica gel

(hexane/EtOAc) to afford 41.8 mg product **8** in 86% yield

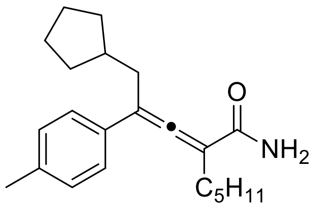

7

2-(3-cyclopentyl-2-(*p*-tolyl)prop-1-en-1-ylidene)heptanamide (7): Pale yellow solid, 40.3 mg, isolated yield 62%. ^{1}H NMR (400 MHz, $CDCl_3$) δ 7.28 (d, *J* = 8.4 Hz, 2H), 7.16 (d, *J* = 8.0 Hz, 2H), 5.88 (br, s, 1H), 5.38 (br, s, 1H), 2.54 (dd, *J* = 7.6, 1.6 Hz, 2H), 2.41 – 2.33 (m, 5H), 2.12 – 2.05 (m, 1H), 1.83 – 1.80 (m, 2H), 1.65 – 1.59 (m, 2H), 1.57 – 1.46 (m, 4H), 1.34 – 1.18 (m, 6H), 0.85 (t, *J* = 7.0 Hz, 3H); ^{13}C NMR (100 MHz, $CDCl_3$) δ 207.2, 168.8, 137.7, 132.0, 129.6, 126.2, 110.9, 105.3, 38.3, 37.3, 33.3, 33.1, 31.8, 28.4, 28.2, 25.5, 25.5, 22.6, 21.3, 14.2. HRMS (ESI): m/z calcd. for $C_{22}H_{32}NO^+$($[M+H]^+$) = 326.2478, found = 326.2475.

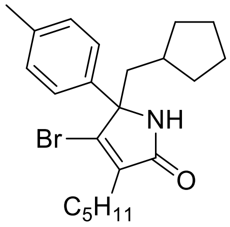

8

4-bromo-5-(cyclopentylmethyl)-3-pentyl-5-(*p*-tolyl)-1*H*-pyrrol-2(5*H*)-one (8): Pale yellow oil, 41.8 mg, isolated yield 86%. ^{1}H NMR (400 MHz, $CDCl_3$) δ 7.29 (d, *J* = 8.4 Hz, 2H), 7.17 – 7.15 (m, 2H), 2.39 – 2.35 (m, 2H), 2.34 (s, 3H), 2.29 – 2.27 (m, 2H), 1.80 – 1.68 (m, 3H), 1.62 – 1.54 (m, 4H), 1.48 – 1.42 (m, 2H), 1.36 – 1.28 (m, 4H), 1.22 – 1.13 (m, 2H), 0.88 (t, *J* = 7.0 Hz, 3H); ^{13}C NMR (100 MHz, $CDCl_3$) δ169.6, 141.9, 138.3, 136.6, 133.4, 129.3, 125.7, 92.7, 42.1, 35.8, 34.2, 34.1, 31.8, 27.1, 25.8, 25.3, 25.1, 22.6, 21.3, 14.2. HRMS (ESI): m/z calcd. for $C_{22}H_{31}BrNO^+$($[M+H]^+$) = 404.1584, found = 404.1583.

6. Control experiment

6.1 Radical trapping experiment

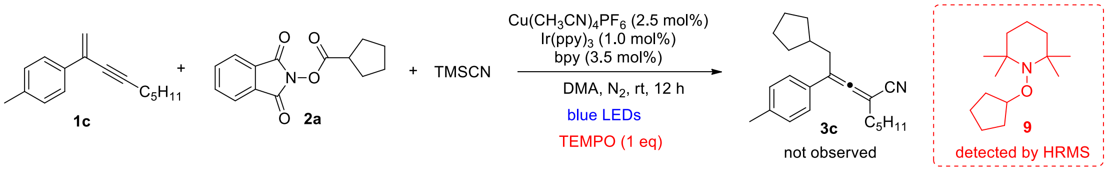

In a 10 mL sealed tube, $Cu(CH_3CN)_4PF_6$ (0.005 mmol, 2.5 mol%), bpy (0.007 mmol, 3.5 mol%), $Ir(ppy)_3$ (0.002 mmol, 1.0 mol%) and NHP ester **2a** (0.2 mmol, 1.0 equiv) were added in degassed DMA under a nitrogen atmosphere, and the mixture was stirred at room temperature for 30 minutes. Then TEMPO (0.2 mmol, 1.0 equiv), 1,3-enyne **1c** (0.2 mmol, 1.0 equiv) and TMSCN (0.4 mmol, 2.0 equiv) were sequentially added. The reaction mixture was stirred at room temperature under 30 W blue LEDs irradiation for 12 hours. The decarboxylative 1,4-carbocyanation was completely inhibited, and the TEMPO-captured **9** was detected by HRMS analysis. HRMS (ESI): m/z calcd. for $C_{14}H_{28}NO^+$([M+H]$^+$) = 226.2165, found = 226.2169.

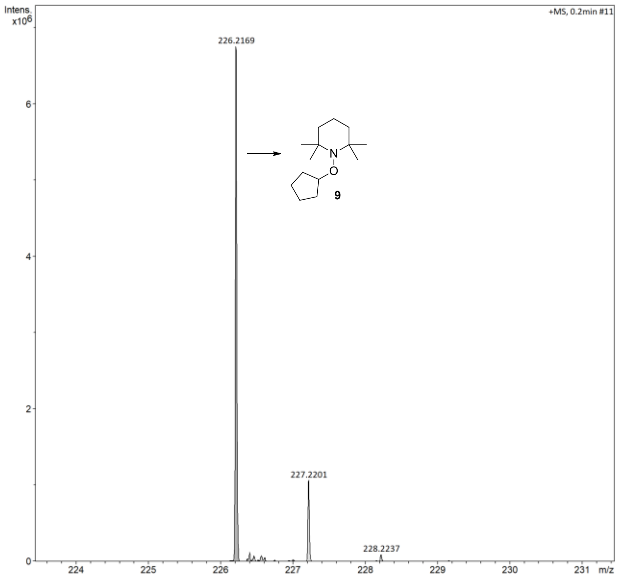

Fig. S1 HRMS of TEMPO-captured **9**

6.2 Ring-opening experiment

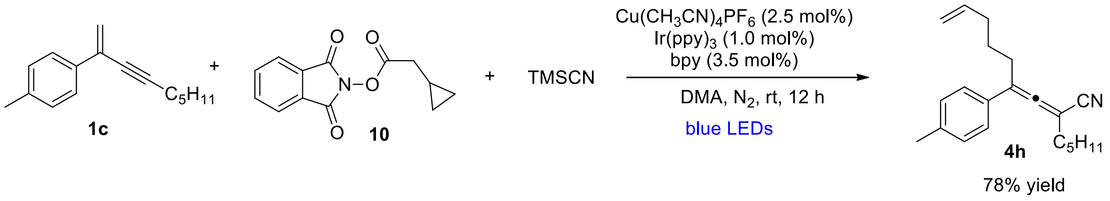

In a 10 mL sealed tube, $Cu(CH_3CN)_4PF_6$ (0.005 mmol, 2.5 mol%), bpy (0.007 mmol, 3.5 mol%), $Ir(ppy)_3$ (0.002 mmol, 1.0 mol%) and NHP ester **10** (0.2 mmol, 1.0 equiv) were added in degassed DMA under a nitrogen atmosphere, and the mixture was stirred at room temperature for 30 minutes. Then 1,3-enyne **1c** (0.2 mmol, 1.0 equiv) and TMSCN (0.4 mmol, 2.0 equiv) were sequentially added. The reaction mixture was stirred at room temperature under 30 W blue LEDs irradiation for 12 hours. After the reaction completion, the reaction mixture was diluted with EtOAc, then washed with brine, dried over Na_2SO_4, filtered, and concentrated under reduced pressure. The residue was purified by column chromatography on silica gel (hexane/EtOAc) to afford the product **4h** (45.7 mg, 78% yield).

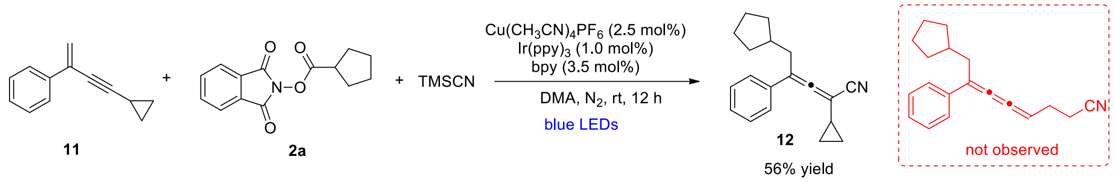

In a 10 mL sealed tube, $Cu(CH_3CN)_4PF_6$ (0.005 mmol, 2.5 mol%), bpy (0.007 mmol, 3.5 mol%), $Ir(ppy)_3$ (0.002 mmol, 1.0 mol%) and NHP ester **2a** (0.2 mmol, 1.0 equiv) were added in degassed DMA under a nitrogen atmosphere, and the mixture was stirred at room temperature for 30 minutes. Then 1,3-enyne **11** (0.2 mmol, 1.0 equiv) and TMSCN (0.4 mmol, 2.0 equiv) were sequentially added. The reaction mixture was stirred at room temperature under 30 W blue LEDs irradiation for 12 hours. After the reaction completion, the reaction mixture was diluted with EtOAc, then washed with brine, dried over Na_2SO_4, filtered, and concentrated under reduced pressure. The residue was purified by column chromatography on silica gel (hexane/EtOAc) to afford the product **12** in 56% yield.

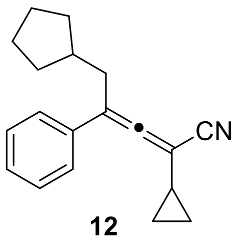

12

5-cyclopentyl-2-cyclopropyl-4-phenylpenta-2,3-dienenitrile (12): Colorless oil, 29.6 mg, isolated yield 56%. ^{1}H NMR (400 MHz, $CDCl_3$) δ 7.39 – 7.28 (m, 5H), 2.53 (d, J = 7.2 Hz, 2H), 2.06 – 2.00 (m, 1H), 1.88 – 1.80 (m, 2H), 1.66 – 1.60 (m, 2H), 1.59 – 1.51 (m, 3H), 1.27 – 1.18 (m, 2H), 0.89 – 0.84 (m, 2H), 0.71 – 0.67 (m, 2H); ^{13}C NMR (100 MHz, $CDCl_3$) δ 212.2, 133.9, 128.9, 128.5, 126.8, 114.7, 113.2, 88.2, 38.1, 37.3, 32.9, 32.9, 25.5, 25.4, 11.8, 6.5, 6.4. HRMS (ESI): m/z calcd. for $C_{19}H_{21}NNa^+$($[M+Na]^+$) = 286.1566, found = 286.1565.

6.3 Ring-closing experiment

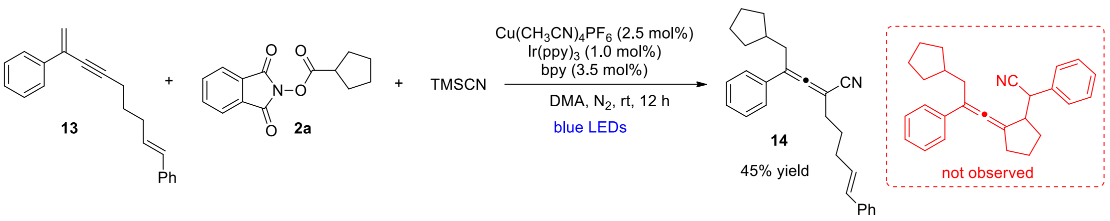

In a 10 mL sealed tube, $Cu(CH_3CN)_4PF_6$ (0.005 mmol, 2.5 mol%), bpy (0.007 mmol, 3.5 mol%), $Ir(ppy)_3$ (0.002 mmol, 1.0 mol%) and NHP ester **2a** (0.2 mmol, 1.0 equiv) were added in degassed DMA under a nitrogen atmosphere, and the mixture was stirred at room temperature for 30 minutes. Then 1,3-enyne **13** (0.2 mmol, 1.0 equiv) and TMSCN (0.4 mmol, 2.0 equiv) were sequentially added. The reaction mixture was stirred at room temperature under 30 W blue LEDs irradiation for 12 hours. After the reaction completion, the reaction mixture was diluted with EtOAc, then washed with brine, dried over Na_2SO_4, filtered, and concentrated under reduced pressure. The residue was purified by column chromatography on silica gel (hexane/EtOAc) to afford the product **14** in 45% yield.

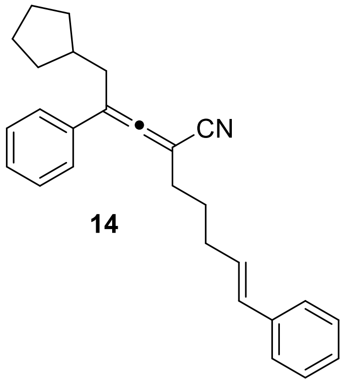

14

(*E*)-2-(3-cyclopentyl-2-phenylprop-1-en-1-ylidene)-7-phenylhept-6-enenitrile (14): Pale yellow oil, 33.1 mg, isolated yield 45%. ^{1}H NMR (400 MHz, $CDCl_3$) δ 7.40 – 7.34 (m, 4H), 7.33 – 7.27 (m, 5H), 7.22 – 7.18 (m, 1H), 6.36 – 6.32 (m, 1H), 6.19 – 6.12 (m, 1H), 2.53 (d, J = 7.6 Hz, 2H), 2.37 – 2.26 (m, 4H), 2.09 – 2.01 (m, 1H), 1.86 – 1.75 (m, 4H), 1.66 – 1.60 (m, 2H), 1.55 – 1.51 (m, 2H), 1.29 – 1.20 (m, 2H); ^{13}C NMR (100 MHz, $CDCl_3$) δ 212.9, 137.6, 133.9, 131.1, 129.4, 128.9, 128.6, 128.5, 127.2, 126.9, 126.1, 115.8, 111.9, 84.4, 38.2, 37.2, 33.0, 32.9, 32.2, 31.4, 27.6, 25.4, 25.4. HRMS (ESI): m/z calcd. for $C_{27}H_{29}NNa^+$ ($[M+Na]^+$) = 390.2192, found = 390.2199.

7. X-ray crystallography of 3r

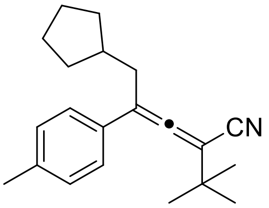

3r

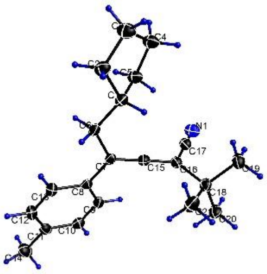

CCDC 2034197 (**3r**) contains the supplementary crystallographic data for this paper. These data can be obtained free of charge from The Cambridge Crystallographic Date Centre via www.ccdc.cam.ac.uk/data_request/cif.

8. References

[1] a) J. Cornella, J. T. Edwards, T. Qin, S. Kawamura, J. Wang, C.-M. Pan, R. Gianatassio, M. Schmidt, M. D. Eastgate, P. S. Baran, *J. Am. Chem. Soc.* **2016**, *138*, 2174. b) T. Qin, J. Cornella, C. Li, L. R. Malins, J. T. Edwards, S. Kawamura, B. D. Maxwell, M. D. Eastgate, P. S. Baran, *Science*. **2016**, *352*, 801.

[2] a) W.-F. Zheng, W. Zhang, C. Huang, P. Wu, H. Qian, L. Wang, Y.-L. Guo, S. Ma, *Nat. Catal*. **2019**, *2*, 997; b) M. T. Muhammad, Y. Jiao, C. Ye, M-F. Chiou, M. Israr, X. Zhu, Y. Li, Z. Wen, A. Studer, H. Bao, *Nat. Commun*. **2020**, *11*, 416; c) C. Ye, Y. Li, X. Zhu, S. Hu, D. Yuan, H. Bao, *Chem. Sci*. **2019**, *10*, 3632; d) X. Zhu, W. Deng, M-F. Chiou, C. Ye, W. Jian, Y. Zeng, Y. Jiao, L. Ge, Y. Li, X. Zhang, H. Bao, *J. Am. Chem. Soc*. **2019**, *141*, 548; e) Y. Liao, X. Yin, X. Wang, W. Yu, D. Fang. L. Hu, M. Wang, J. Liao, *Angew. Chem. Int. Ed.* **2020**, *132*, 1192; f) R. R. Singidi, T. V. RajanBabu, *Org. Lett.* **2010**, *12*, 1192.

[3] a) P. M. Greaves, P. D. Landor, S. R. Landor, O. Odyek, *Tetrahedron* **1974**, *30*, 1427; b) Y.-F. Ao, D.-X. Wang, L. Zhao, M.-X. Wang, *J. Org. Chem.* **2014**, *79*, 3103.

9. Copy of NMR spectra

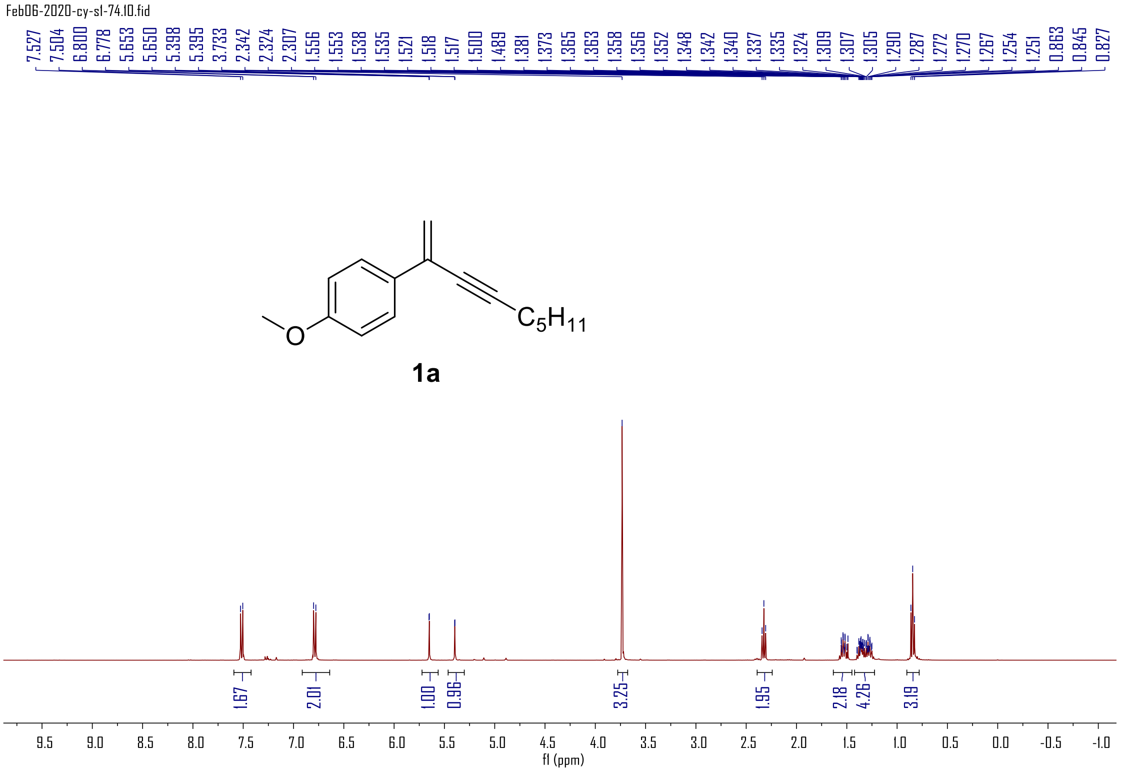

1a

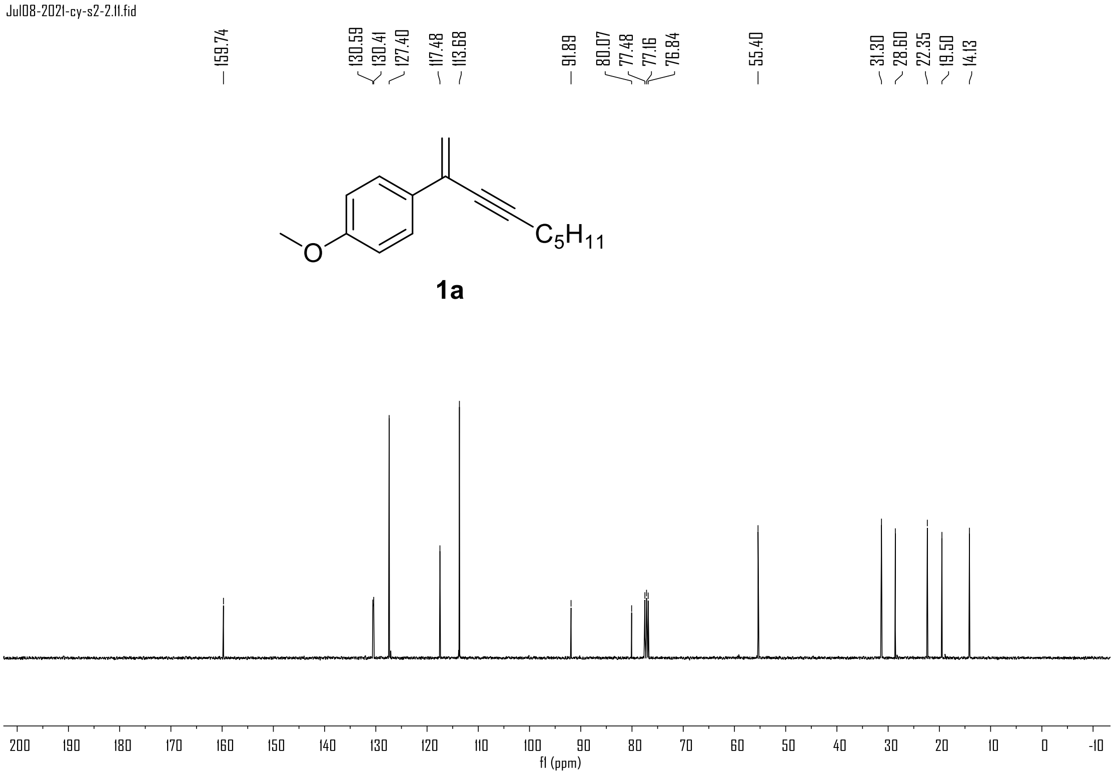

1a

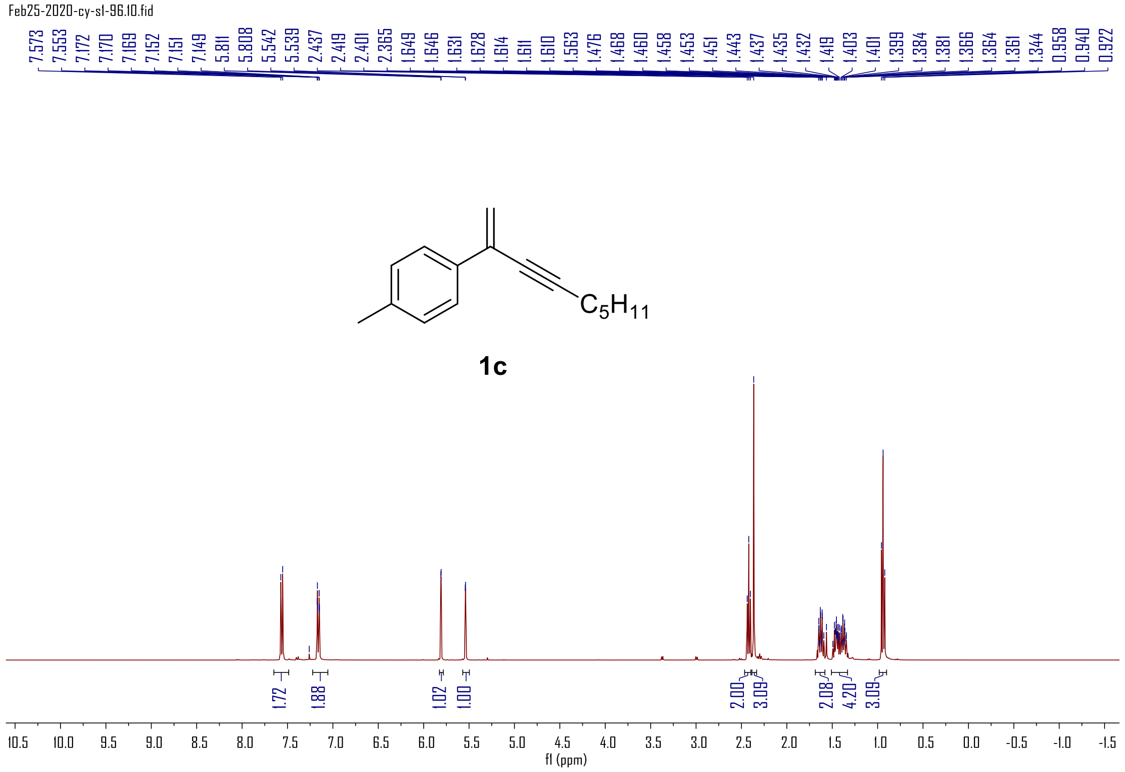
Feb25-2020-cy-sl-96.10.fid
7.573 7.553 7.172 7.170 7.169 7.152 7.151 7.149 5.811 5.808 5.542 5.539 2.437 2.419 2.401 2.365 1.649 1.646 1.631 1.628 1.614 1.611 1.610 1.563 1.476 1.468 1.460 1.458 1.453 1.451 1.443 1.437 1.435 1.432 1.419 1.403 1.401 1.399 1.384 1.381 1.366 1.364 1.361 1.344 0.958 0.940 0.922
C5H11
1c
1.72 1.88 1.02 1.00 2.00 3.09 2.08 4.20 3.09
10.5 10.0 9.5 9.0 8.5 8.0 7.5 7.0 6.5 6.0 5.5 5.0 4.5 4.0 3.5 3.0 2.5 2.0 1.5 1.0 0.5 0.0 -0.5 -1.0 -1.5
f1 (ppm)

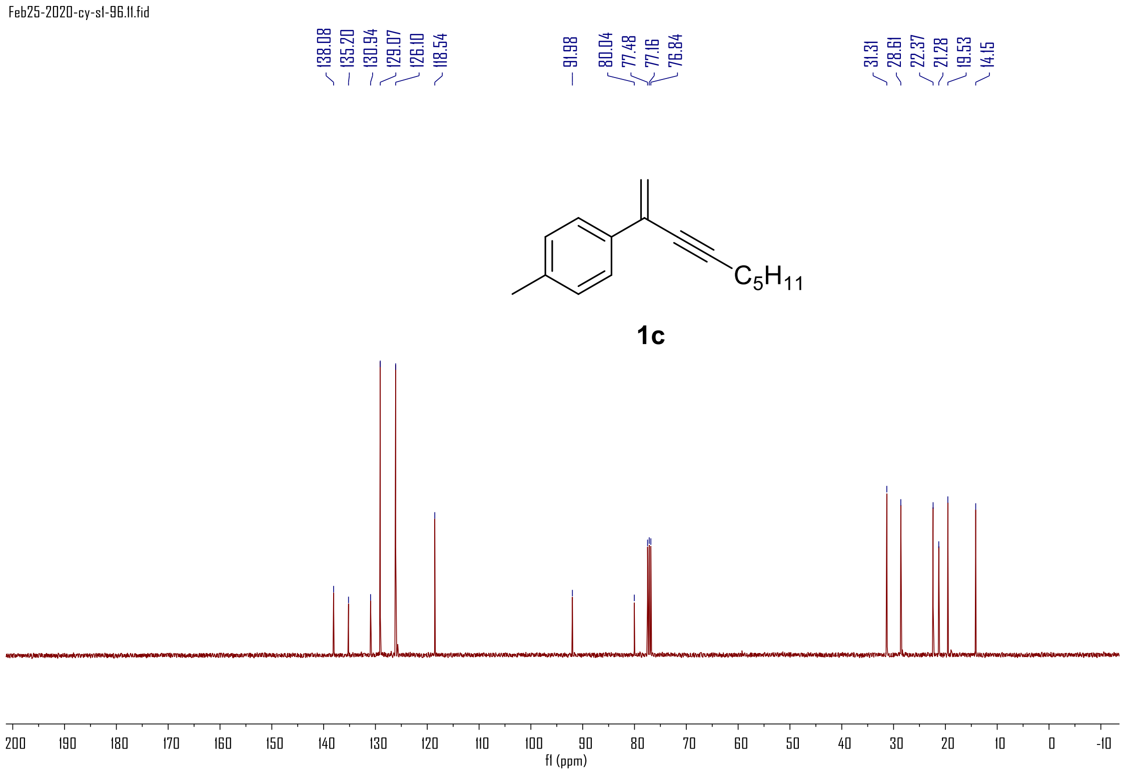
Feb25-2020-cy-sl-96.11.fid
138.08 135.20 130.94 129.07 126.10 118.54 91.98 80.04 77.48 77.16 76.84 31.31 28.61 22.37 21.28 19.53 14.15
C5H11
1c
200 190 180 170 160 150 140 130 120 110 100 90 80 70 60 50 40 30 20 10 0 -10
f1 (ppm)

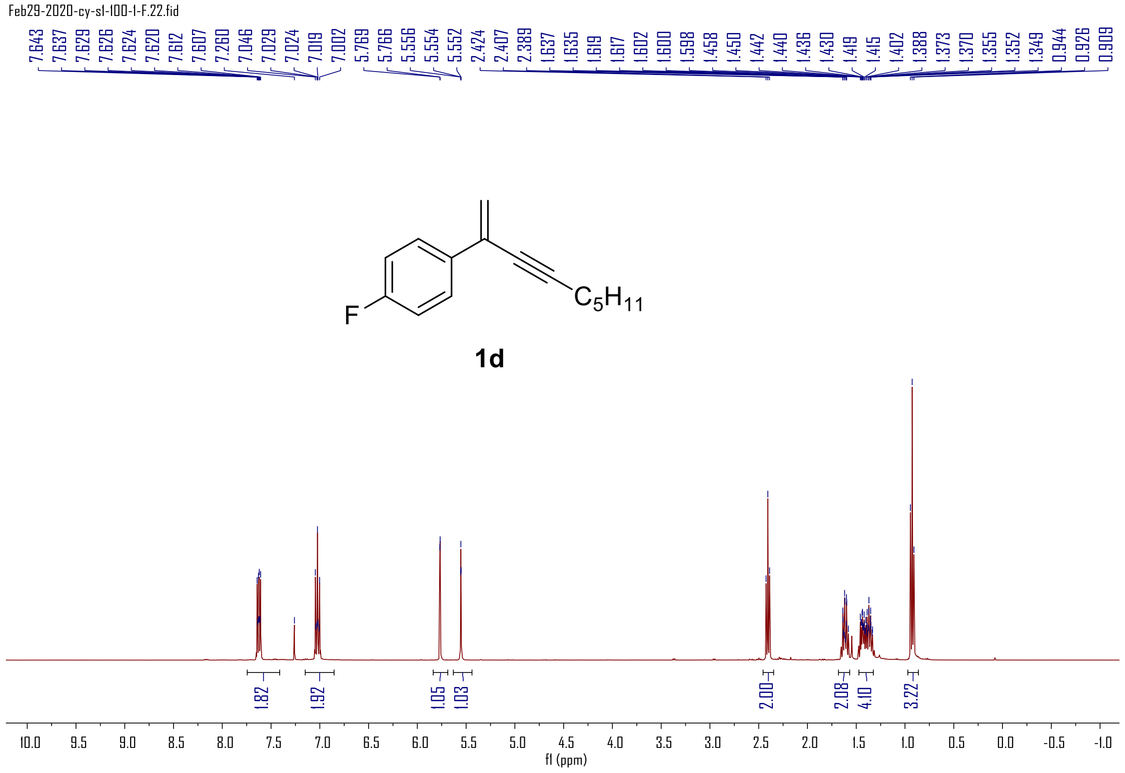
Feb29-2020-cy-sl-100-1-F.22.fid
7.643 7.637 7.629 7.626 7.624 7.620 7.612 7.607 7.260 7.046 7.029 7.024 7.019 7.002 5.769 5.766 5.556 5.554 5.552 2.424 2.407 2.389 1.637 1.635 1.619 1.617 1.602 1.600 1.598 1.458 1.450 1.442 1.440 1.436 1.430 1.419 1.415 1.402 1.388 1.373 1.370 1.355 1.352 1.349 0.944 0.926 0.909
F
C5H11
1d
1.82 1.92 1.05 1.03 2.00 2.08 4.10 3.22
f1 (ppm)

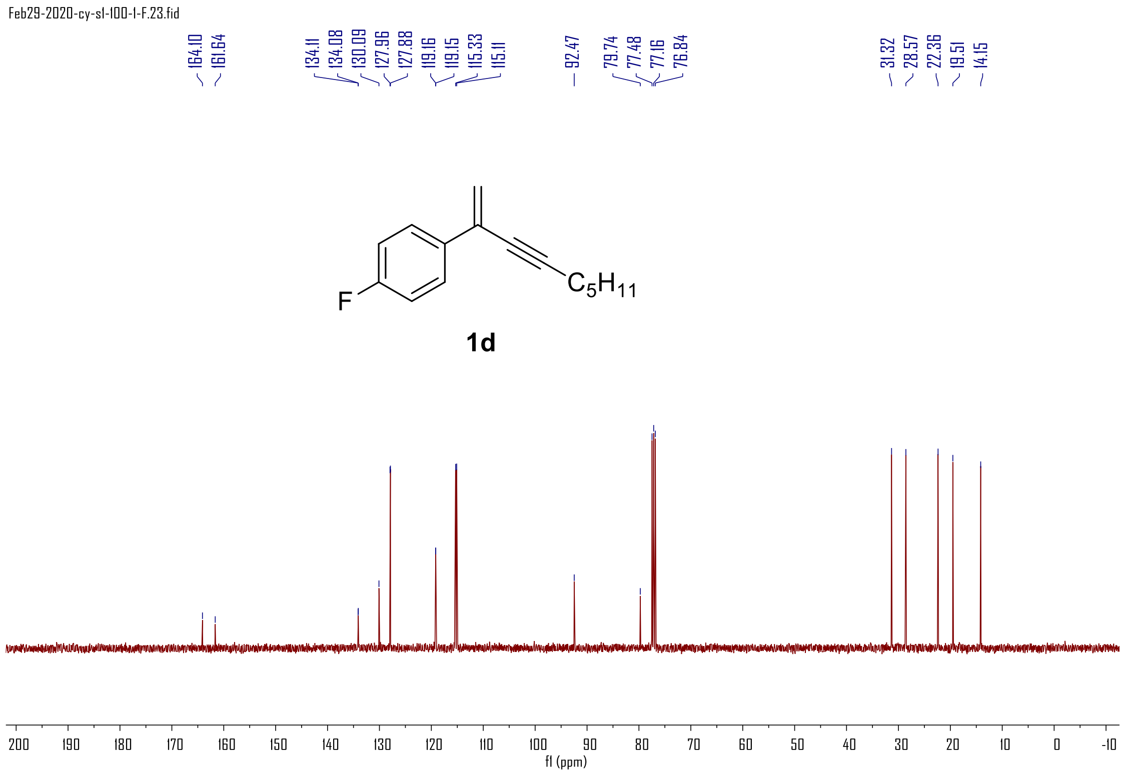
Feb29-2020-cy-sl-100-1-F.23.fid
164.10 161.64 134.11 134.08 130.09 127.96 127.88 119.16 119.15 115.33 115.11 92.47 79.74 77.48 77.16 76.84 31.32 28.57 22.36 19.51 14.15
F
C5H11
1d
f1 (ppm)

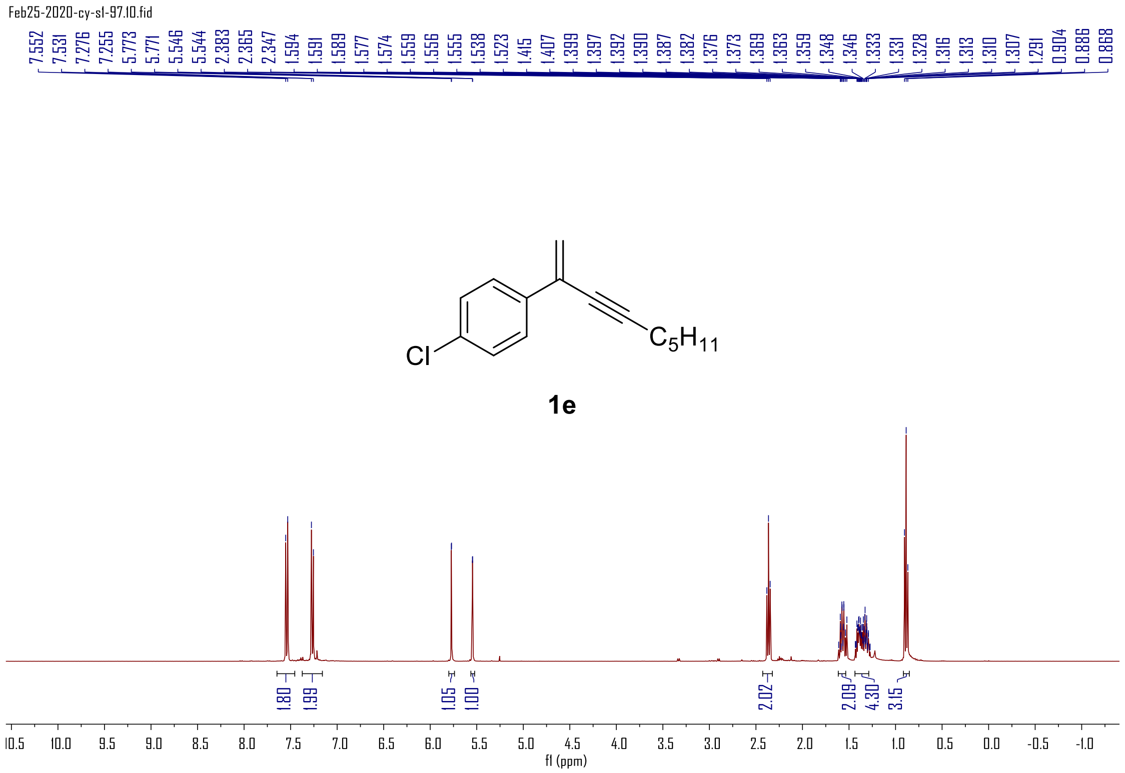
Feb25-2020-cy-sl-97.10.fid
7.552 7.531 7.276 7.255 5.773 5.771 5.546 5.544 2.383 2.365 2.347 1.594 1.591 1.589 1.577 1.574 1.559 1.556 1.555 1.538 1.523 1.415 1.407 1.399 1.397 1.392 1.390 1.387 1.382 1.376 1.373 1.369 1.363 1.359 1.348 1.346 1.333 1.331 1.328 1.316 1.313 1.310 1.307 1.291 0.904 0.886 0.868
Cl
C5H11
1e
1.80
1.99
1.05
1.00
2.02
2.09
4.30
3.15
fl (ppm)

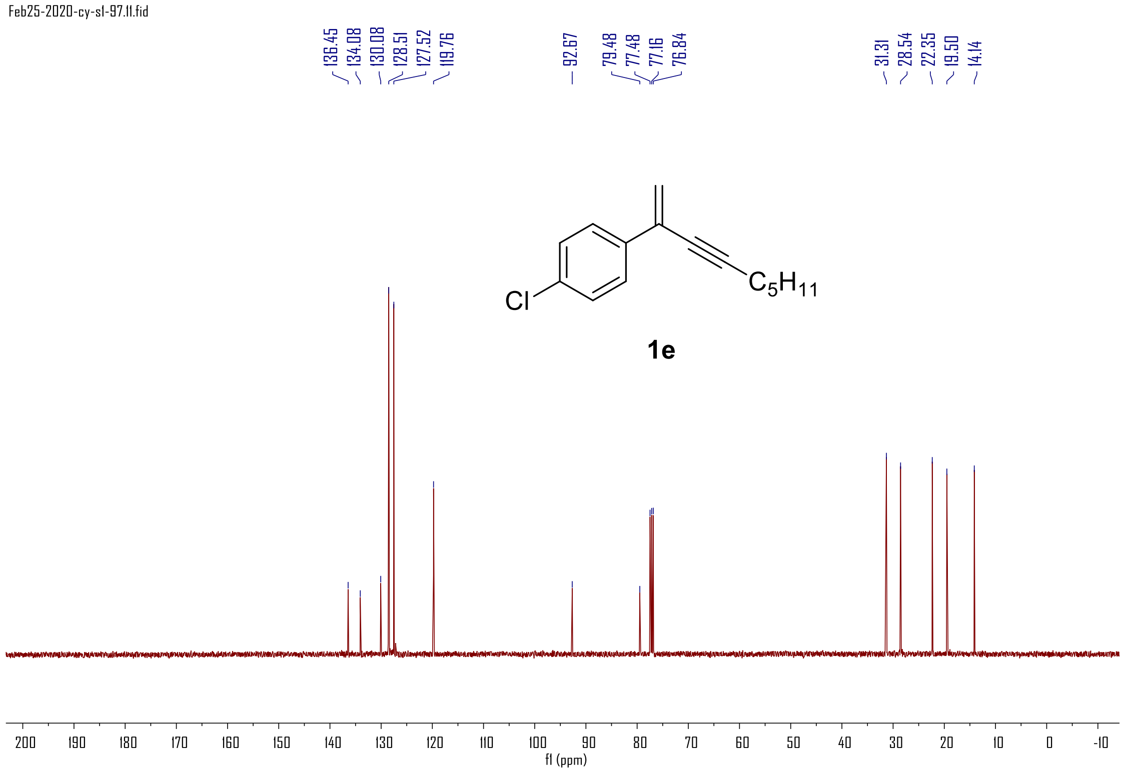
Feb25-2020-cy-sl-97.11.fid
136.45 134.08 130.08 128.51 127.52 119.76
92.67
79.48 77.48 77.16 76.84
31.31 28.54 22.35 19.50 14.14
Cl
C5H11
1e
fl (ppm)

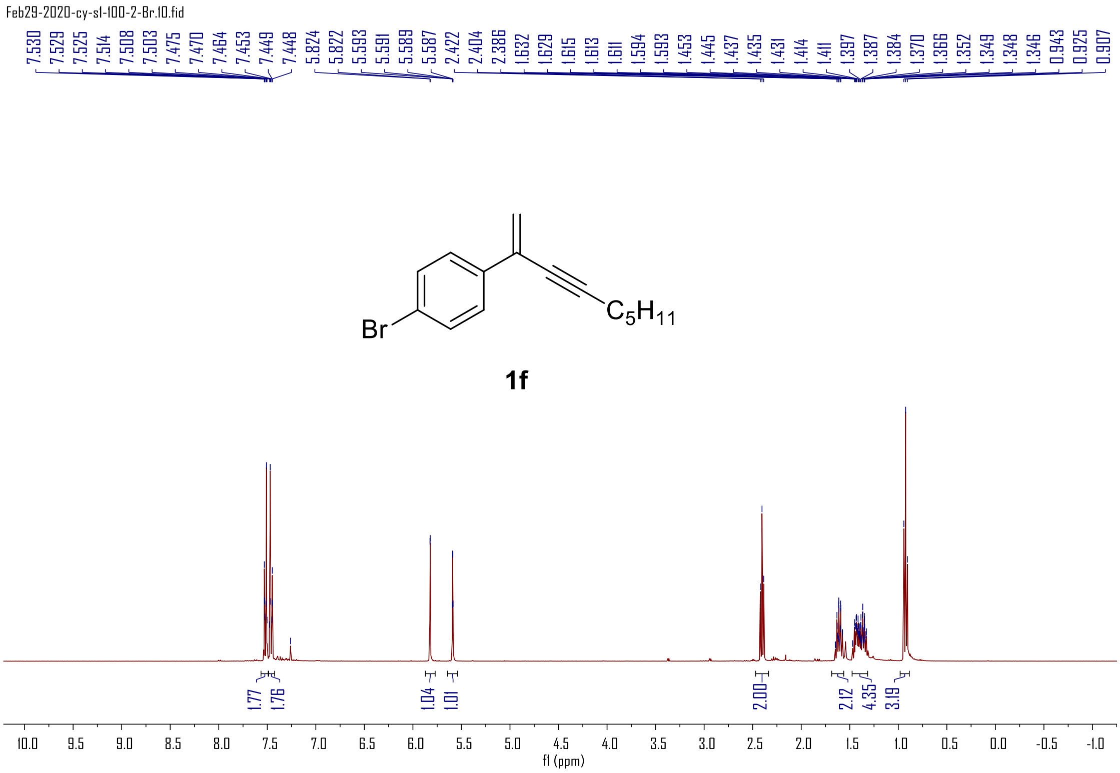
Feb29-2020-cy-sl-100-2-Br.10.fid
7.530 7.529 7.525 7.514 7.508 7.503 7.475 7.470 7.464 7.453 7.449 7.448 5.824 5.822 5.593 5.591 5.589 5.587 2.422 2.404 2.386 1.632 1.629 1.615 1.613 1.611 1.594 1.593 1.453 1.445 1.437 1.435 1.431 1.414 1.411 1.397 1.387 1.384 1.370 1.366 1.352 1.349 1.348 1.346 0.943 0.925 0.907
Br
C5H11
1f
1.77 1.76 1.04 1.01 2.00 2.12 4.35 3.19
f1 (ppm)

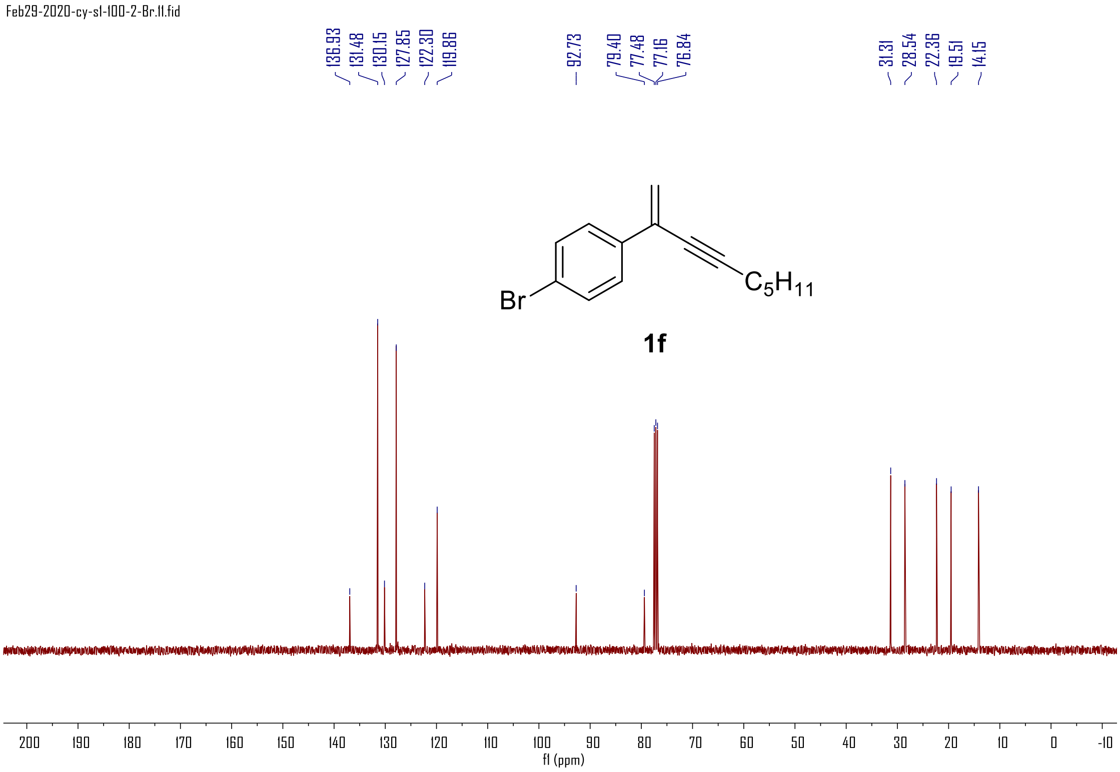
Feb29-2020-cy-sl-100-2-Br.11.fid
136.93 131.48 130.15 127.85 122.30 119.86 92.73 79.40 77.48 77.16 76.84 31.31 28.54 22.36 19.51 14.15
Br
C5H11
1f
f1 (ppm)

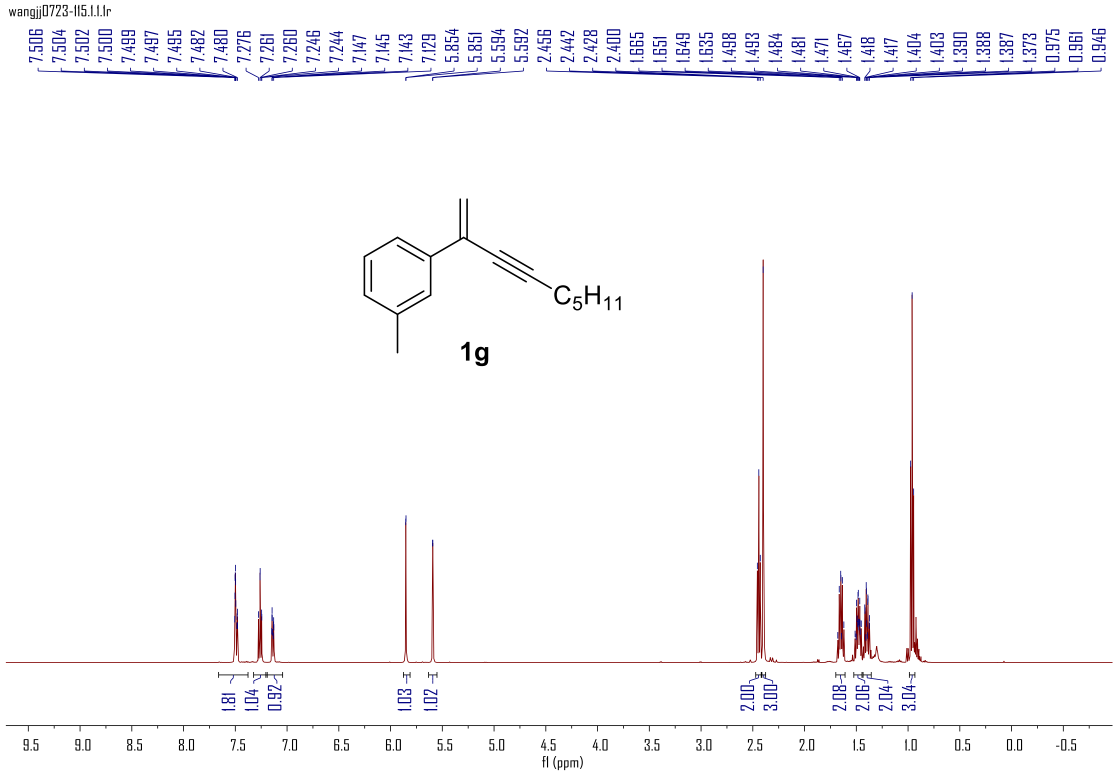
wangjj0723-115.1.1.1r
7.506 7.504 7.502 7.500 7.499 7.497 7.495 7.482 7.480 7.276 7.261 7.260 7.246 7.244 7.147 7.145 7.143 7.129 5.854 5.851 5.594 5.592 2.456 2.442 2.428 2.400 1.665 1.651 1.649 1.635 1.498 1.493 1.484 1.481 1.471 1.467 1.418 1.417 1.404 1.403 1.390 1.388 1.387 1.373 0.975 0.961 0.946
C_5H_{11}
1g
1.81 1.04 0.92 1.03 1.02 2.00 3.00 2.08 2.06 2.04 3.04
9.5 9.0 8.5 8.0 7.5 7.0 6.5 6.0 5.5 5.0 4.5 4.0 3.5 3.0 2.5 2.0 1.5 1.0 0.5 0.0 -0.5
f1 (ppm)

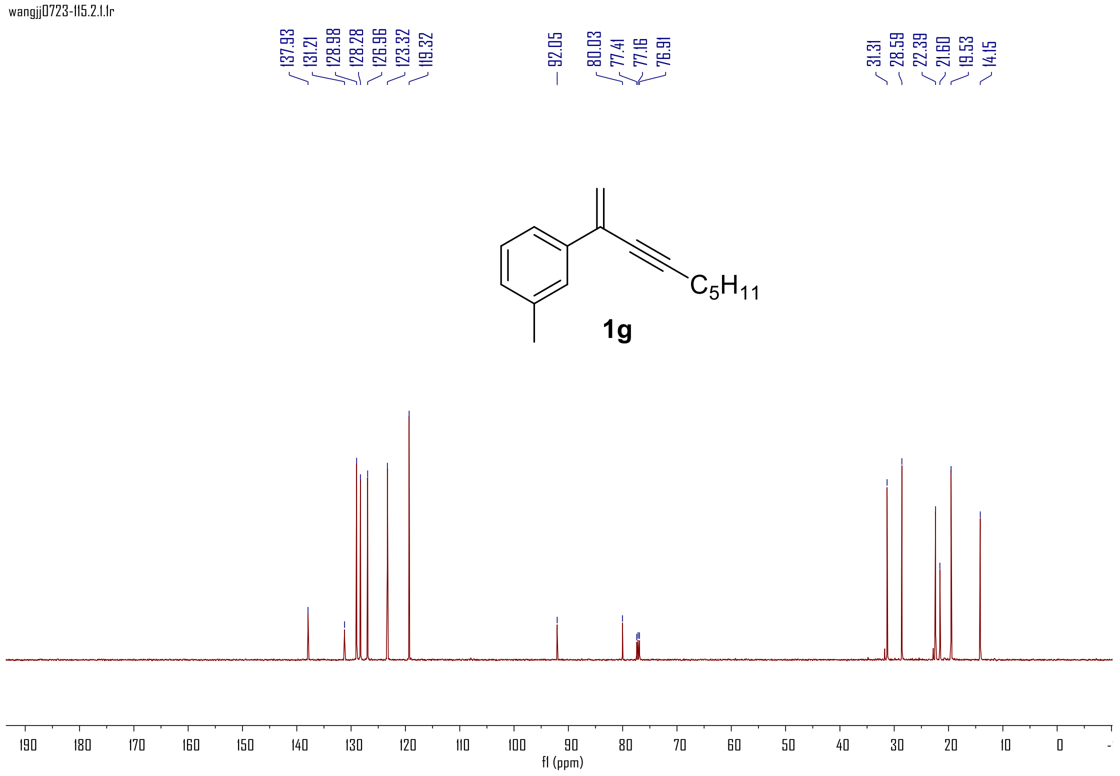
wangjj0723-115.2.1.1r
137.93 131.21 128.98 128.28 126.96 123.32 119.32 92.05 80.03 77.41 77.16 76.91 31.31 28.59 22.39 21.60 19.53 14.15
C_5H_{11}
1g
190 180 170 160 150 140 130 120 110 100 90 80 70 60 50 40 30 20 10 0
f1 (ppm)

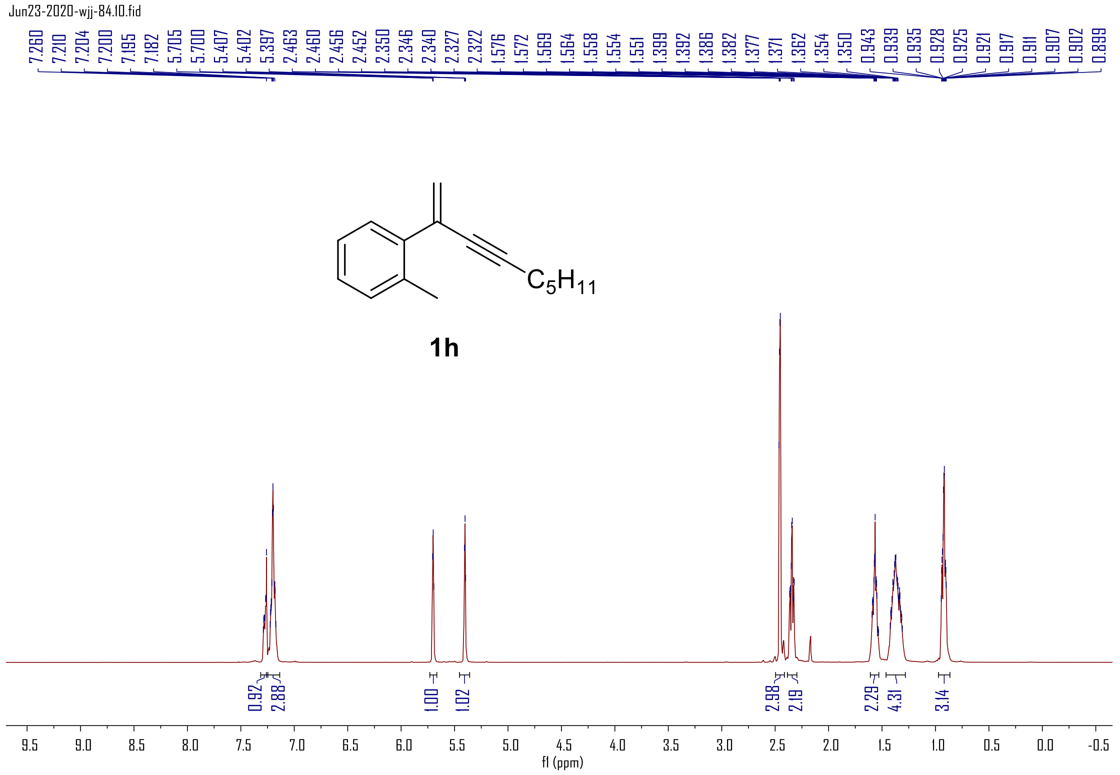
Jun23-2020-wjj-84.10.fid
7.260 7.210 7.204 7.200 7.195 7.182 5.705 5.700 5.407 5.402 5.397 2.463 2.460 2.456 2.452 2.350 2.346 2.340 2.327 2.322 1.576 1.572 1.569 1.564 1.558 1.554 1.551 1.399 1.392 1.386 1.382 1.377 1.371 1.362 1.354 1.350 0.943 0.939 0.935 0.928 0.925 0.921 0.917 0.911 0.907 0.902 0.899
C_5H_{11}
1h
0.92 2.88 1.00 1.02 2.98 2.19 2.29 4.31 3.14
f1 (ppm)

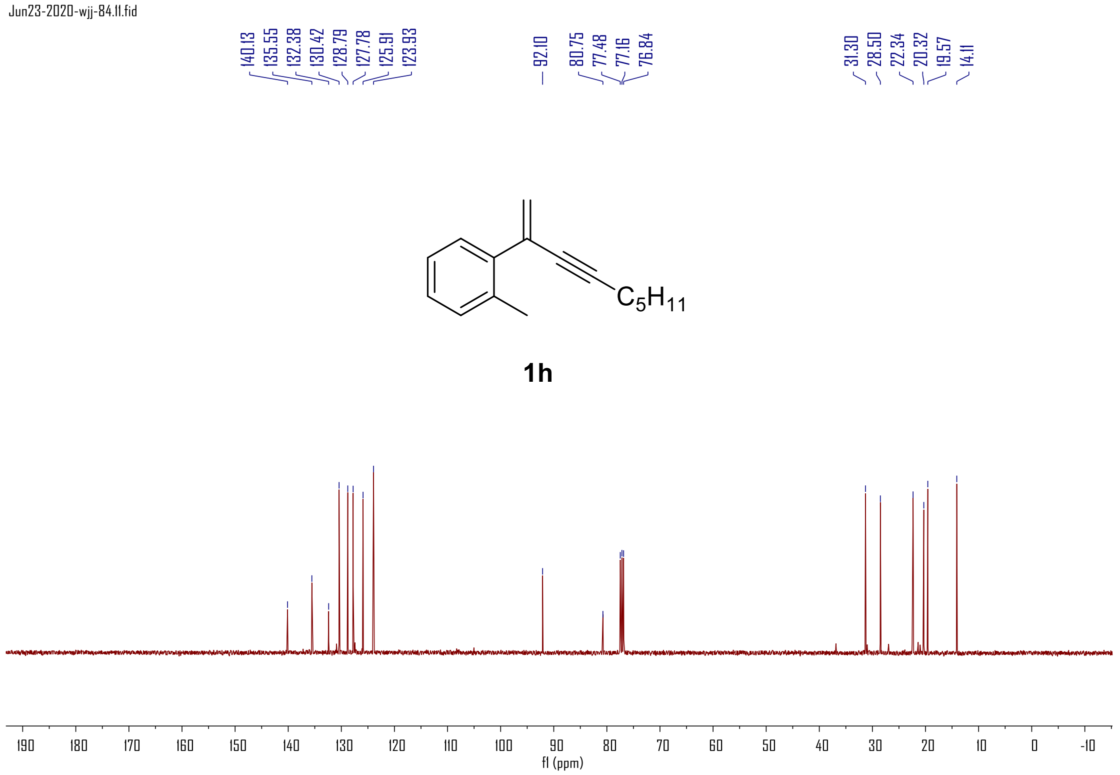
Jun23-2020-wjj-84.11.fid
140.13 135.55 132.38 130.42 128.79 127.78 125.91 123.93
92.10
80.75 77.48 77.16 76.84
31.30 28.50 22.34 20.32 19.57 14.11
C_5H_{11}
1h
f1 (ppm)

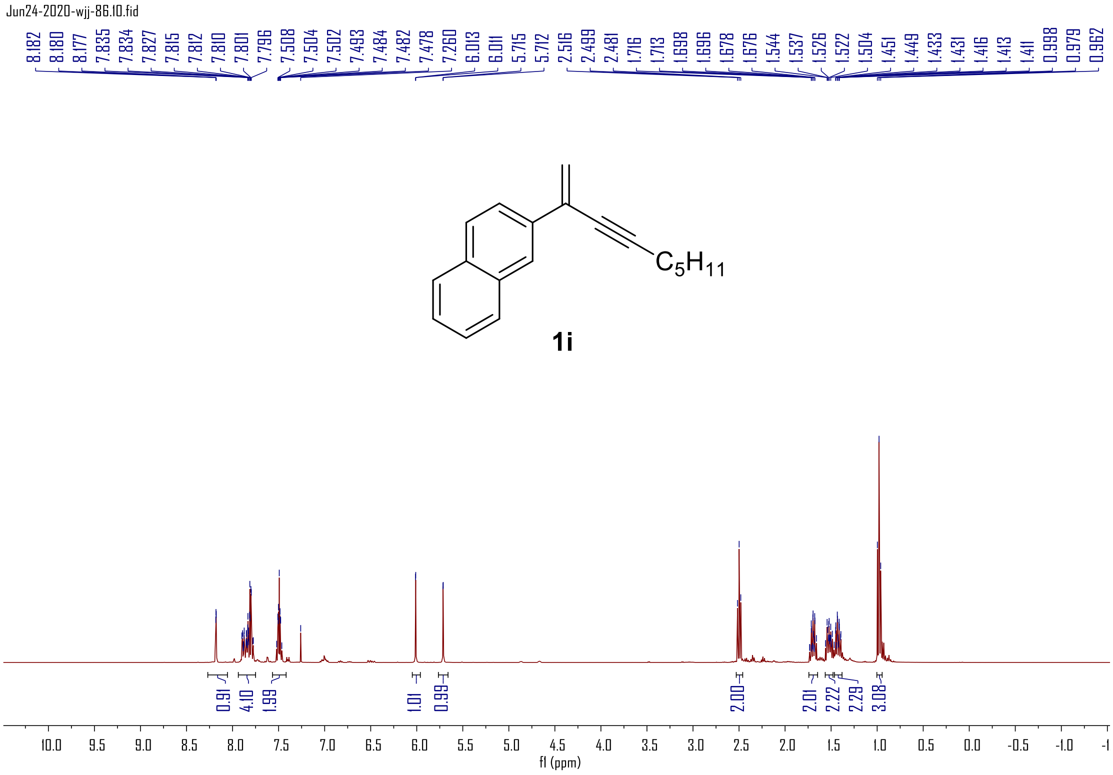
Jun24-2020-wjj-86.10.fid
8.182 8.180 8.177 7.835 7.834 7.827 7.815 7.812 7.810 7.801 7.796 7.508 7.504 7.502 7.493 7.484 7.482 7.478 7.260 6.013 6.011 5.715 5.712 2.516 2.499 2.481 1.716 1.713 1.698 1.696 1.678 1.676 1.544 1.537 1.526 1.522 1.504 1.451 1.449 1.433 1.431 1.416 1.413 1.411 0.998 0.979 0.962
C5H11
1i
0.91 4.10 1.99 1.01 0.99 2.00 2.01 2.22 2.29 3.08
10.0 9.5 9.0 8.5 8.0 7.5 7.0 6.5 6.0 5.5 5.0 4.5 4.0 3.5 3.0 2.5 2.0 1.5 1.0 0.5 0.0 -0.5 -1.0
f1 (ppm)

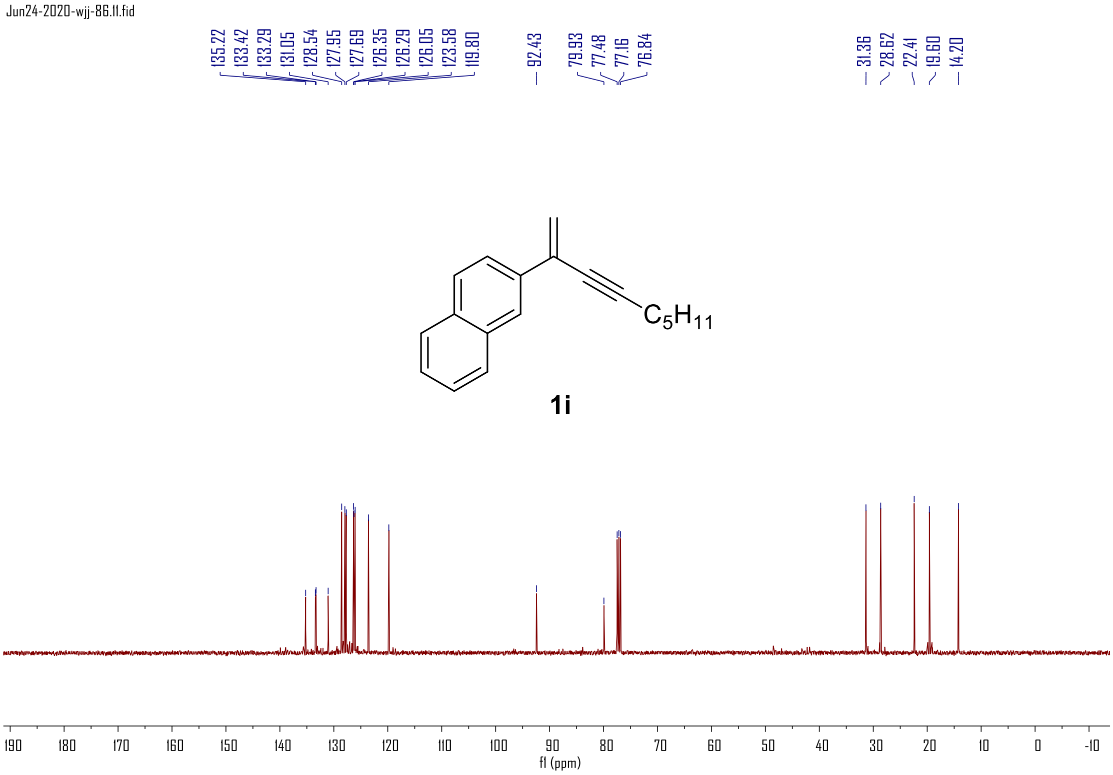
Jun24-2020-wjj-86.11.fid
135.22 133.42 133.29 131.05 128.54 127.95 127.69 126.35 126.29 126.05 123.58 119.80
92.43
79.93 77.48 77.16 76.84
31.36 28.62 22.41 19.60 14.20
C5H11
1i
190 180 170 160 150 140 130 120 110 100 90 80 70 60 50 40 30 20 10 0 -10
f1 (ppm)

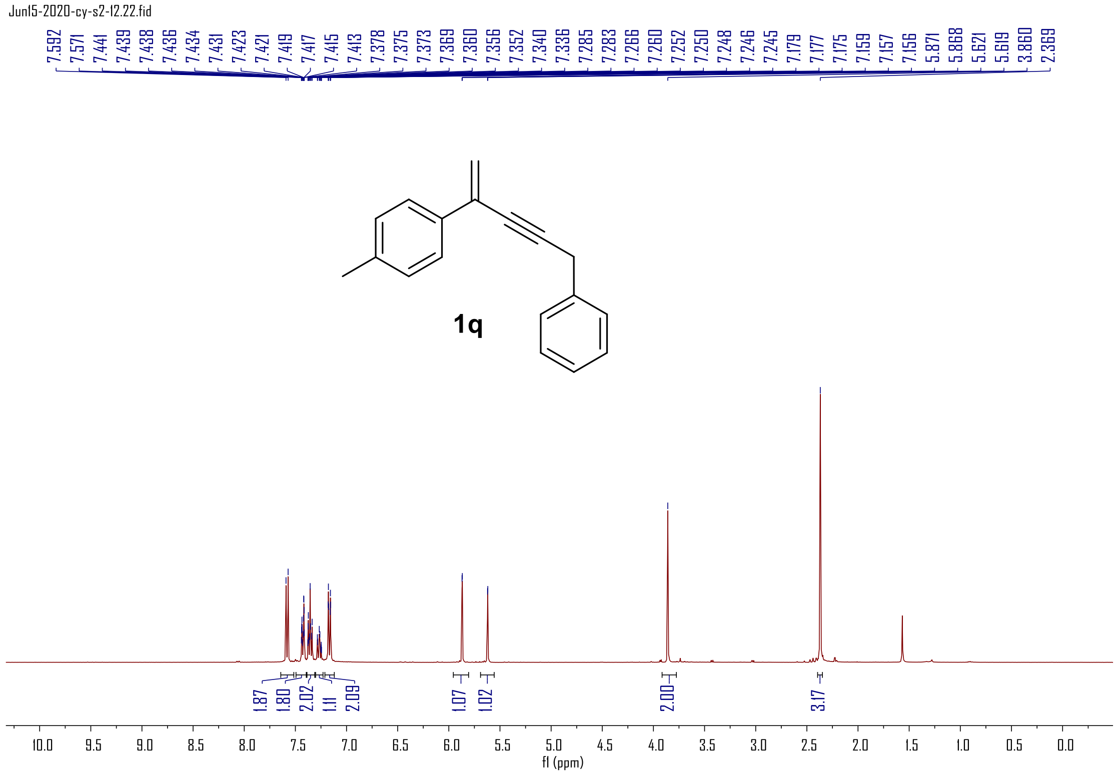
Jun15-2020-cy-s2-12.22.fid
7.592 7.571 7.441 7.439 7.438 7.436 7.434 7.431 7.423 7.421 7.419 7.417 7.415 7.413 7.378 7.375 7.373 7.369 7.360 7.356 7.352 7.340 7.336 7.285 7.283 7.266 7.260 7.252 7.250 7.248 7.246 7.245 7.179 7.177 7.175 7.159 7.157 7.156 5.871 5.868 5.621 5.619 3.860 2.369
1q
1.87 1.80 2.02 1.11 2.09 1.07 1.02 2.00 3.17
10.0 9.5 9.0 8.5 8.0 7.5 7.0 6.5 6.0 5.5 5.0 4.5 4.0 3.5 3.0 2.5 2.0 1.5 1.0 0.5 0.0
f1 (ppm)

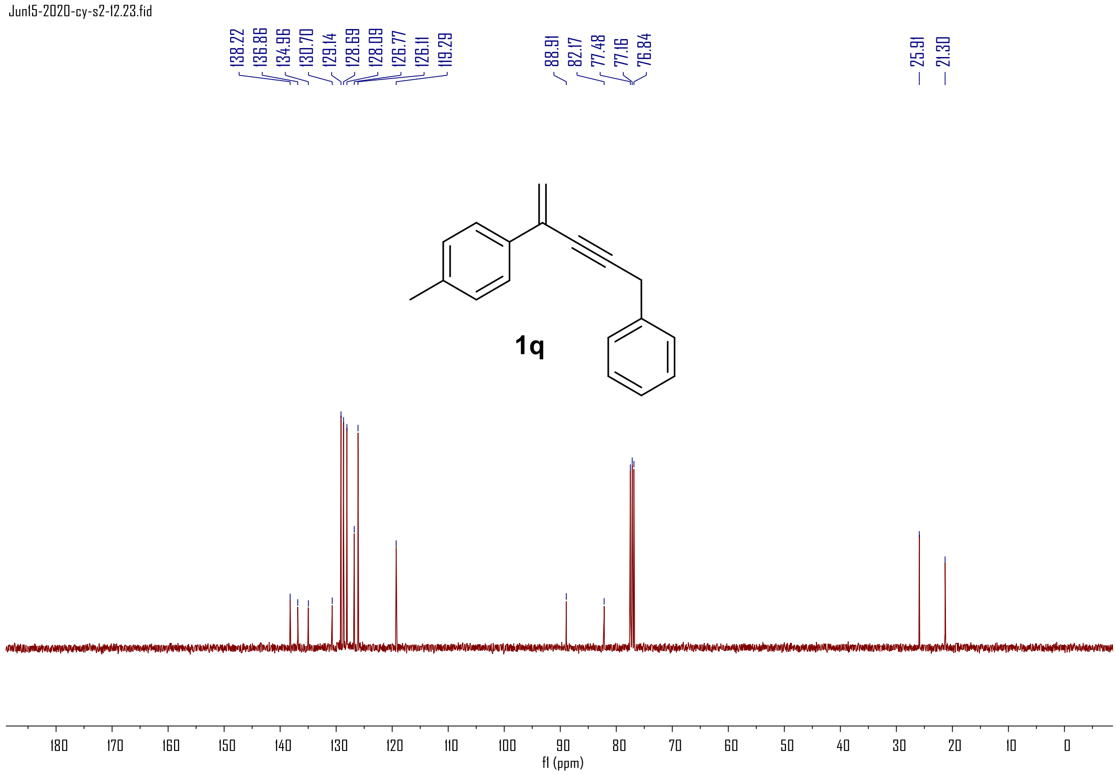
Jun15-2020-cy-s2-12.23.fid
138.22 136.86 134.96 130.70 129.14 128.69 128.09 126.77 126.11 119.29
88.91 82.17 77.48 77.16 76.84
25.91 21.30
1q
180 170 160 150 140 130 120 110 100 90 80 70 60 50 40 30 20 10 0
f1 (ppm)

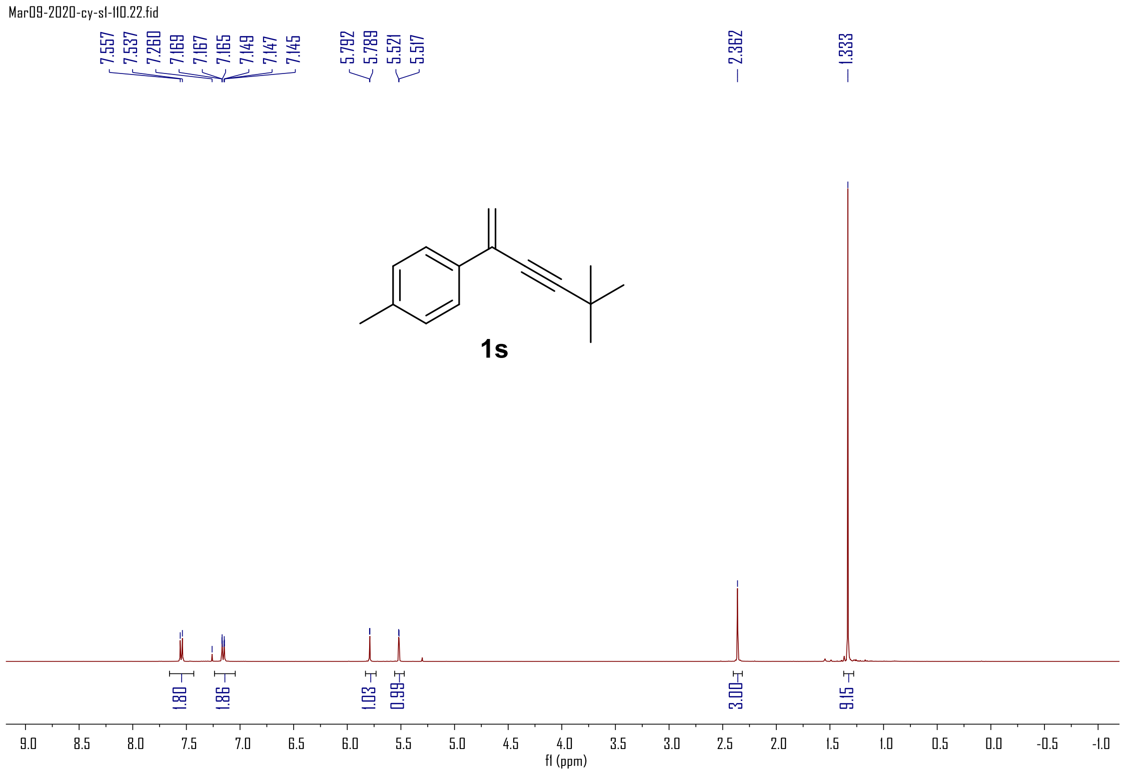
Mar09-2020-cy-sl-110.22.fid
7.557
7.537
7.260
7.169
7.167
7.165
7.149
7.147
7.145
5.792
5.789
5.521
5.517
2.362
1.333
1s
1.80
1.86
1.03
0.99
3.00
9.15
f1 (ppm)

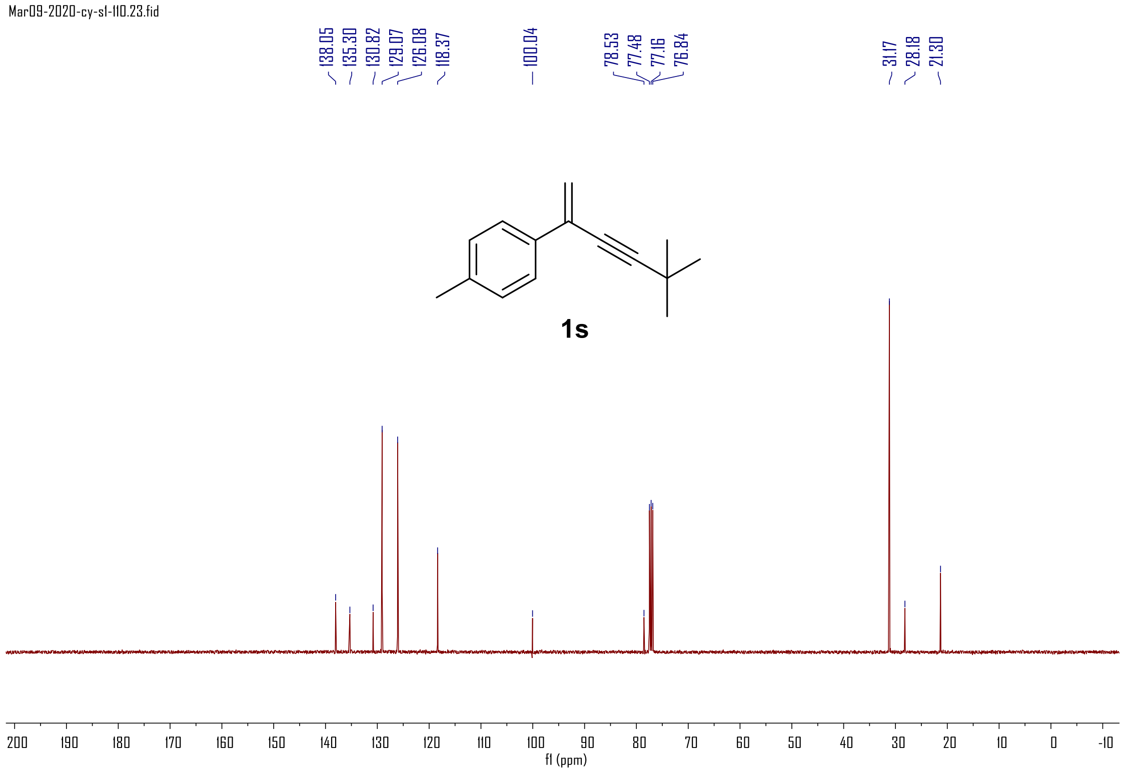
Mar09-2020-cy-sl-110.23.fid
138.05
135.30
130.82
129.07
126.08
118.37
100.04
78.53
77.48
77.16
76.84
31.17
28.18
21.30
1s
f1 (ppm)

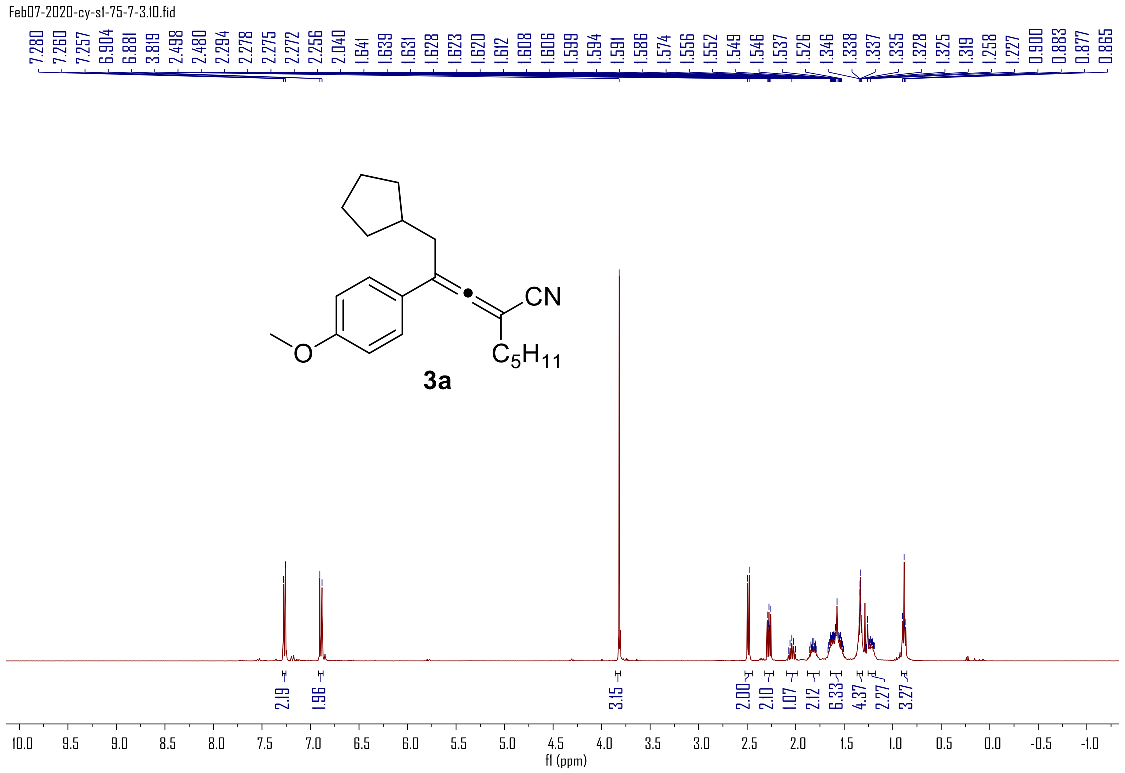
Feb07-2020-cy-sl-75-7-3.10.fid
7.280 7.260 7.257 6.904 6.881 3.819 2.498 2.480 2.294 2.278 2.275 2.272 2.256 2.040 1.641 1.639 1.631 1.628 1.623 1.620 1.612 1.608 1.606 1.599 1.594 1.591 1.586 1.574 1.556 1.552 1.549 1.546 1.537 1.526 1.346 1.338 1.337 1.335 1.328 1.325 1.319 1.258 1.227 0.900 0.883 0.877 0.865
CN
O
C_5H_{11}
3a
2.19 1.96 3.15 2.00 2.10 1.07 2.12 6.33 4.37 2.27 3.27
fl (ppm)

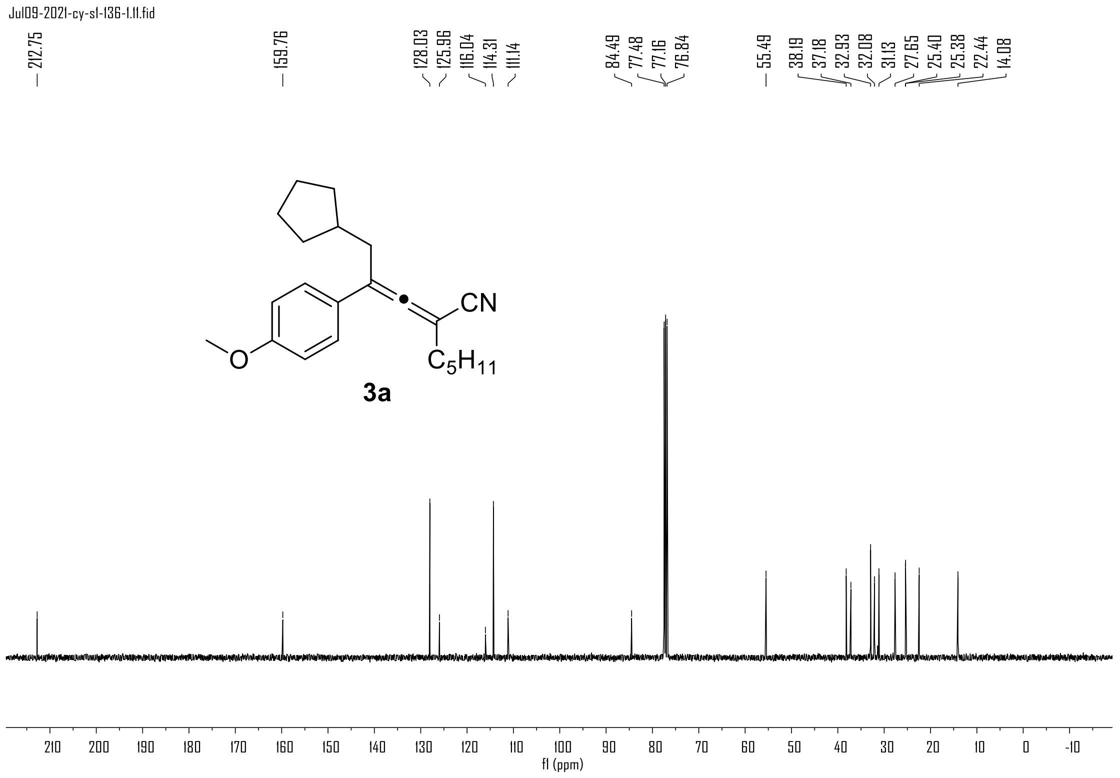
Jul09-2021-cy-sl-136-1.11.fid
212.75 159.76 128.03 125.96 116.04 114.31 111.14 84.49 77.48 77.16 76.84 55.49 38.19 37.18 32.93 32.08 31.13 27.65 25.40 25.38 22.44 14.08
CN
O
C_5H_{11}
3a
fl (ppm)

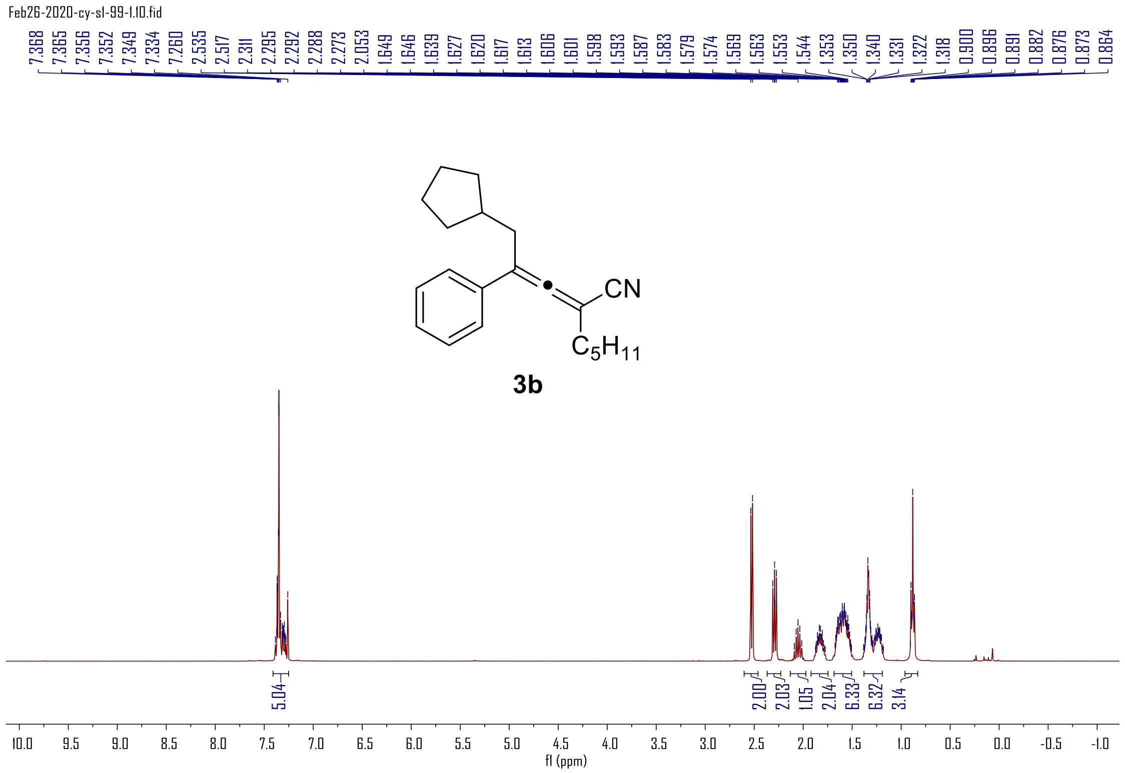

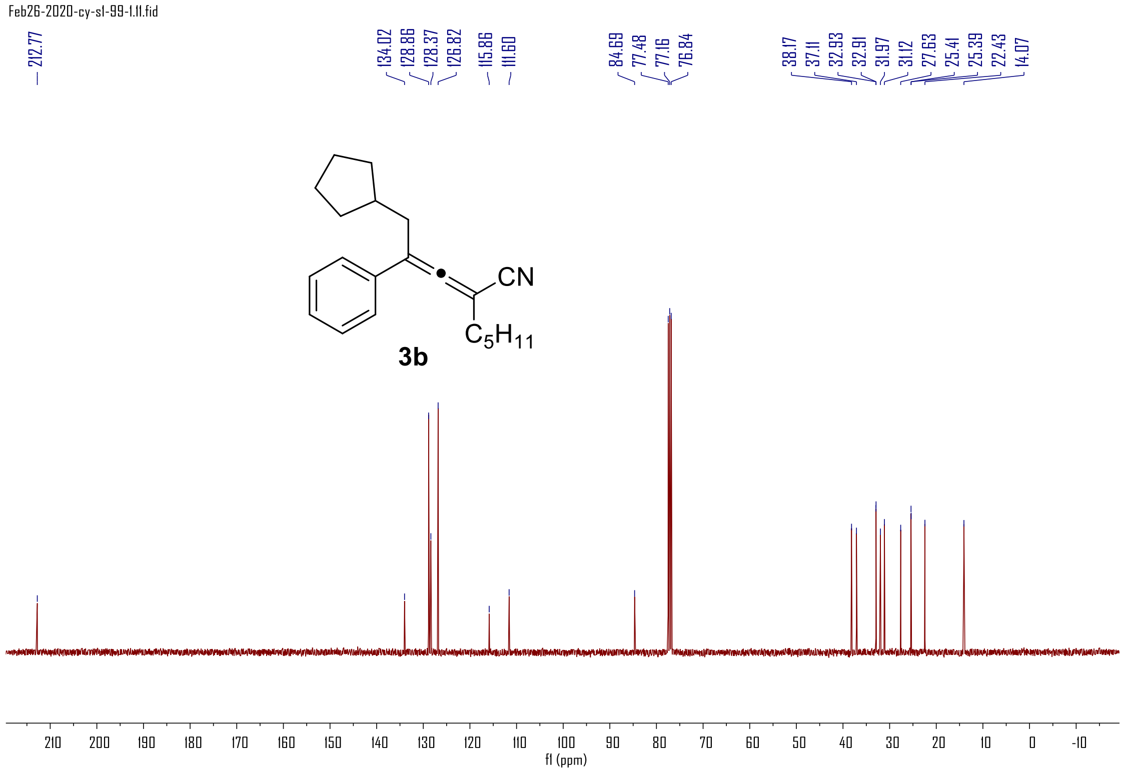

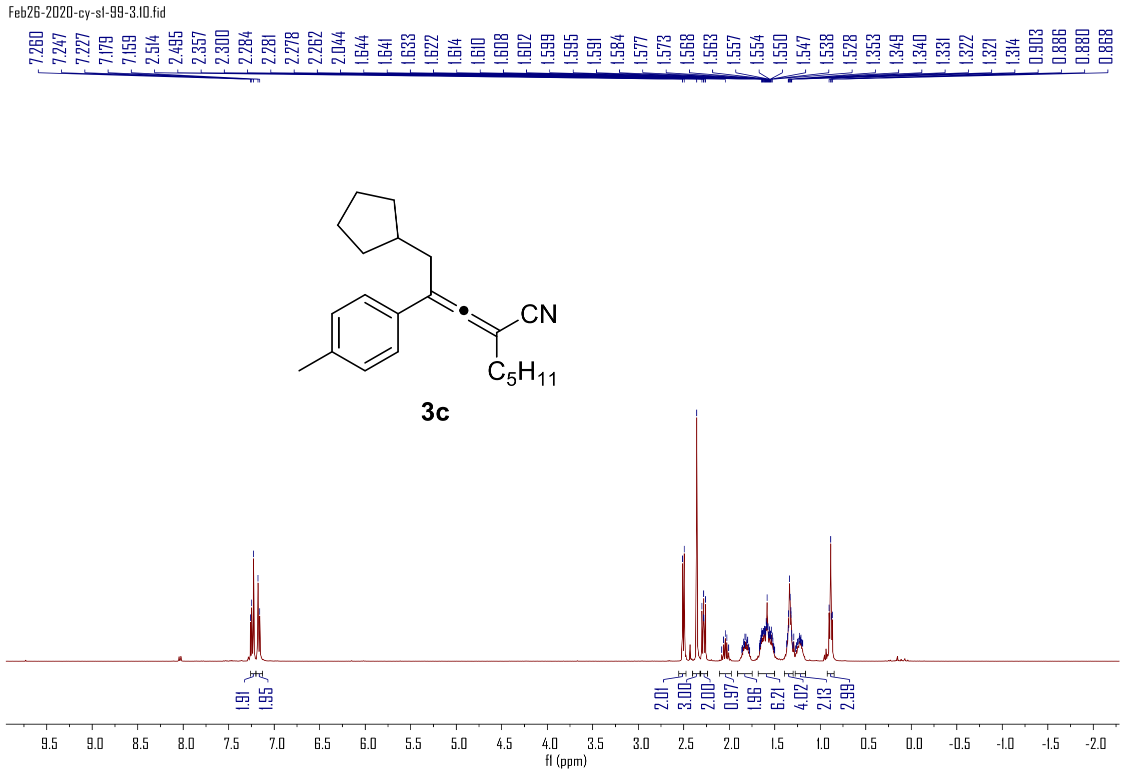
Feb26-2020-cy-sl-99-3.10.fid
7.260 7.247 7.227 7.179 7.159 2.514 2.495 2.357 2.300 2.284 2.281 2.278 2.262 2.044 1.644 1.641 1.633 1.622 1.614 1.610 1.608 1.602 1.599 1.595 1.591 1.584 1.577 1.573 1.568 1.563 1.557 1.554 1.550 1.547 1.538 1.528 1.353 1.349 1.340 1.331 1.322 1.321 1.314 0.903 0.886 0.880 0.868
CN
C_5H_{11}
3c
1.91 1.95
2.01 3.00 2.00 0.97 1.96 6.21 4.02 2.13 2.99
9.5 9.0 8.5 8.0 7.5 7.0 6.5 6.0 5.5 5.0 4.5 4.0 3.5 3.0 2.5 2.0 1.5 1.0 0.5 0.0 -0.5 -1.0 -1.5 -2.0
f1 (ppm)

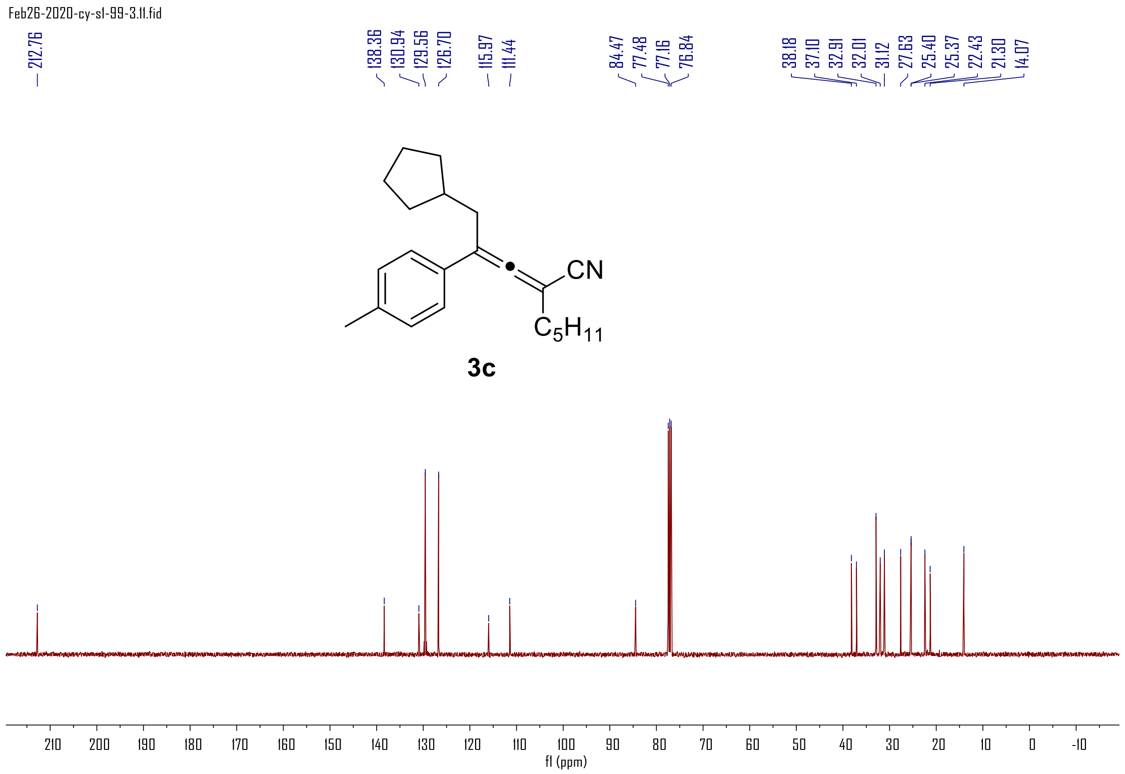
Feb26-2020-cy-sl-99-3.11.fid
212.76
138.36 130.94 129.56 126.70
115.97 111.44
84.47 77.48 77.16 76.84
38.18 37.10 32.91 32.01 31.12 27.63 25.40 25.37 22.43 21.30 14.07
CN
C_5H_{11}
3c
210 200 190 180 170 160 150 140 130 120 110 100 90 80 70 60 50 40 30 20 10 0 -10
f1 (ppm)

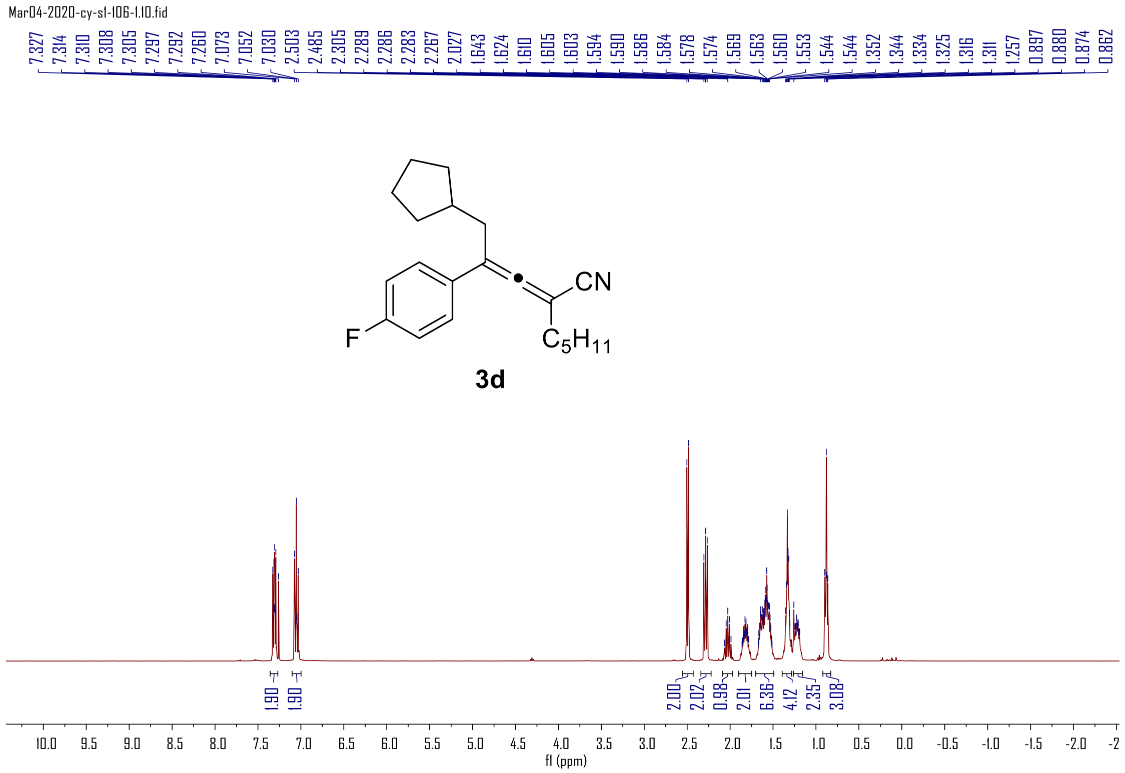
Mar04-2020-cy-sl-106-1.10.fid
7.327 7.314 7.310 7.308 7.305 7.297 7.292 7.260 7.073 7.052 7.030 2.503 2.485 2.305 2.289 2.286 2.283 2.267 2.027 1.643 1.624 1.610 1.605 1.603 1.594 1.590 1.586 1.584 1.578 1.574 1.569 1.563 1.560 1.553 1.544 1.544 1.352 1.344 1.334 1.325 1.316 1.311 1.257 0.897 0.880 0.874 0.862
CN
F
C5H11
3d
1.90 1.90 2.00 2.02 0.98 2.01 6.36 4.12 2.35 3.08
10.0 9.5 9.0 8.5 8.0 7.5 7.0 6.5 6.0 5.5 5.0 4.5 4.0 3.5 3.0 2.5 2.0 1.5 1.0 0.5 0.0 -0.5 -1.0 -1.5 -2.0 -2
f1 (ppm)

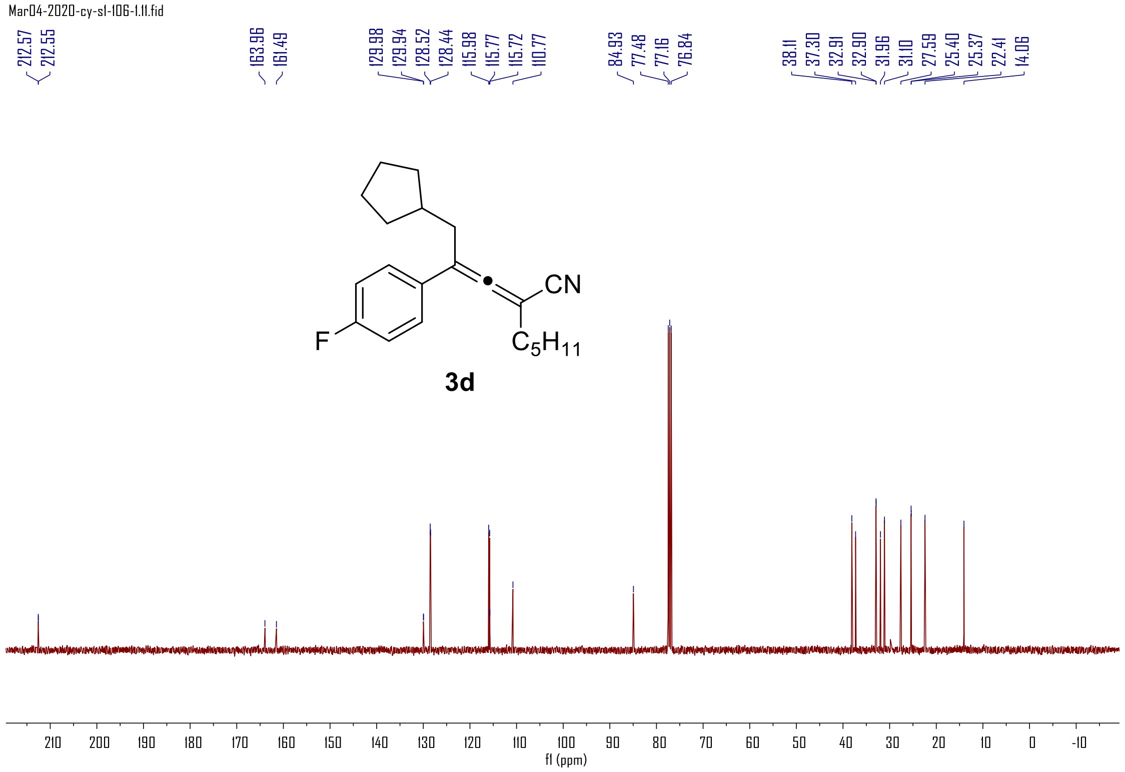
Mar04-2020-cy-sl-106-1.11.fid
212.57 212.55 163.96 161.49 129.98 129.94 128.52 128.44 115.98 115.77 115.72 110.77 84.93 77.48 77.16 76.84 38.11 37.30 32.91 32.90 31.96 31.10 27.59 25.40 25.37 22.41 14.06
CN
F
C5H11
3d
210 200 190 180 170 160 150 140 130 120 110 100 90 80 70 60 50 40 30 20 10 0 -10
f1 (ppm)

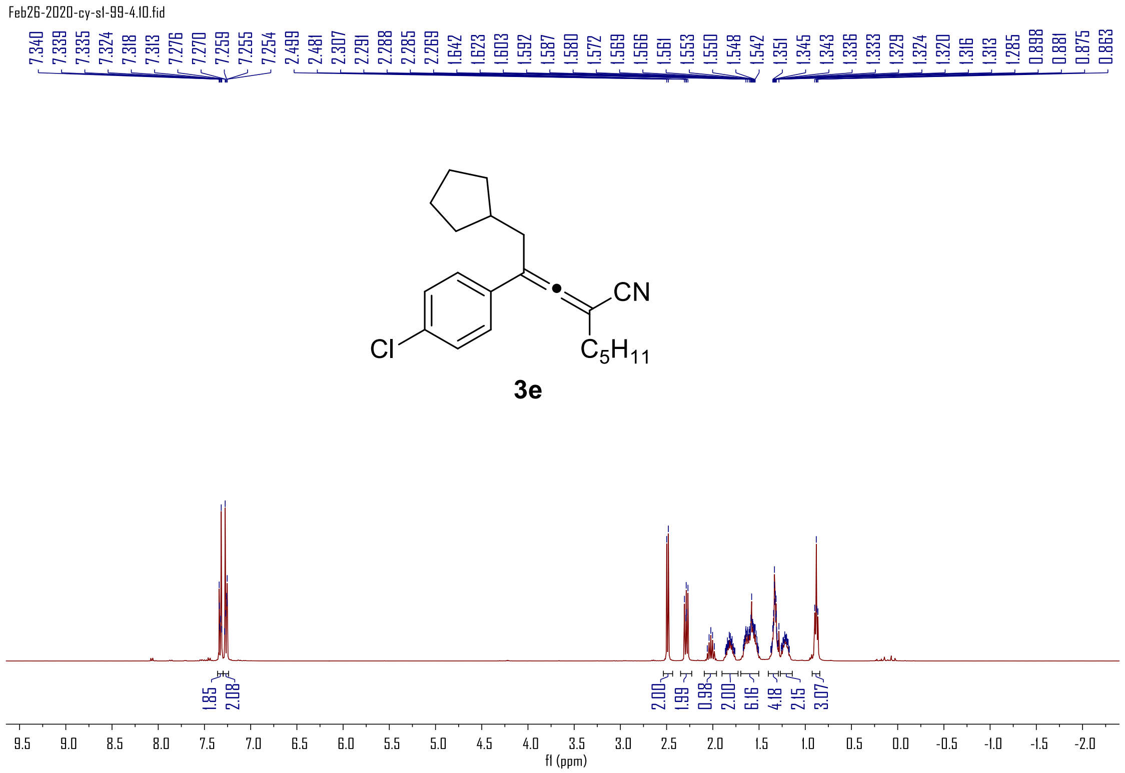
Feb26-2020-cy-sl-99-4.10.fid
7.340 7.339 7.335 7.324 7.318 7.313 7.276 7.270 7.259 7.255 7.254 2.499 2.481 2.307 2.291 2.288 2.285 2.269 1.642 1.623 1.603 1.592 1.587 1.580 1.572 1.569 1.566 1.561 1.553 1.550 1.548 1.542 1.351 1.345 1.343 1.336 1.333 1.329 1.324 1.320 1.316 1.313 1.285 0.898 0.881 0.875 0.863
CN
Cl
C_5H_{11}
3e
1.85 2.08 2.00 1.99 0.98 2.00 6.16 4.18 2.15 3.07
9.5 9.0 8.5 8.0 7.5 7.0 6.5 6.0 5.5 5.0 4.5 4.0 3.5 3.0 2.5 2.0 1.5 1.0 0.5 0.0 -0.5 -1.0 -1.5 -2.0
f1 (ppm)

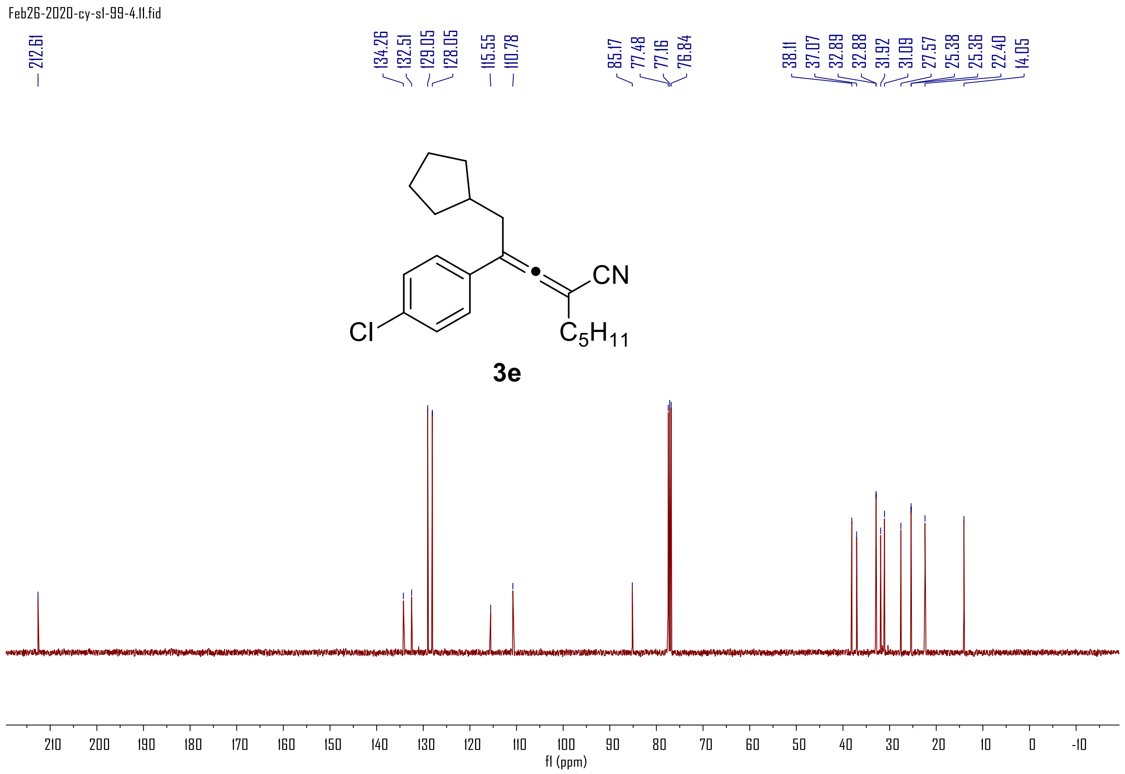
Feb26-2020-cy-sl-99-4.11.fid
212.61 134.26 132.51 129.05 128.05 115.55 110.78 85.17 77.48 77.16 76.84 38.11 37.07 32.89 32.88 31.92 31.09 27.57 25.38 25.36 22.40 14.05
CN
Cl
C_5H_{11}
3e
210 200 190 180 170 160 150 140 130 120 110 100 90 80 70 60 50 40 30 20 10 0 -10
f1 (ppm)

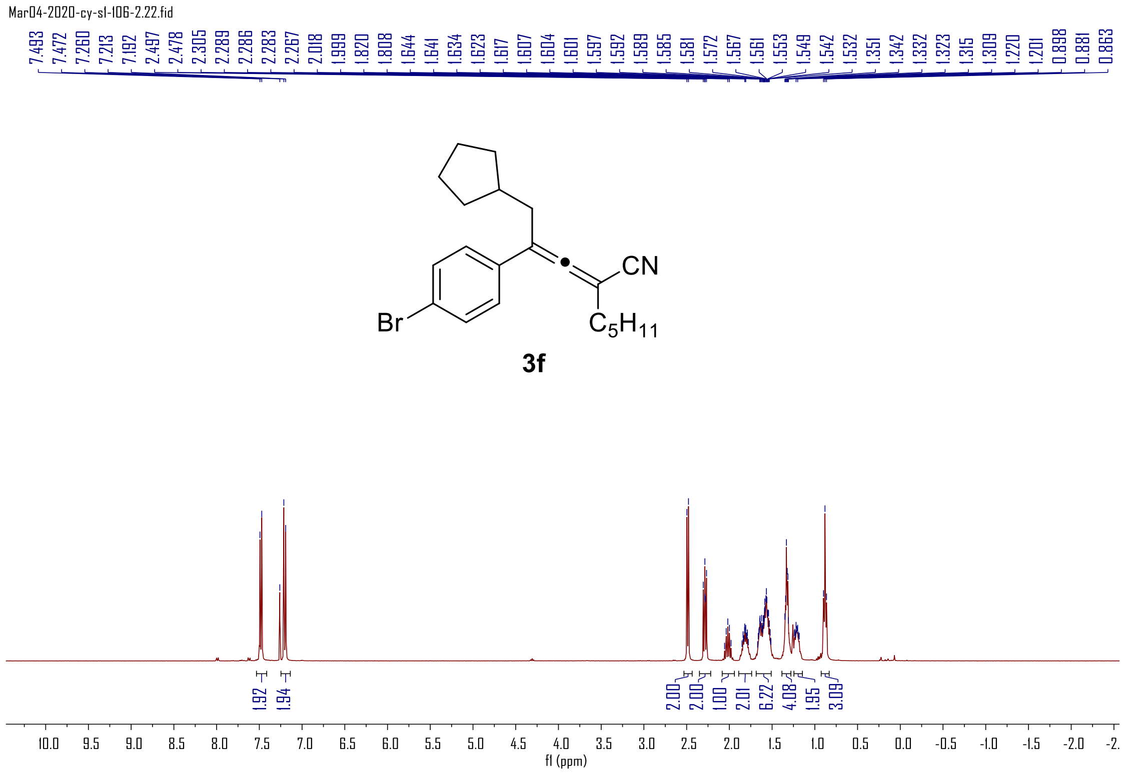
Mar04-2020-cy-sl-106-2.22.fid
7.493 7.472 7.260 7.213 7.192 2.497 2.478 2.305 2.289 2.286 2.283 2.267 2.018 1.999 1.820 1.808 1.644 1.641 1.634 1.623 1.617 1.607 1.604 1.601 1.597 1.592 1.589 1.585 1.581 1.572 1.567 1.561 1.553 1.549 1.542 1.532 1.351 1.342 1.332 1.323 1.315 1.309 1.220 1.201 0.898 0.881 0.863
CN
Br
C_5H_{11}
3f
1.92 1.94 2.00 2.00 1.00 2.01 6.22 4.08 1.95 3.09
f1 (ppm)

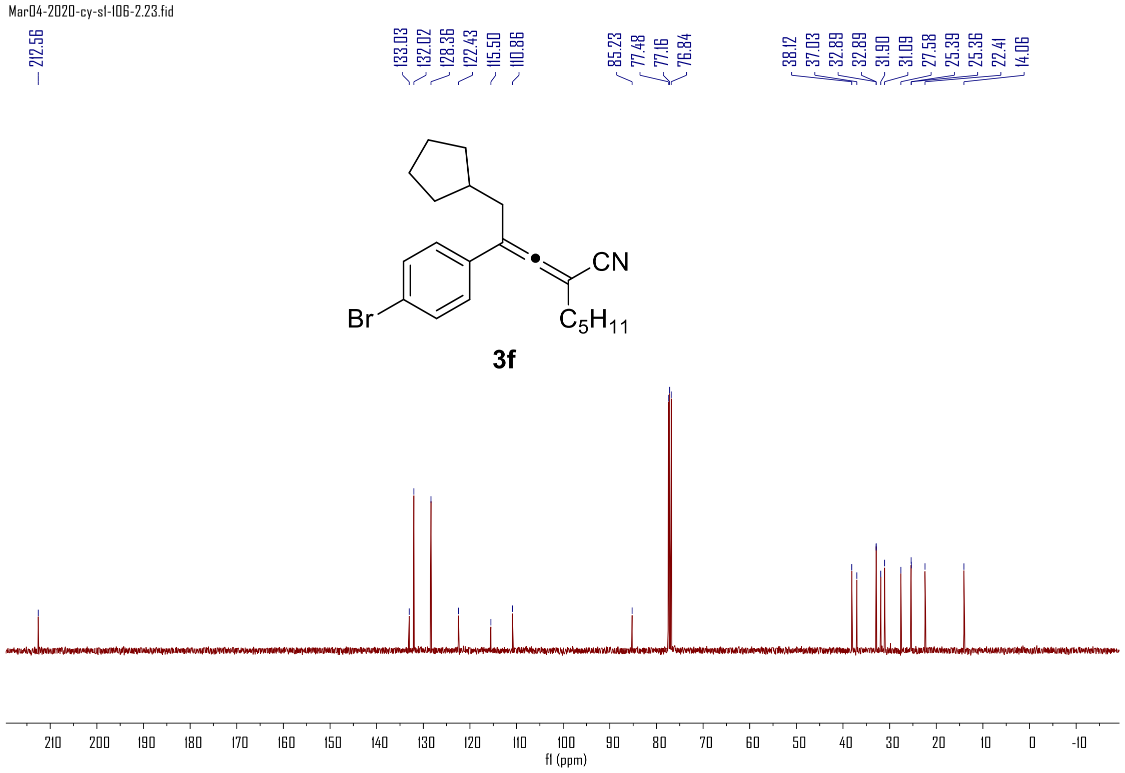
Mar04-2020-cy-sl-106-2.23.fid
212.56
133.03 132.02 128.36 122.43 115.50 110.86
85.23 77.48 77.16 76.84
38.12 37.03 32.89 32.89 31.90 31.09 27.58 25.39 25.36 22.41 14.06
CN
Br
C_5H_{11}
3f
f1 (ppm)

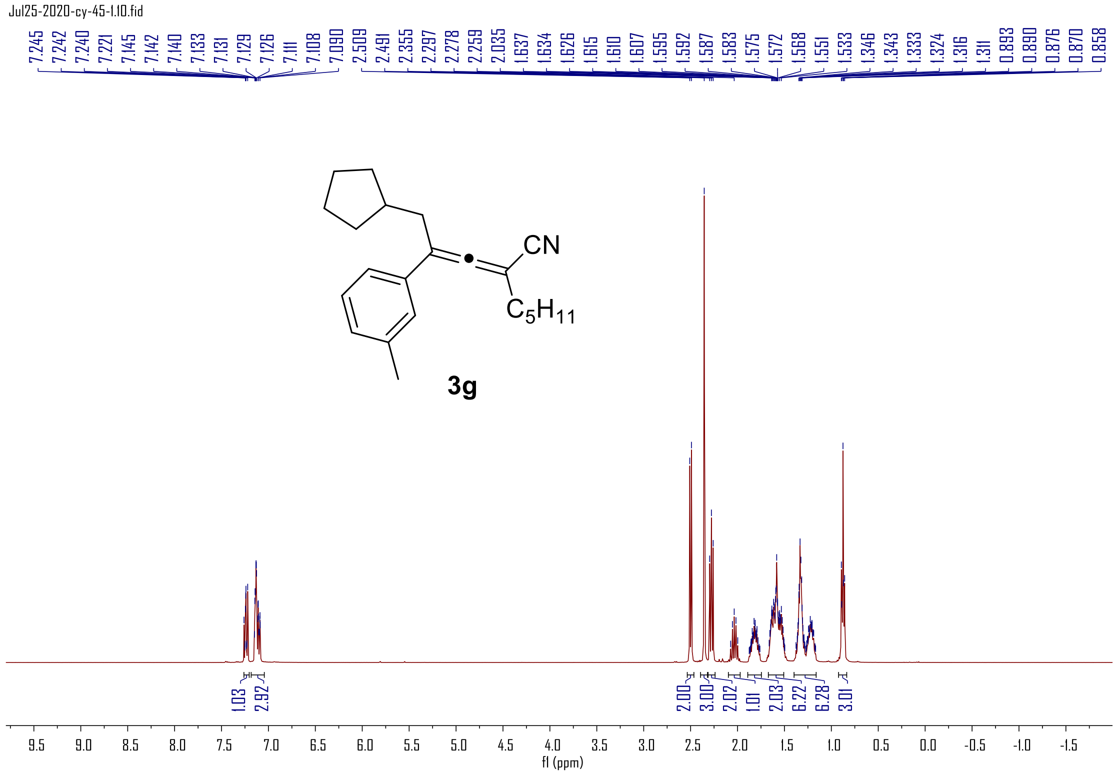
Jul25-2020-cy-45-1.10.fid
7.245 7.242 7.240 7.221 7.145 7.142 7.140 7.133 7.131 7.129 7.126 7.111 7.108 7.090 2.509 2.491 2.355 2.297 2.278 2.259 2.035 1.637 1.634 1.626 1.615 1.610 1.607 1.595 1.592 1.587 1.583 1.575 1.572 1.568 1.551 1.533 1.346 1.343 1.333 1.324 1.316 1.311 0.893 0.890 0.876 0.870 0.858
CN
C5H11
3g
1.03 2.92 2.00 3.00 2.02 1.01 2.03 6.22 6.28 3.01
fl (ppm)

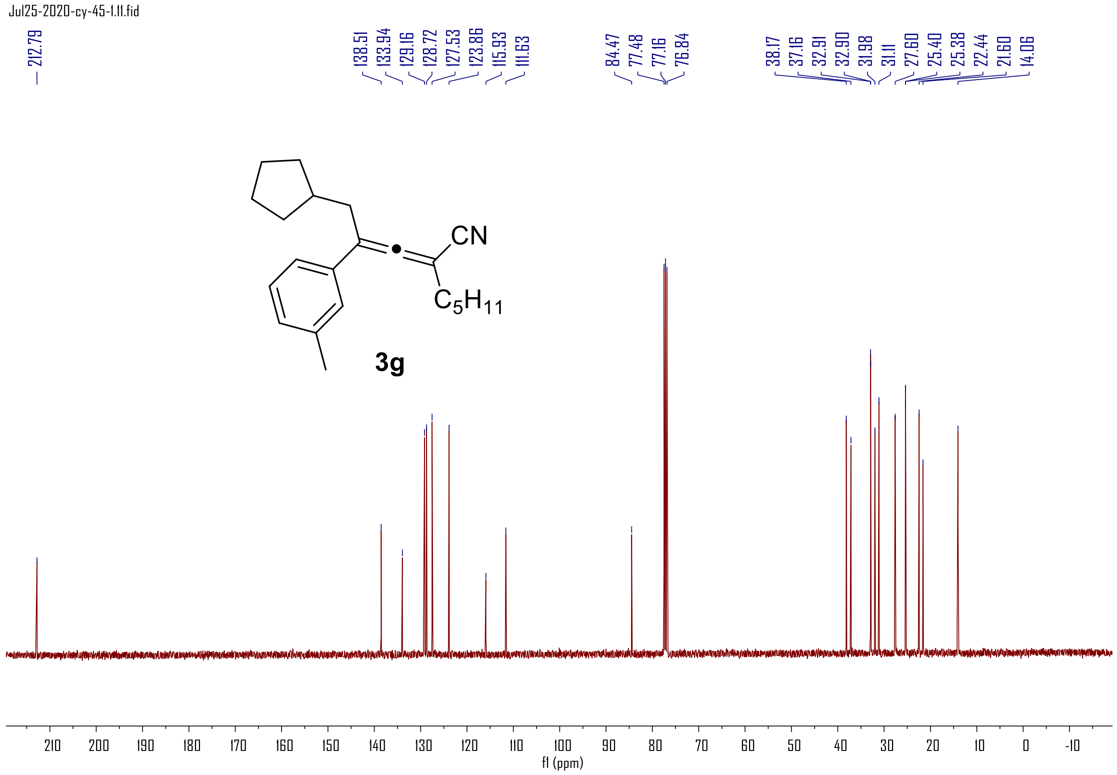
Jul25-2020-cy-45-1.11.fid
212.79 138.51 133.94 129.16 128.72 127.53 123.86 115.93 111.63 84.47 77.48 77.16 76.84 38.17 37.16 32.91 32.90 31.98 31.11 27.60 25.40 25.38 22.44 21.60 14.06
CN
C5H11
3g
fl (ppm)

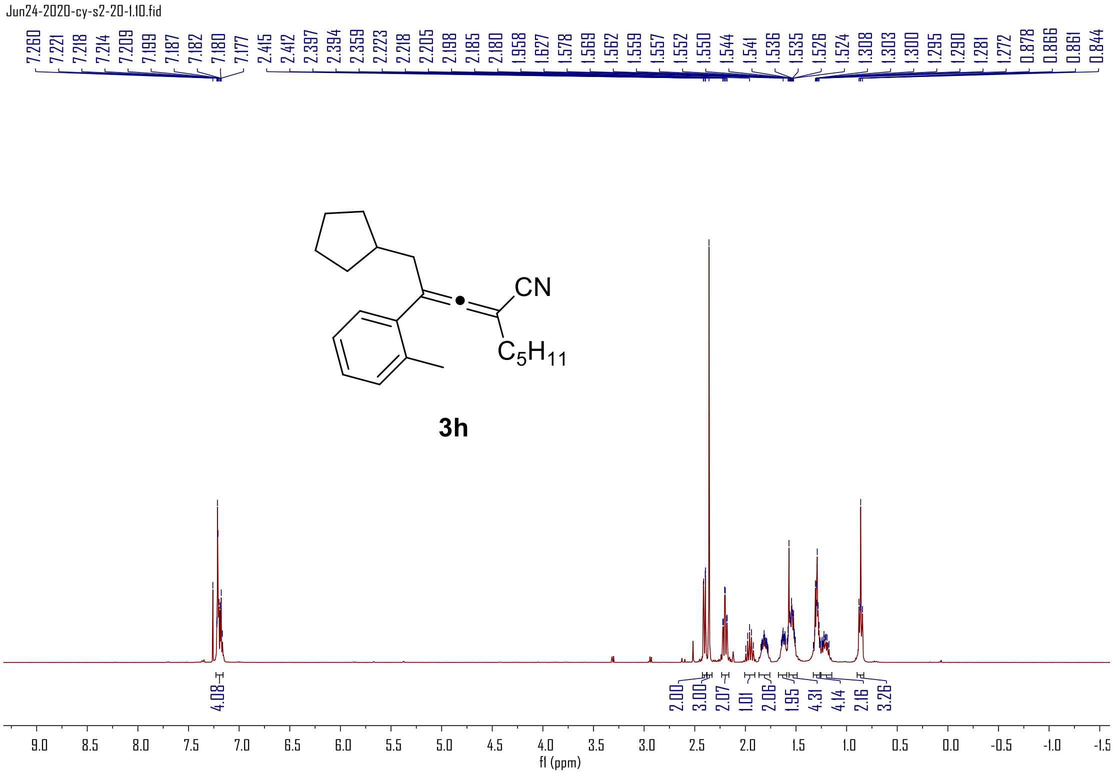
Jun24-2020-cy-s2-20-1.10.fid
7.260 7.221 7.218 7.214 7.209 7.199 7.187 7.182 7.180 7.177 2.415 2.412 2.397 2.394 2.359 2.223 2.218 2.205 2.198 2.185 2.180 1.958 1.627 1.578 1.569 1.562 1.559 1.557 1.552 1.550 1.544 1.541 1.536 1.535 1.526 1.524 1.308 1.303 1.300 1.295 1.290 1.281 1.272 0.878 0.866 0.861 0.844
CN
C_5H_{11}
3h
4.08
2.00 3.00 2.07 1.01 2.06 1.95 4.31 4.14 2.16 3.26
9.0 8.5 8.0 7.5 7.0 6.5 6.0 5.5 5.0 4.5 4.0 3.5 3.0 2.5 2.0 1.5 1.0 0.5 0.0 -0.5 -1.0 -1.5
f1 (ppm)

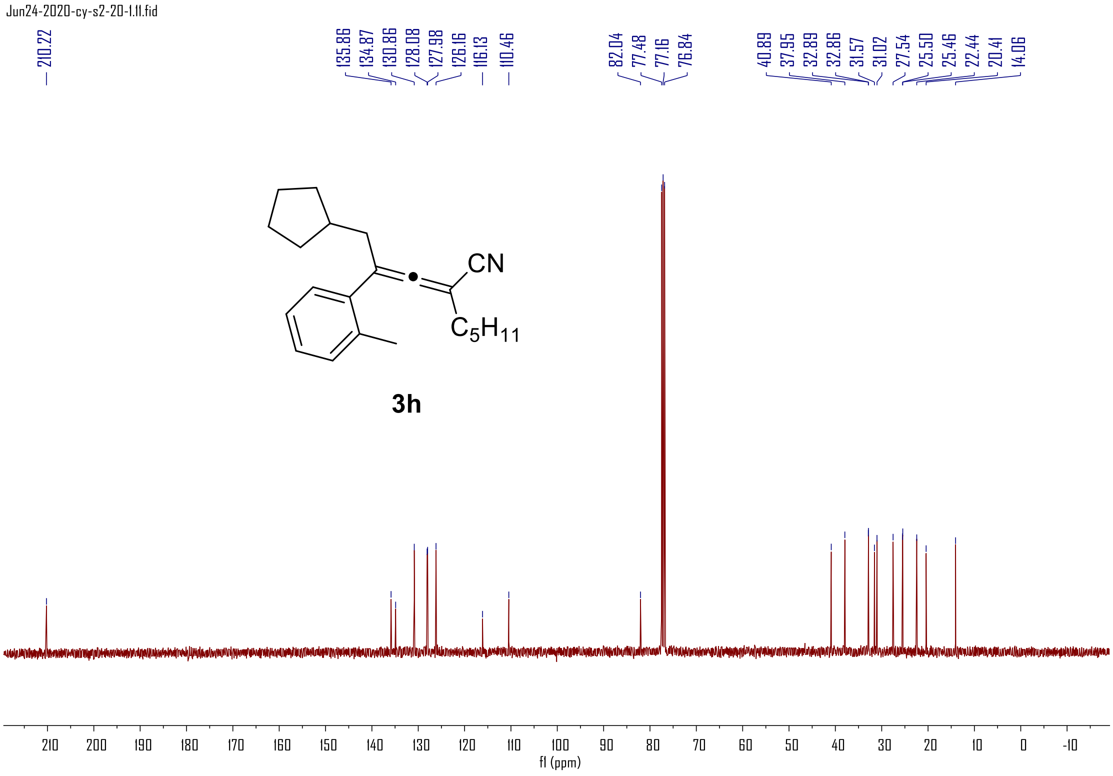
Jun24-2020-cy-s2-20-1.11.fid
210.22
135.86 134.87 130.86 128.08 127.98 126.16 116.13 110.46
82.04 77.48 77.16 76.84
40.89 37.95 32.89 32.86 31.57 31.02 27.54 25.50 25.46 22.44 20.41 14.06
CN
C_5H_{11}
3h
210 200 190 180 170 160 150 140 130 120 110 100 90 80 70 60 50 40 30 20 10 0 -10
f1 (ppm)

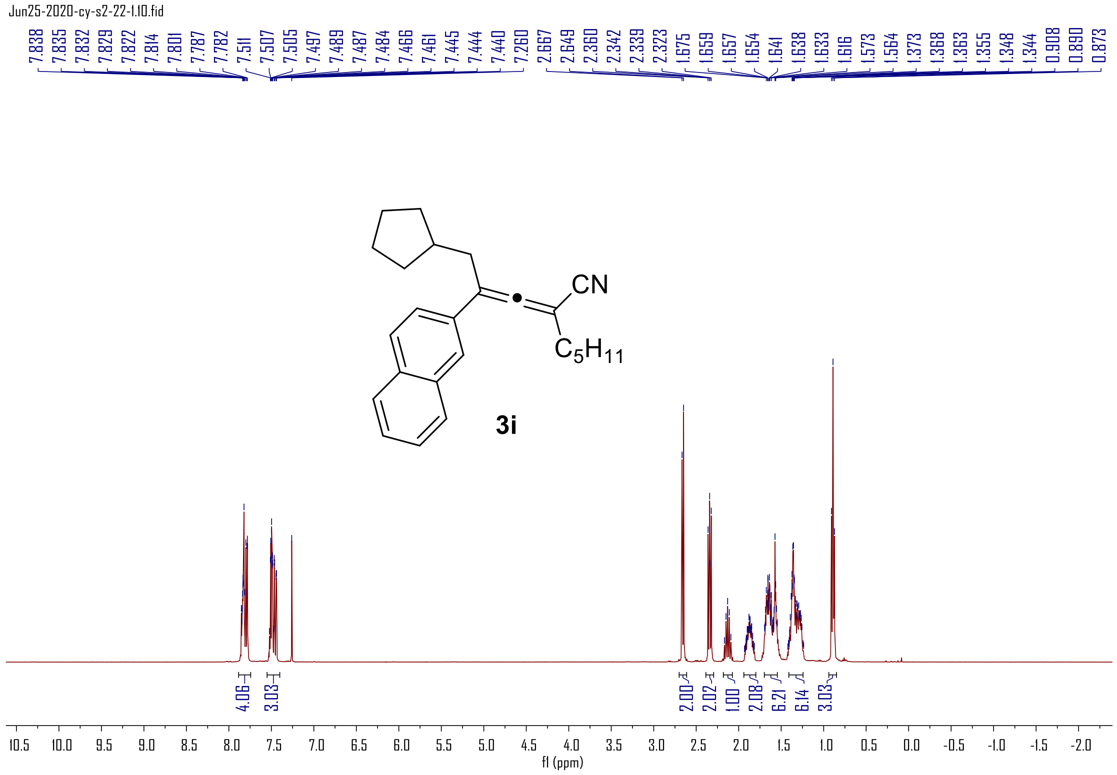

Jun25-2020-cy-s2-22-1.10.fid
7.838 7.835 7.832 7.829 7.822 7.814 7.801 7.787 7.782 7.511 7.507 7.505 7.497 7.489 7.487 7.484 7.466 7.461 7.445 7.444 7.440 7.260 2.667 2.649 2.360 2.342 2.339 2.323 1.675 1.659 1.657 1.654 1.641 1.638 1.633 1.616 1.573 1.564 1.373 1.368 1.363 1.355 1.348 1.344 0.908 0.890 0.873
CN
C_5H_{11}
3i
4.06 3.03 2.00 2.02 1.00 2.08 6.21 6.14 3.03
fl (ppm)

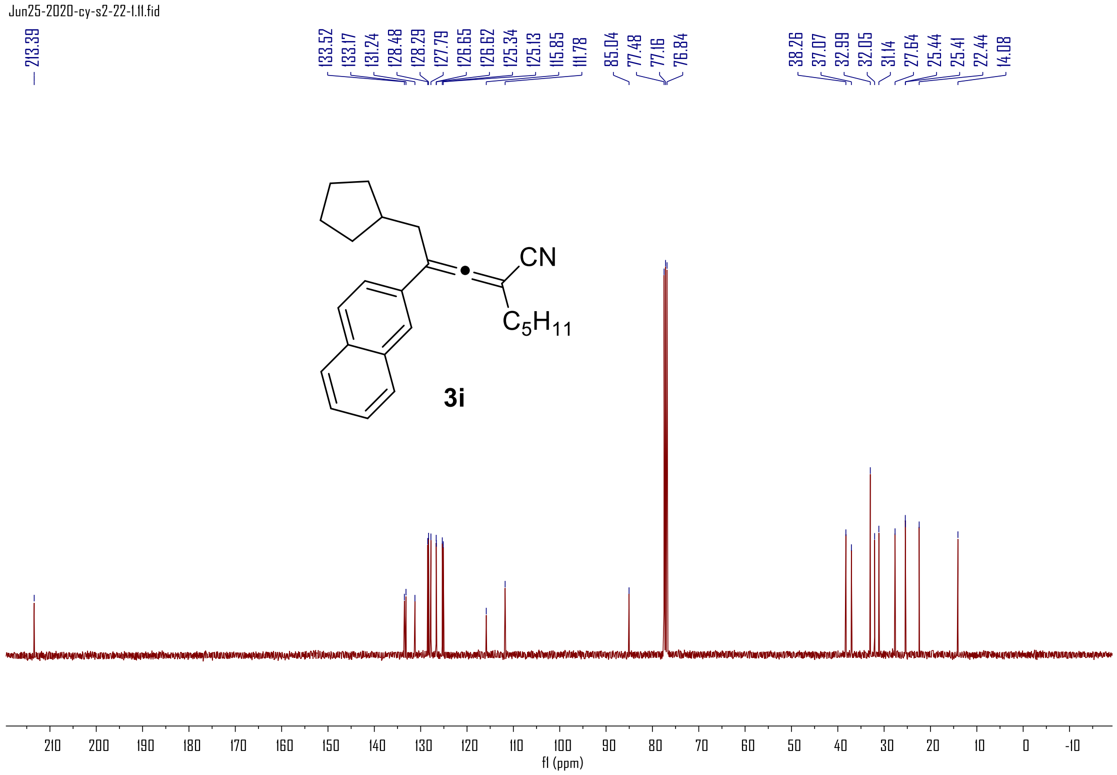

Jun25-2020-cy-s2-22-1.11.fid
213.39
133.52 133.17 131.24 128.48 128.29 127.79 126.65 126.62 125.34 125.13 115.85 111.78 85.04 77.48 77.16 76.84
38.26 37.07 32.99 32.05 31.14 27.64 25.44 25.41 22.44 14.08
CN
C_5H_{11}
3i
fl (ppm)

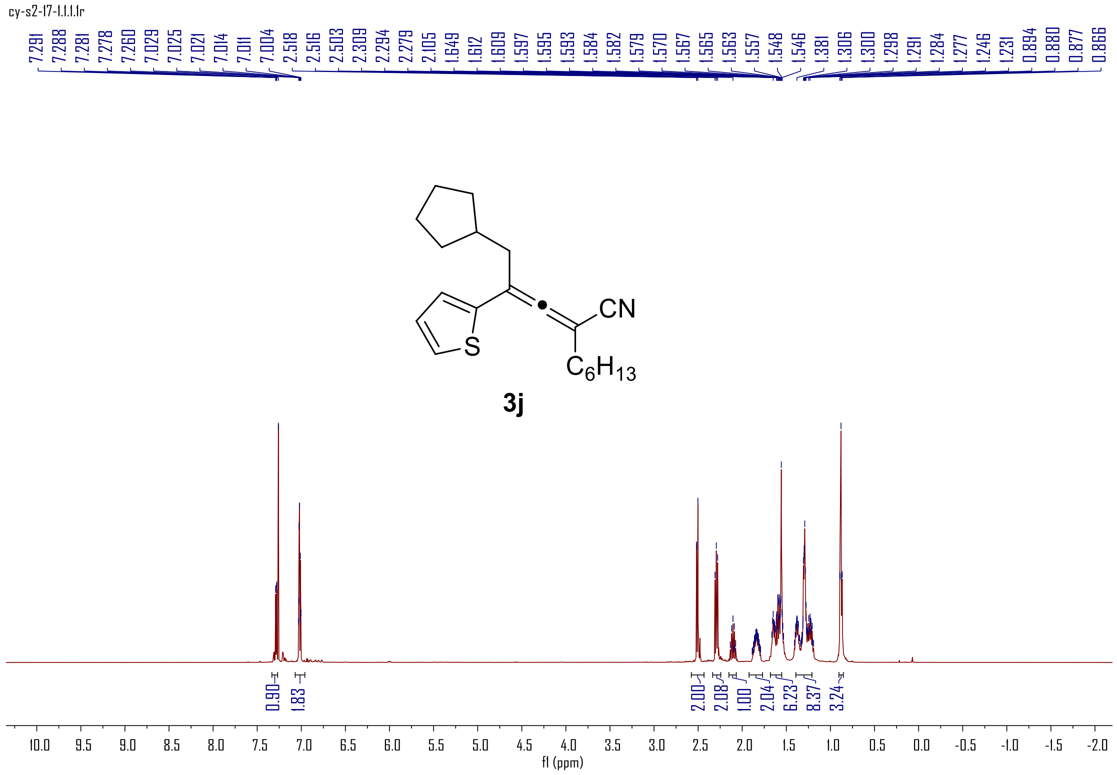
cy-s2-17-1.1.1.1r
7.291 7.288 7.281 7.278 7.260 7.029 7.025 7.021 7.014 7.011 7.004 2.518 2.516 2.503 2.309 2.294 2.279 2.105 1.649 1.612 1.609 1.597 1.595 1.593 1.584 1.582 1.579 1.570 1.567 1.565 1.563 1.557 1.548 1.546 1.381 1.306 1.300 1.298 1.291 1.284 1.277 1.246 1.231 0.894 0.880 0.877 0.866
CN
S
C6H13
3j
0.90 1.83 2.00 2.08 1.00 2.04 6.23 8.37 3.24
f1 (ppm)

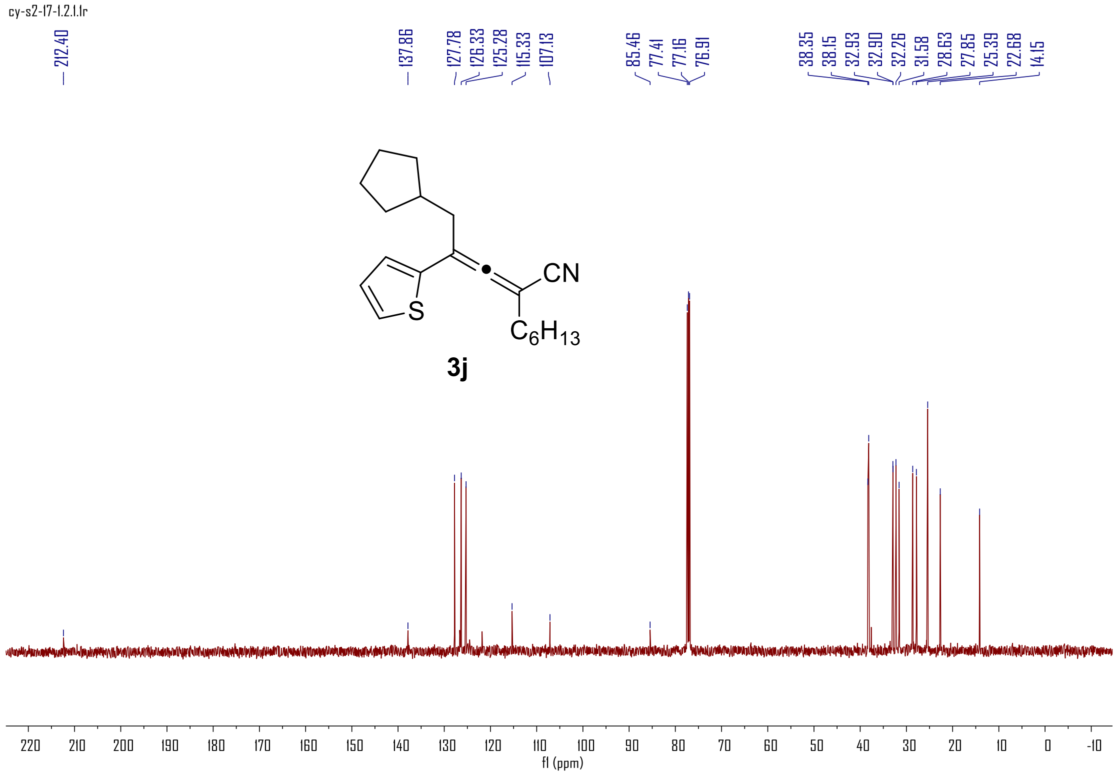
cy-s2-17-1.2.1.1r
212.40 137.86 127.78 126.33 125.28 115.33 107.13 85.46 77.41 77.16 76.91 38.35 38.15 32.93 32.90 32.26 31.58 28.63 27.85 25.39 22.68 14.15
CN
S
C6H13
3j
f1 (ppm)

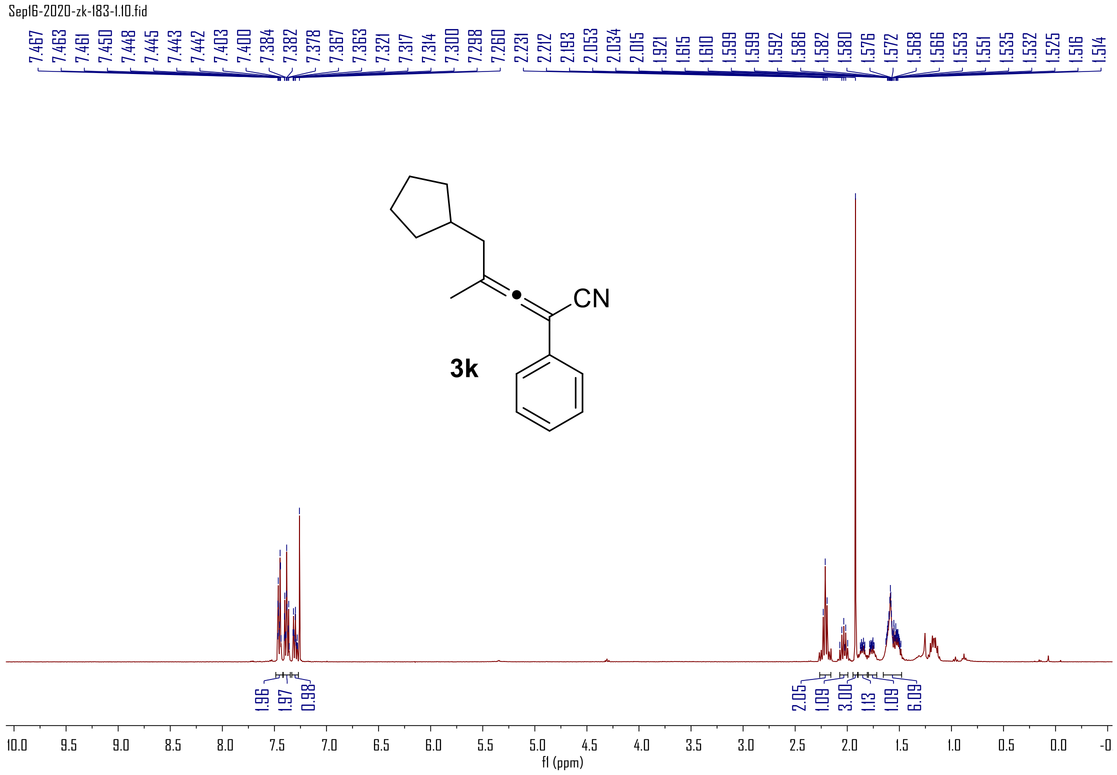
Sep16-2020-zk-183-1.10.fid
7.467
7.463
7.461
7.450
7.448
7.445
7.443
7.442
7.403
7.400
7.384
7.382
7.378
7.367
7.363
7.321
7.317
7.314
7.300
7.298
7.260
2.231
2.212
2.193
2.053
2.034
2.015
1.921
1.615
1.610
1.599
1.599
1.592
1.586
1.582
1.580
1.576
1.572
1.568
1.566
1.553
1.551
1.535
1.532
1.525
1.516
1.514
CN
3k
1.96
1.97
0.98
2.05
1.09
3.00
1.13
1.09
6.09
f1 (ppm)

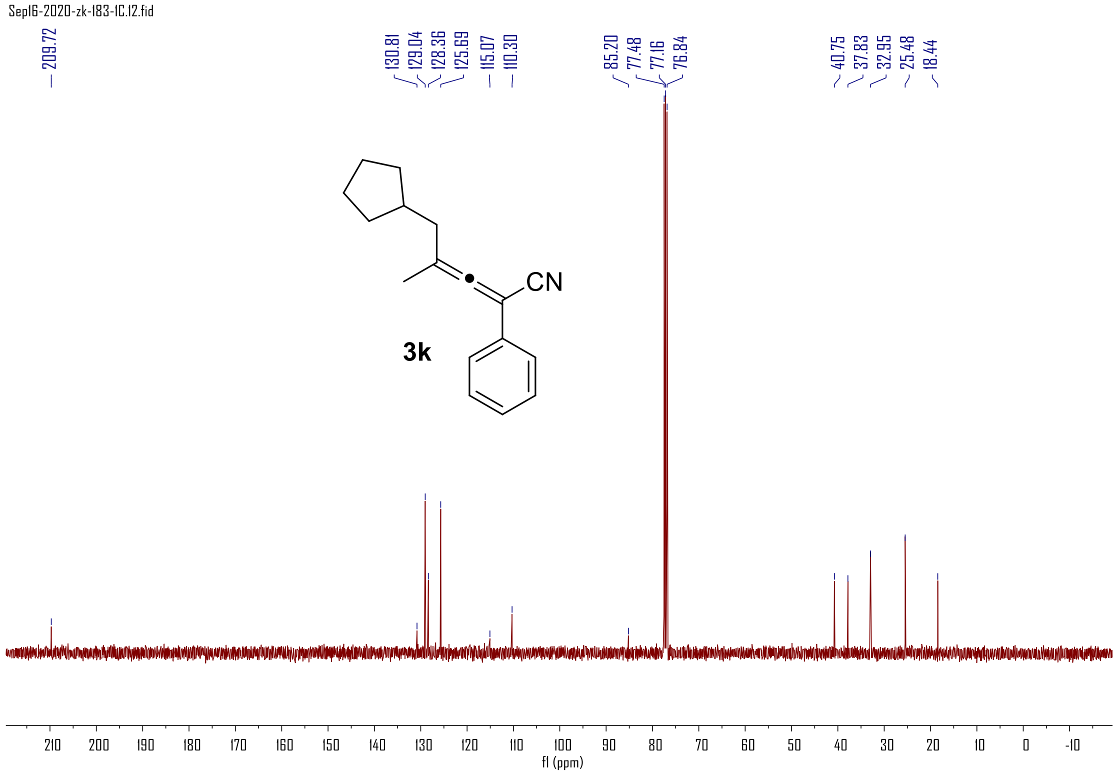
Sep16-2020-zk-183-1C.12.fid
209.72
130.81
129.04
128.36
125.69
115.07
110.30
85.20
77.48
77.16
76.84
40.75
37.83
32.95
25.48
18.44
CN
3k
f1 (ppm)

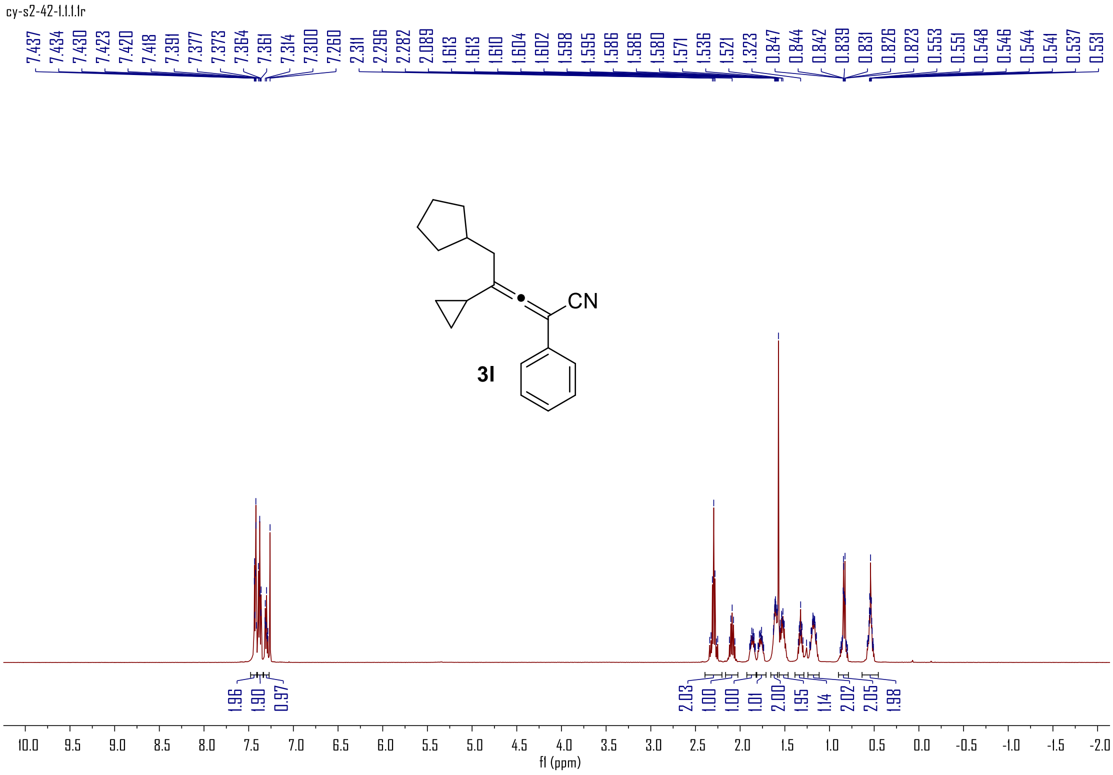
cy-s2-42-1.1.1.1r
7.437 7.434 7.430 7.423 7.420 7.418 7.391 7.377 7.373 7.364 7.361 7.314 7.300 7.260 2.311 2.296 2.282 2.089 1.613 1.613 1.610 1.604 1.602 1.598 1.595 1.586 1.580 1.571 1.536 1.521 1.323 0.847 0.844 0.842 0.839 0.831 0.826 0.823 0.553 0.551 0.548 0.546 0.544 0.541 0.537 0.531
CN
3l
1.96 1.90 0.97 2.03 1.00 1.00 1.01 2.00 1.95 1.14 2.02 2.05 1.98
10.0 9.5 9.0 8.5 8.0 7.5 7.0 6.5 6.0 5.5 5.0 4.5 4.0 3.5 3.0 2.5 2.0 1.5 1.0 0.5 0.0 -0.5 -1.0 -1.5 -2.0
f1 (ppm)

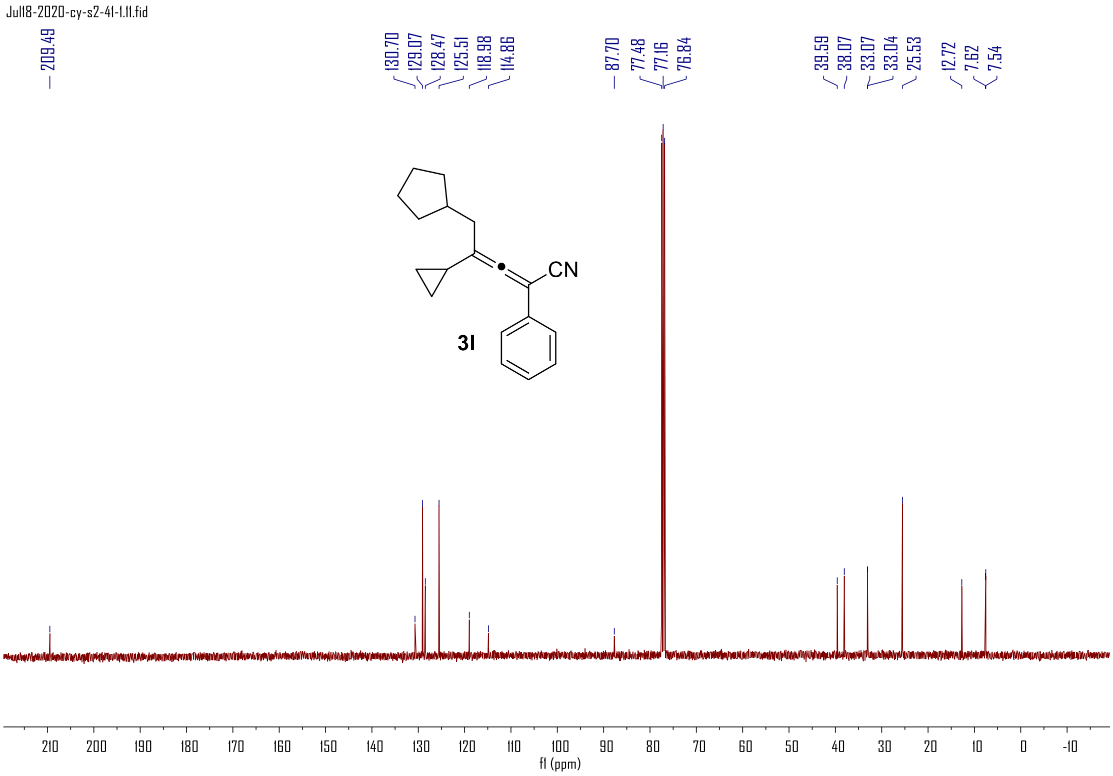
Jul18-2020-cy-s2-41-1.11.fid
209.49
130.70 129.07 128.47 125.51 118.98 114.86
87.70 77.48 77.16 76.84
39.59 38.07 33.07 33.04 25.53
12.72 7.62 7.54
CN
3l
210 200 190 180 170 160 150 140 130 120 110 100 90 80 70 60 50 40 30 20 10 0 -10
f1 (ppm)

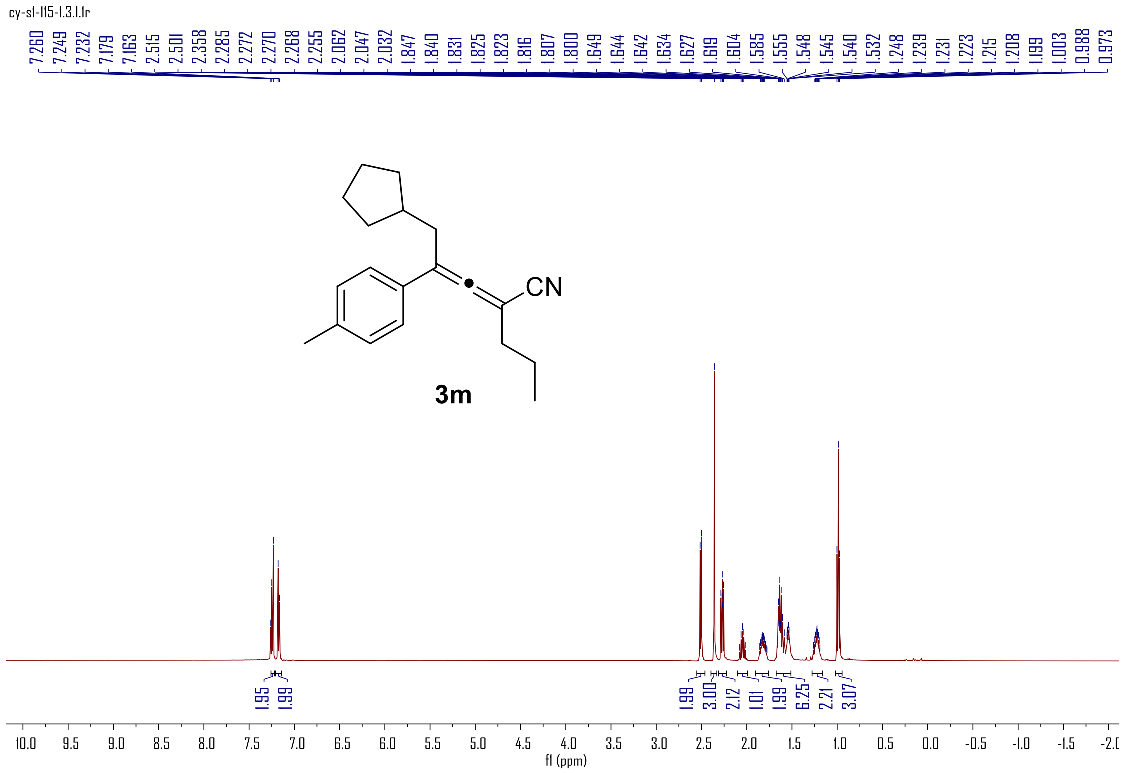
cy-sl-115-1.3.1.1r
7.260
7.249
7.232
7.179
7.163
2.515
2.501
2.358
2.285
2.272
2.270
2.268
2.255
2.062
2.047
2.032
1.847
1.840
1.831
1.825
1.823
1.816
1.807
1.800
1.649
1.644
1.642
1.634
1.627
1.619
1.604
1.585
1.555
1.548
1.545
1.540
1.532
1.248
1.239
1.231
1.223
1.215
1.208
1.199
1.003
0.988
0.973
CN
3m
1.95
1.99
1.99
3.00
2.12
1.01
1.99
6.25
2.21
3.07
f1 (ppm)

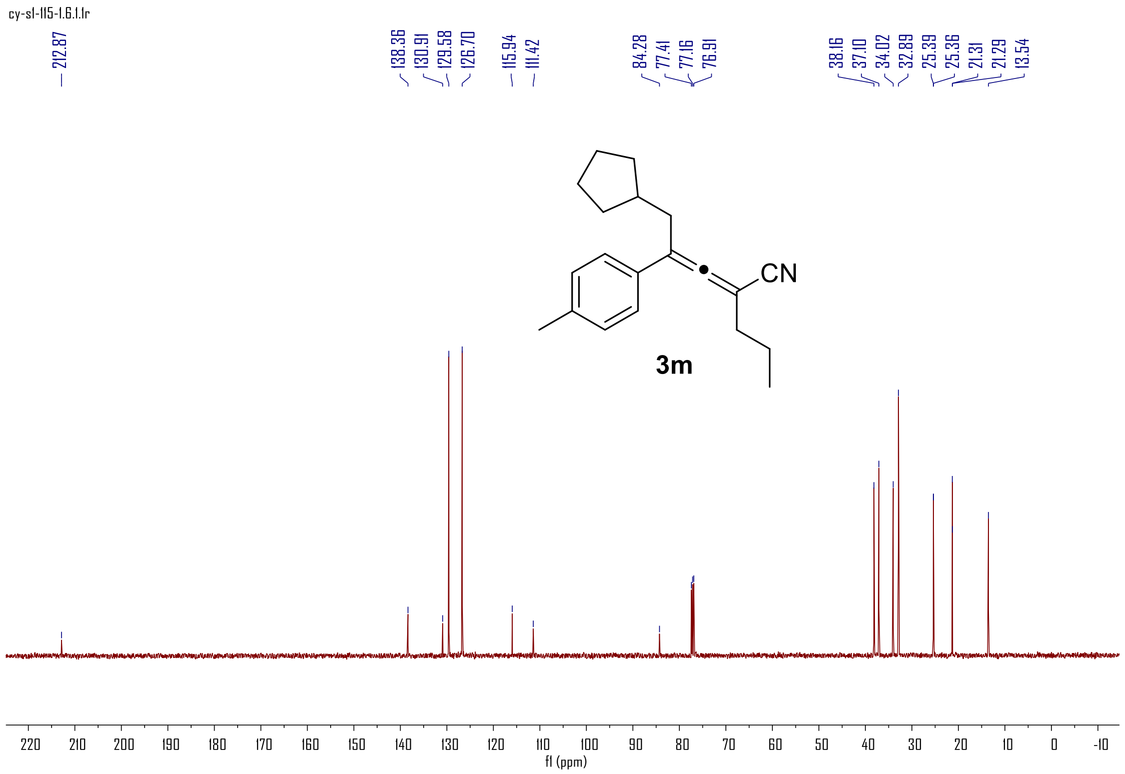
cy-sl-115-1.6.1.1r
212.87
138.36
130.91
129.58
126.70
115.94
111.42
84.28
77.41
77.16
76.91
38.16
37.10
34.02
32.89
25.39
25.36
21.31
21.29
13.54
CN
3m
f1 (ppm)

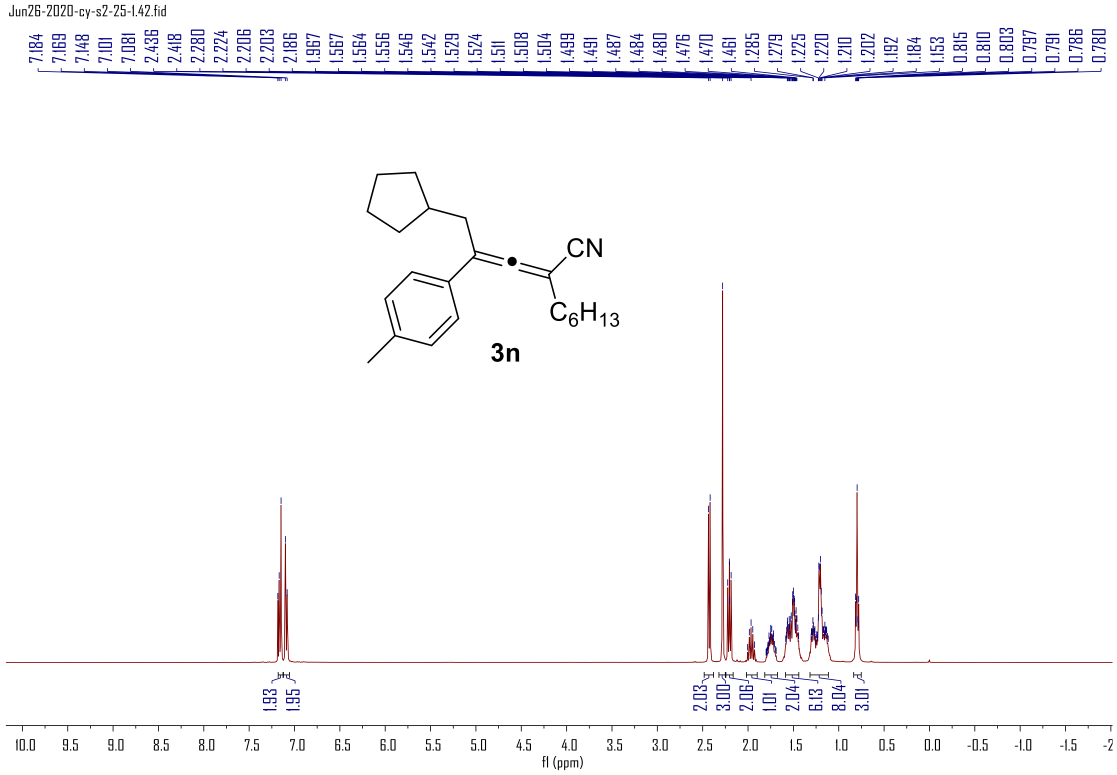
Jun26-2020-cy-s2-25-1.42.fid
7.184 7.169 7.148 7.101 7.081 2.436 2.418 2.280 2.224 2.206 2.203 2.186 1.967 1.567 1.564 1.556 1.546 1.542 1.529 1.524 1.511 1.508 1.504 1.499 1.491 1.487 1.484 1.480 1.476 1.470 1.461 1.285 1.279 1.225 1.220 1.210 1.202 1.192 1.184 1.153 0.815 0.810 0.803 0.797 0.791 0.786 0.780
CN
C6H13
3n
1.93 1.95 2.03 3.00 2.06 1.01 2.04 6.13 8.04 3.01
f1 (ppm)

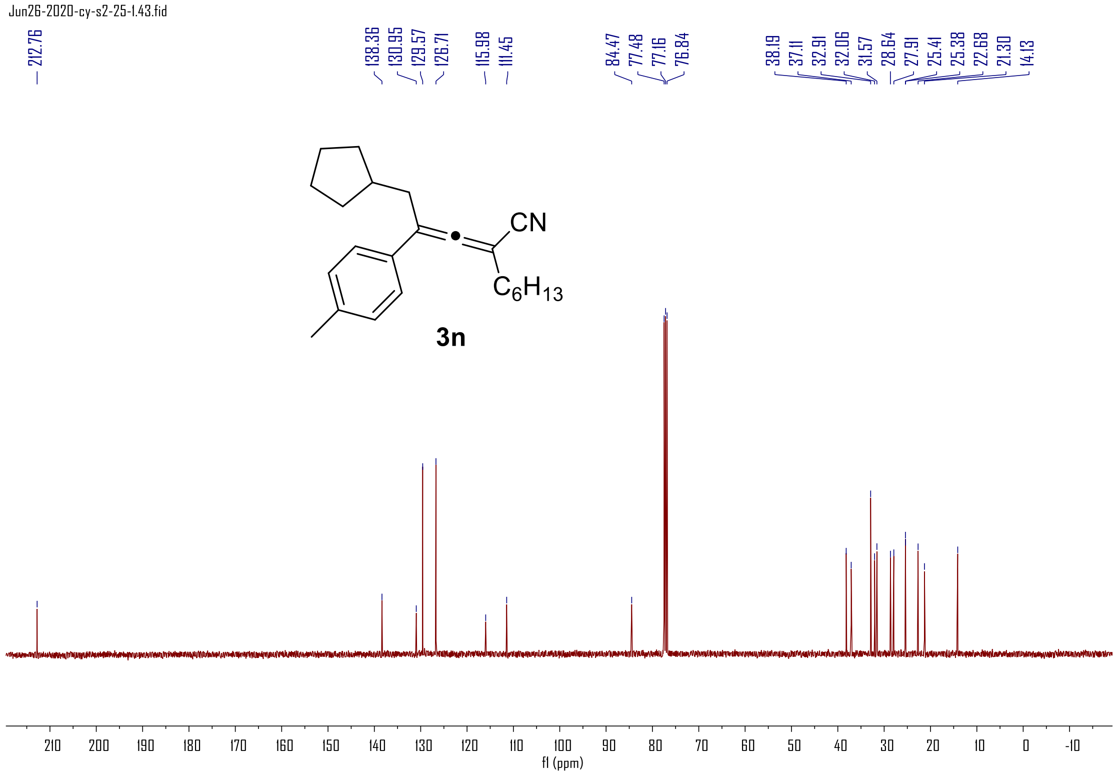
Jun26-2020-cy-s2-25-1.43.fid
212.76 138.36 130.95 129.57 126.71 115.98 111.45 84.47 77.48 77.16 76.84 38.19 37.11 32.91 32.06 31.57 28.64 27.91 25.41 25.38 22.68 21.30 14.13
CN
C6H13
3n
f1 (ppm)

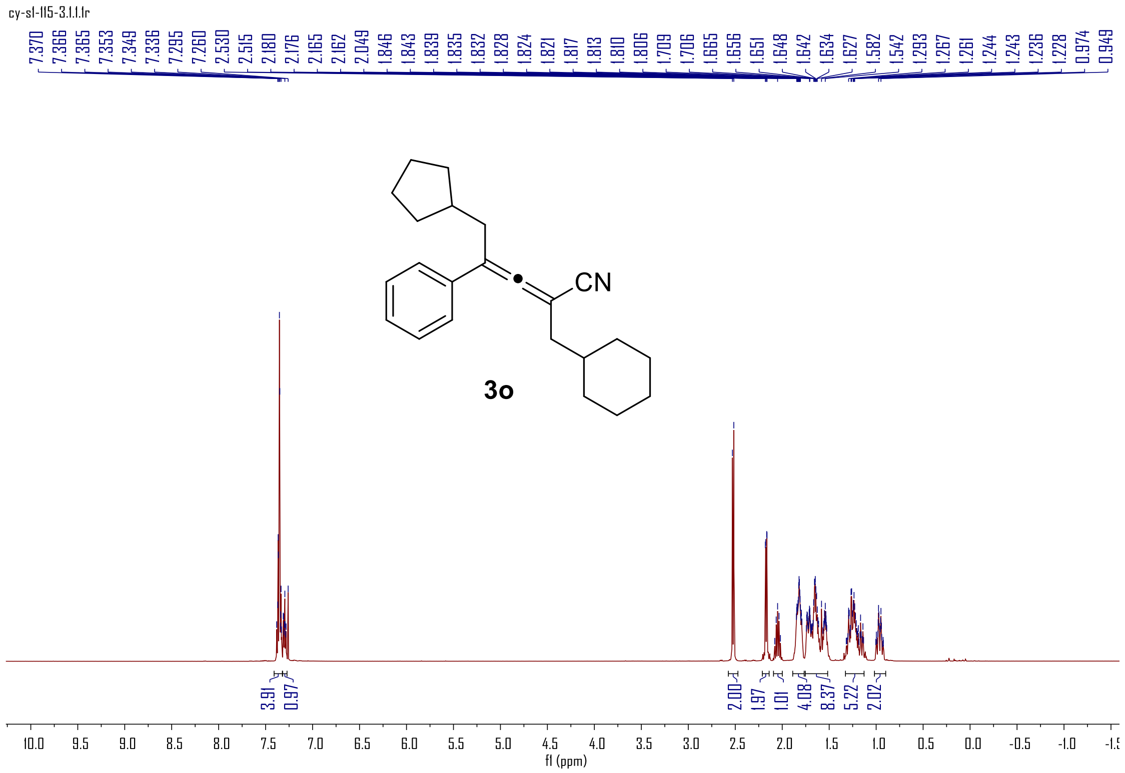
cy-sl-115-3.1.1.fr
7.370 7.366 7.365 7.353 7.349 7.336 7.295 7.260 2.530 2.515 2.180 2.176 2.165 2.162 2.049 1.846 1.843 1.839 1.835 1.832 1.828 1.824 1.821 1.817 1.813 1.810 1.806 1.709 1.706 1.665 1.656 1.651 1.648 1.642 1.634 1.627 1.582 1.542 1.293 1.267 1.261 1.244 1.243 1.236 1.228 0.974 0.949
CN
3o
3.91 0.97 2.00 1.97 1.01 4.08 8.37 5.22 2.02
f1 (ppm)

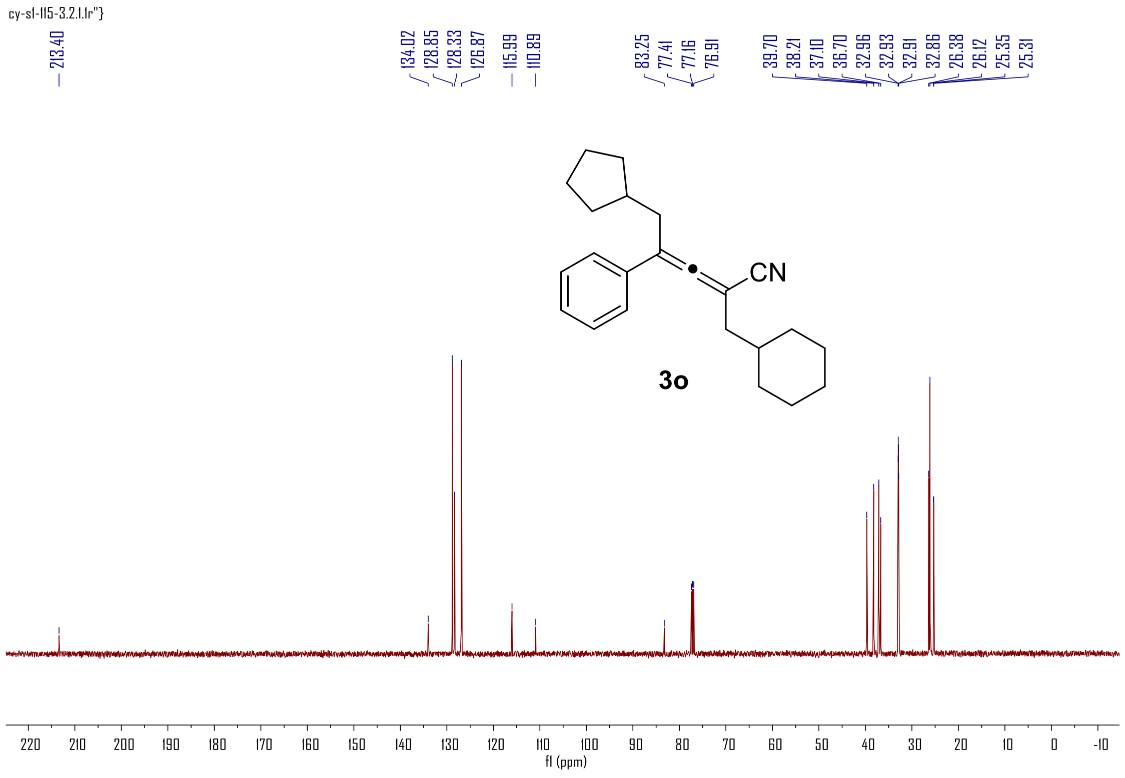
cy-sl-115-3.2.1.fr"}
213.40
134.02 128.85 128.33 126.87 115.99 110.89
83.25 77.41 77.16 76.91
39.70 38.21 37.10 36.70 32.96 32.93 32.91 32.86 26.38 26.12 25.35 25.31
CN
3o
f1 (ppm)

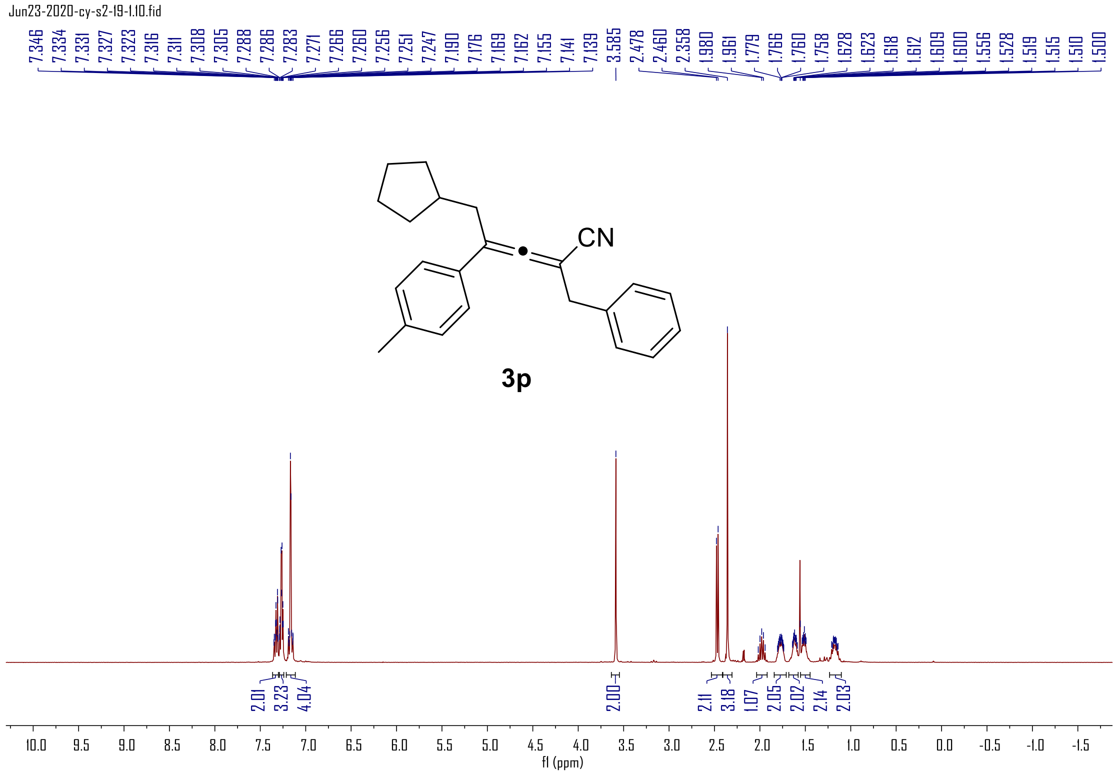
Jun23-2020-cy-s2-19-1.10.fid
7.346 7.334 7.331 7.327 7.323 7.316 7.311 7.308 7.305 7.288 7.286 7.283 7.271 7.266 7.260 7.256 7.251 7.247 7.190 7.176 7.169 7.162 7.155 7.141 7.139 3.585 2.478 2.460 2.358 1.980 1.961 1.779 1.766 1.760 1.758 1.628 1.623 1.618 1.612 1.609 1.600 1.556 1.528 1.519 1.515 1.510 1.500
CN
3p
2.01 3.23 4.04 2.00 2.11 3.18 1.07 2.05 2.02 2.14 2.03
10.0 9.5 9.0 8.5 8.0 7.5 7.0 6.5 6.0 5.5 5.0 4.5 4.0 3.5 3.0 2.5 2.0 1.5 1.0 0.5 0.0 -0.5 -1.0 -1.5
f1 (ppm)

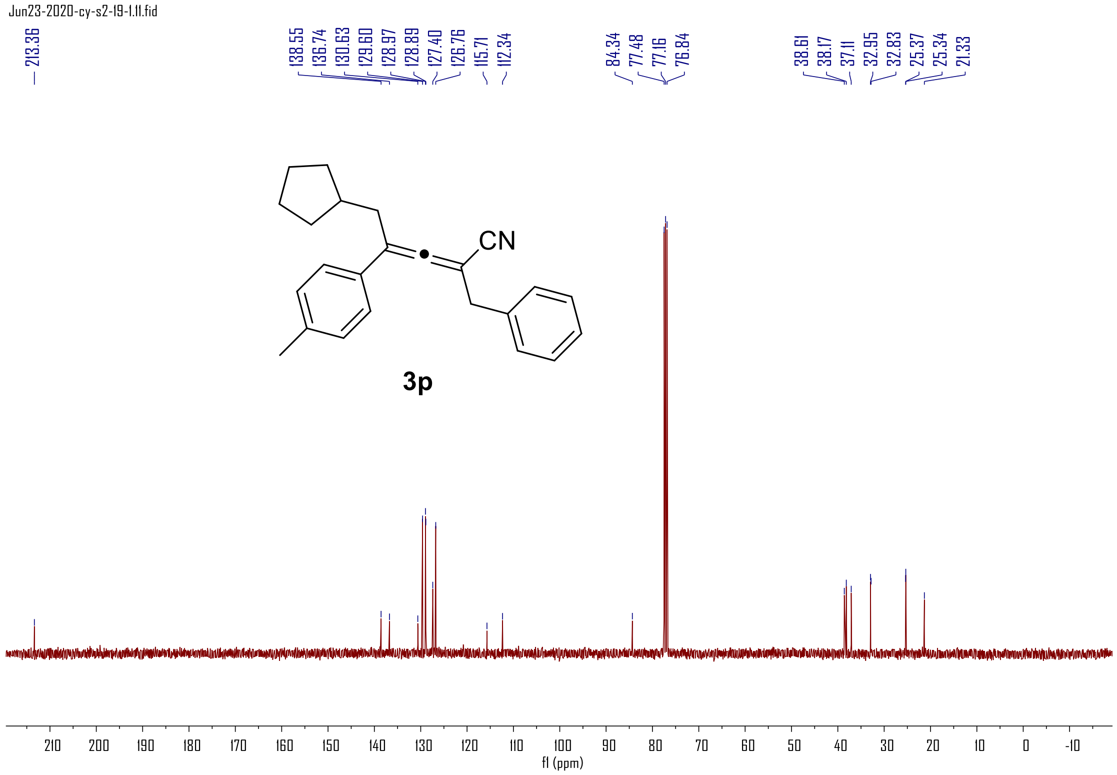
Jun23-2020-cy-s2-19-1.11.fid
213.36 138.55 136.74 130.63 129.60 128.97 128.89 127.40 126.76 115.71 112.34 84.34 77.48 77.16 76.84 38.61 38.17 37.11 32.95 32.83 25.37 25.34 21.33
CN
3p
210 200 190 180 170 160 150 140 130 120 110 100 90 80 70 60 50 40 30 20 10 0 -10
f1 (ppm)

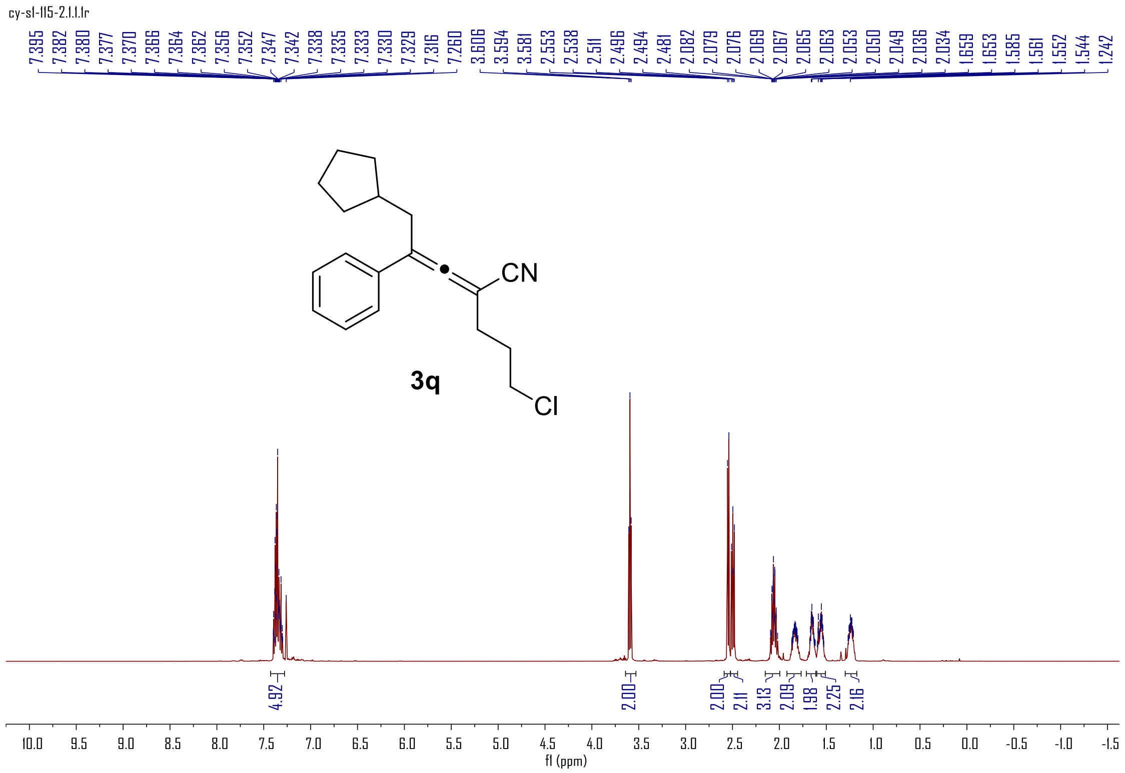
cy-sl-115-2.1.1.fr
7.395 7.382 7.380 7.377 7.370 7.366 7.364 7.362 7.356 7.352 7.347 7.342 7.338 7.335 7.333 7.330 7.329 7.316 7.260 3.606 3.594 3.581 2.553 2.538 2.511 2.496 2.494 2.481 2.082 2.079 2.076 2.069 2.067 2.065 2.063 2.053 2.050 2.049 2.036 2.034 1.659 1.653 1.585 1.561 1.552 1.544 1.242
CN
Cl
3q
4.92
2.00
2.00
2.11
3.13
2.09
1.98
2.25
2.16
10.0 9.5 9.0 8.5 8.0 7.5 7.0 6.5 6.0 5.5 5.0 4.5 4.0 3.5 3.0 2.5 2.0 1.5 1.0 0.5 0.0 -0.5 -1.0 -1.5
f1 (ppm)

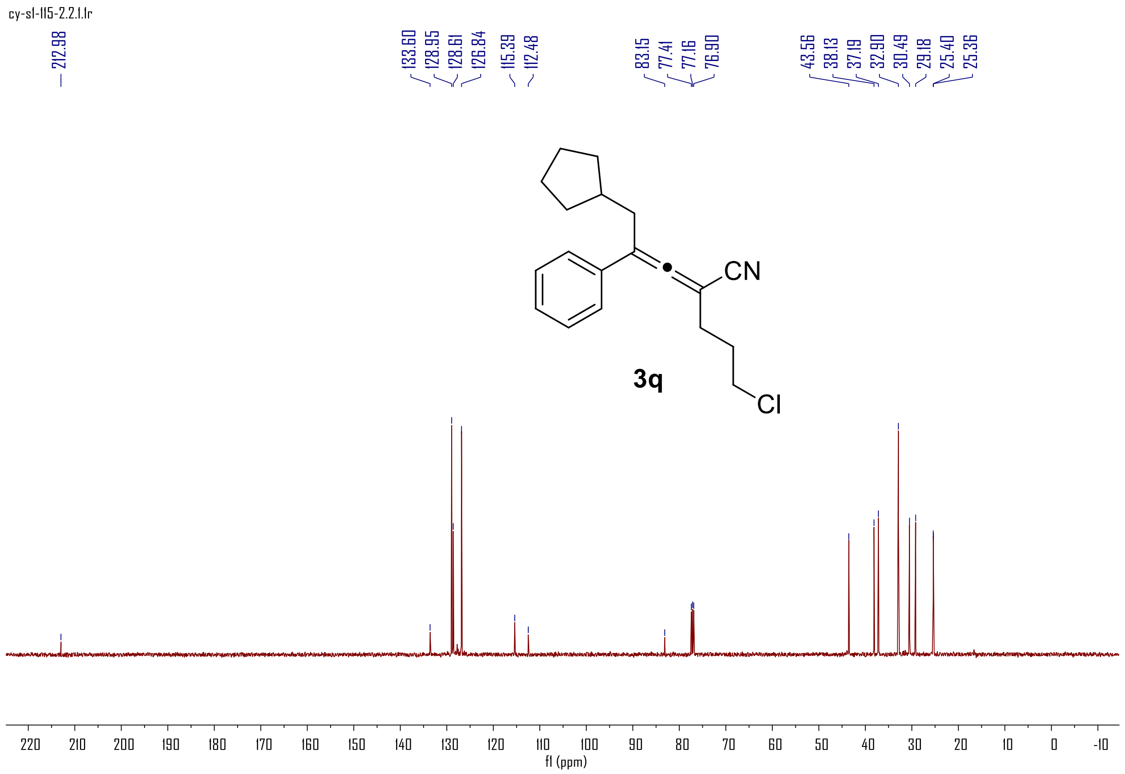
cy-sl-115-2.2.1.fr
212.98
133.60 128.95 128.61 126.84 115.39 112.48
83.15 77.41 77.16 76.90
43.56 38.13 37.19 32.90 30.49 29.18 25.40 25.36
CN
Cl
3q
220 210 200 190 180 170 160 150 140 130 120 110 100 90 80 70 60 50 40 30 20 10 0 -10
f1 (ppm)

Mar10-2020-cy-sl-112-2.22.fid

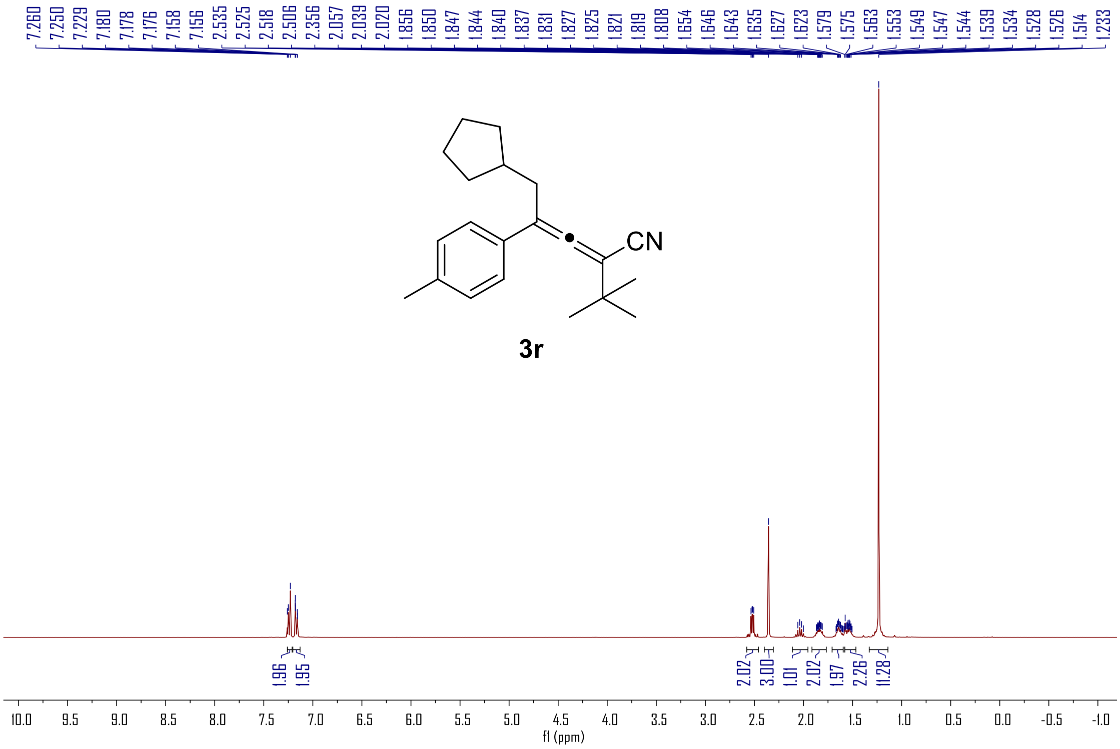

Mar10-2020-cy-sl-112-2.23.fid

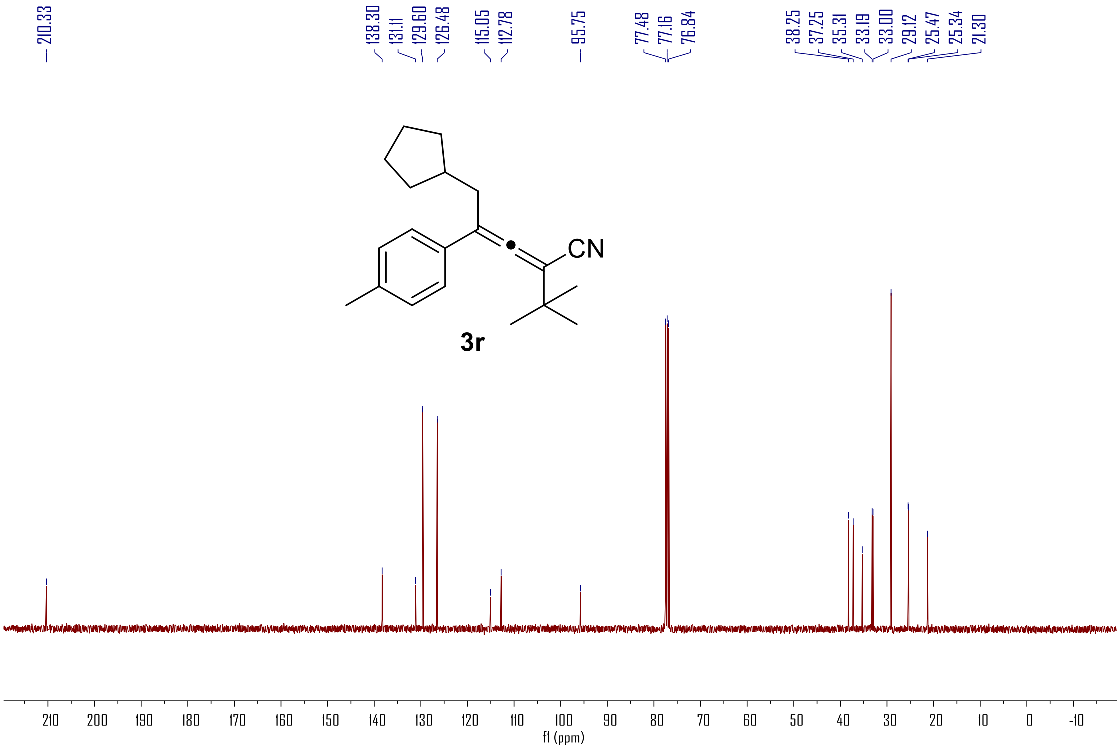

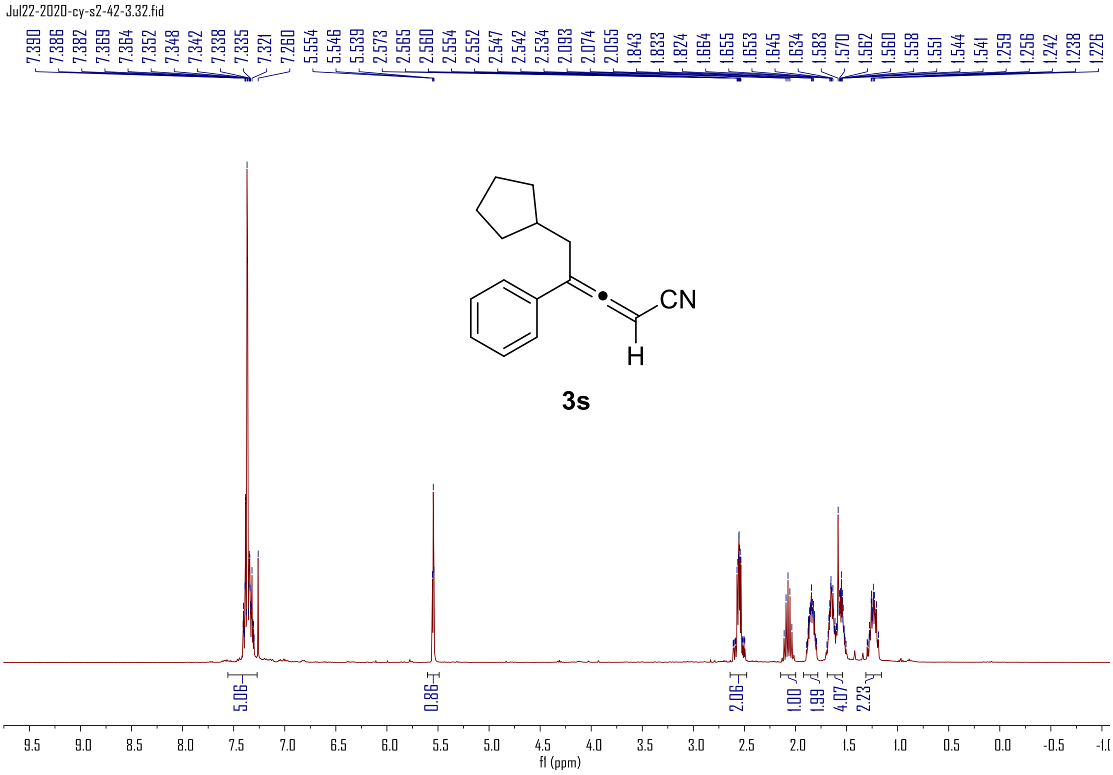
Jul22-2020-cy-s2-42-3.32.fid
7.390 7.386 7.382 7.369 7.364 7.352 7.348 7.342 7.338 7.335 7.321 7.260 5.554 5.546 5.539 2.573 2.565 2.560 2.554 2.552 2.547 2.542 2.534 2.093 2.074 2.055 1.843 1.833 1.824 1.664 1.655 1.653 1.645 1.634 1.583 1.570 1.562 1.560 1.558 1.551 1.544 1.541 1.259 1.256 1.242 1.238 1.226
CN
H
3s
5.06 0.86 2.06 1.00 1.99 4.07 2.23
9.5 9.0 8.5 8.0 7.5 7.0 6.5 6.0 5.5 5.0 4.5 4.0 3.5 3.0 2.5 2.0 1.5 1.0 0.5 0.0 -0.5
f1 (ppm)

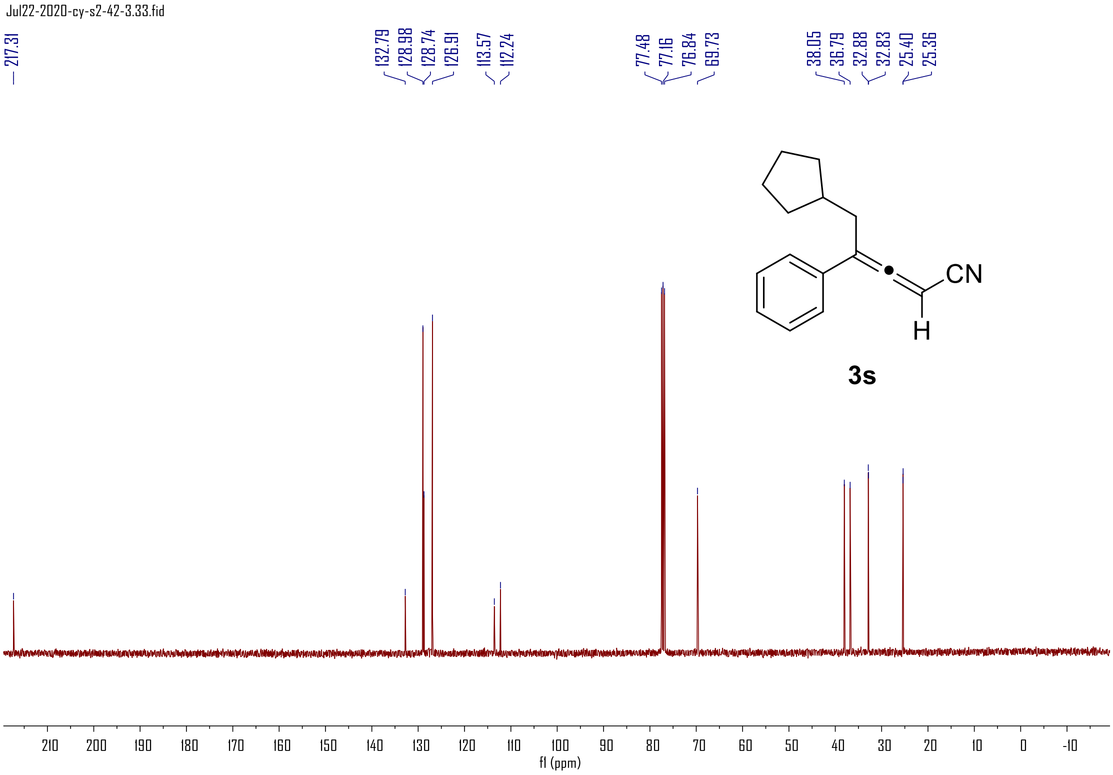
Jul22-2020-cy-s2-42-3.33.fid
217.31
132.79 128.98 128.74 126.91 113.57 112.24
77.48 77.16 76.84 69.73
38.05 36.79 32.88 32.83 25.40 25.36
CN
H
3s
210 200 190 180 170 160 150 140 130 120 110 100 90 80 70 60 50 40 30 20 10 0 -10
f1 (ppm)

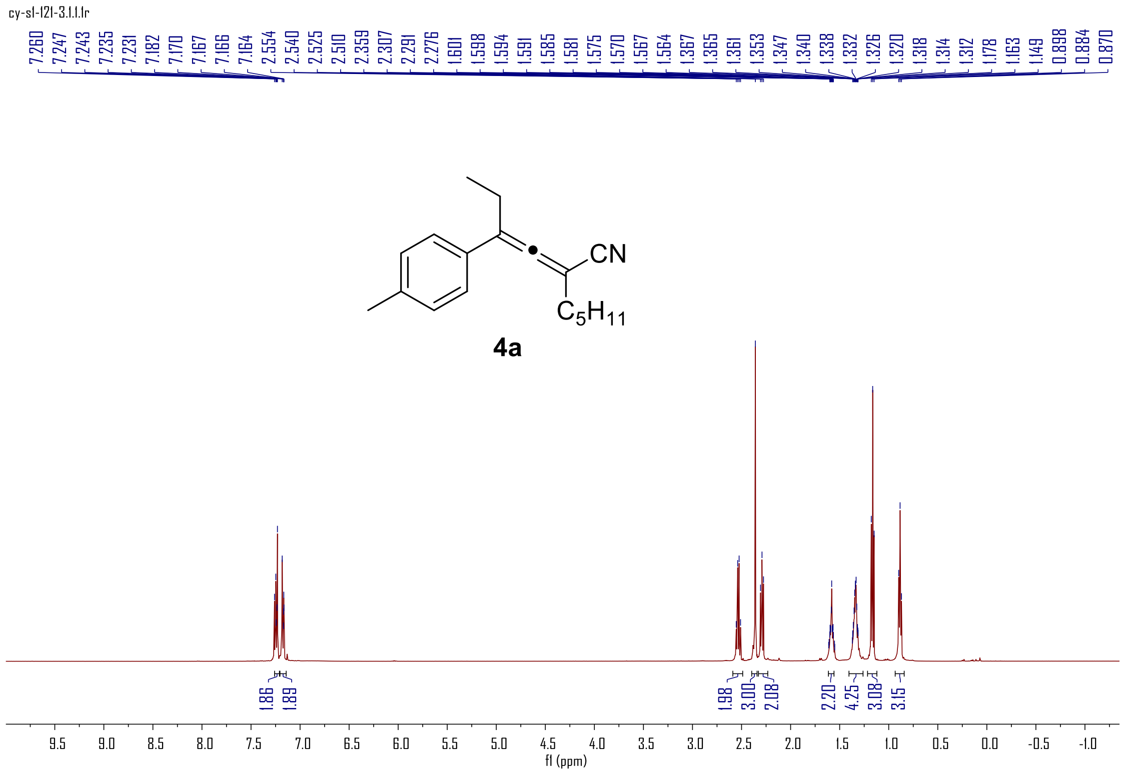
cy-sl-121-3.1.1.1r
7.260 7.247 7.243 7.235 7.231 7.182 7.170 7.167 7.166 7.164 2.554 2.540 2.525 2.510 2.359 2.307 2.291 2.276 1.601 1.598 1.594 1.591 1.585 1.581 1.575 1.570 1.567 1.564 1.367 1.365 1.361 1.353 1.347 1.340 1.338 1.332 1.326 1.320 1.318 1.314 1.312 1.178 1.163 1.149 0.898 0.884 0.870
CN
C_5H_{11}
4a
1.86 1.89 1.98 3.00 2.08 2.20 4.25 3.08 3.15
f1 (ppm)

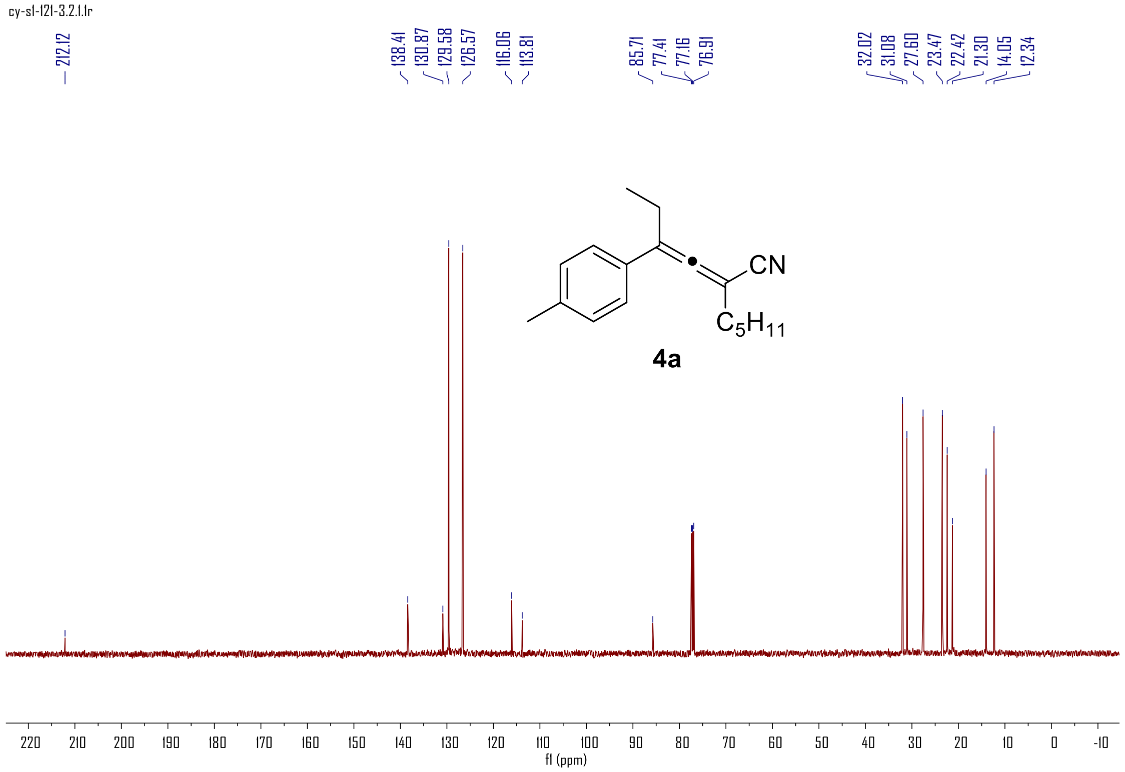
cy-sl-121-3.2.1.1r
212.12
138.41 130.87 129.58 126.57 116.06 113.81
85.71 77.41 77.16 76.91
32.02 31.08 27.60 23.47 22.42 21.30 14.05 12.34
CN
C_5H_{11}
4a
f1 (ppm)

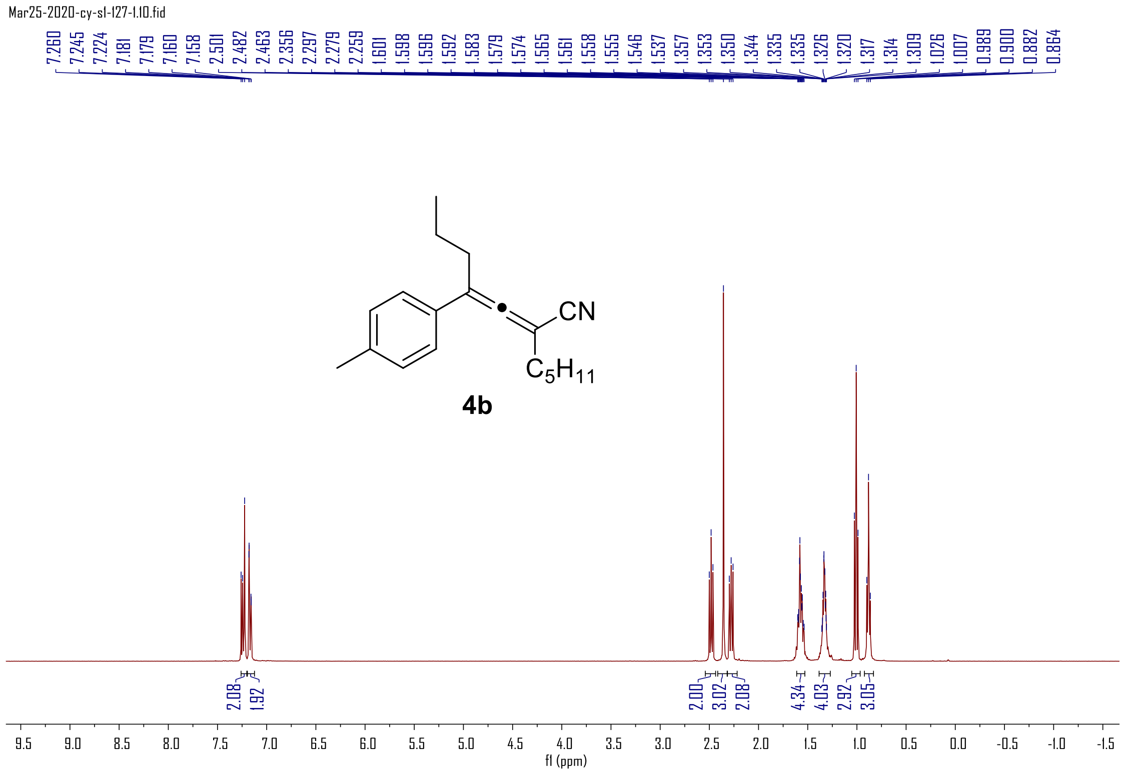
Mar25-2020-cy-sl-127-1.10.fid
7.260 7.245 7.224 7.181 7.179 7.160 7.158 2.501 2.482 2.463 2.356 2.297 2.279 2.259 1.601 1.598 1.596 1.592 1.583 1.579 1.574 1.565 1.561 1.558 1.555 1.546 1.537 1.357 1.353 1.350 1.344 1.335 1.335 1.326 1.320 1.317 1.314 1.309 1.026 1.007 0.989 0.900 0.882 0.864
CN
C5H11
4b
2.08 1.92 2.00 3.02 2.08 4.34 4.03 2.92 3.05
9.5 9.0 8.5 8.0 7.5 7.0 6.5 6.0 5.5 5.0 4.5 4.0 3.5 3.0 2.5 2.0 1.5 1.0 0.5 0.0 -0.5 -1.0 -1.5
f1 (ppm)

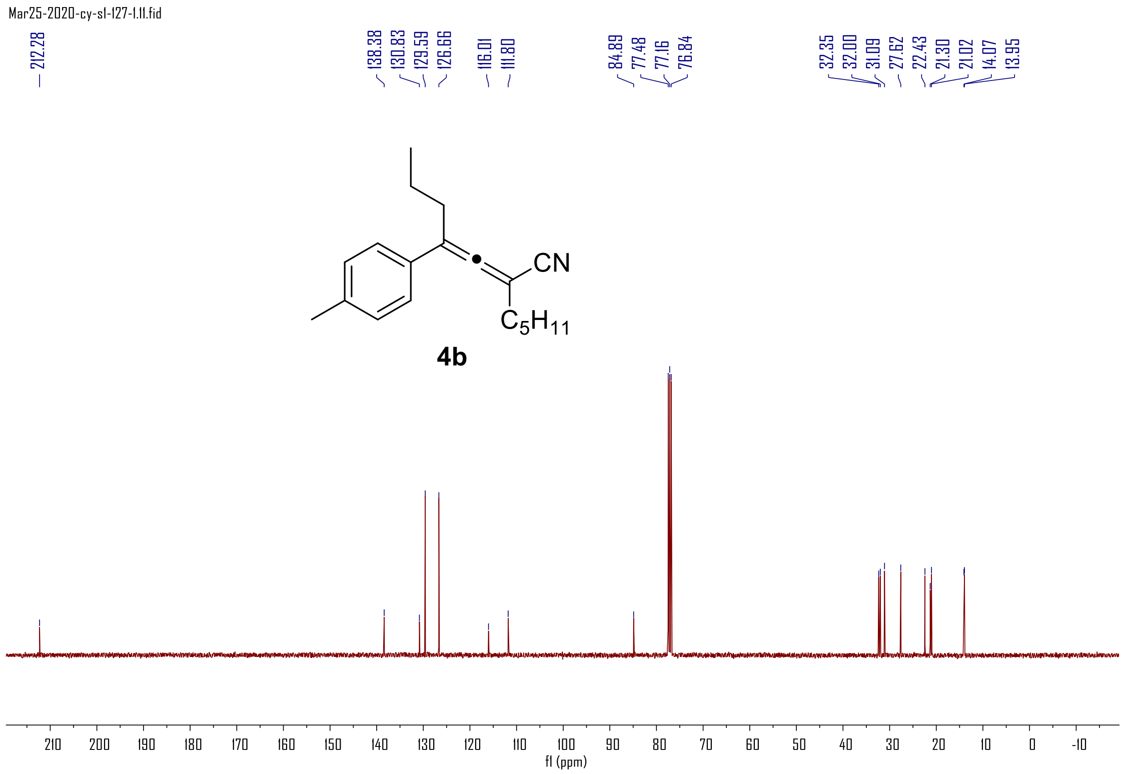
Mar25-2020-cy-sl-127-1.11.fid
212.28
138.38 130.83 129.59 126.66 116.01 111.80
84.89 77.48 77.16 76.84
32.35 32.00 31.09 27.62 22.43 21.30 21.02 14.07 13.95
CN
C5H11
4b
210 200 190 180 170 160 150 140 130 120 110 100 90 80 70 60 50 40 30 20 10 0 -10
f1 (ppm)

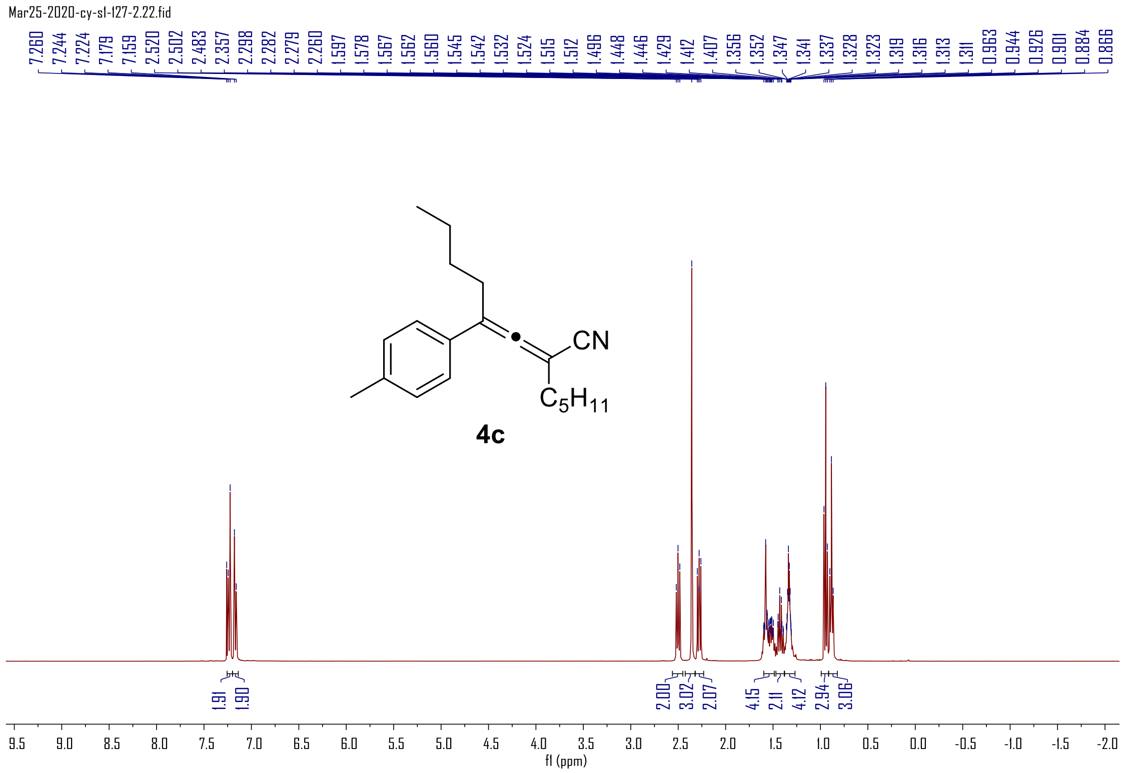
Mar25-2020-cy-sl-127-2.22.fid
4c
CN
C_5H_{11}
fl (ppm)

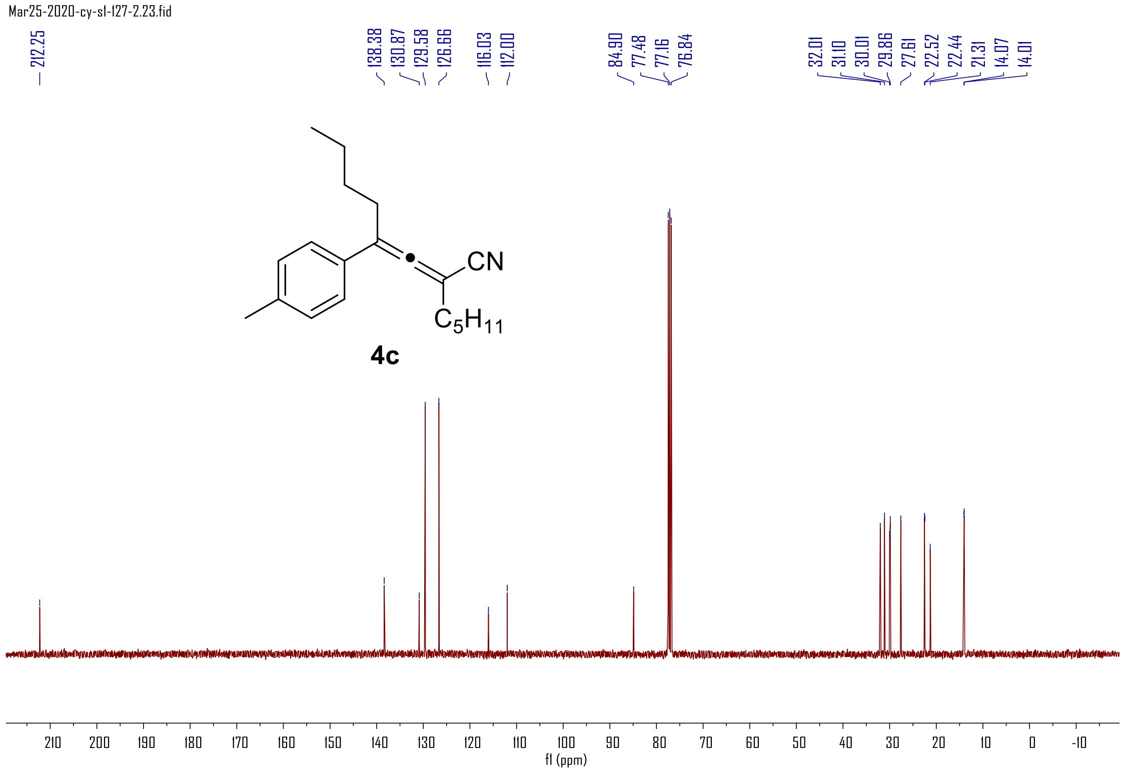
Mar25-2020-cy-sl-127-2.23.fid
4c
CN
C_5H_{11}
fl (ppm)

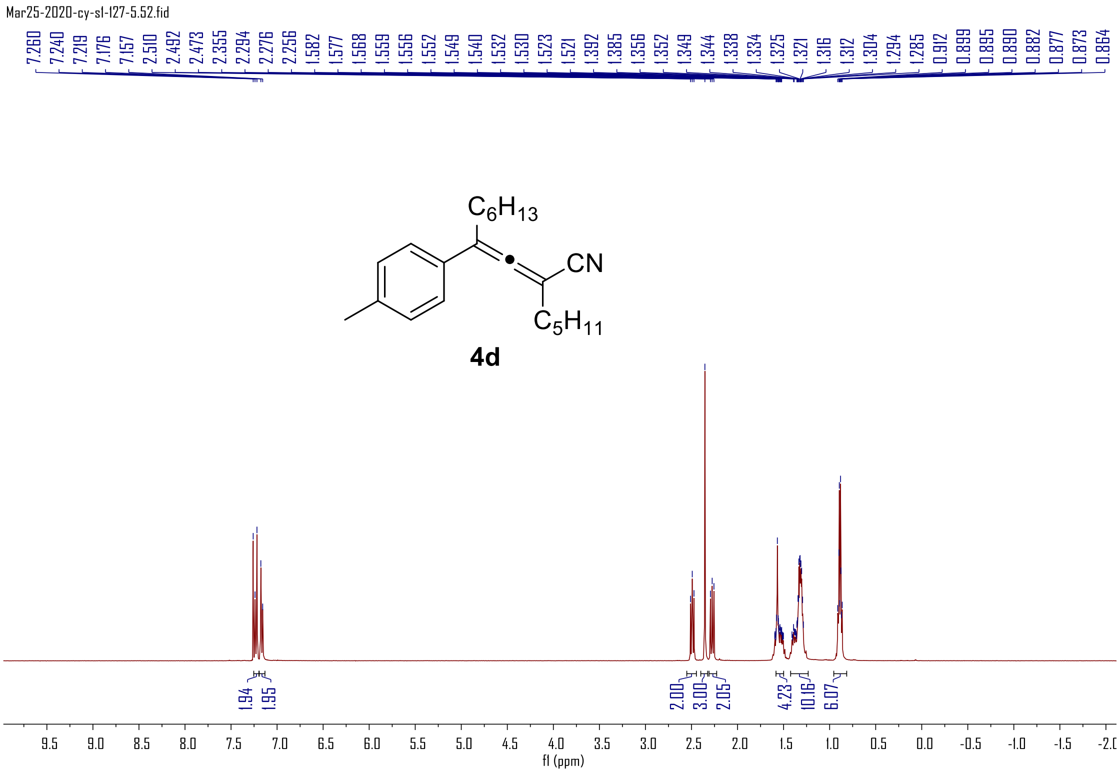
Mar25-2020-cy-sl-127-5.52.fid
7.260 7.240 7.219 7.176 7.157 2.510 2.492 2.473 2.355 2.294 2.276 2.256 1.582 1.577 1.568 1.559 1.556 1.552 1.549 1.540 1.532 1.530 1.523 1.521 1.392 1.385 1.356 1.352 1.349 1.344 1.338 1.334 1.325 1.321 1.316 1.312 1.304 1.294 1.285 0.912 0.899 0.895 0.890 0.882 0.877 0.873 0.864
C6H13
CN
C5H11
4d
1.94 1.95 2.00 3.00 2.05 4.23 10.16 6.07
f1 (ppm)

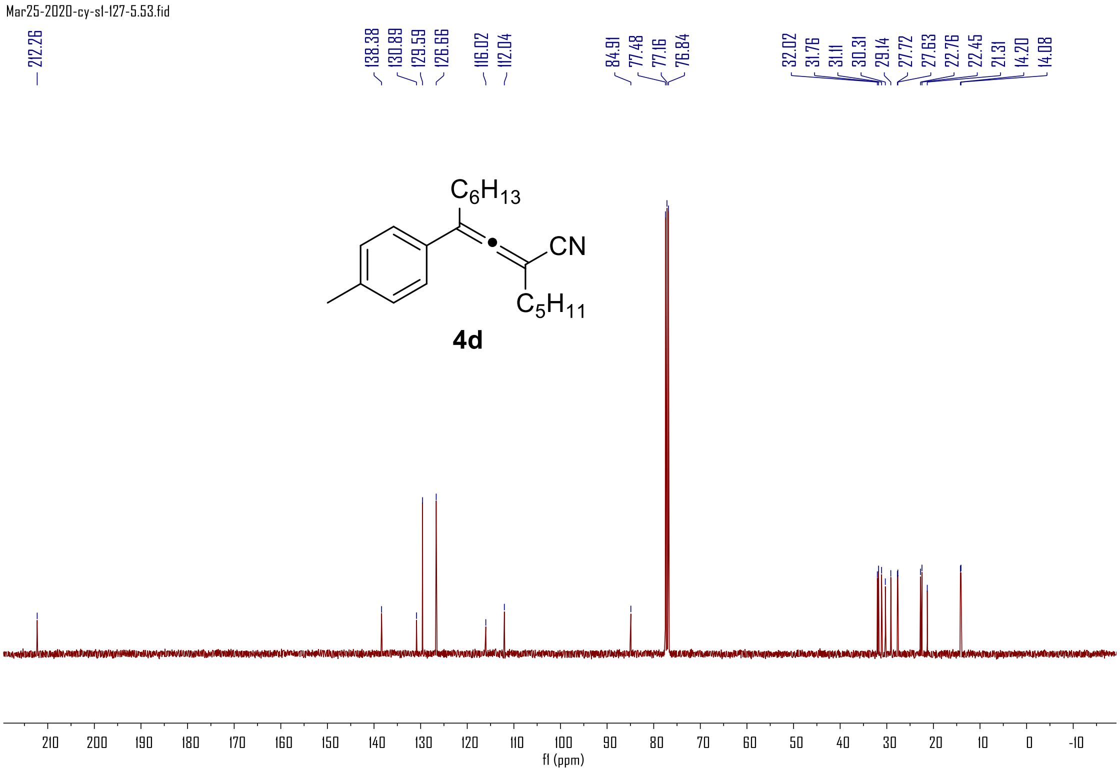
Mar25-2020-cy-sl-127-5.53.fid
212.26
138.38 130.89 129.59 126.66 116.02 112.04
84.91 77.48 77.16 76.84
32.02 31.76 31.11 30.31 29.14 27.72 27.63 22.76 22.45 21.31 14.20 14.08
C6H13
CN
C5H11
4d
f1 (ppm)

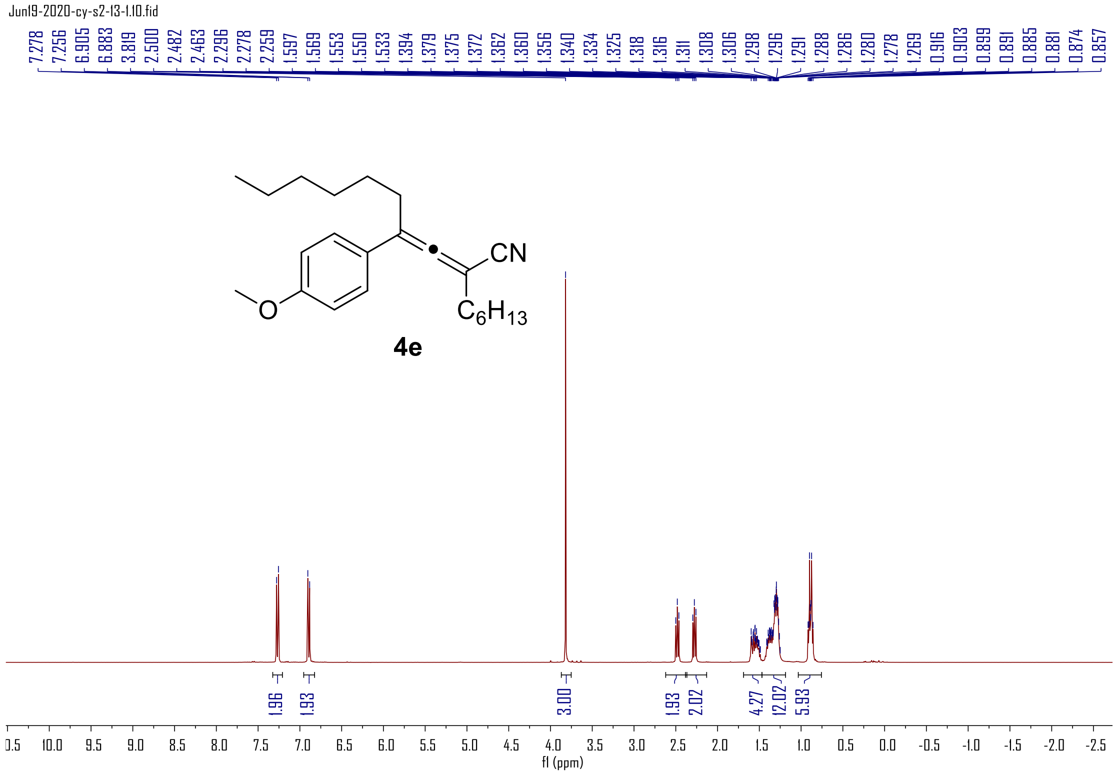
Jun19-2020-cy-s2-13-1.10.fid
CN
C6H13
4e
1.96
1.93
3.00
1.93
2.02
4.27
12.02
5.93
f1 (ppm)

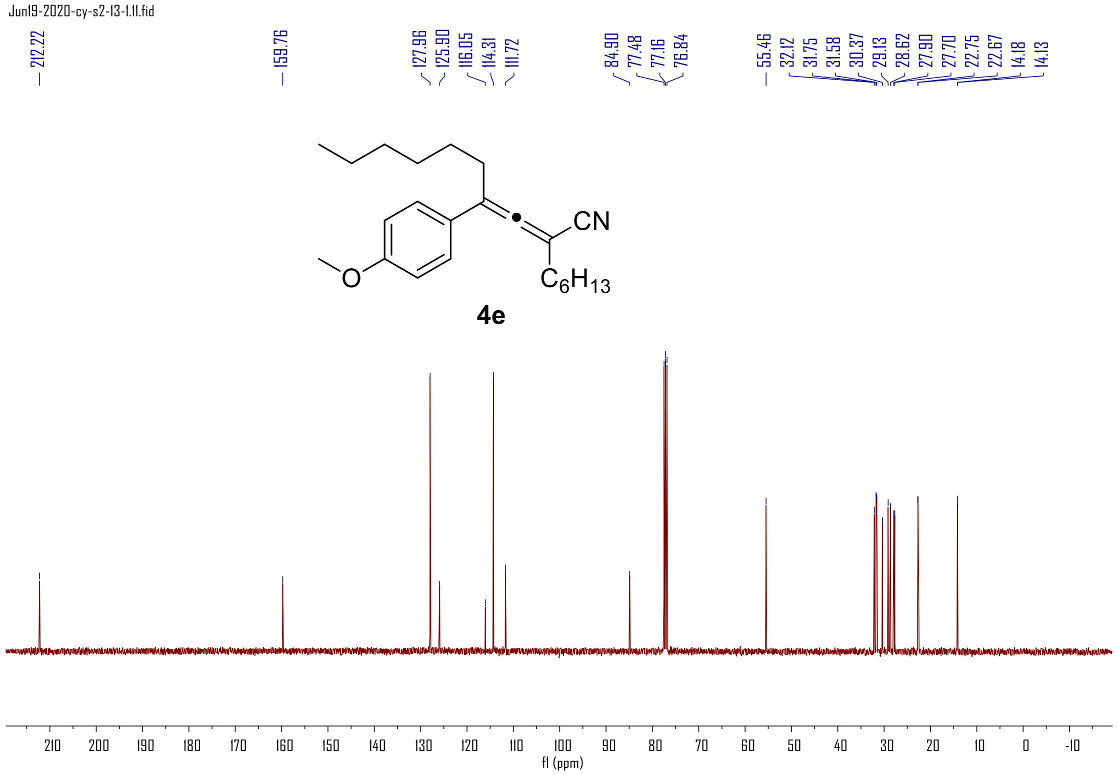
Jun19-2020-cy-s2-13-1.11.fid
212.22
159.76
127.96
125.90
116.05
114.31
111.72
84.90
77.48
77.16
76.84
55.46
32.12
31.75
31.58
30.37
29.13
28.62
27.90
27.70
22.75
22.67
14.18
14.13
CN
C6H13
4e
f1 (ppm)

Mar25-2020-cy-sl-121-4.32.fid

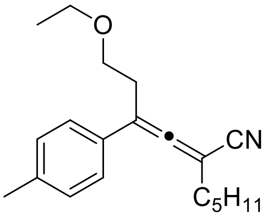

4f

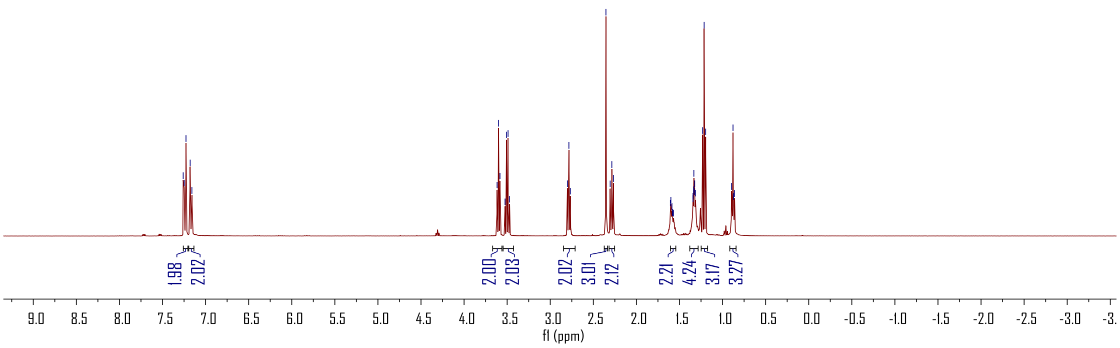

Mar25-2020-cy-sl-121-4.33.fid

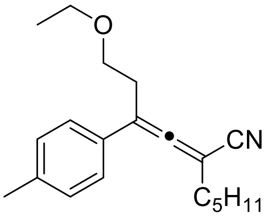

4f

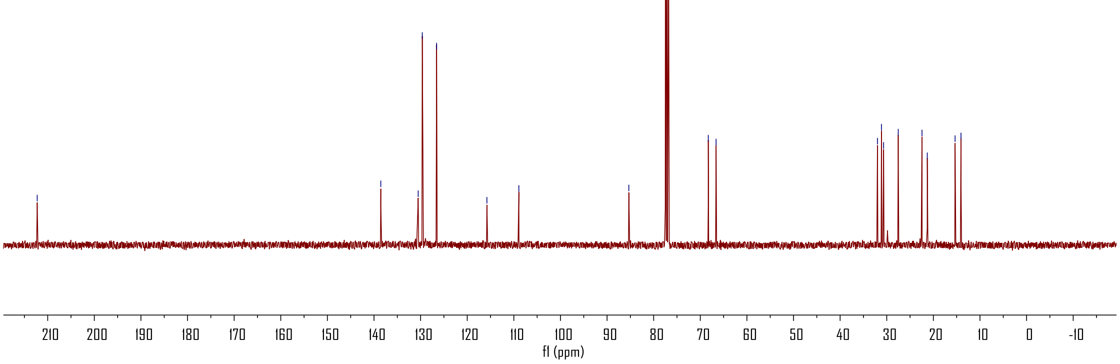

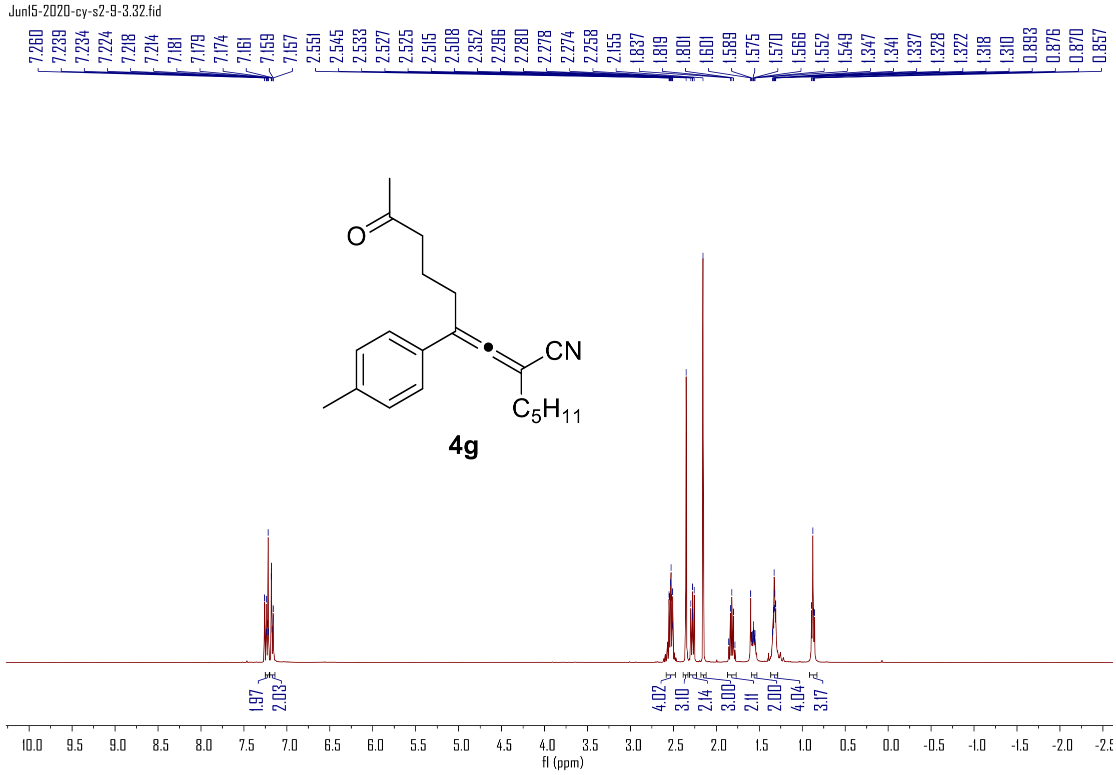
Jun15-2020-cy-s2-9-3.32.fid
7.260 7.239 7.234 7.224 7.218 7.214 7.181 7.179 7.174 7.161 7.159 7.157 2.551 2.545 2.533 2.527 2.525 2.515 2.508 2.352 2.296 2.280 2.278 2.274 2.258 2.155 1.837 1.819 1.801 1.601 1.589 1.575 1.570 1.566 1.552 1.549 1.347 1.341 1.337 1.328 1.322 1.318 1.310 0.893 0.876 0.870 0.857
O
CN
C_5H_{11}
4g
1.97 2.03 4.02 3.10 2.14 3.00 2.11 2.00 4.04 3.17
f1 (ppm)

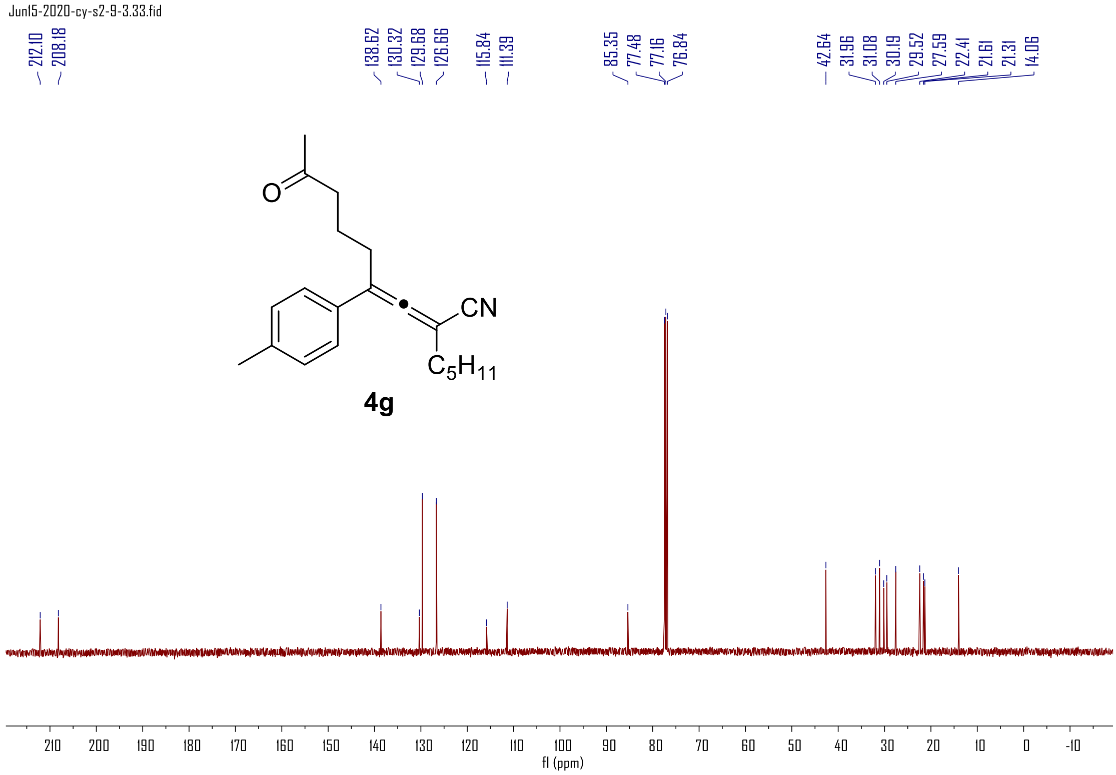
Jun15-2020-cy-s2-9-3.33.fid
212.10 208.18 138.62 130.32 129.68 126.66 115.84 111.39 85.35 77.48 77.16 76.84 42.64 31.96 31.08 30.19 29.52 27.59 22.41 21.61 21.31 14.06
O
CN
C_5H_{11}
4g
f1 (ppm)

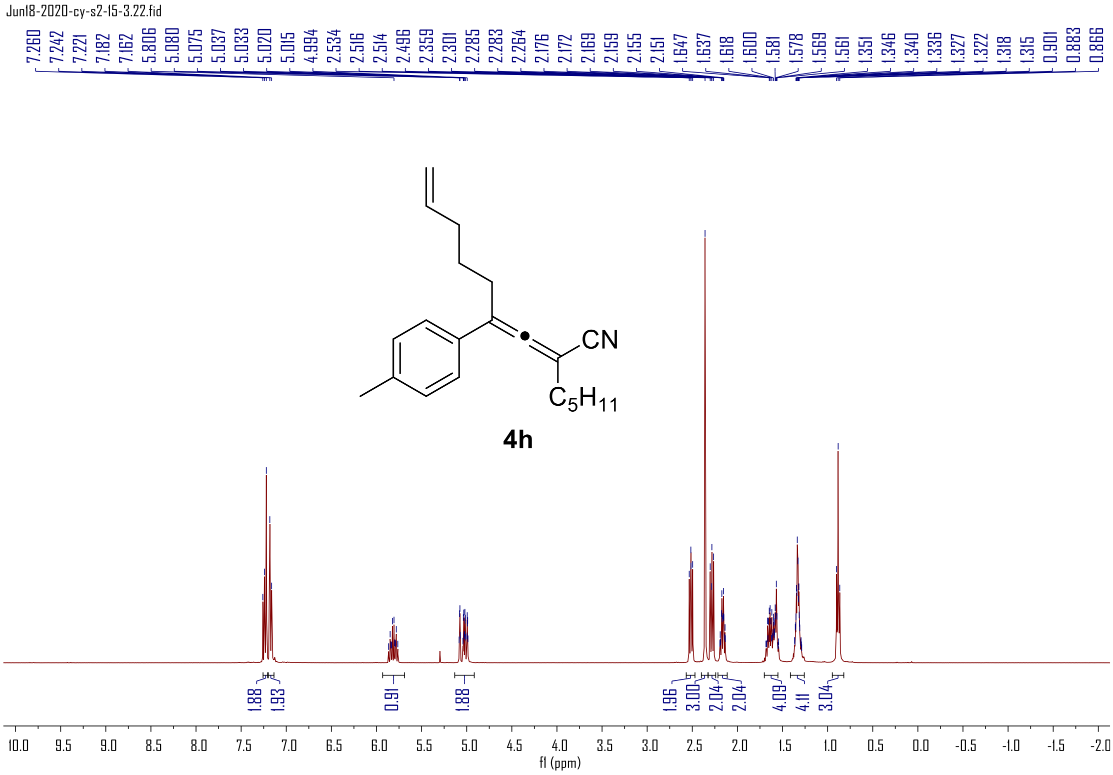
Jun18-2020-cy-s2-15-3.22.fid
7.260 7.242 7.221 7.182 7.162 5.806 5.080 5.075 5.037 5.033 5.020 5.015 4.994 2.534 2.516 2.514 2.496 2.359 2.301 2.285 2.283 2.264 2.176 2.172 2.169 2.159 2.155 2.151 1.647 1.637 1.618 1.600 1.581 1.578 1.569 1.561 1.351 1.346 1.340 1.336 1.327 1.322 1.318 1.315 0.901 0.883 0.866
CN
C_5H_{11}
4h
1.88 1.93 0.91 1.88 1.96 3.00 2.04 2.04 4.09 4.11 3.04
10.0 9.5 9.0 8.5 8.0 7.5 7.0 6.5 6.0 5.5 5.0 4.5 4.0 3.5 3.0 2.5 2.0 1.5 1.0 0.5 0.0 -0.5 -1.0 -1.5 -2.0
f1 (ppm)

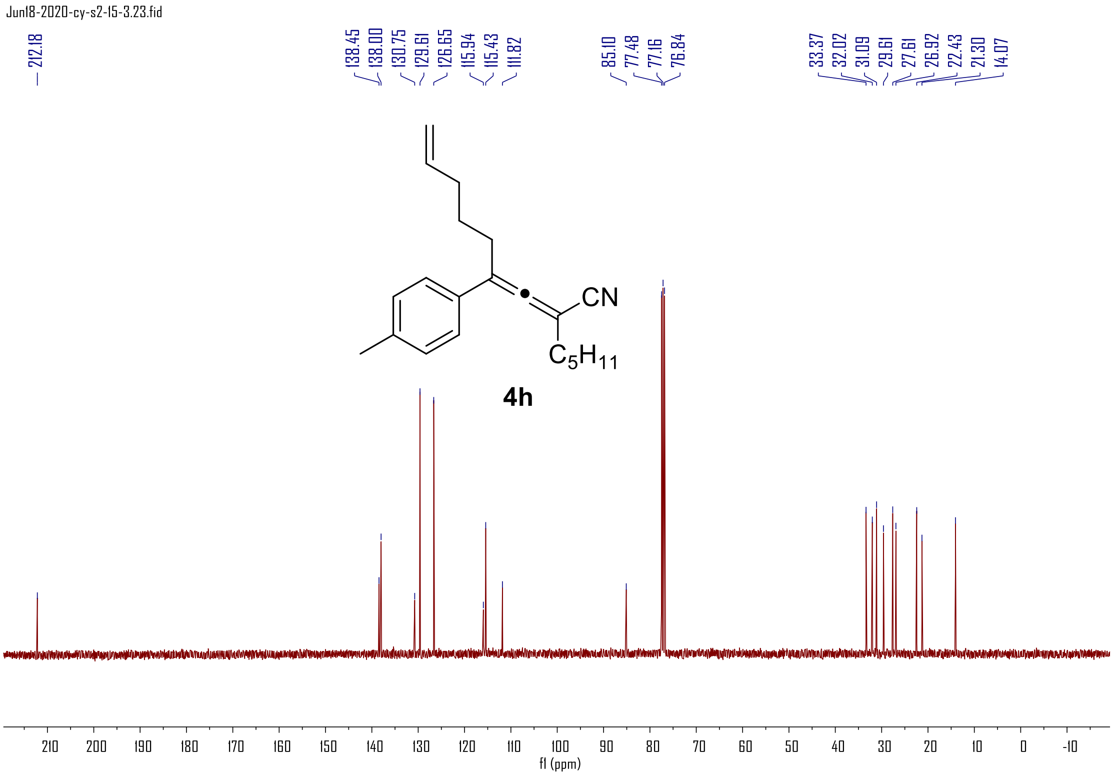
Jun18-2020-cy-s2-15-3.23.fid
212.18
138.45 138.00 130.75 129.61 126.65 115.94 115.43 111.82
85.10 77.48 77.16 76.84
33.37 32.02 31.09 29.61 27.61 26.92 22.43 21.30 14.07
CN
C_5H_{11}
4h
210 200 190 180 170 160 150 140 130 120 110 100 90 80 70 60 50 40 30 20 10 0 -10
f1 (ppm)

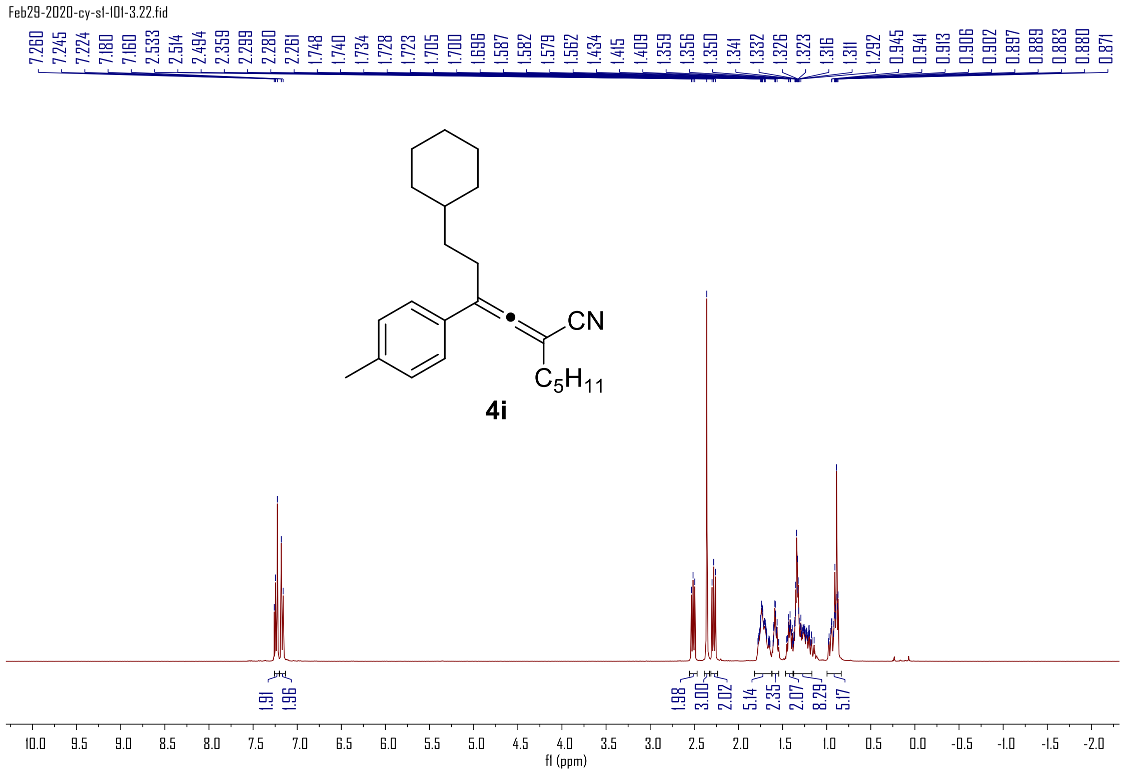
Feb29-2020-cy-sl-101-3.22.fid
7.260 7.245 7.224 7.180 7.160 2.533 2.514 2.494 2.359 2.299 2.280 2.261 1.748 1.740 1.734 1.728 1.723 1.705 1.700 1.696 1.587 1.582 1.579 1.562 1.434 1.415 1.409 1.359 1.356 1.350 1.341 1.332 1.326 1.323 1.316 1.311 1.292 0.945 0.941 0.913 0.906 0.902 0.897 0.889 0.883 0.880 0.871
CN
C_5H_{11}
4i
1.91 1.96 1.98 3.00 2.02 5.14 2.35 2.07 8.29 5.17
10.0 9.5 9.0 8.5 8.0 7.5 7.0 6.5 6.0 5.5 5.0 4.5 4.0 3.5 3.0 2.5 2.0 1.5 1.0 0.5 0.0 -0.5 -1.0 -1.5 -2.0
f1 (ppm)

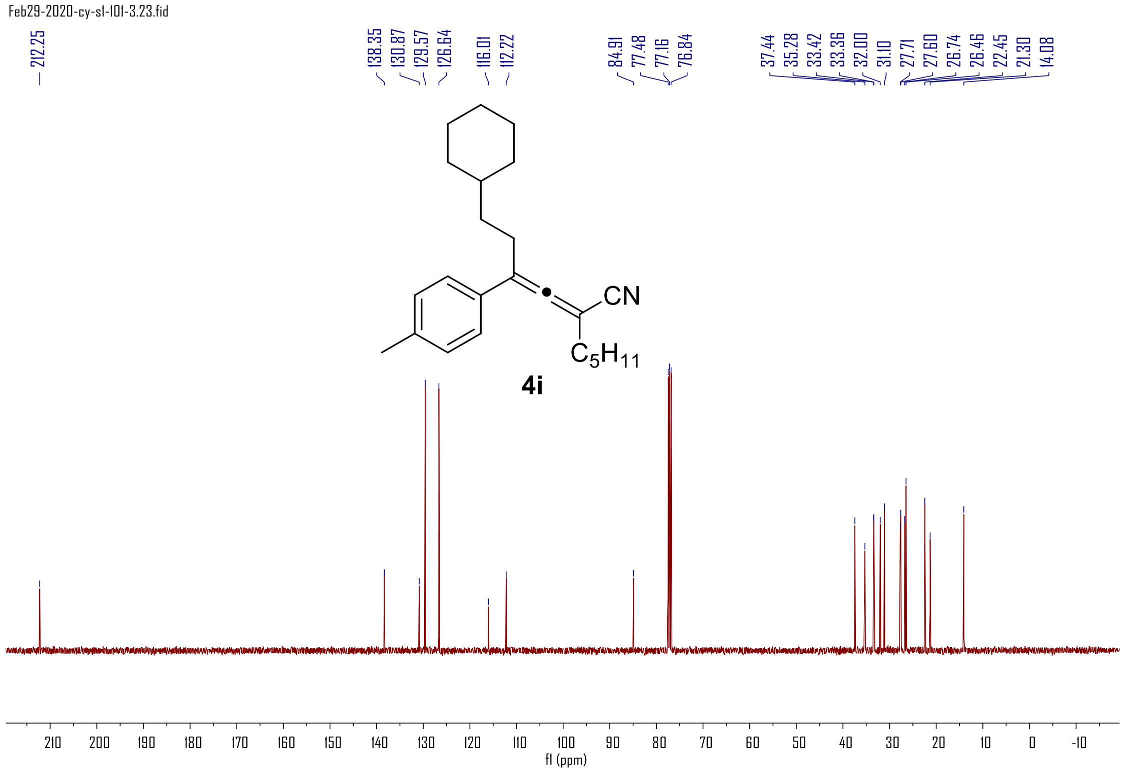
Feb29-2020-cy-sl-101-3.23.fid
212.25 138.35 130.87 129.57 126.64 116.01 112.22 84.91 77.48 77.16 76.84 37.44 35.28 33.42 33.36 32.00 31.10 27.71 27.60 26.74 26.46 22.45 21.30 14.08
CN
C_5H_{11}
4i
210 200 190 180 170 160 150 140 130 120 110 100 90 80 70 60 50 40 30 20 10 0 -10
f1 (ppm)

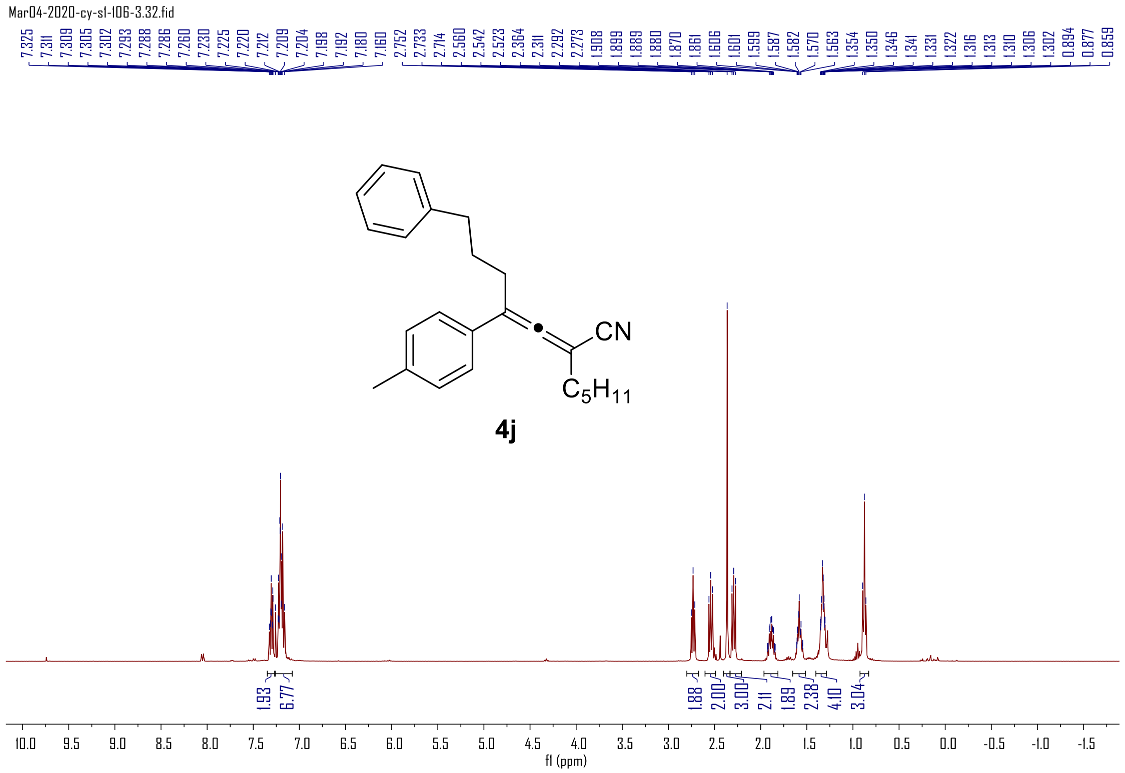
Mar04-2020-cy-sl-106-3.32.fid
7.325 7.311 7.309 7.305 7.302 7.293 7.288 7.286 7.260 7.230 7.225 7.220 7.212 7.209 7.204 7.198 7.192 7.180 7.160 2.752 2.733 2.714 2.560 2.542 2.523 2.364 2.311 2.292 2.273 1.908 1.899 1.889 1.880 1.870 1.861 1.606 1.601 1.599 1.587 1.582 1.570 1.563 1.354 1.350 1.346 1.341 1.331 1.322 1.316 1.313 1.310 1.306 1.302 0.894 0.877 0.859
CN
C_5H_{11}
4j
1.93 6.77 1.88 2.00 3.00 2.11 1.89 2.38 4.10 3.04
10.0 9.5 9.0 8.5 8.0 7.5 7.0 6.5 6.0 5.5 5.0 4.5 4.0 3.5 3.0 2.5 2.0 1.5 1.0 0.5 0.0 -0.5 -1.0 -1.5
f1 (ppm)

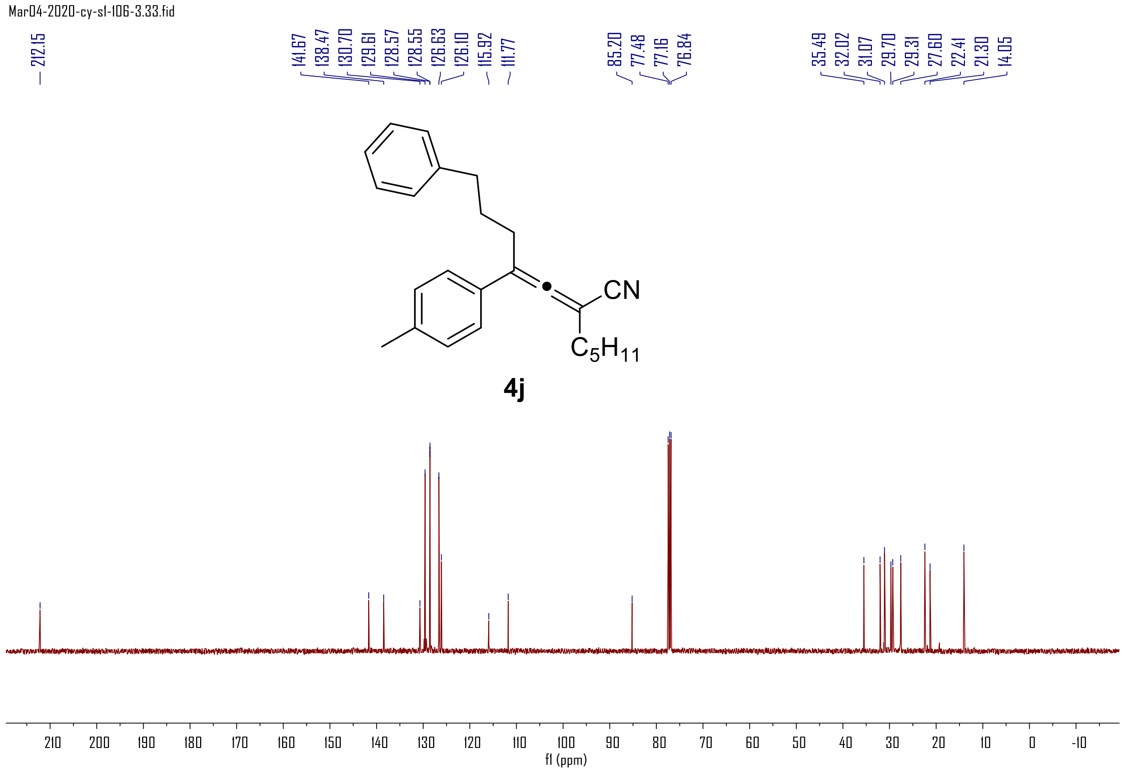
Mar04-2020-cy-sl-106-3.33.fid
212.15 141.67 138.47 130.70 129.61 128.57 128.55 126.63 126.10 115.92 111.77 85.20 77.48 77.16 76.84 35.49 32.02 31.07 29.70 29.31 27.60 22.41 21.30 14.05
CN
C_5H_{11}
4j
210 200 190 180 170 160 150 140 130 120 110 100 90 80 70 60 50 40 30 20 10 0 -10
f1 (ppm)

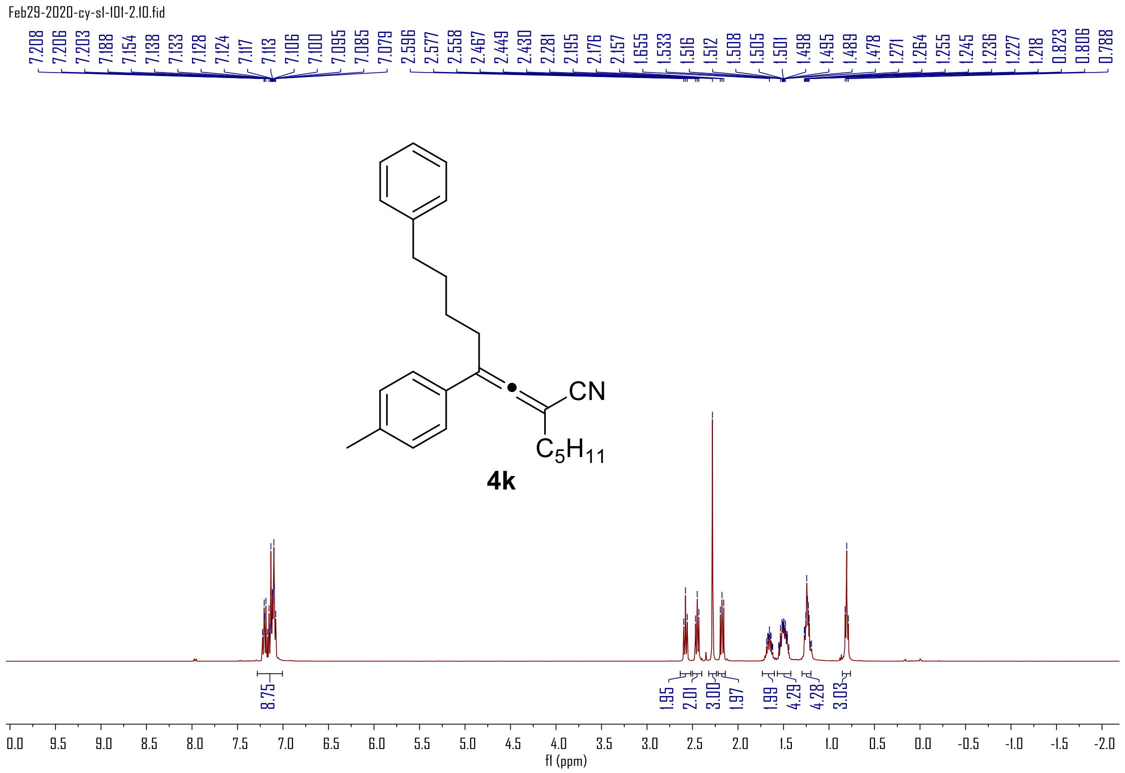
Feb29-2020-cy-sl-101-2.10.fid
7.208 7.206 7.203 7.188 7.154 7.138 7.133 7.128 7.124 7.117 7.113 7.106 7.100 7.095 7.085 7.079 2.596 2.577 2.558 2.467 2.449 2.430 2.281 2.195 2.176 2.157 1.655 1.533 1.516 1.512 1.508 1.505 1.501 1.498 1.495 1.489 1.478 1.271 1.264 1.255 1.245 1.236 1.227 1.218 0.823 0.806 0.788
CN
C_5H_{11}
4k
8.75
1.95 2.01 3.00 1.97 1.99 4.29 4.28 3.03
f1 (ppm)

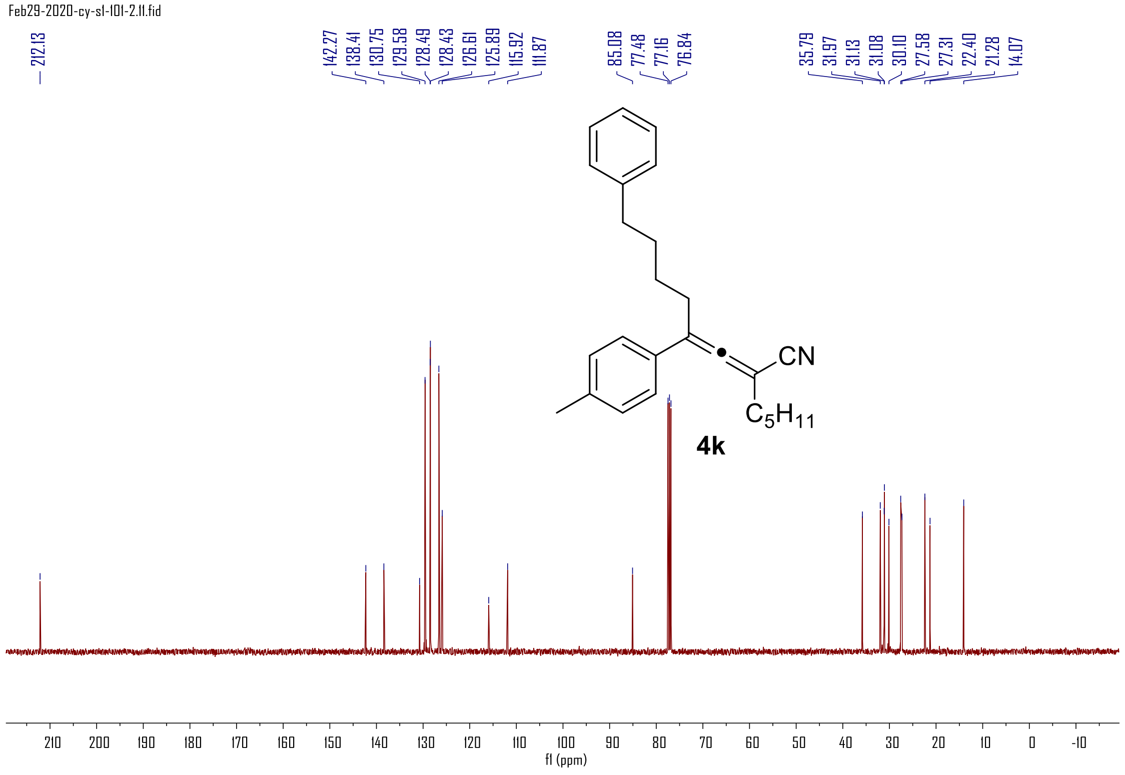
Feb29-2020-cy-sl-101-2.11.fid
212.13
142.27 138.41 130.75 129.58 128.49 128.43 126.61 125.89 115.92 111.87
85.08 77.48 77.16 76.84
35.79 31.97 31.13 31.08 30.10 27.58 27.31 22.40 21.28 14.07
CN
C_5H_{11}
4k
f1 (ppm)

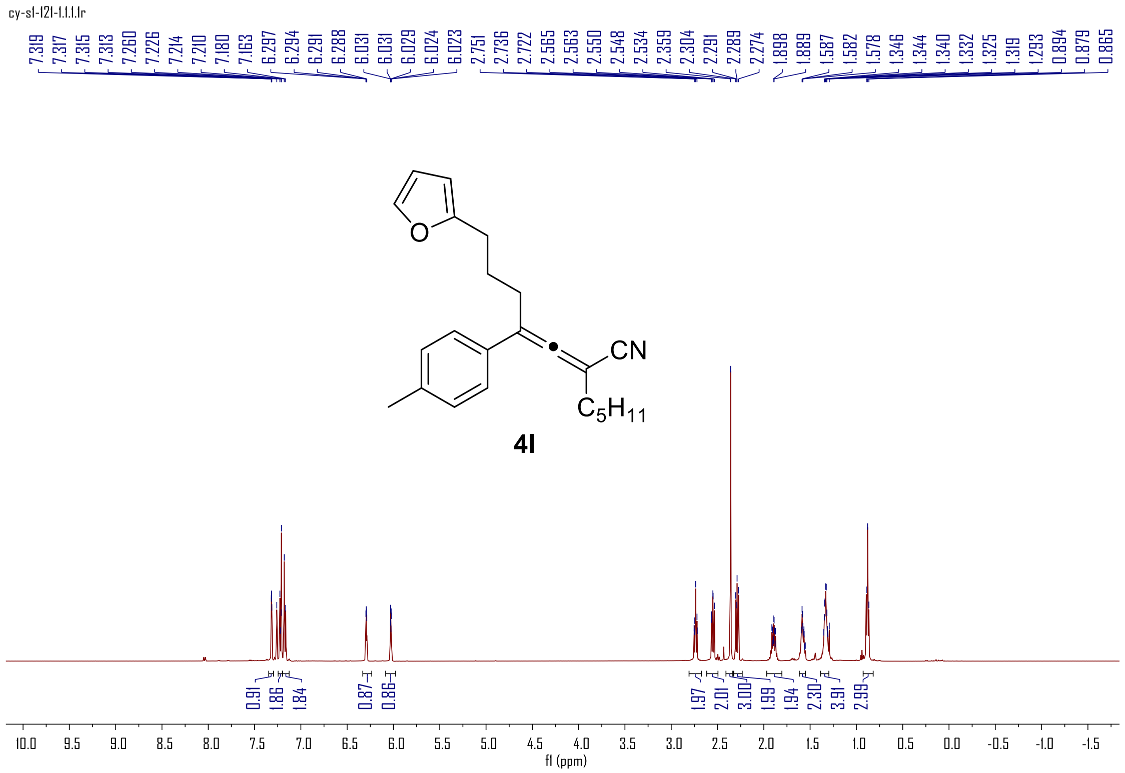
cy-sl-121-1.1.1.fr
7.319 7.317 7.315 7.313 7.260 7.226 7.214 7.210 7.180 7.163 6.297 6.294 6.291 6.288 6.031 6.029 6.024 6.023 2.751 2.736 2.722 2.565 2.563 2.550 2.548 2.534 2.359 2.304 2.291 2.289 2.274 1.898 1.889 1.587 1.582 1.578 1.346 1.344 1.340 1.332 1.325 1.319 1.293 0.894 0.879 0.865
CN
O
4l
0.91 1.86 1.84 0.87 0.86 1.97 2.01 3.00 1.99 1.94 2.30 3.91 2.99
f1 (ppm)

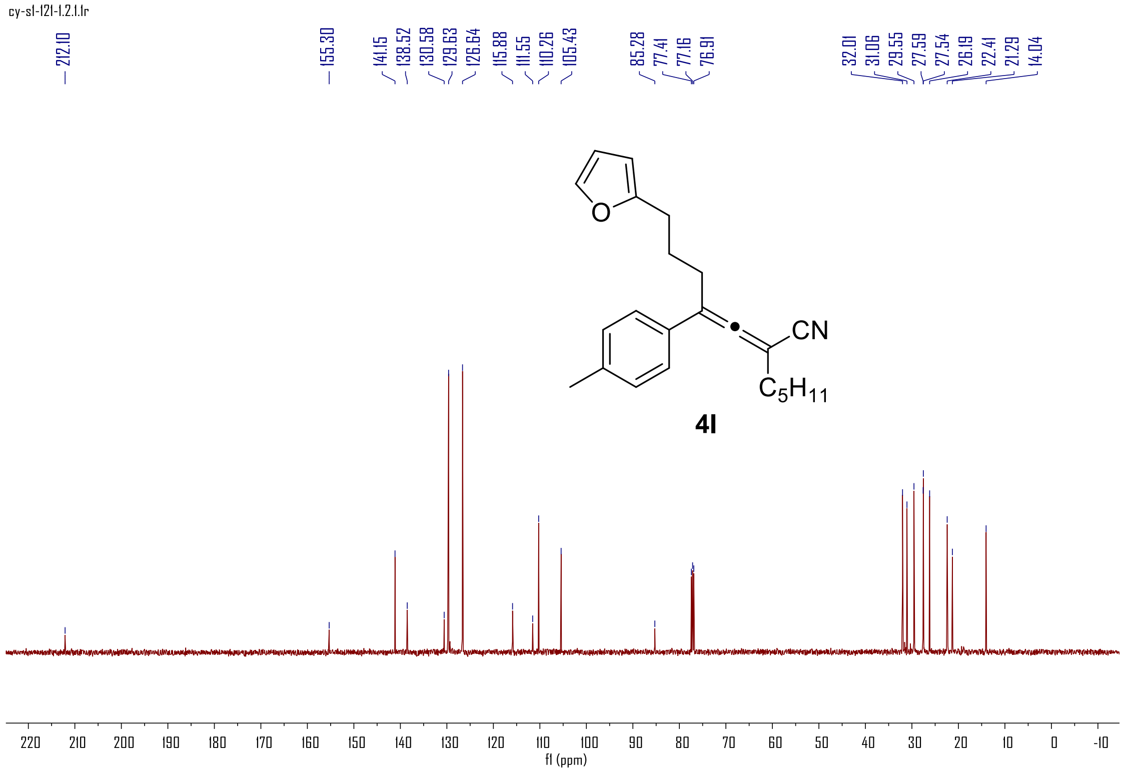
cy-sl-121-1.2.1.fr
212.10 155.30 141.15 138.52 130.58 129.63 126.64 115.88 111.55 110.26 105.43 85.28 77.41 77.16 76.91 32.01 31.06 29.55 27.59 27.54 26.19 22.41 21.29 14.04
CN
O
4l
f1 (ppm)

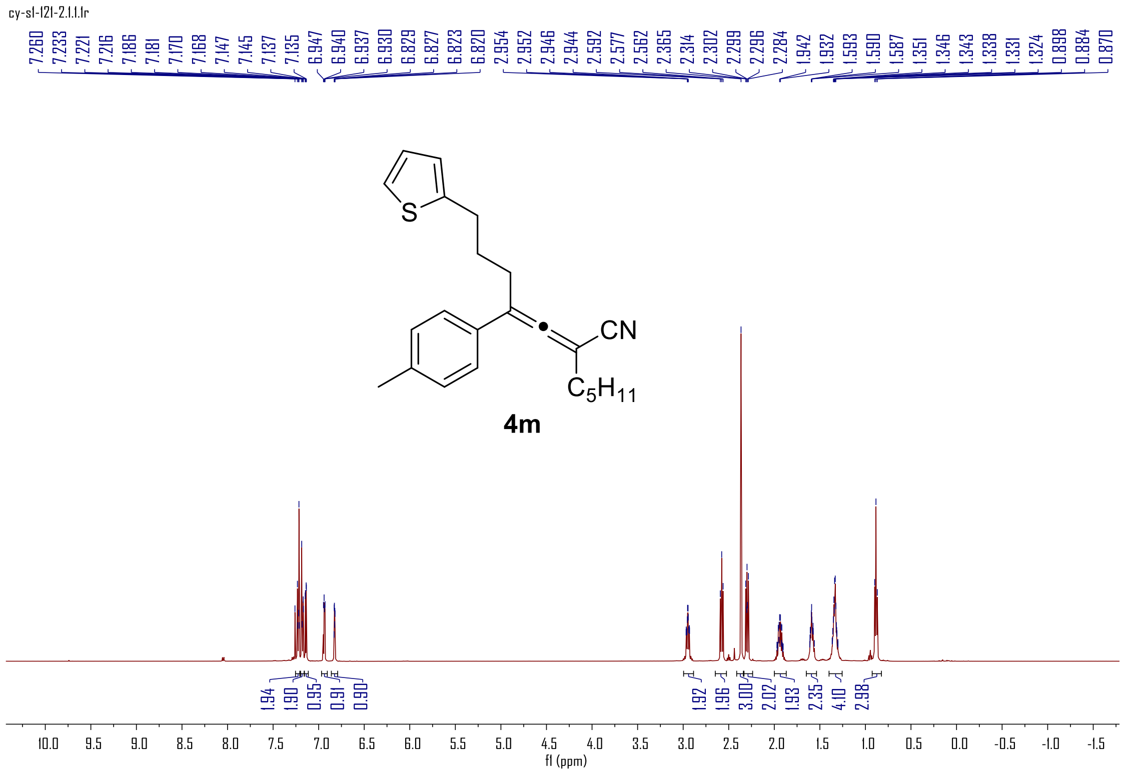
cy-sl-121-2.1.1.1r
7.260 7.233 7.221 7.216 7.186 7.181 7.170 7.168 7.147 7.145 7.137 7.135 6.947 6.940 6.937 6.930 6.829 6.827 6.823 6.820 2.954 2.952 2.946 2.944 2.592 2.577 2.562 2.365 2.314 2.302 2.299 2.296 2.284 1.942 1.932 1.593 1.590 1.587 1.351 1.346 1.343 1.338 1.331 1.324 0.898 0.884 0.870
S
CN
C_5H_{11}
4m
1.94 1.90 0.95 0.91 0.90 1.92 1.96 3.00 2.02 1.93 2.35 4.10 2.98
f1 (ppm)

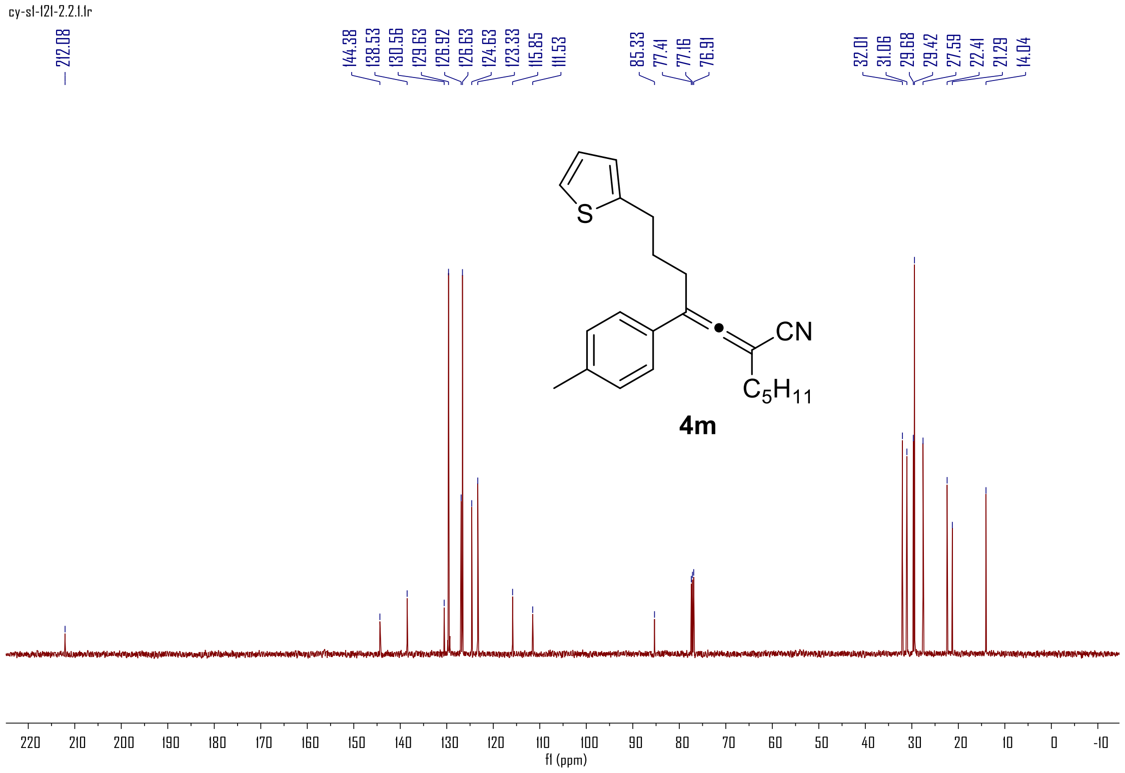
cy-sl-121-2.2.1.1r
212.08
144.38 138.53 130.56 129.63 126.92 126.63 124.63 123.33 115.85 111.53
85.33 77.41 77.16 76.91
32.01 31.06 29.68 29.42 27.59 22.41 21.29 14.04
S
CN
C_5H_{11}
4m
f1 (ppm)

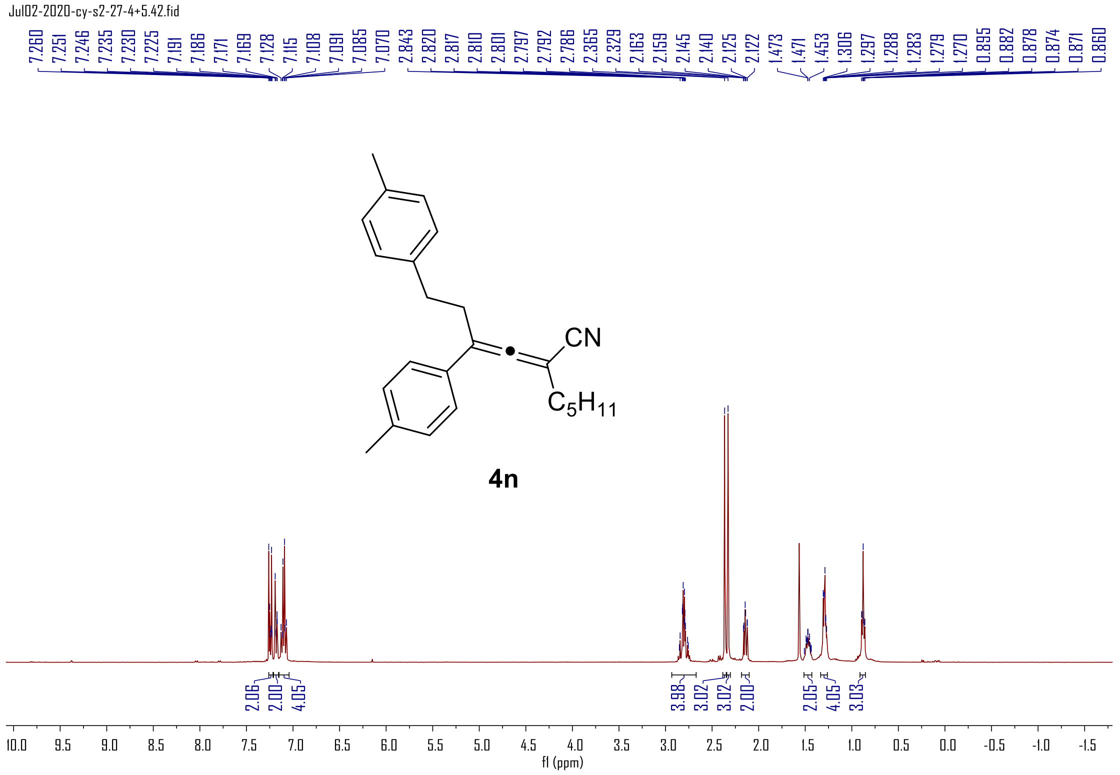
Jul02-2020-cy-s2-27-4+5.42.fid
7.260 7.251 7.246 7.235 7.230 7.225 7.191 7.186 7.171 7.169 7.128 7.115 7.108 7.091 7.085 7.070 2.843 2.820 2.817 2.810 2.801 2.797 2.792 2.786 2.365 2.329 2.163 2.159 2.145 2.140 2.125 2.122 1.473 1.471 1.453 1.306 1.297 1.288 1.283 1.279 1.270 0.895 0.882 0.878 0.874 0.871 0.860
CN
C5H11
4n
2.06 2.00 4.05 3.98 3.02 3.02 2.00 2.05 4.05 3.03
fl (ppm)

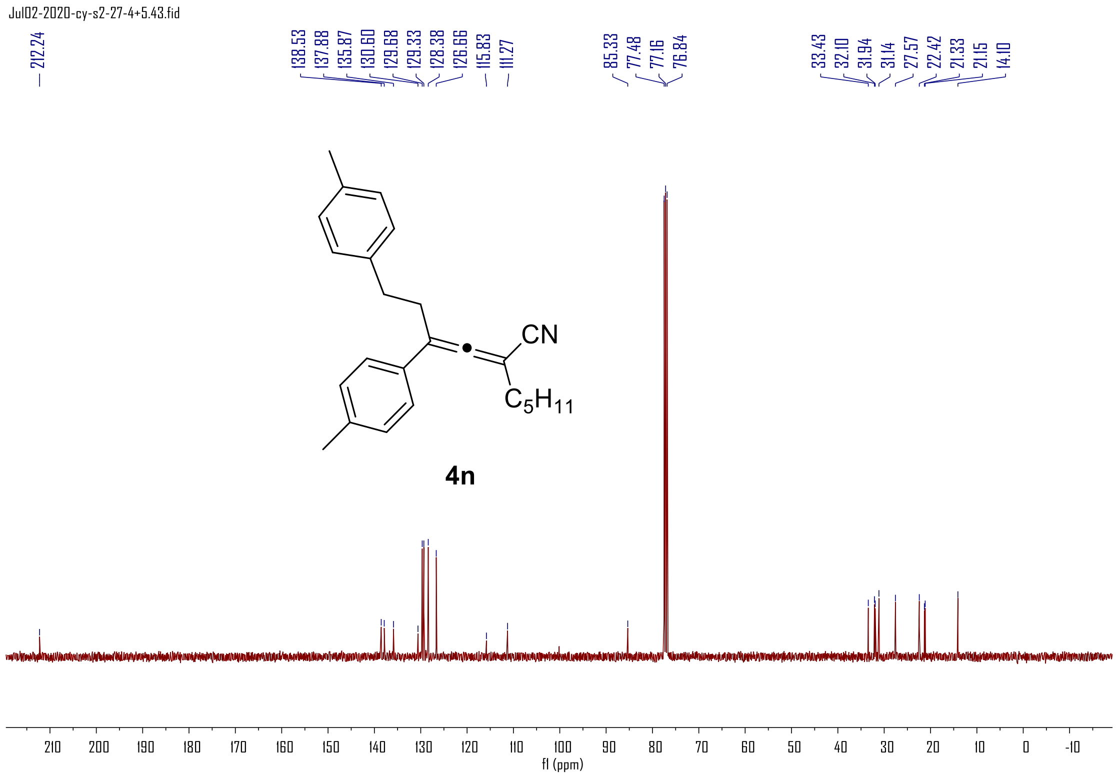
Jul02-2020-cy-s2-27-4+5.43.fid
212.24 138.53 137.88 135.87 130.60 129.68 129.33 128.38 126.66 115.83 111.27 85.33 77.48 77.16 76.84 33.43 32.10 31.94 31.14 27.57 22.42 21.33 21.15 14.10
CN
C5H11
4n
fl (ppm)

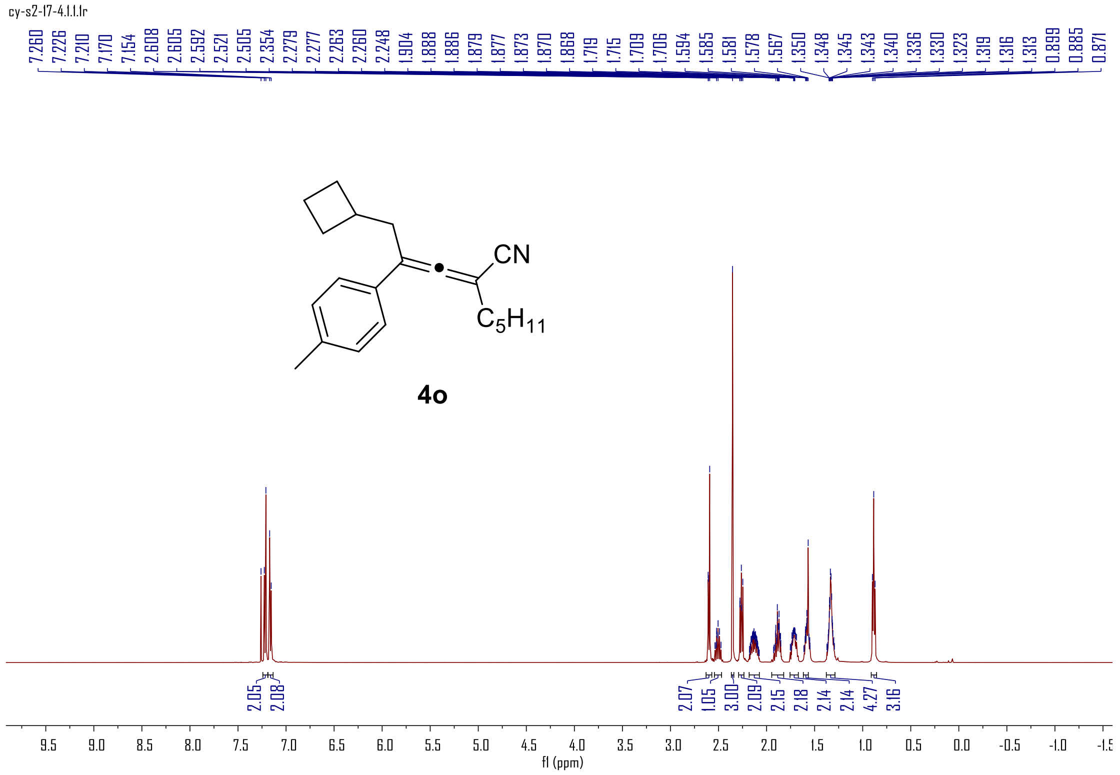
cy-s2-17-4.1.1.1r
CN
C5H11
4o
fl (ppm)

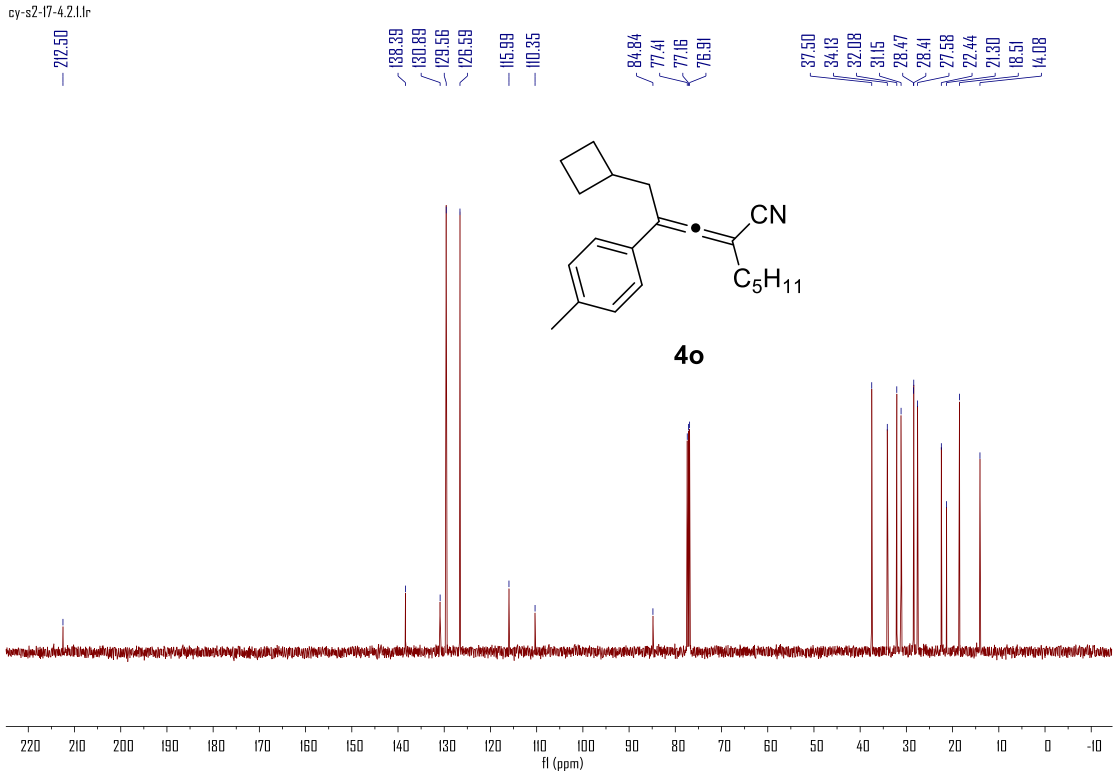
cy-s2-17-4.2.1.1r
CN
C5H11
4o
fl (ppm)

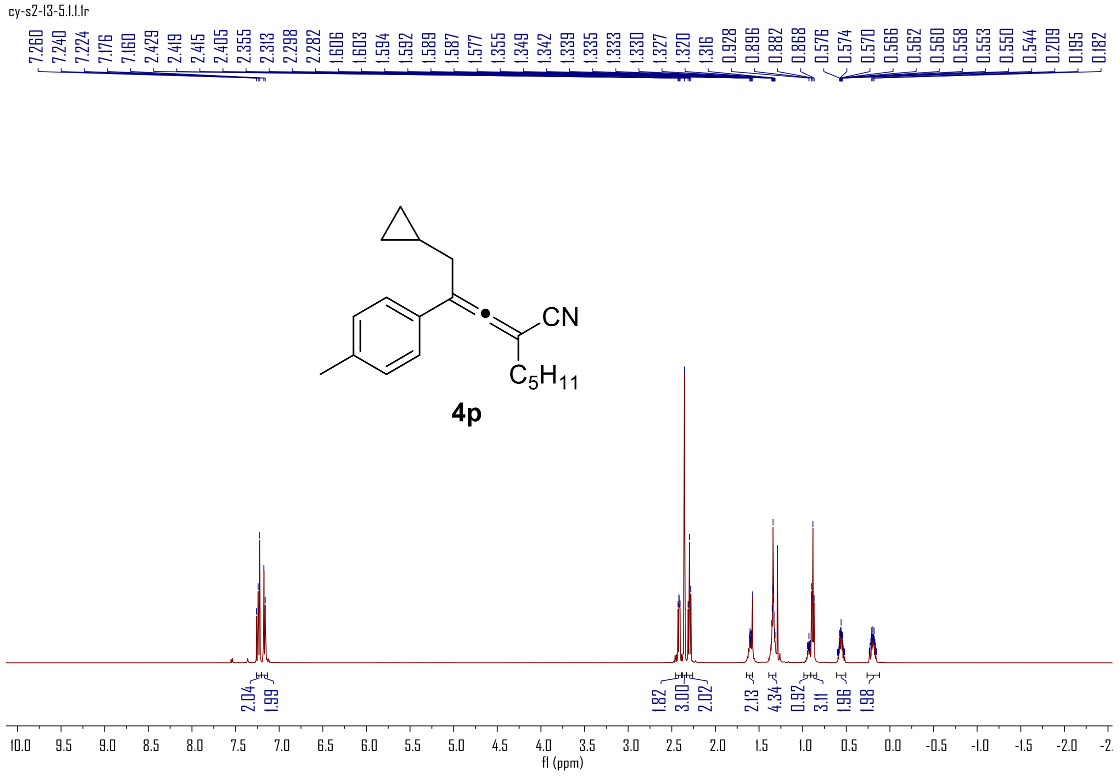
cy-s2-13-5.1.1.1r
7.260
7.240
7.224
7.176
7.160
2.429
2.419
2.415
2.405
2.355
2.313
2.298
2.282
1.606
1.603
1.594
1.592
1.589
1.587
1.577
1.355
1.349
1.342
1.339
1.335
1.333
1.330
1.327
1.320
1.316
0.928
0.896
0.882
0.868
0.576
0.574
0.570
0.566
0.562
0.560
0.558
0.553
0.550
0.544
0.209
0.195
0.182
CN
4p
2.04
1.99
1.82
3.00
2.02
2.13
4.34
0.92
3.11
1.96
1.98
f1 (ppm)

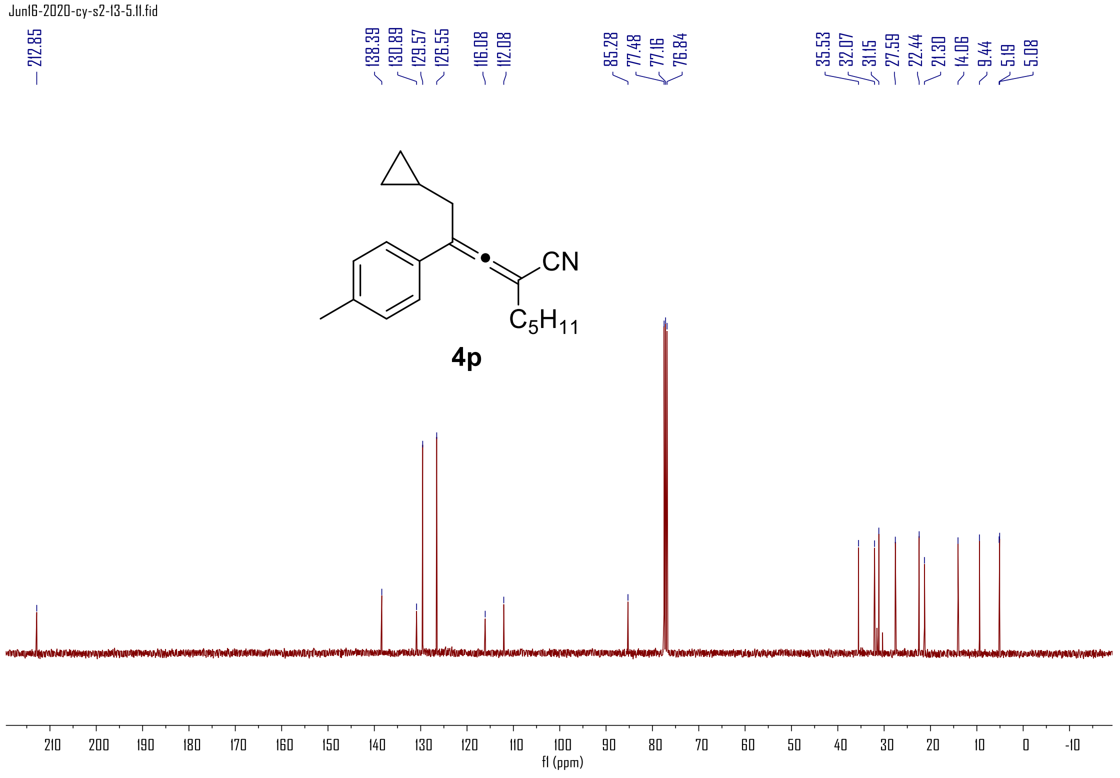
Jun16-2020-cy-s2-13-5.11.fid
212.85
138.39
130.89
129.57
126.55
116.08
112.08
85.28
77.48
77.16
76.84
35.53
32.07
31.15
27.59
22.44
21.30
14.06
9.44
5.19
5.08
CN
4p
f1 (ppm)

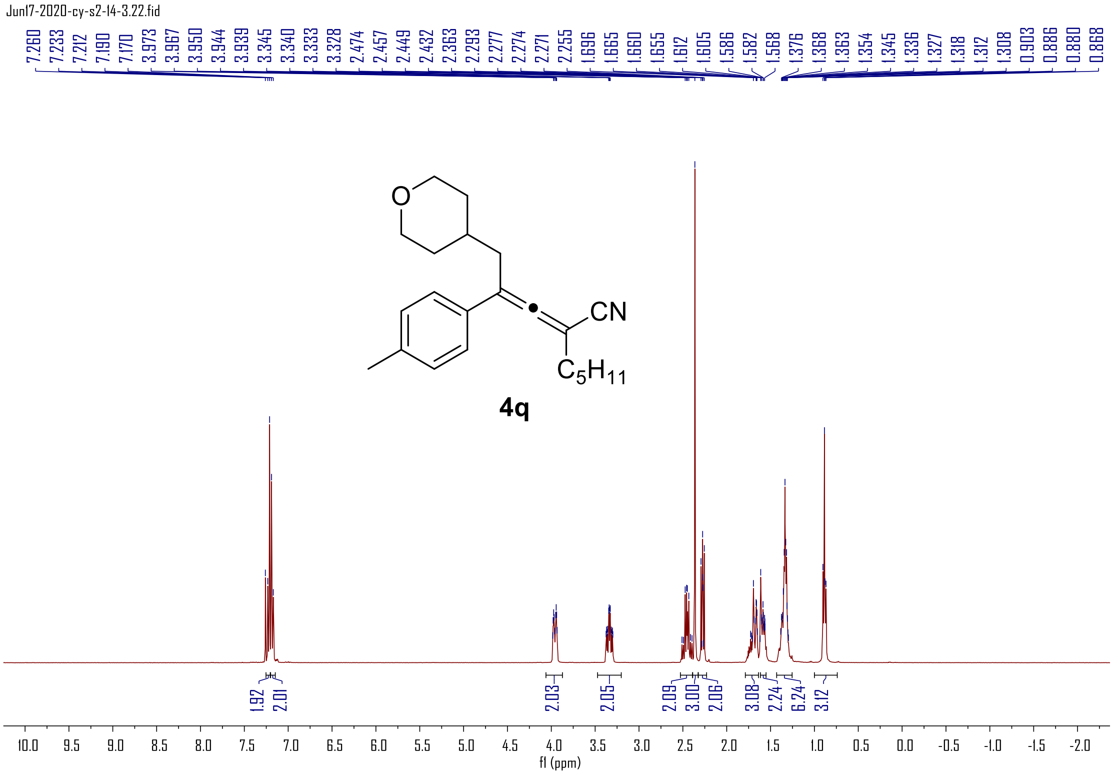
Jun17-2020-cy-s2-14-3.22.fid
4q
CN
fl (ppm)

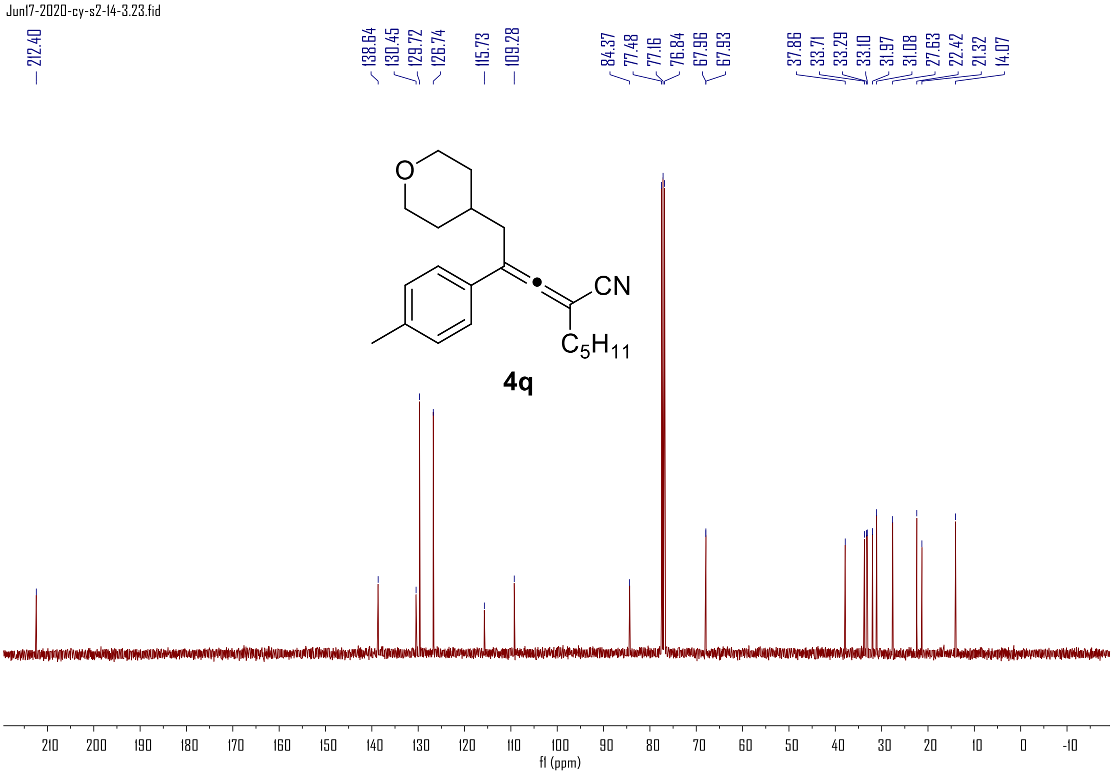
Jun17-2020-cy-s2-14-3.23.fid
4q
CN
fl (ppm)

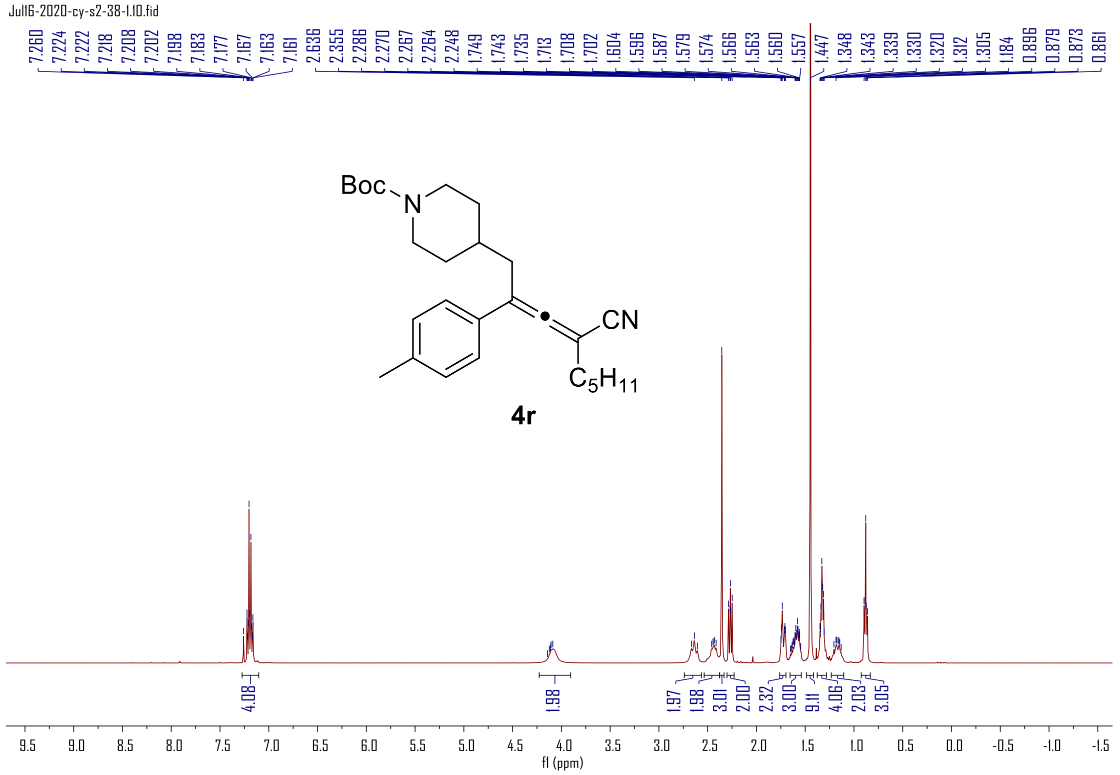
Jul16-2020-cy-s2-38-1.10.fid
7.260 7.224 7.222 7.218 7.208 7.202 7.198 7.183 7.177 7.167 7.163 7.161 2.636 2.355 2.286 2.270 2.267 2.264 2.248 1.749 1.743 1.735 1.713 1.708 1.702 1.604 1.596 1.587 1.579 1.574 1.566 1.563 1.560 1.557 1.447 1.348 1.343 1.339 1.330 1.320 1.312 1.305 1.184 0.896 0.879 0.873 0.861
Boc
N
CN
C_5H_{11}
4r
4.08
1.98
1.97 1.98 3.01 2.00 2.32 3.00 9.11 4.06 2.03 3.05
f1 (ppm)

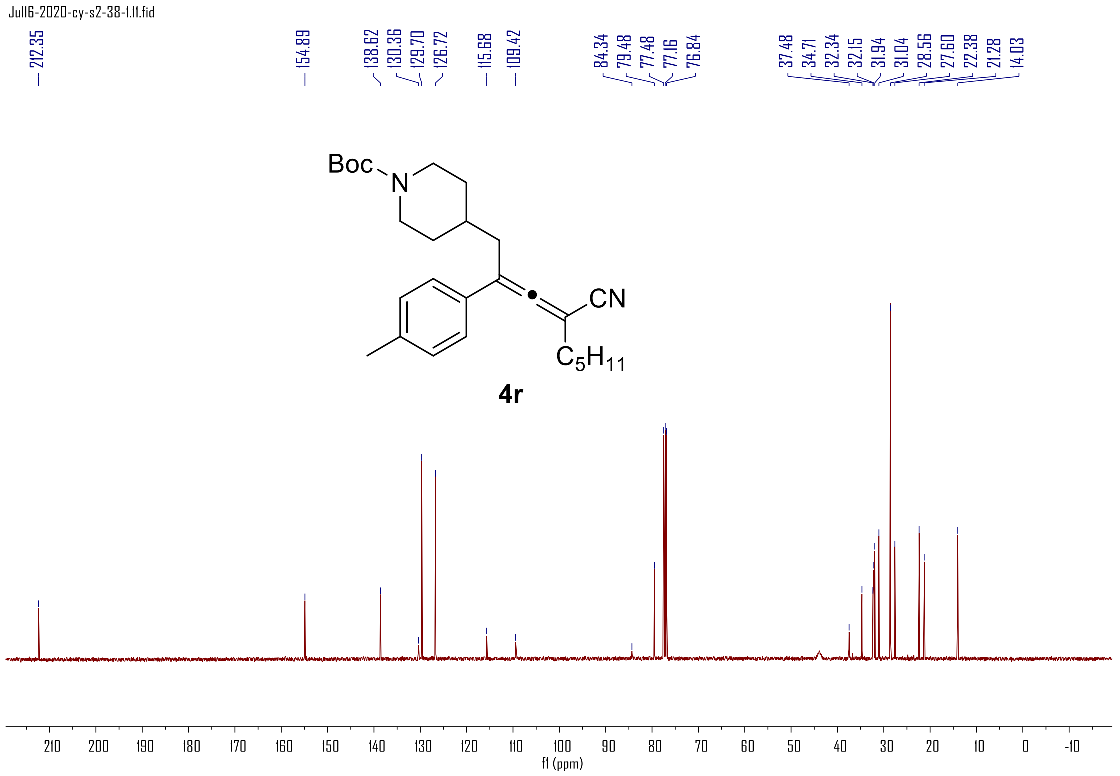
Jul16-2020-cy-s2-38-1.11.fid
212.35 154.89 138.62 130.36 129.70 126.72 115.68 109.42 84.34 79.48 77.48 77.16 76.84 37.48 34.71 32.34 32.15 31.94 31.04 28.56 27.60 22.38 21.28 14.03
Boc
N
CN
C_5H_{11}
4r
f1 (ppm)

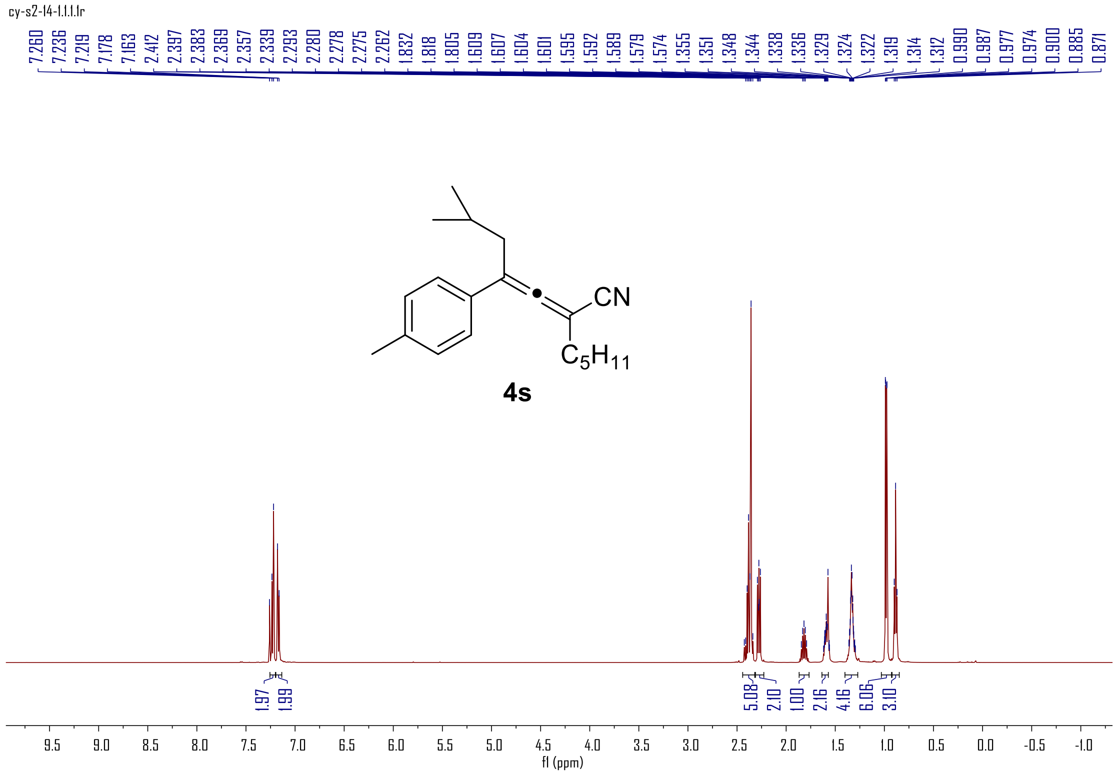
cy-s2-14-1.1.1.fr
7.260 7.236 7.219 7.178 7.163 2.412 2.397 2.383 2.369 2.357 2.339 2.293 2.280 2.278 2.275 2.262 1.832 1.818 1.805 1.609 1.607 1.604 1.601 1.595 1.592 1.589 1.579 1.574 1.355 1.351 1.348 1.344 1.338 1.336 1.329 1.324 1.322 1.319 1.314 1.312 0.990 0.987 0.977 0.974 0.900 0.885 0.871
CN
C_5H_{11}
4s
1.97 1.99 5.08 2.10 1.00 2.16 4.16 6.06 3.10
9.5 9.0 8.5 8.0 7.5 7.0 6.5 6.0 5.5 5.0 4.5 4.0 3.5 3.0 2.5 2.0 1.5 1.0 0.5 0.0 -0.5 -1.0
f1 (ppm)

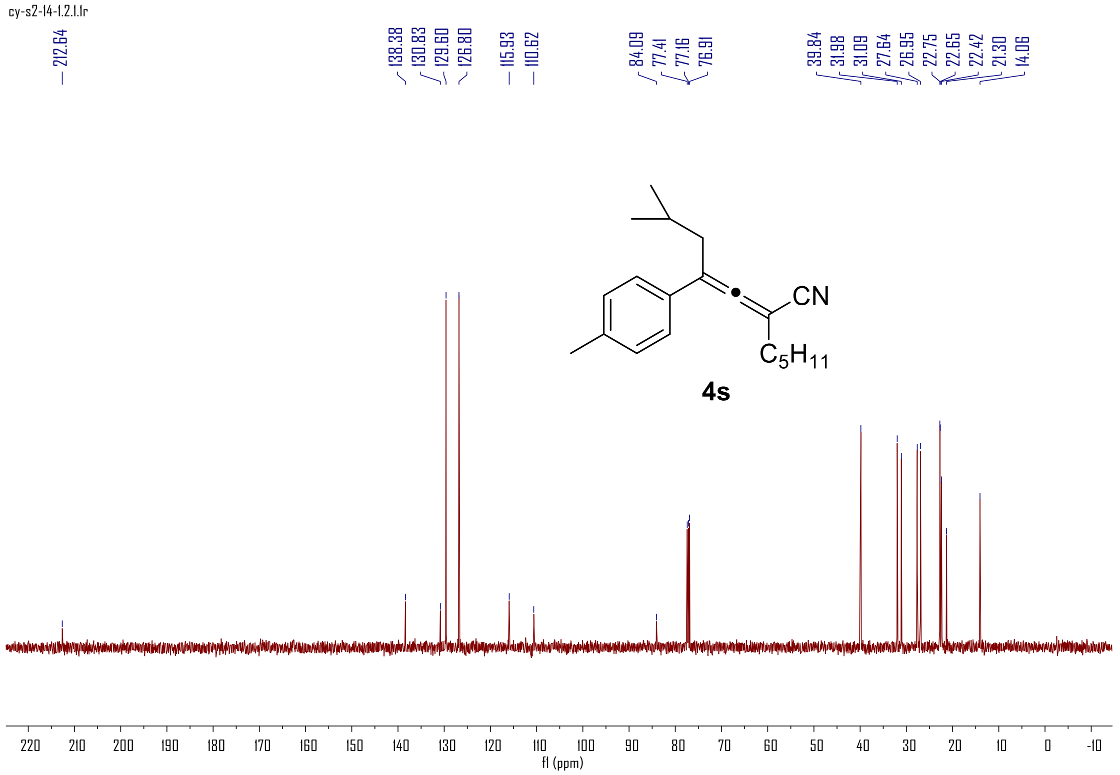
cy-s2-14-1.2.1.fr
212.64
138.38 130.83 129.60 126.80 115.93 110.62
84.09 77.41 77.16 76.91
39.84 31.98 31.09 27.64 26.95 22.75 22.65 22.42 21.30 14.06
CN
C_5H_{11}
4s
220 210 200 190 180 170 160 150 140 130 120 110 100 90 80 70 60 50 40 30 20 10 0 -10
f1 (ppm)

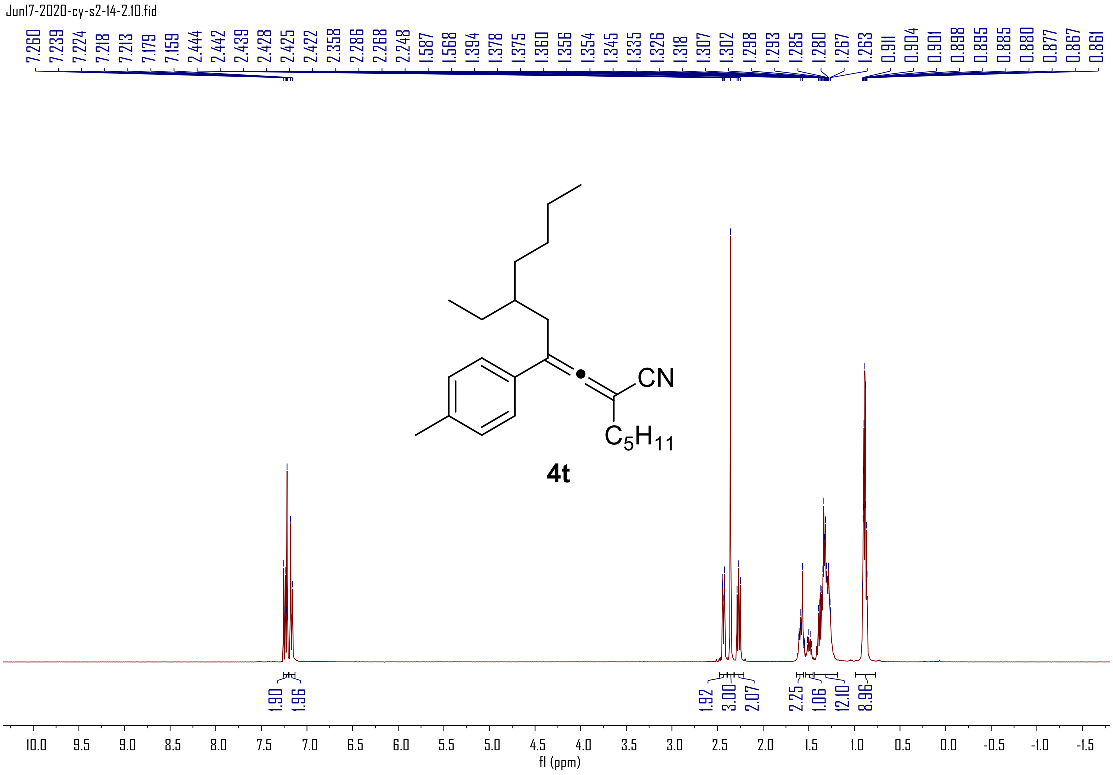

Jun17-2020-cy-s2-14-2.10.fid
7.260 7.239 7.224 7.218 7.213 7.179 7.159 2.444 2.442 2.439 2.428 2.425 2.422 2.358 2.286 2.268 2.248 1.587 1.568 1.394 1.378 1.375 1.360 1.356 1.354 1.345 1.335 1.326 1.318 1.307 1.302 1.298 1.293 1.285 1.280 1.267 1.263 0.911 0.904 0.901 0.898 0.895 0.885 0.880 0.877 0.867 0.861
CN
C_5H_{11}
4t
1.90 1.96 1.92 3.00 2.07 2.25 1.06 12.10 8.96
10.0 9.5 9.0 8.5 8.0 7.5 7.0 6.5 6.0 5.5 5.0 4.5 4.0 3.5 3.0 2.5 2.0 1.5 1.0 0.5 0.0 -0.5 -1.0 -1.5
f1 (ppm)

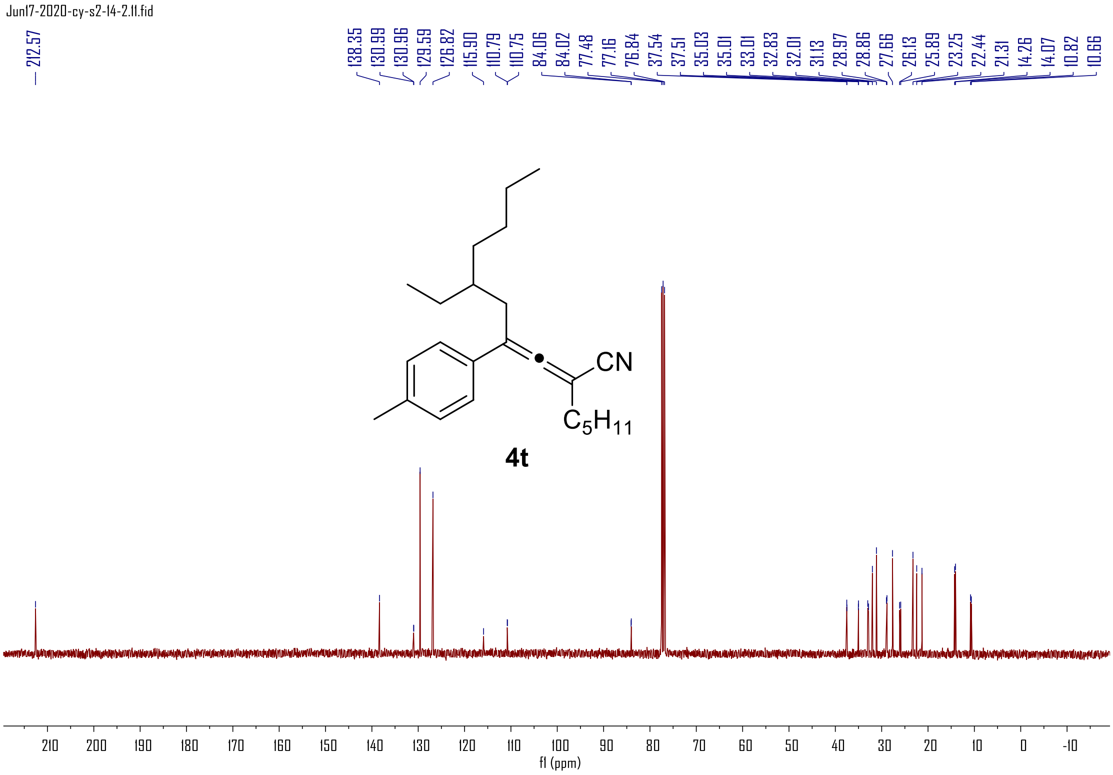

Jun17-2020-cy-s2-14-2.11.fid
212.57 138.35 130.99 130.96 129.59 126.82 115.90 110.79 110.75 84.06 84.02 77.48 77.16 76.84 37.54 37.51 35.03 35.01 33.01 32.83 32.01 31.13 28.97 28.86 27.66 26.13 25.89 23.25 22.44 21.31 14.26 14.07 10.82 10.66
CN
C_5H_{11}
4t
210 200 190 180 170 160 150 140 130 120 110 100 90 80 70 60 50 40 30 20 10 0 -10
f1 (ppm)

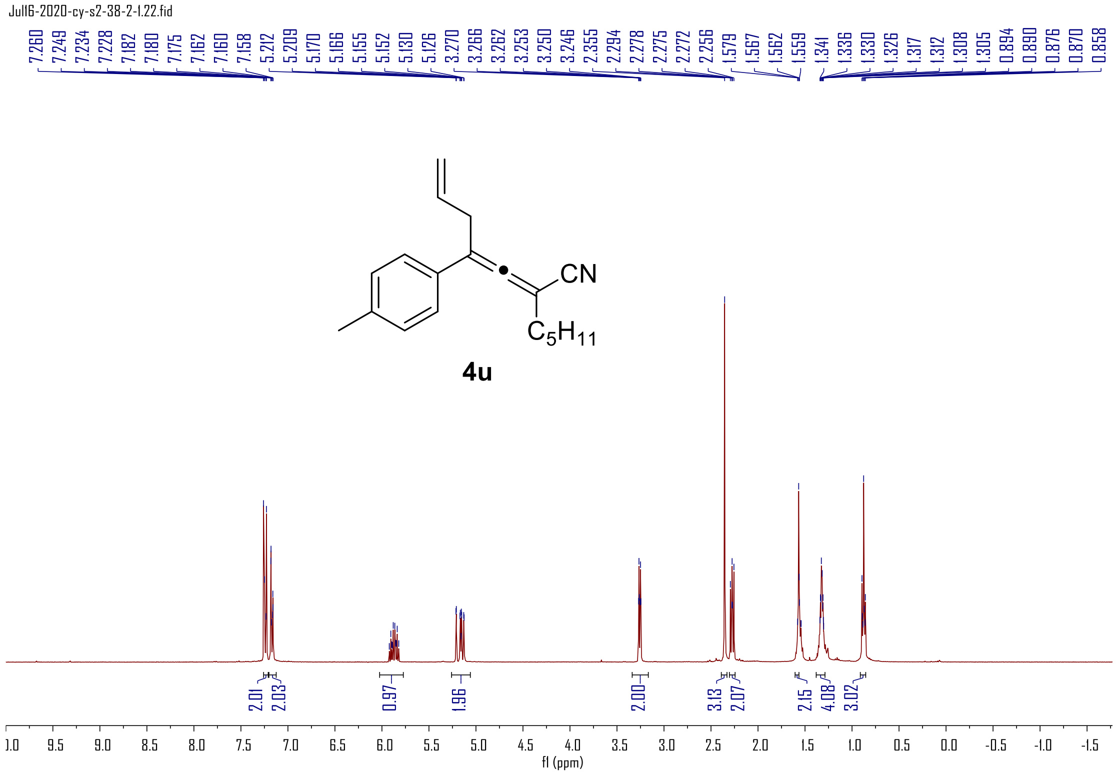
Jul16-2020-cy-s2-38-2-1.22.fid
7.260 7.249 7.234 7.228 7.182 7.180 7.175 7.162 7.160 7.158 5.212 5.209 5.170 5.166 5.155 5.152 5.130 5.126 3.270 3.266 3.262 3.253 3.250 3.246 2.355 2.294 2.278 2.275 2.272 2.256 1.579 1.567 1.562 1.559 1.341 1.336 1.330 1.326 1.317 1.312 1.308 1.305 0.894 0.890 0.876 0.870 0.858
CN
C5H11
4u
2.01 2.03 0.97 1.96 2.00 3.13 2.07 2.15 4.08 3.02
f1 (ppm)

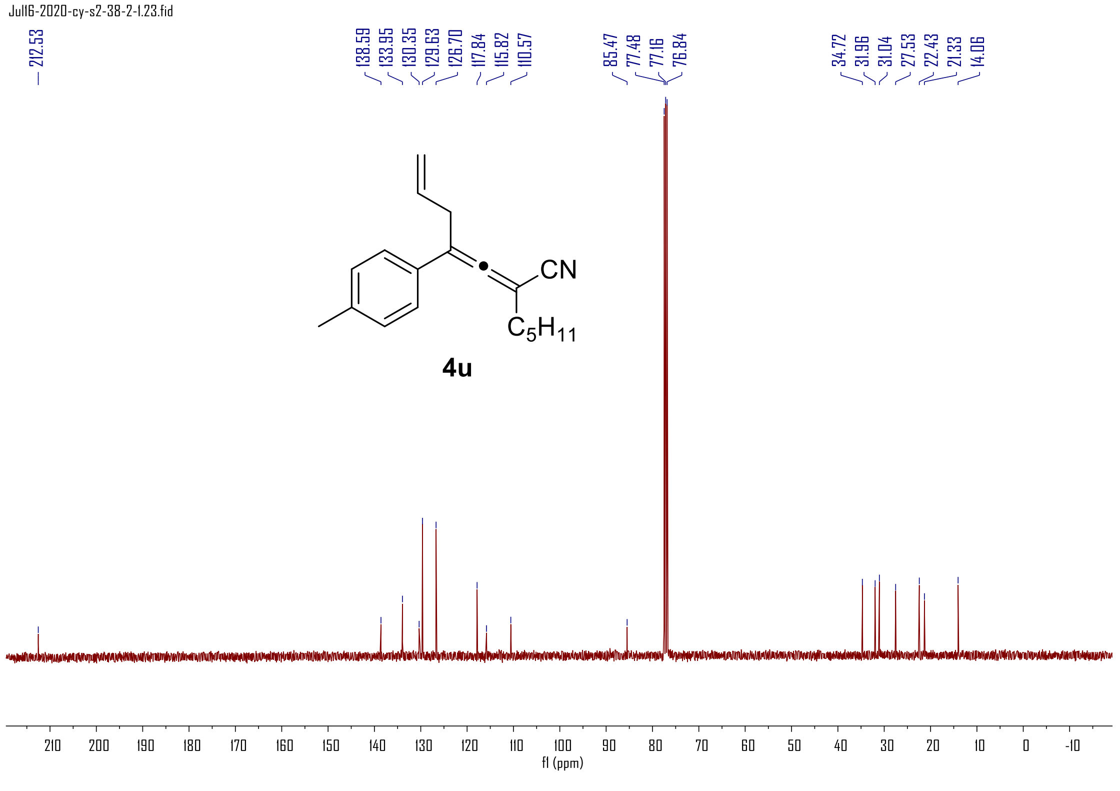
Jul16-2020-cy-s2-38-2-1.23.fid
212.53
138.59 133.95 130.35 129.63 126.70 117.84 115.82 110.57
85.47 77.48 77.16 76.84
34.72 31.96 31.04 27.53 22.43 21.33 14.06
CN
C5H11
4u
f1 (ppm)

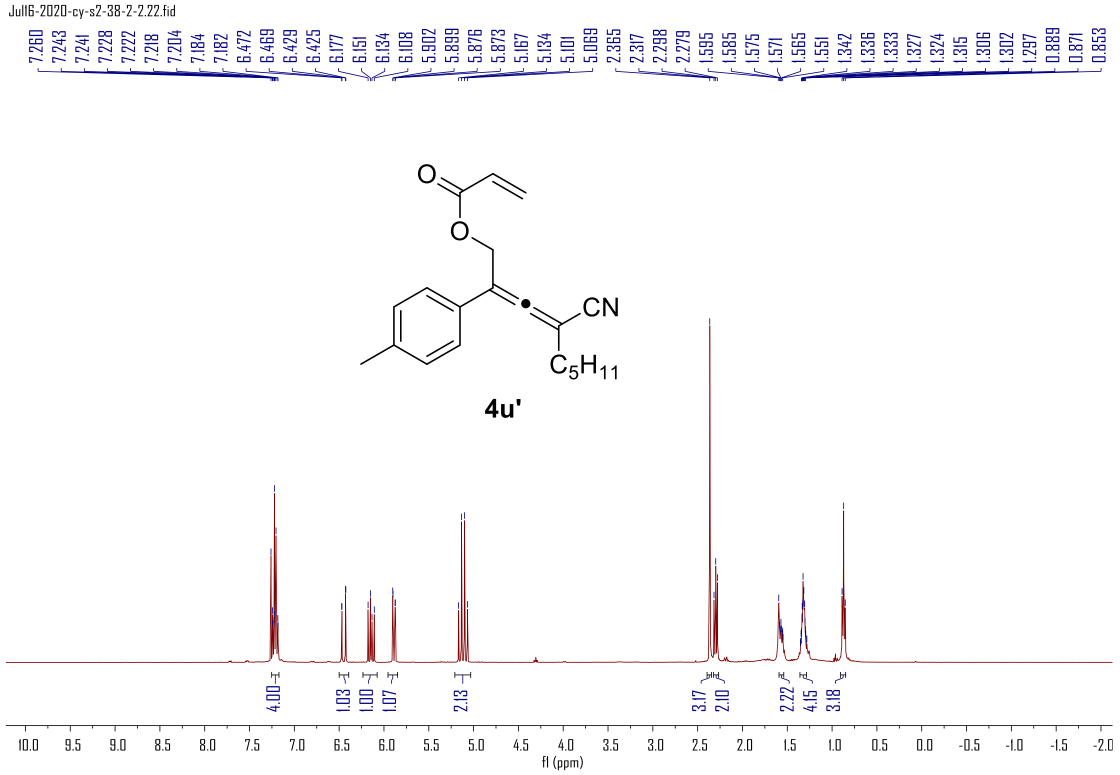
Jul16-2020-cy-s2-38-2-2.22.fid
4u'
fl (ppm)

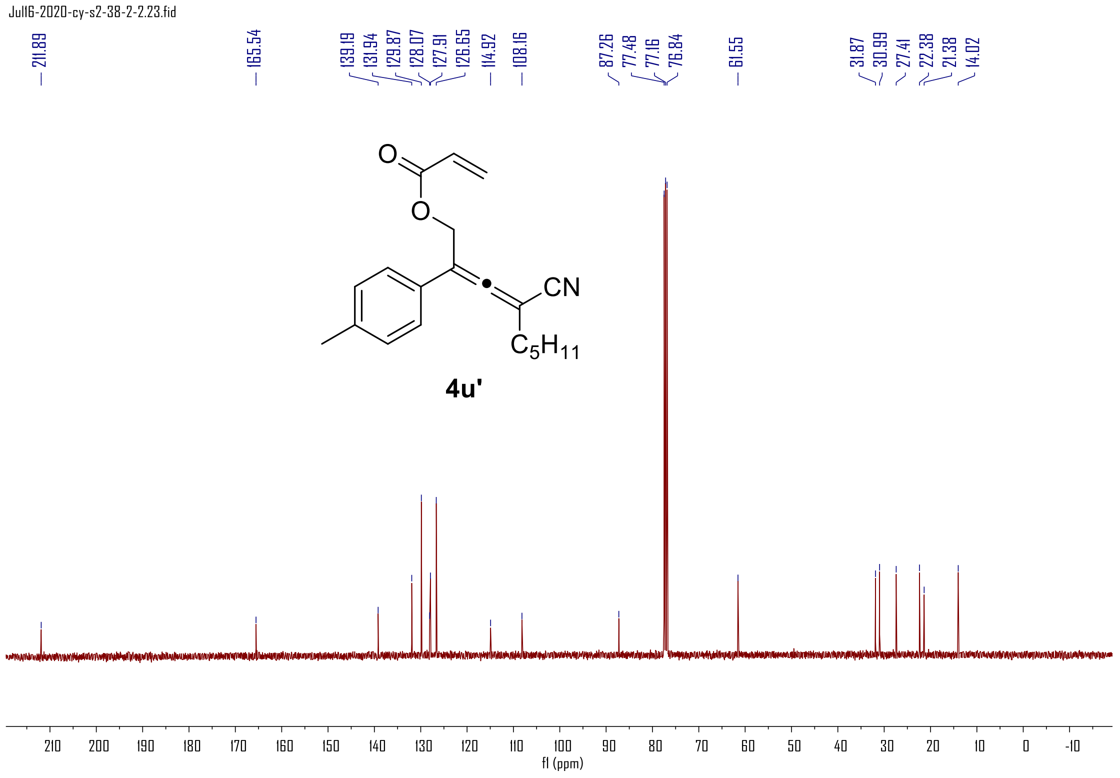
Jul16-2020-cy-s2-38-2-2.23.fid
4u'
fl (ppm)

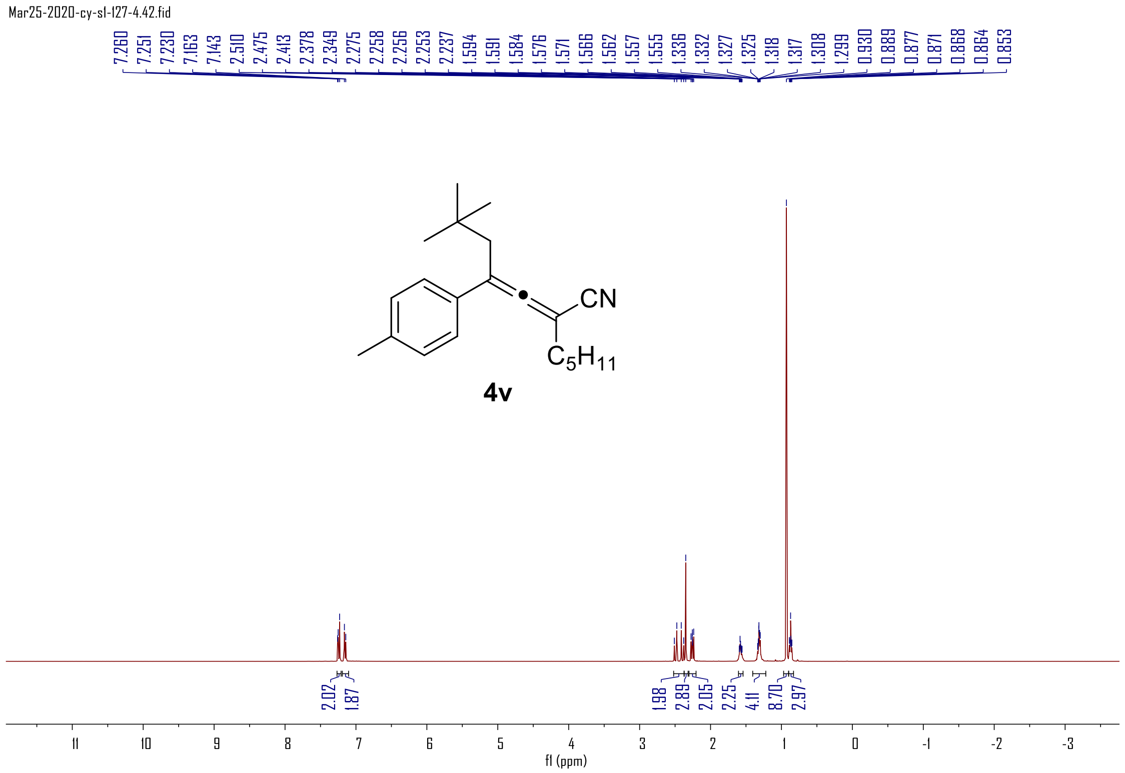
Mar25-2020-cy-sl-127-4.42.fid
7.260 7.251 7.230 7.163 7.143 2.510 2.475 2.413 2.378 2.349 2.275 2.258 2.256 2.253 2.237 1.594 1.591 1.584 1.576 1.571 1.566 1.562 1.557 1.555 1.336 1.332 1.327 1.325 1.318 1.317 1.308 1.299 0.930 0.889 0.877 0.871 0.868 0.864 0.853
CN
C_5H_{11}
4v
2.02 1.87 1.98 2.89 2.05 2.25 4.11 8.70 2.97
f1 (ppm)

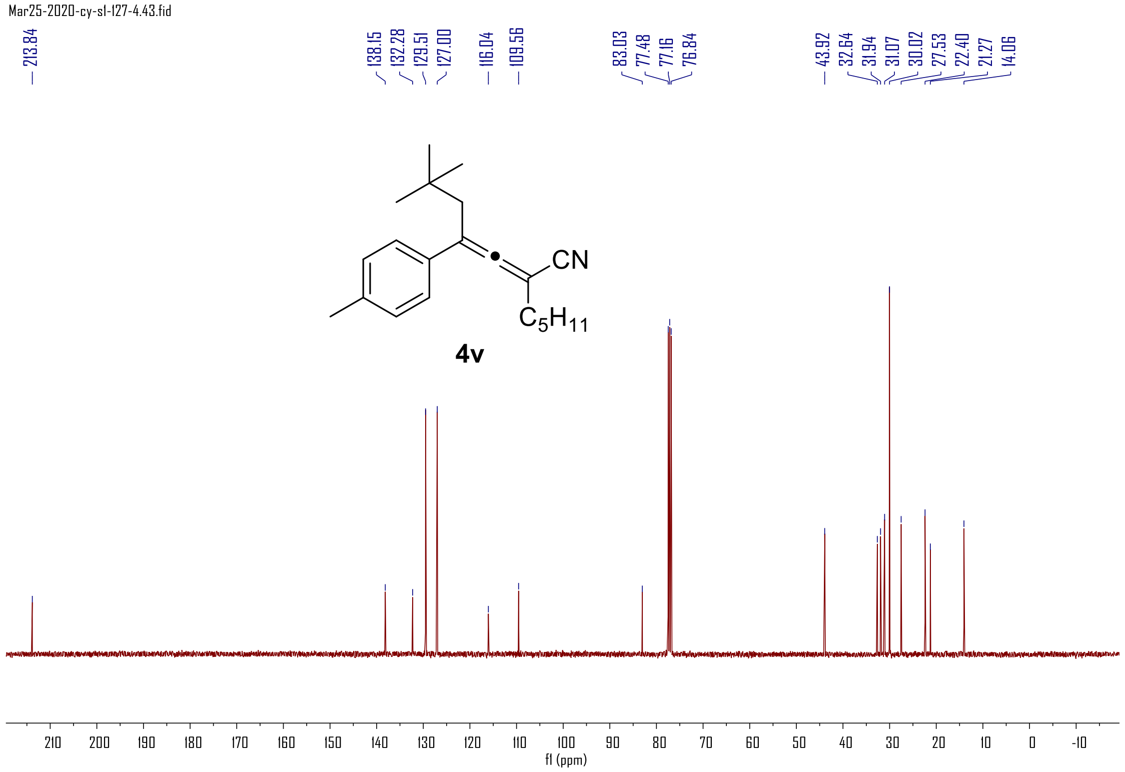
Mar25-2020-cy-sl-127-4.43.fid
213.84 138.15 132.28 129.51 127.00 116.04 109.56 83.03 77.48 77.16 76.84 43.92 32.64 31.94 31.07 30.02 27.53 22.40 21.27 14.06
CN
C_5H_{11}
4v
f1 (ppm)

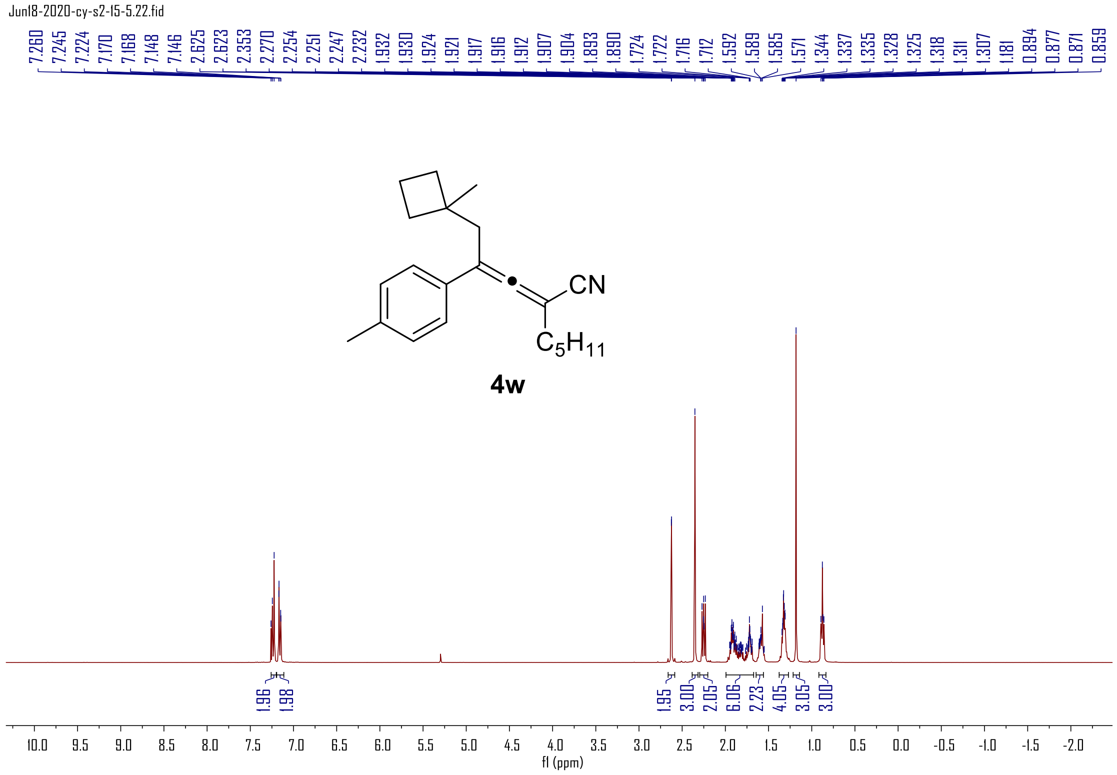
Jun18-2020-cy-s2-15-5.22.fid
7.260 7.245 7.224 7.170 7.168 7.148 7.146 2.625 2.623 2.353 2.270 2.254 2.251 2.247 2.232 1.932 1.930 1.924 1.921 1.917 1.916 1.912 1.907 1.904 1.893 1.890 1.724 1.722 1.716 1.712 1.592 1.589 1.585 1.571 1.344 1.337 1.335 1.328 1.325 1.318 1.311 1.307 1.181 0.894 0.877 0.871 0.859
CN
C_5H_{11}
4w
1.96 1.98 1.95 3.00 2.05 6.06 2.23 4.05 3.05 3.00
f1 (ppm)

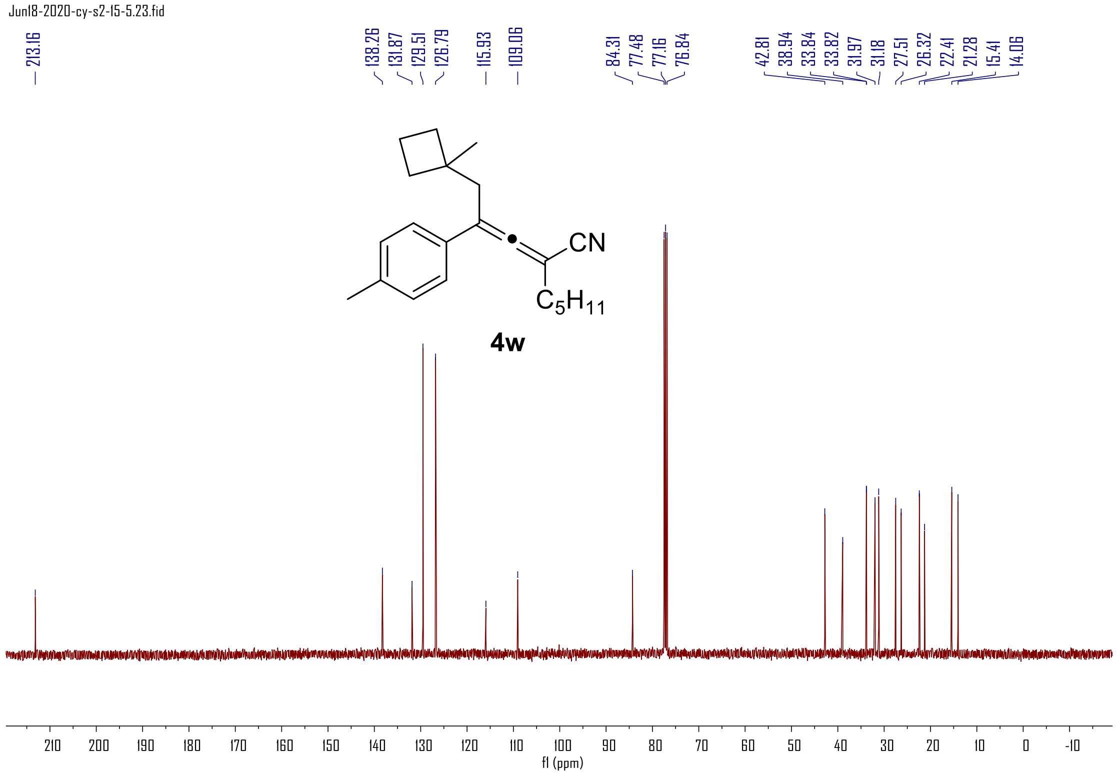
Jun18-2020-cy-s2-15-5.23.fid
213.16 138.26 131.87 129.51 126.79 115.93 109.06 84.31 77.48 77.16 76.84 42.81 38.94 33.84 33.82 31.97 31.18 27.51 26.32 22.41 21.28 15.41 14.06
CN
C_5H_{11}
4w
f1 (ppm)

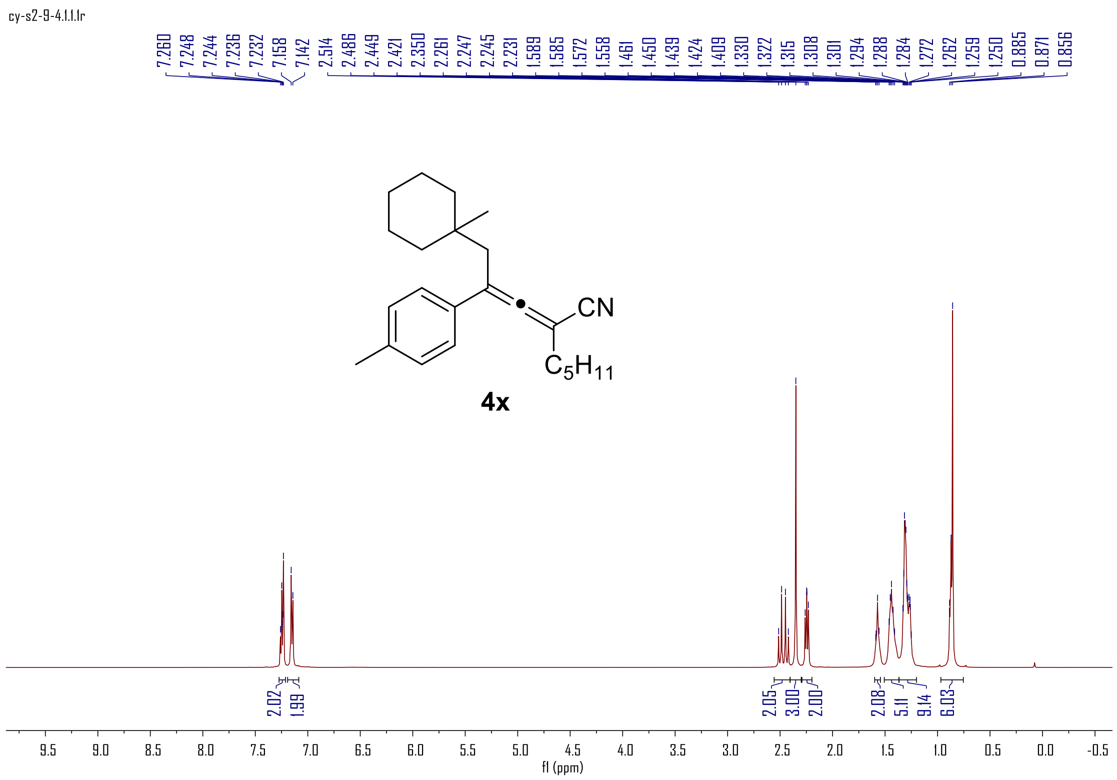
cy-s2-9-4.1.1.fr
7.260
7.248
7.244
7.236
7.232
7.158
7.142
2.514
2.486
2.449
2.421
2.350
2.261
2.247
2.245
2.231
1.589
1.585
1.572
1.558
1.461
1.450
1.439
1.424
1.409
1.330
1.322
1.315
1.308
1.301
1.294
1.288
1.284
1.272
1.262
1.259
1.250
0.885
0.871
0.856
CN
C_5H_{11}
4x
2.02
1.99
2.05
3.00
2.00
2.08
5.11
9.14
6.03
f1 (ppm)

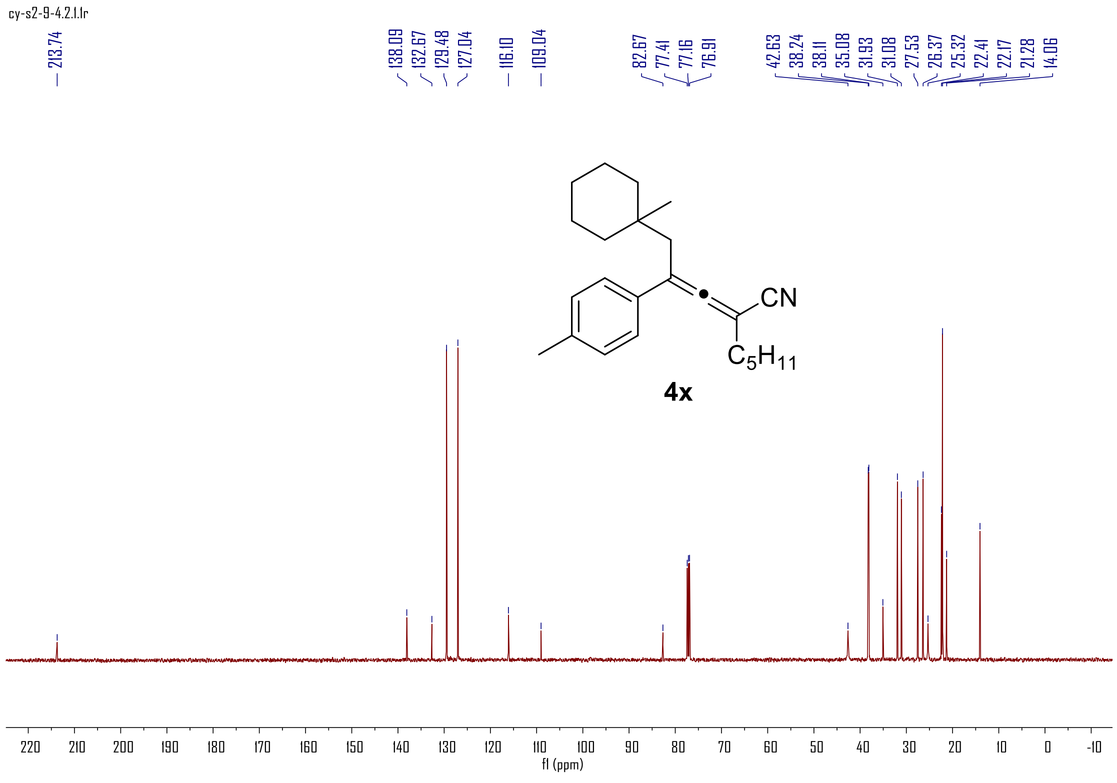
cy-s2-9-4.2.1.fr
213.74
138.09
132.67
129.48
127.04
116.10
109.04
82.67
77.41
77.16
76.91
42.63
38.24
38.11
35.08
31.93
31.08
27.53
26.37
25.32
22.41
22.17
21.28
14.06
CN
C_5H_{11}
4x
f1 (ppm)

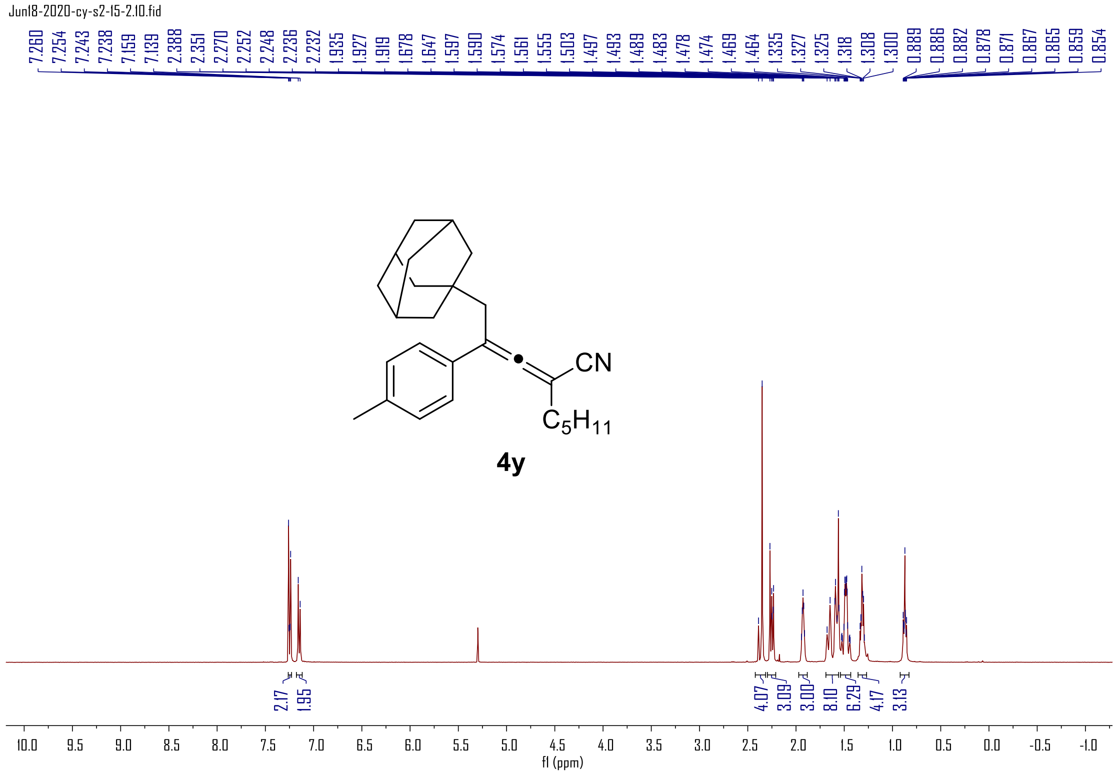
Jun18-2020-cy-s2-15-2.10.fid
7.260 7.254 7.243 7.238 7.159 7.139 2.388 2.351 2.270 2.252 2.248 2.236 2.232 1.935 1.927 1.919 1.678 1.647 1.597 1.590 1.574 1.561 1.555 1.503 1.497 1.493 1.489 1.483 1.478 1.474 1.469 1.464 1.335 1.327 1.325 1.318 1.308 1.300 0.889 0.886 0.882 0.878 0.871 0.867 0.865 0.859 0.854
CN
C_5H_{11}
4y
2.17 1.95 4.07 3.09 3.00 8.10 6.29 4.17 3.13
fl (ppm)

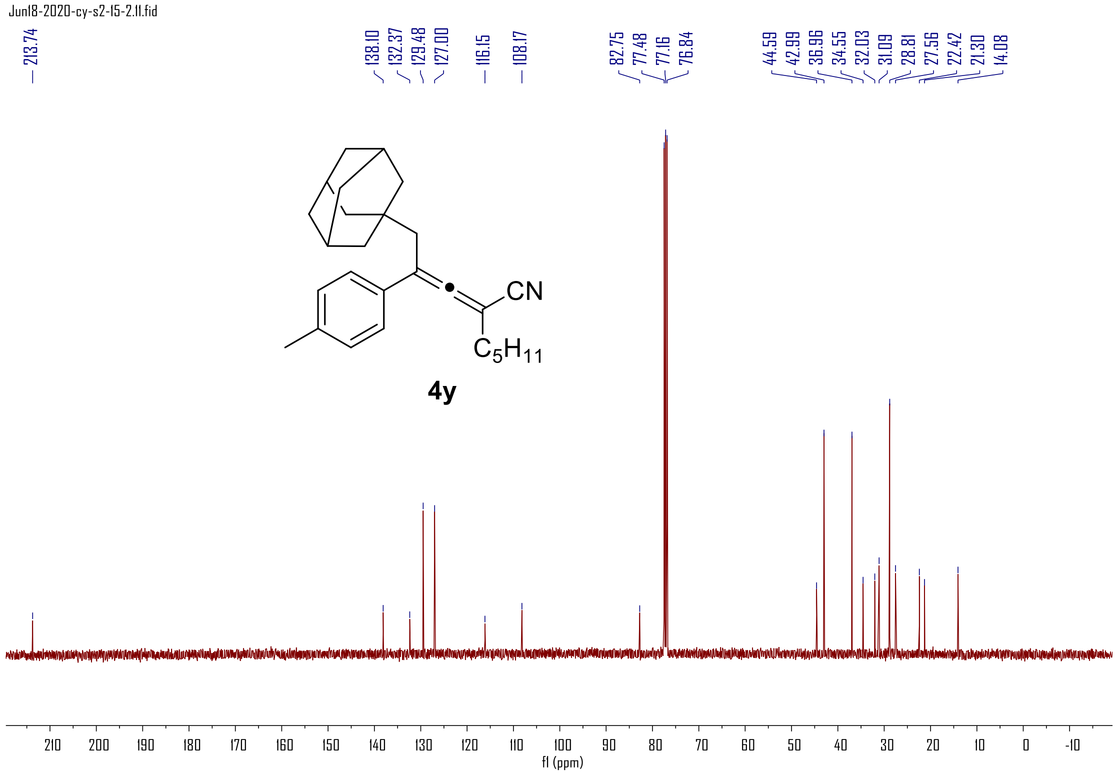
Jun18-2020-cy-s2-15-2.11.fid
213.74 138.10 132.37 129.48 127.00 116.15 108.17 82.75 77.48 77.16 76.84 44.59 42.99 36.96 34.55 32.03 31.09 28.81 27.56 22.42 21.30 14.08
CN
C_5H_{11}
4y
fl (ppm)

Jun20-2020-cy-s2-17-3.10.fid

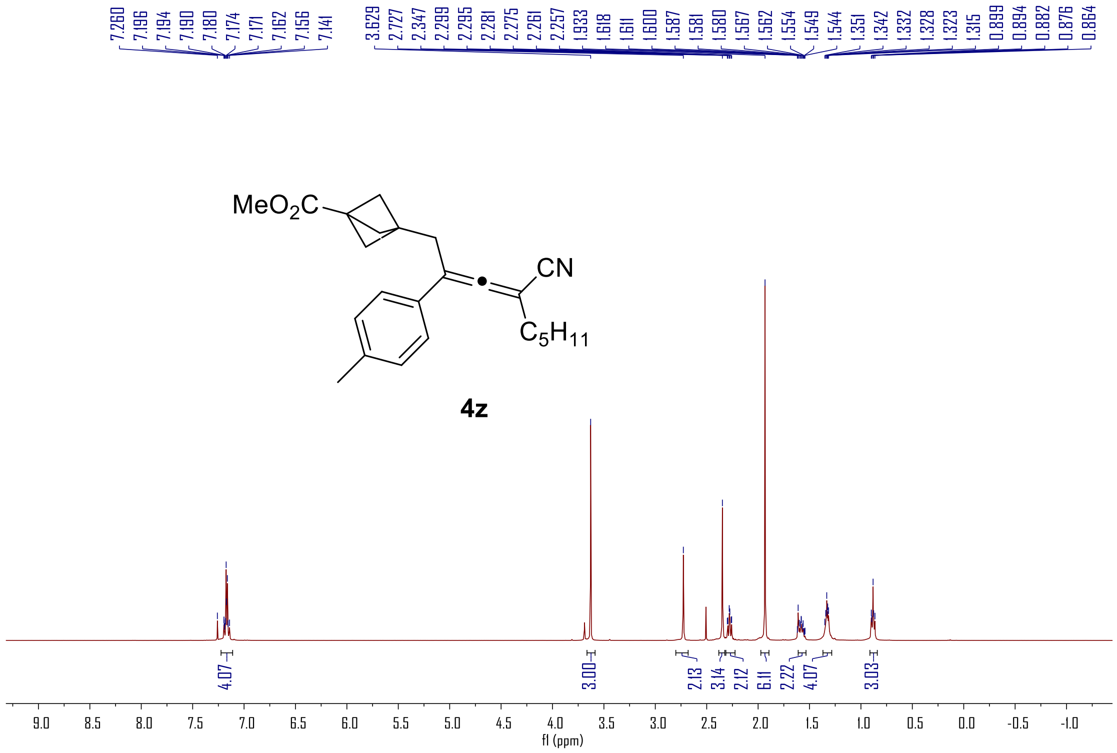

Jun20-2020-cy-s2-17-3.11.fid

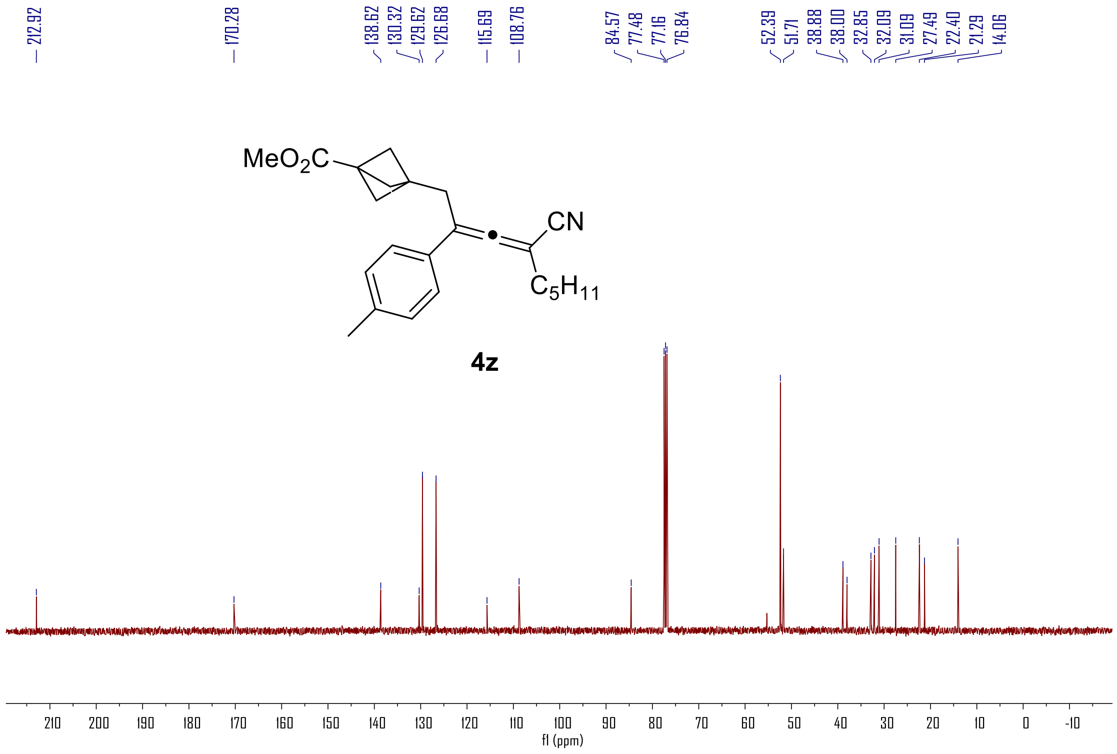

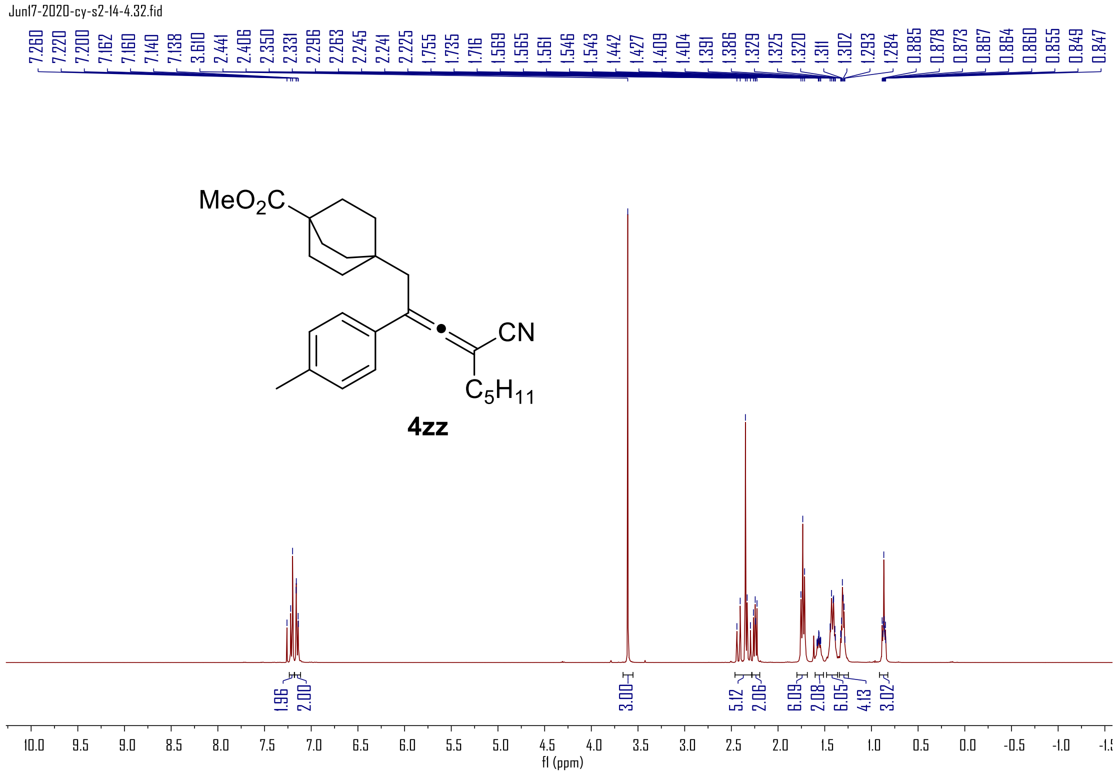
Jun17-2020-cy-s2-14-4.32.fid
7.260 7.220 7.200 7.162 7.160 7.140 7.138 3.610 2.441 2.406 2.350 2.331 2.296 2.263 2.245 2.241 2.225 1.755 1.735 1.716 1.569 1.565 1.561 1.546 1.543 1.442 1.427 1.409 1.404 1.391 1.386 1.329 1.325 1.320 1.311 1.302 1.293 1.284 0.885 0.878 0.873 0.867 0.864 0.860 0.855 0.849 0.847
MeO2C
CN
C5H11
4zz
1.96 2.00 3.00 5.12 2.06 6.09 2.08 6.05 4.13 3.02
f1 (ppm)

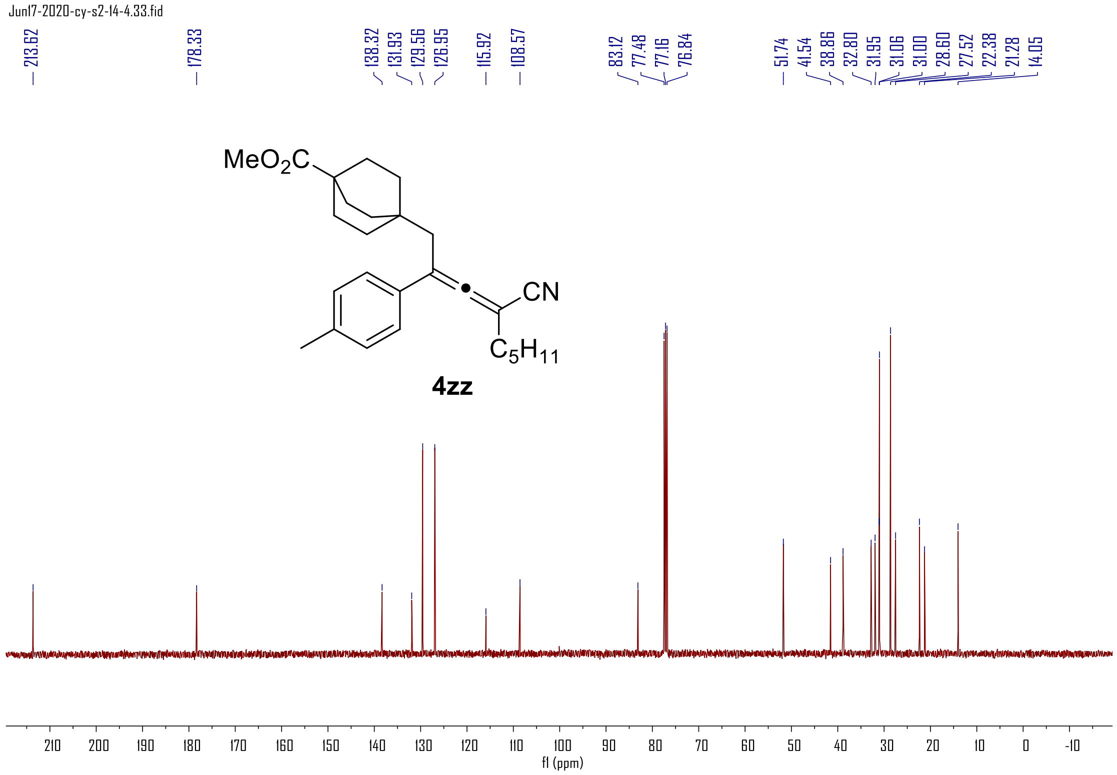
Jun17-2020-cy-s2-14-4.33.fid
213.62 178.33 138.32 131.93 129.56 126.95 115.92 108.57 83.12 77.48 77.16 76.84 51.74 41.54 38.86 32.80 31.95 31.06 31.00 28.60 27.52 22.38 21.28 14.05
MeO2C
CN
C5H11
4zz
f1 (ppm)

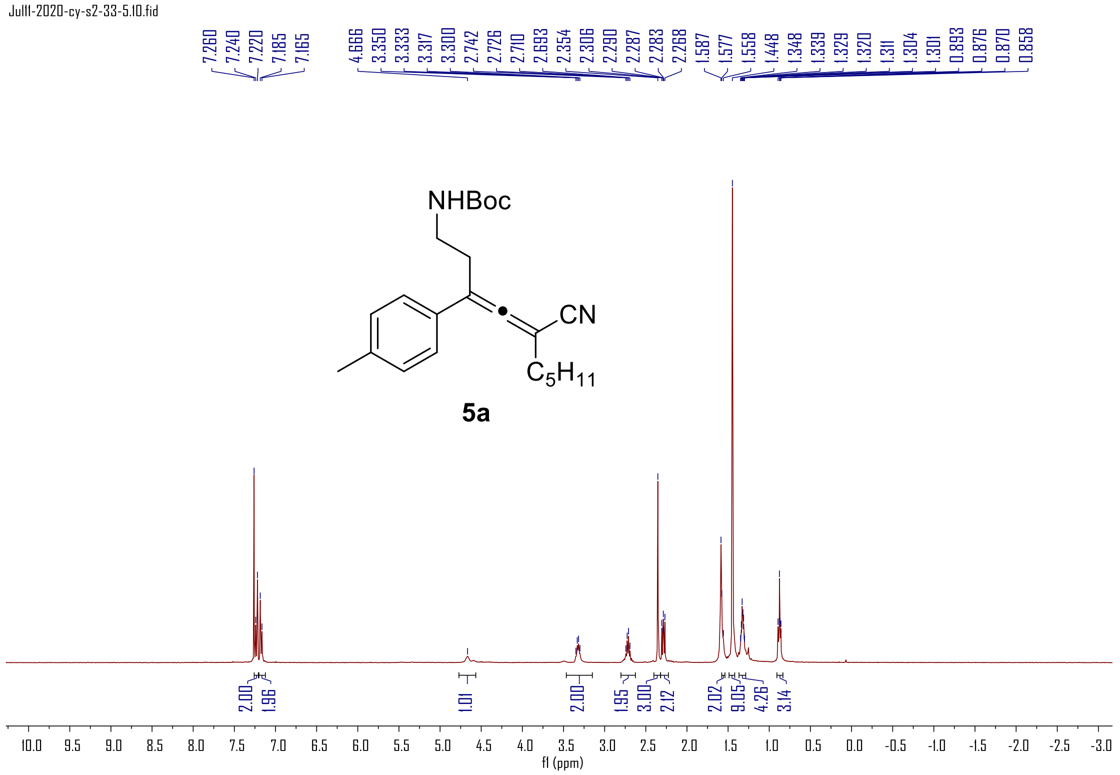

Jul11-2020-cy-s2-33-5.10.fid
7.260
7.240
7.220
7.185
7.165
4.666
3.350
3.333
3.317
3.300
2.742
2.726
2.710
2.693
2.354
2.306
2.290
2.287
2.283
2.268
1.587
1.577
1.558
1.448
1.348
1.339
1.329
1.320
1.311
1.304
1.301
0.893
0.876
0.870
0.858
NHBoc
CN
C5H11
5a
2.00
1.96
1.01
2.00
1.95
3.00
2.12
2.02
9.05
4.26
3.14
f1 (ppm)

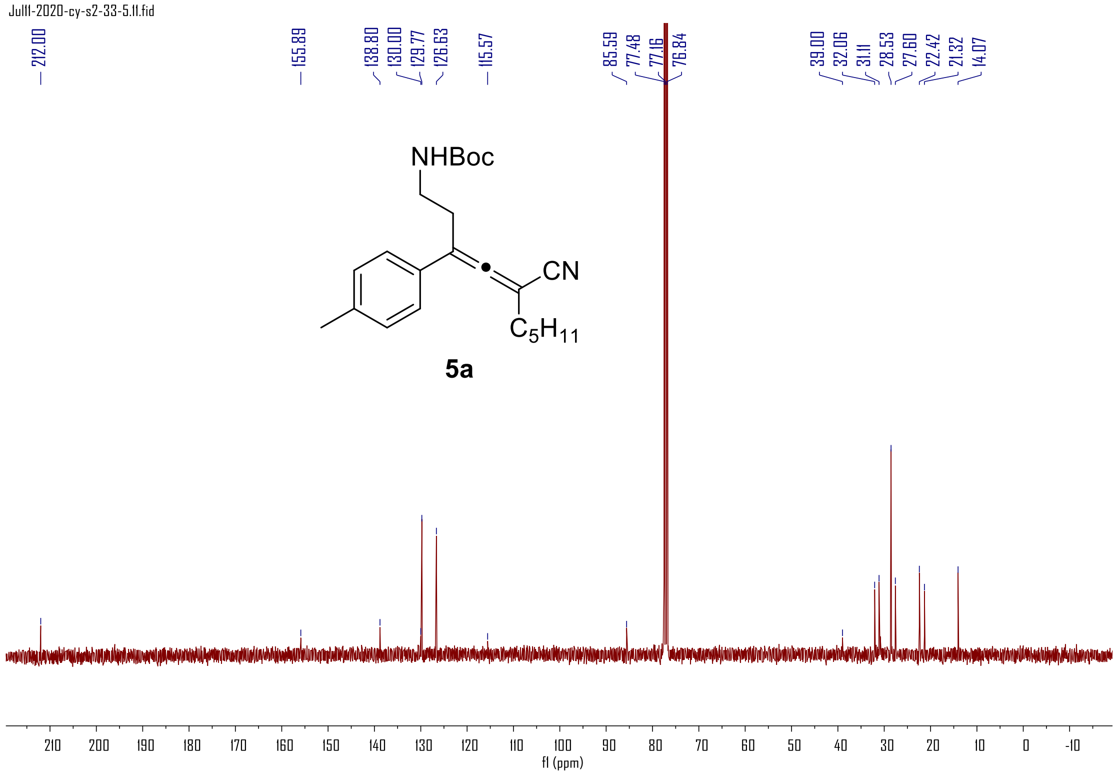

Jul11-2020-cy-s2-33-5.11.fid
212.00
155.89
138.80
130.00
129.77
126.63
115.57
85.59
77.48
77.16
76.84
39.00
32.06
31.11
28.53
27.60
22.42
21.32
14.07
NHBoc
CN
C5H11
5a
f1 (ppm)

Jun26-2020-cy-s2-24-2.12.fid

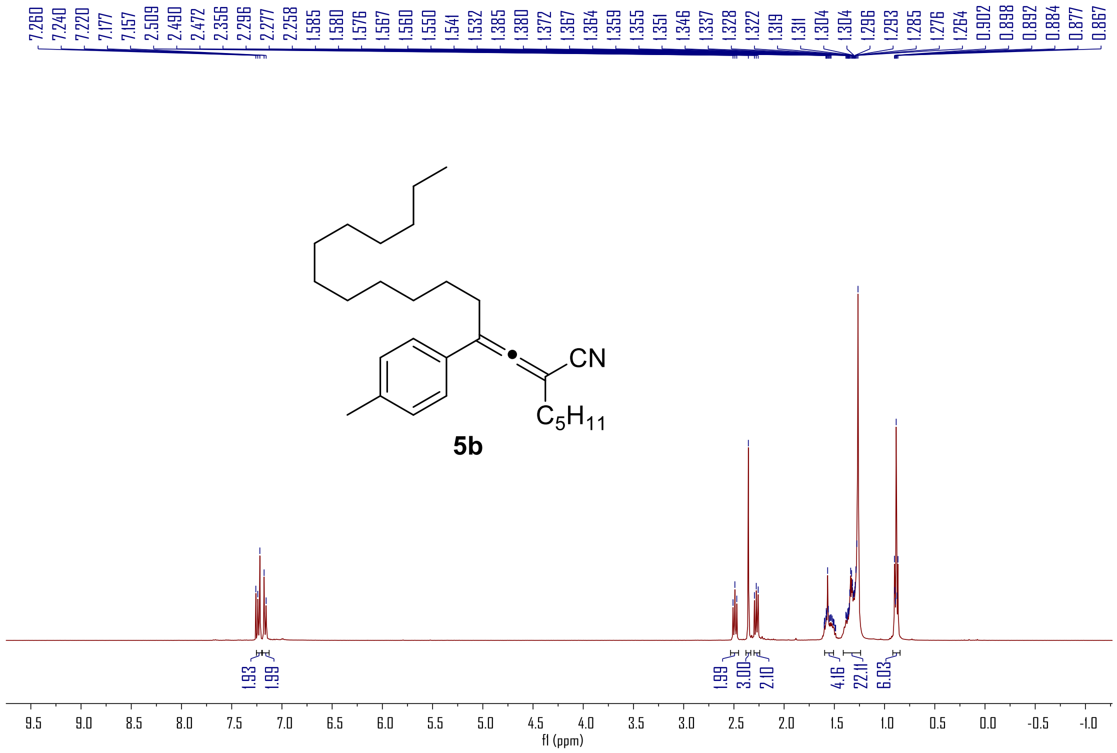

Jun26-2020-cy-s2-24-2.13.fid

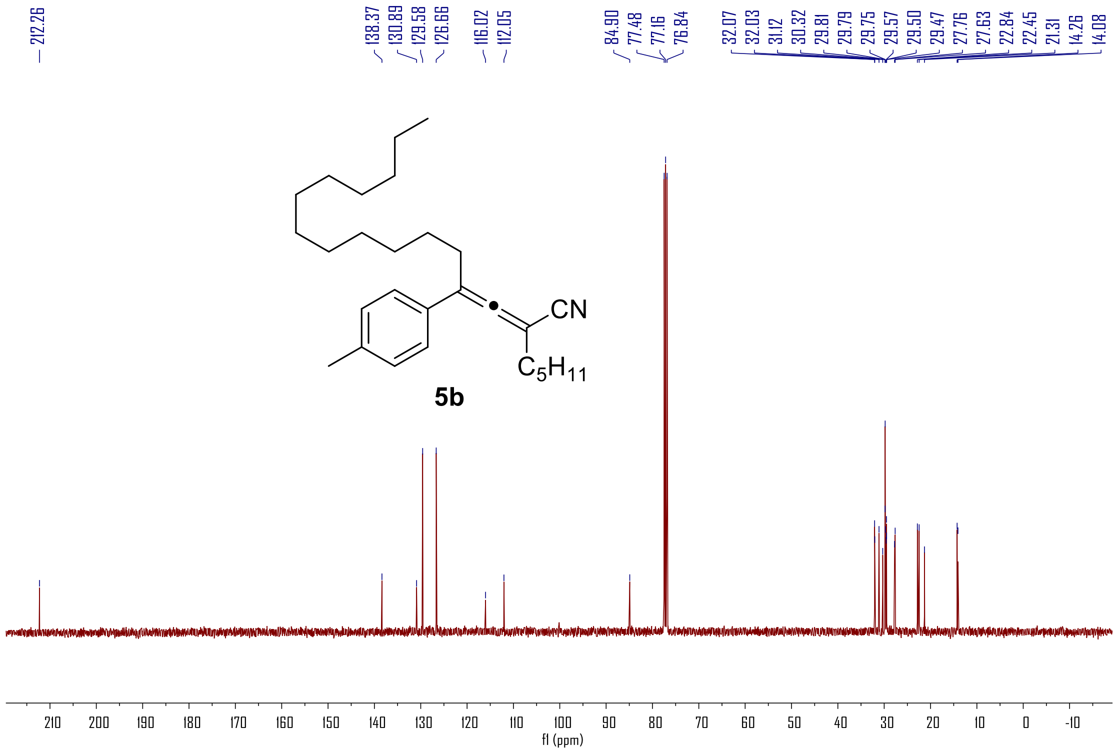

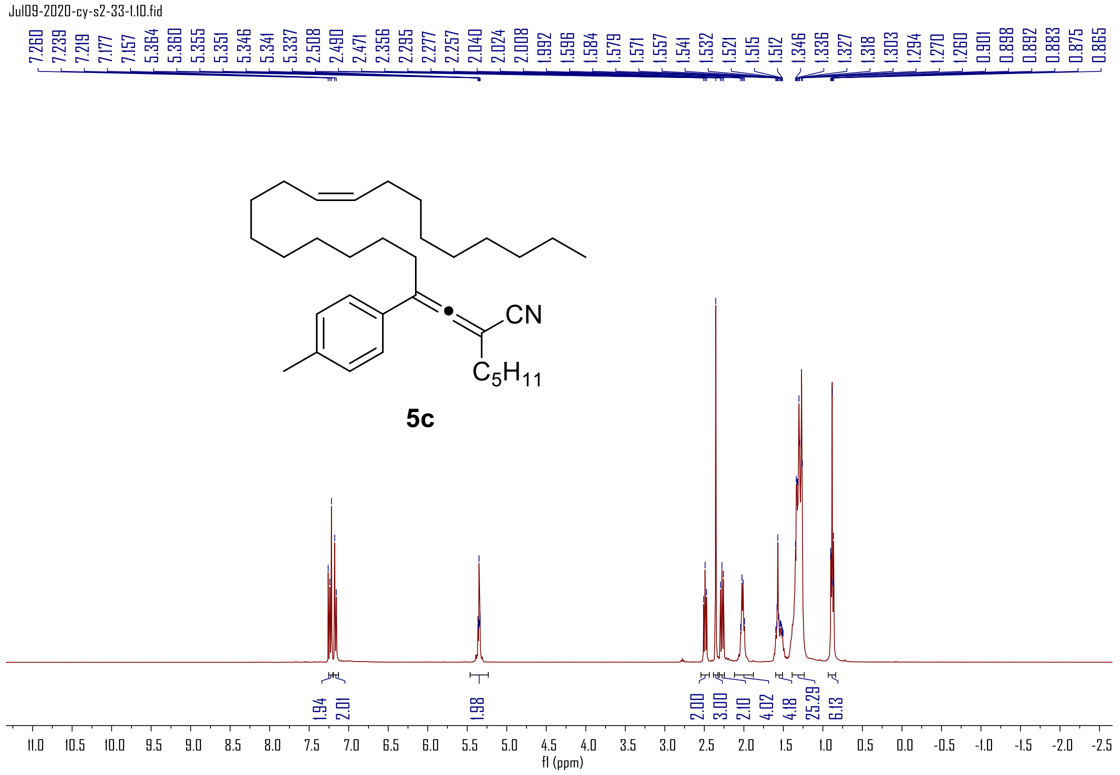

Jul09-2020-cy-s2-33-1.10.fid
7.260 7.239 7.219 7.177 7.157 5.364 5.360 5.355 5.351 5.346 5.341 5.337 2.508 2.490 2.471 2.356 2.295 2.277 2.257 2.040 2.024 2.008 1.992 1.596 1.584 1.579 1.571 1.557 1.541 1.532 1.521 1.515 1.512 1.346 1.336 1.327 1.318 1.303 1.294 1.270 1.260 0.901 0.898 0.892 0.883 0.875 0.865
CN
C_5H_{11}
5c
1.94 2.01 1.98 2.00 3.00 2.10 4.02 4.18 25.29 6.13
11.0 10.5 10.0 9.5 9.0 8.5 8.0 7.5 7.0 6.5 6.0 5.5 5.0 4.5 4.0 3.5 3.0 2.5 2.0 1.5 1.0 0.5 0.0 -0.5 -1.0 -1.5 -2.0 -2.5
f1 (ppm)

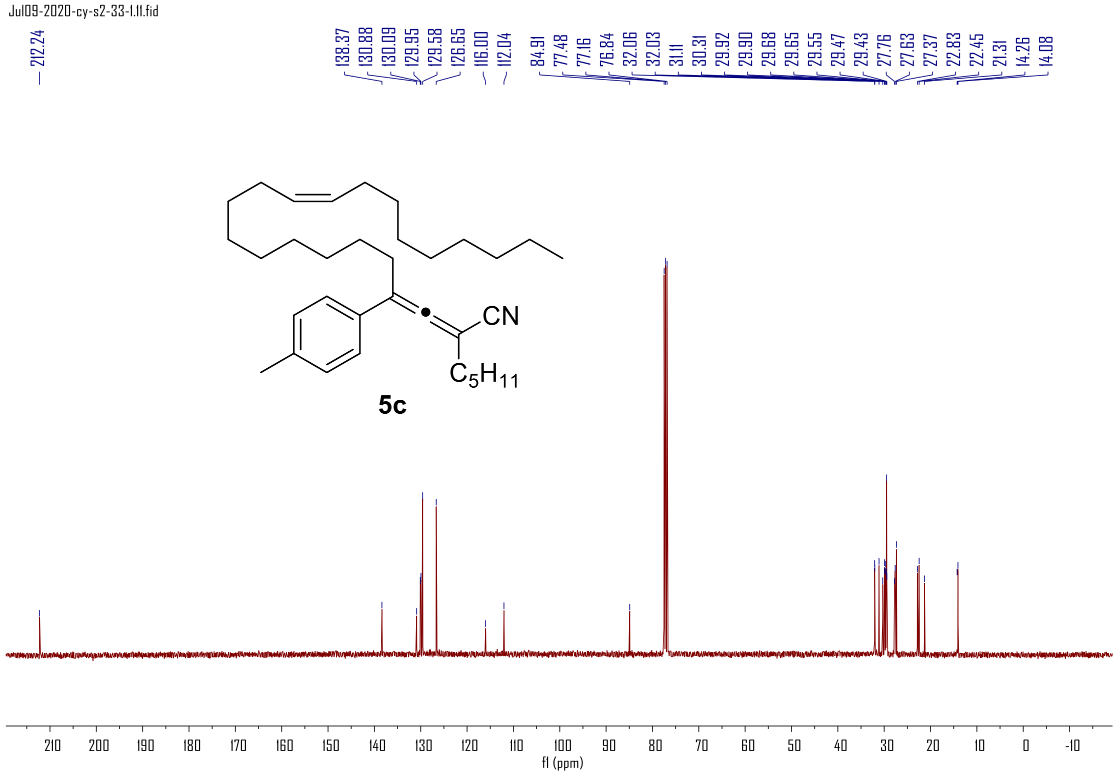

Jul09-2020-cy-s2-33-1.11.fid
212.24
138.37 130.88 130.09 129.95 129.58 126.65 116.00 112.04 84.91 77.48 77.16 76.84 32.06 32.03 31.11 30.31 29.92 29.90 29.68 29.65 29.55 29.47 29.43 27.76 27.63 27.37 22.83 22.45 21.31 14.26 14.08
CN
C_5H_{11}
5c
210 200 190 180 170 160 150 140 130 120 110 100 90 80 70 60 50 40 30 20 10 0 -10
f1 (ppm)

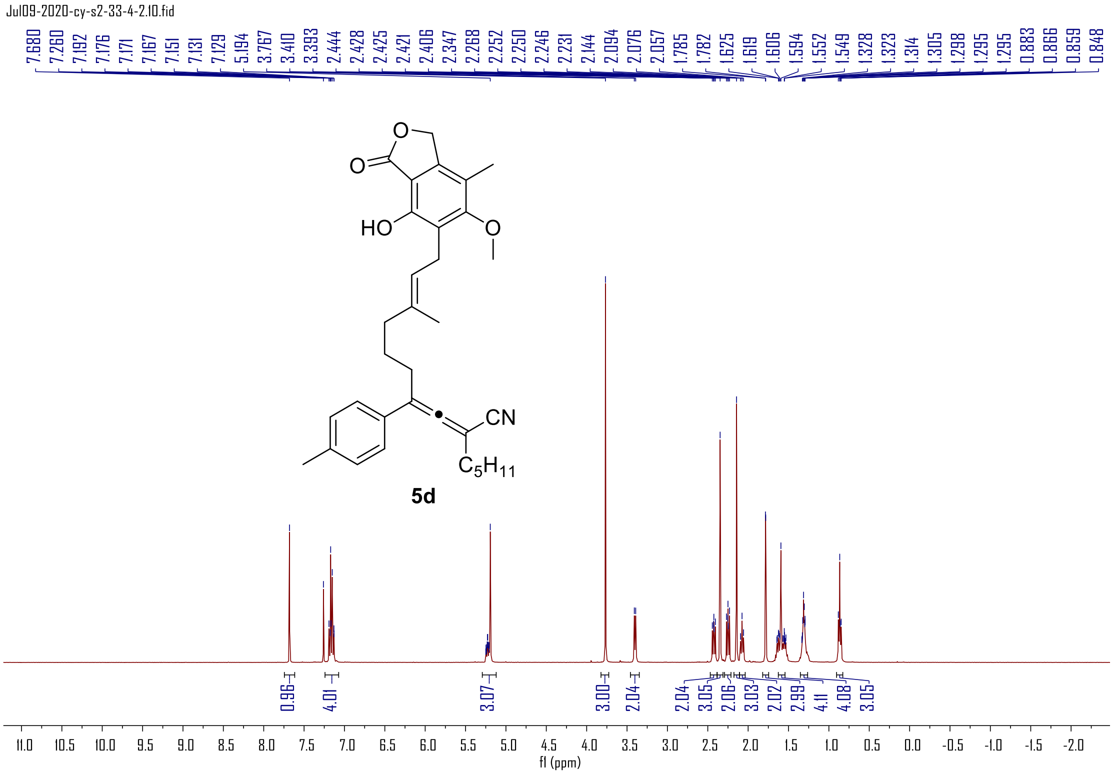
Jul09-2020-cy-s2-33-4-2.10.fid
7.680 7.260 7.192 7.176 7.171 7.167 7.151 7.131 7.129 5.194 3.767 3.410 3.393 2.444 2.428 2.425 2.421 2.406 2.347 2.268 2.252 2.250 2.246 2.231 2.144 2.094 2.076 2.057 1.785 1.782 1.625 1.619 1.606 1.594 1.552 1.549 1.328 1.323 1.314 1.305 1.298 1.295 1.295 0.883 0.866 0.859 0.848
O
O
HO
O
CN
C_5H_{11}
5d
0.96 4.01 3.07 3.00 2.04 2.04 3.05 2.06 3.03 2.02 2.99 4.11 4.08 3.05
11.0 10.5 10.0 9.5 9.0 8.5 8.0 7.5 7.0 6.5 6.0 5.5 5.0 4.5 4.0 3.5 3.0 2.5 2.0 1.5 1.0 0.5 0.0 -0.5 -1.0 -1.5 -2.0
f1 (ppm)

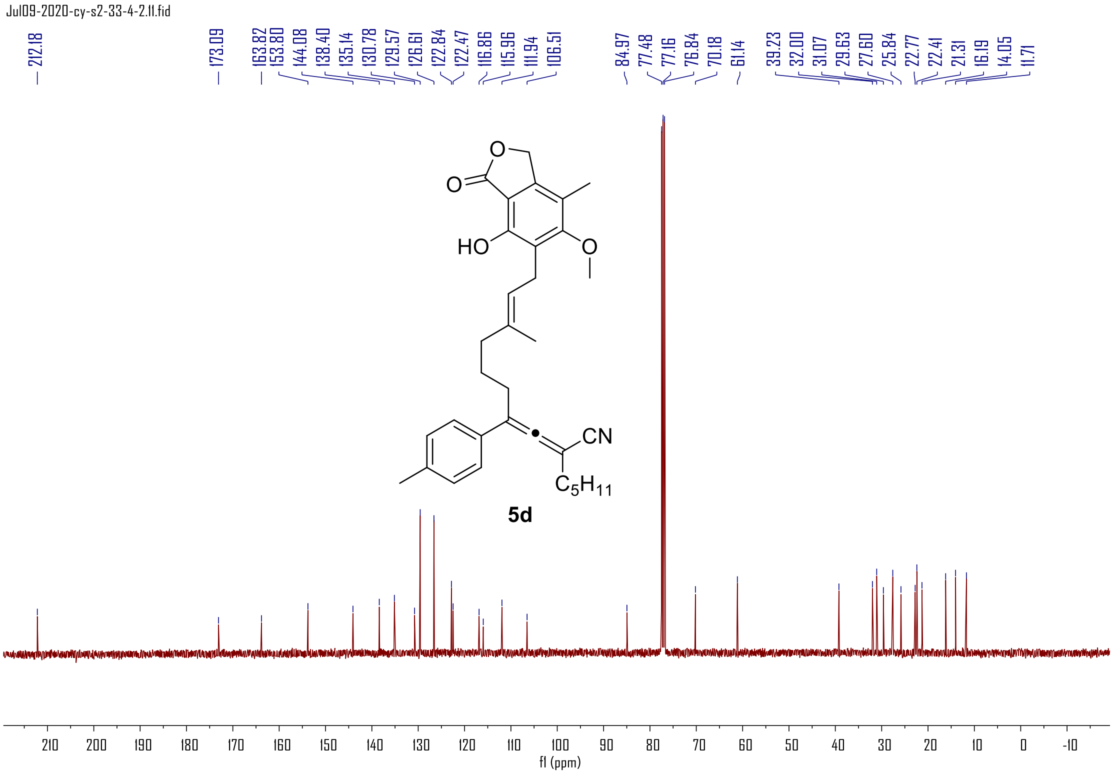
Jul09-2020-cy-s2-33-4-2.11.fid
212.18 173.09 163.82 153.80 144.08 138.40 135.14 130.78 129.57 126.61 122.84 122.47 116.86 115.96 111.94 106.51 84.97 77.48 77.16 76.84 70.18 61.14 39.23 32.00 31.07 29.63 27.60 25.84 22.77 22.41 21.31 16.19 14.05 11.71
O
O
HO
O
CN
C_5H_{11}
5d
210 200 190 180 170 160 150 140 130 120 110 100 90 80 70 60 50 40 30 20 10 0 -10
f1 (ppm)

cy-s2-33-2.1.1.fr

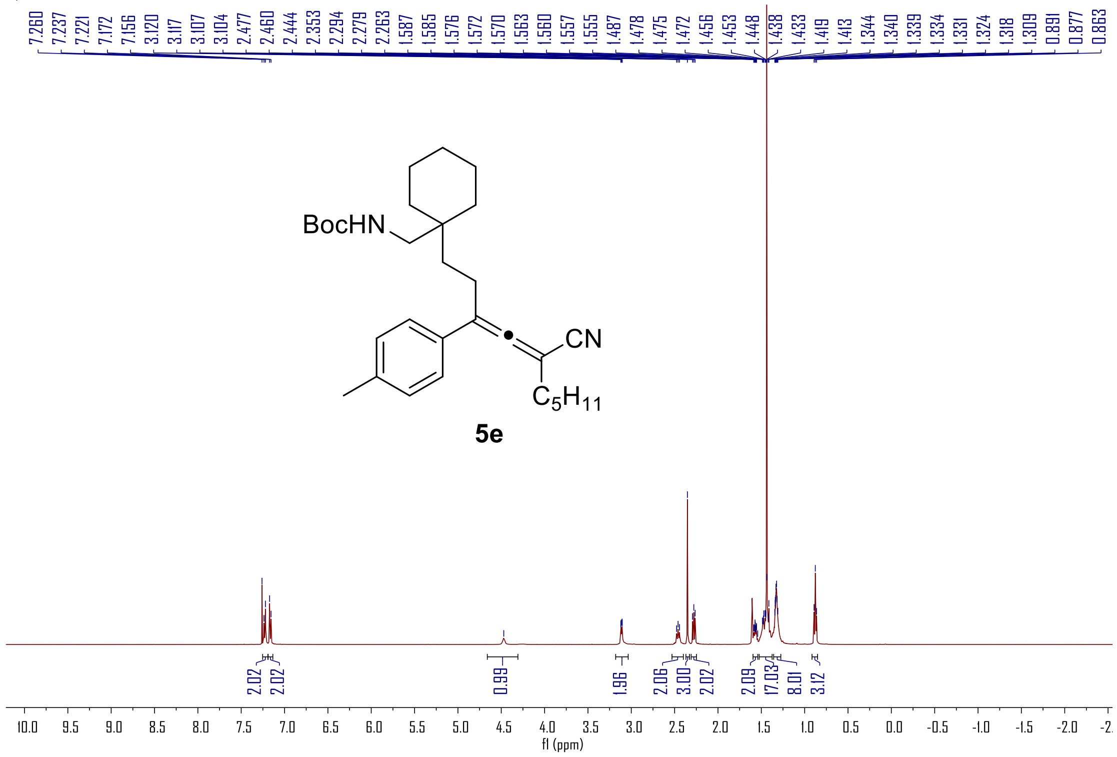

cy-s2-33-2.2.1.fr

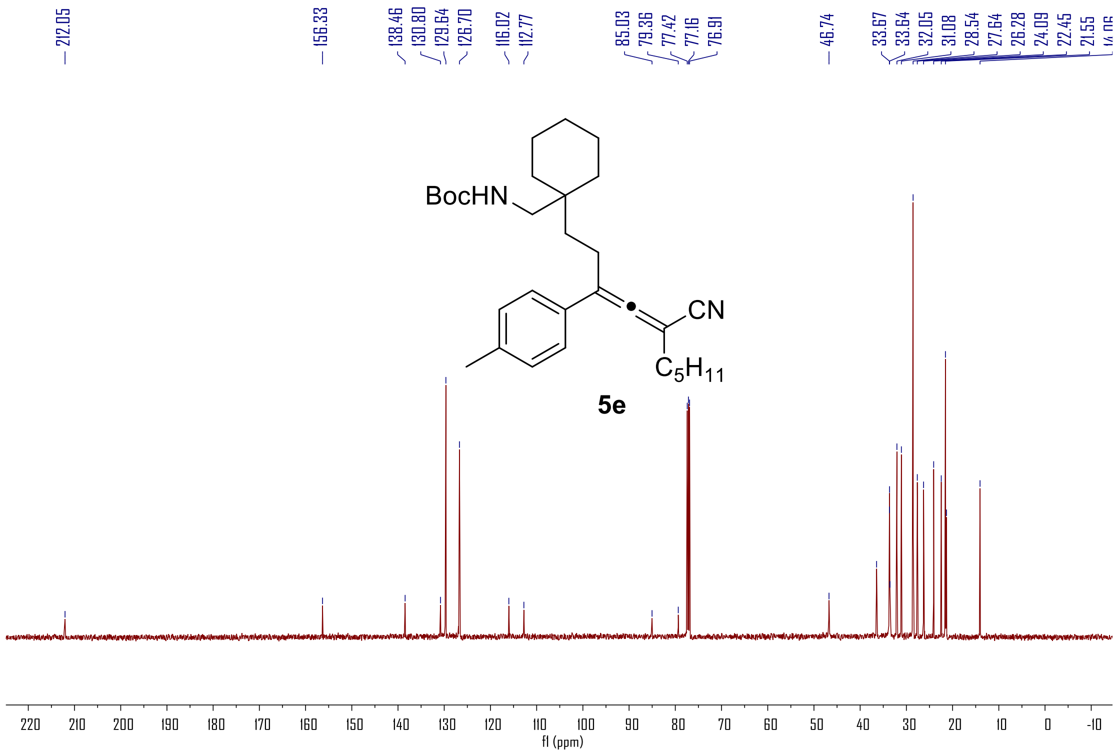

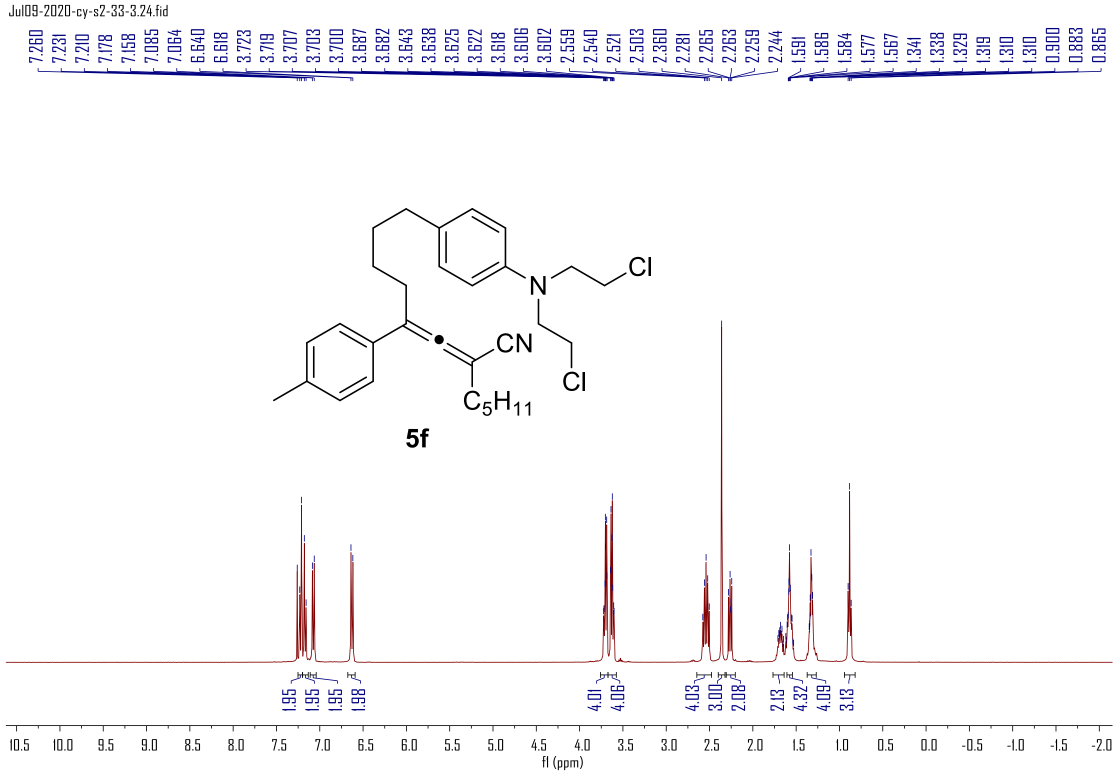

Jul09-2020-cy-s2-33-3.24.fid
7.260 7.231 7.210 7.178 7.158 7.085 7.064 6.640 6.618 3.723 3.719 3.707 3.703 3.700 3.687 3.682 3.643 3.638 3.625 3.622 3.618 3.606 3.602 2.559 2.540 2.521 2.503 2.360 2.281 2.265 2.263 2.259 2.244 1.591 1.586 1.584 1.577 1.567 1.341 1.338 1.329 1.319 1.310 1.310 0.900 0.883 0.865
CN
Cl
N
Cl
C_5H_{11}
5f
1.95 1.95 1.95 1.98 4.01 4.06 4.03 3.00 2.08 2.13 4.32 4.09 3.13
10.5 10.0 9.5 9.0 8.5 8.0 7.5 7.0 6.5 6.0 5.5 5.0 4.5 4.0 3.5 3.0 2.5 2.0 1.5 1.0 0.5 0.0 -0.5 -1.0 -1.5 -2.0
f1 (ppm)

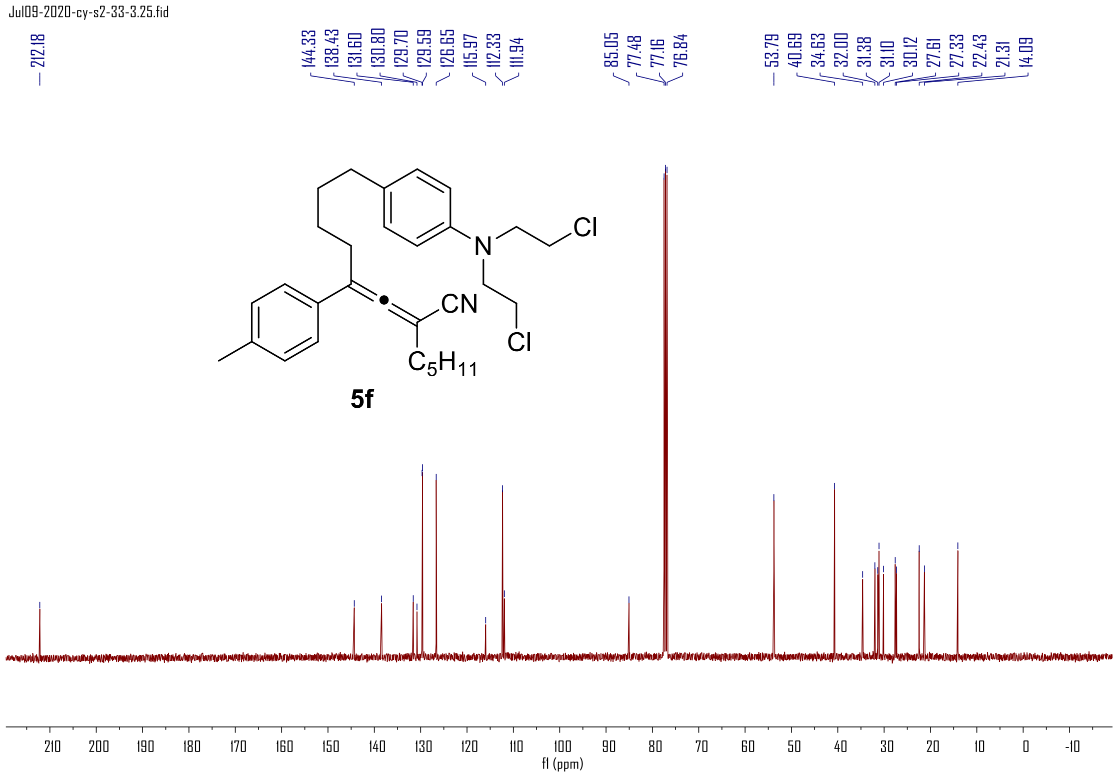

Jul09-2020-cy-s2-33-3.25.fid
212.18
144.33 138.43 131.60 130.80 129.70 129.59 126.65 115.97 112.33 111.94
85.05 77.48 77.16 76.84
53.79 40.69 34.63 32.00 31.38 31.10 30.12 27.61 27.33 22.43 21.31 14.09
CN
Cl
N
Cl
C_5H_{11}
5f
210 200 190 180 170 160 150 140 130 120 110 100 90 80 70 60 50 40 30 20 10 0 -10
f1 (ppm)

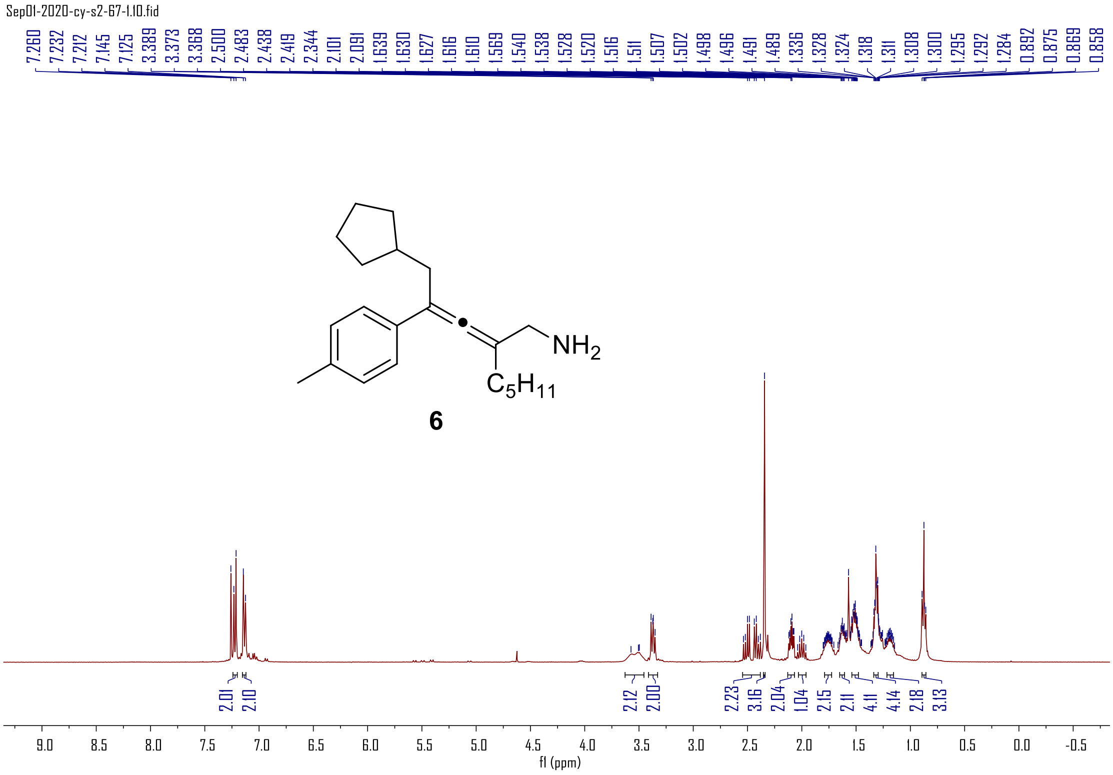
Sep01-2020-cy-s2-67-1.10.fid
7.260 7.232 7.212 7.145 7.125 3.389 3.373 3.368 2.500 2.483 2.438 2.419 2.344 2.101 2.091 1.639 1.630 1.627 1.616 1.610 1.569 1.540 1.538 1.528 1.520 1.516 1.511 1.507 1.502 1.498 1.496 1.491 1.489 1.336 1.328 1.324 1.318 1.311 1.308 1.300 1.295 1.292 1.284 0.892 0.875 0.869 0.858
NH2
C5H11
6
2.01 2.10 2.12 2.00 2.23 3.16 2.04 1.04 2.15 2.11 4.11 4.14 2.18 3.13
9.0 8.5 8.0 7.5 7.0 6.5 6.0 5.5 5.0 4.5 4.0 3.5 3.0 2.5 2.0 1.5 1.0 0.5 0.0 -0.5
f1 (ppm)

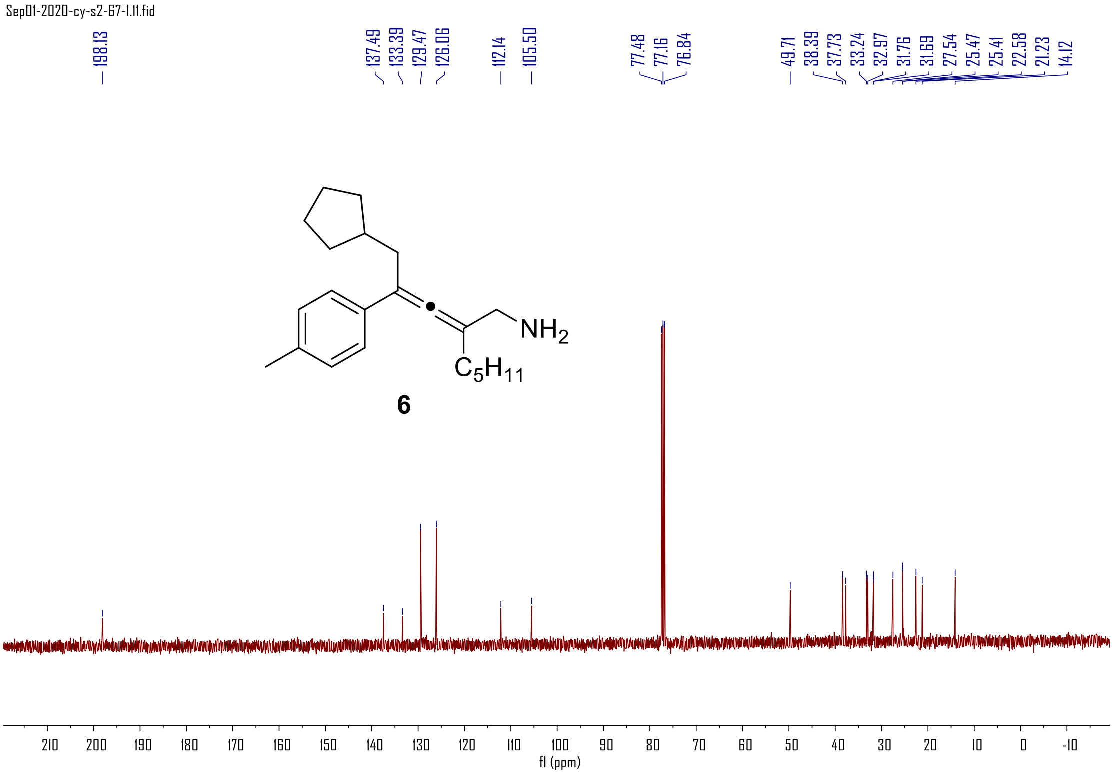
Sep01-2020-cy-s2-67-1.11.fid
198.13
137.49 133.39 129.47 126.06
112.14 105.50
77.48 77.16 76.84
49.71 38.39 37.73 33.24 32.97 31.76 31.69 27.54 25.47 25.41 22.58 21.23 14.12
NH2
C5H11
6
210 200 190 180 170 160 150 140 130 120 110 100 90 80 70 60 50 40 30 20 10 0 -10
f1 (ppm)

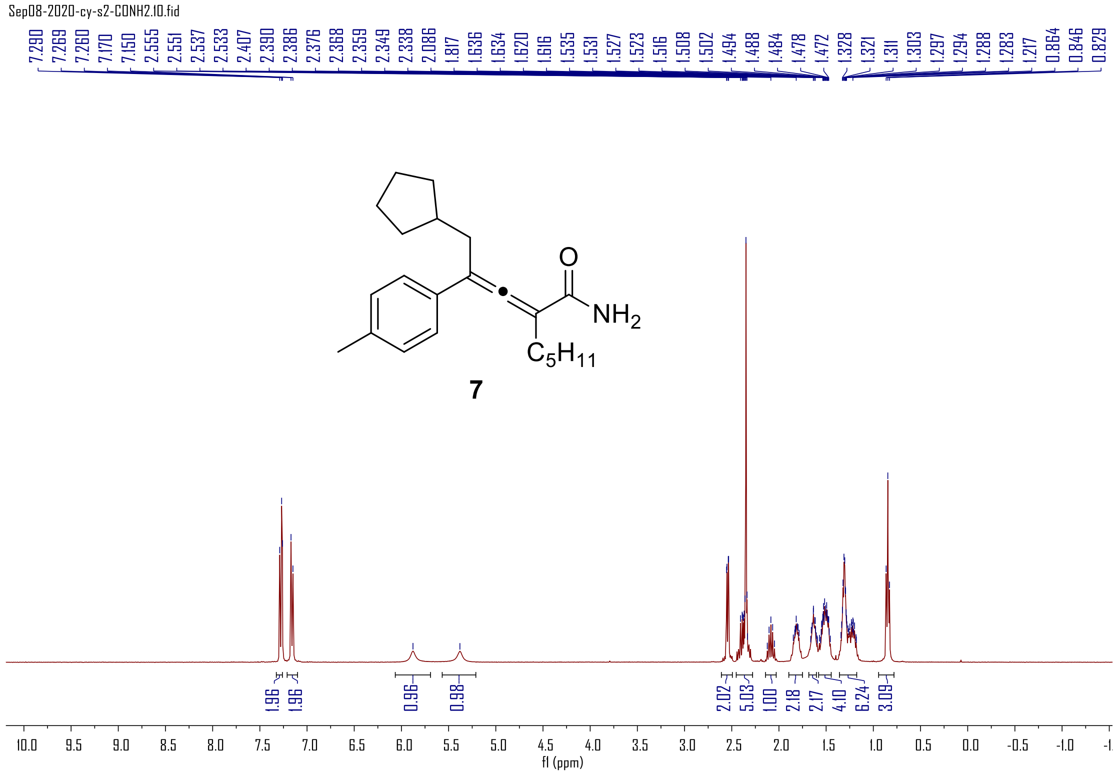
Sep08-2020-cy-s2-CONH2.10.fid
7.290 7.269 7.260 7.170 7.150 2.555 2.551 2.537 2.533 2.407 2.390 2.386 2.376 2.368 2.359 2.349 2.338 2.086 1.817 1.636 1.634 1.620 1.616 1.535 1.531 1.527 1.523 1.516 1.508 1.502 1.494 1.488 1.484 1.478 1.472 1.328 1.321 1.311 1.303 1.297 1.294 1.288 1.283 1.217 0.864 0.846 0.829
O
NH2
C5H11
7
1.96 1.96 0.96 0.98 2.02 5.03 1.00 2.18 2.17 4.10 6.24 3.09
10.0 9.5 9.0 8.5 8.0 7.5 7.0 6.5 6.0 5.5 5.0 4.5 4.0 3.5 3.0 2.5 2.0 1.5 1.0 0.5 0.0 -0.5 -1.0
f1 (ppm)

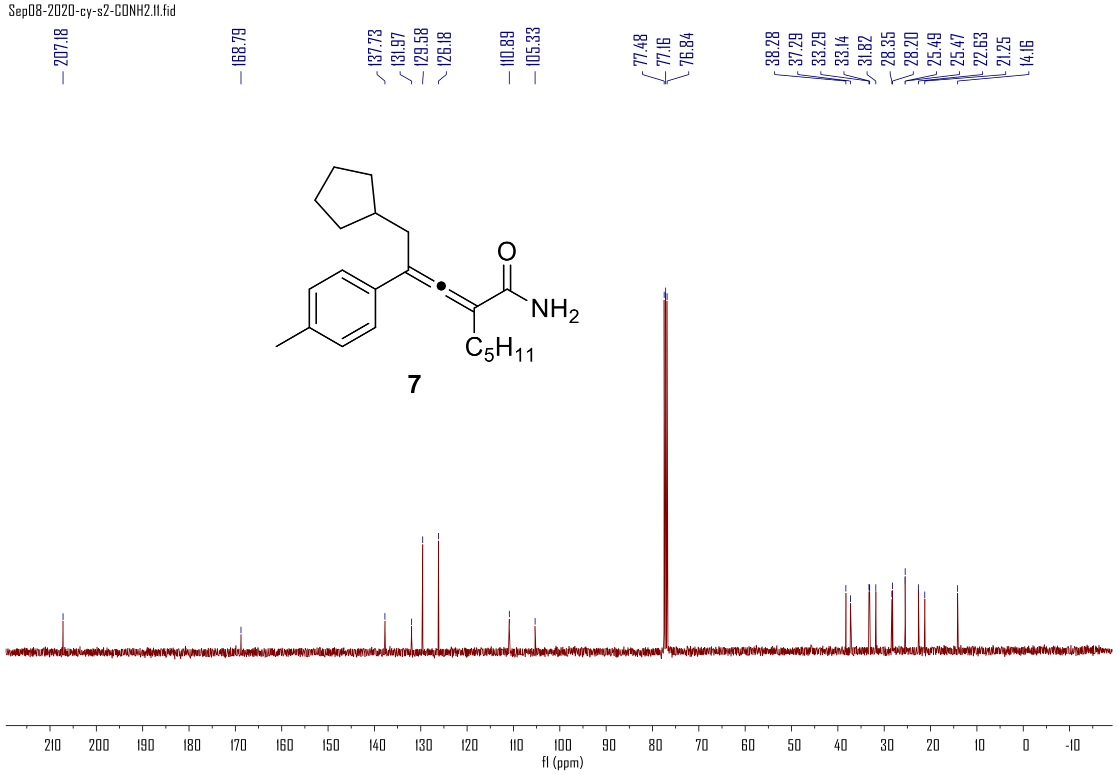
Sep08-2020-cy-s2-CONH2.11.fid
207.18 168.79 137.73 131.97 129.58 126.18 110.89 105.33 77.48 77.16 76.84 38.28 37.29 33.29 33.14 31.82 28.35 28.20 25.49 25.47 22.63 21.25 14.16
O
NH2
C5H11
7
210 200 190 180 170 160 150 140 130 120 110 100 90 80 70 60 50 40 30 20 10 0 -10
f1 (ppm)

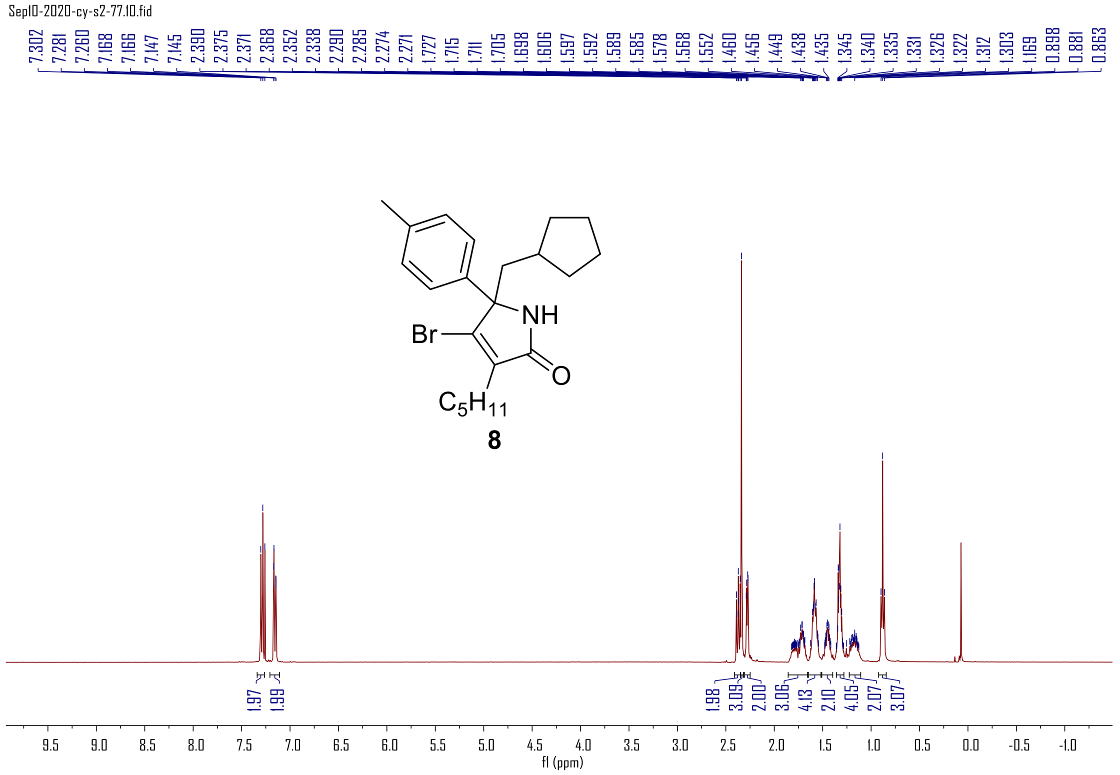
Sep10-2020-cy-s2-77.10.fid
7.302 7.281 7.260 7.168 7.166 7.147 7.145 2.390 2.375 2.371 2.368 2.352 2.338 2.290 2.285 2.274 2.271 1.727 1.715 1.711 1.705 1.698 1.606 1.597 1.592 1.589 1.585 1.578 1.568 1.552 1.460 1.456 1.449 1.438 1.435 1.345 1.340 1.335 1.331 1.326 1.322 1.312 1.303 1.169 0.898 0.881 0.863
Br
NH
O
C_5H_{11}
8
1.97 1.99
1.98 3.09 2.00 3.06 4.13 2.10 4.05 2.07 3.07
9.5 9.0 8.5 8.0 7.5 7.0 6.5 6.0 5.5 5.0 4.5 4.0 3.5 3.0 2.5 2.0 1.5 1.0 0.5 0.0 -0.5 -1.0
f1 (ppm)

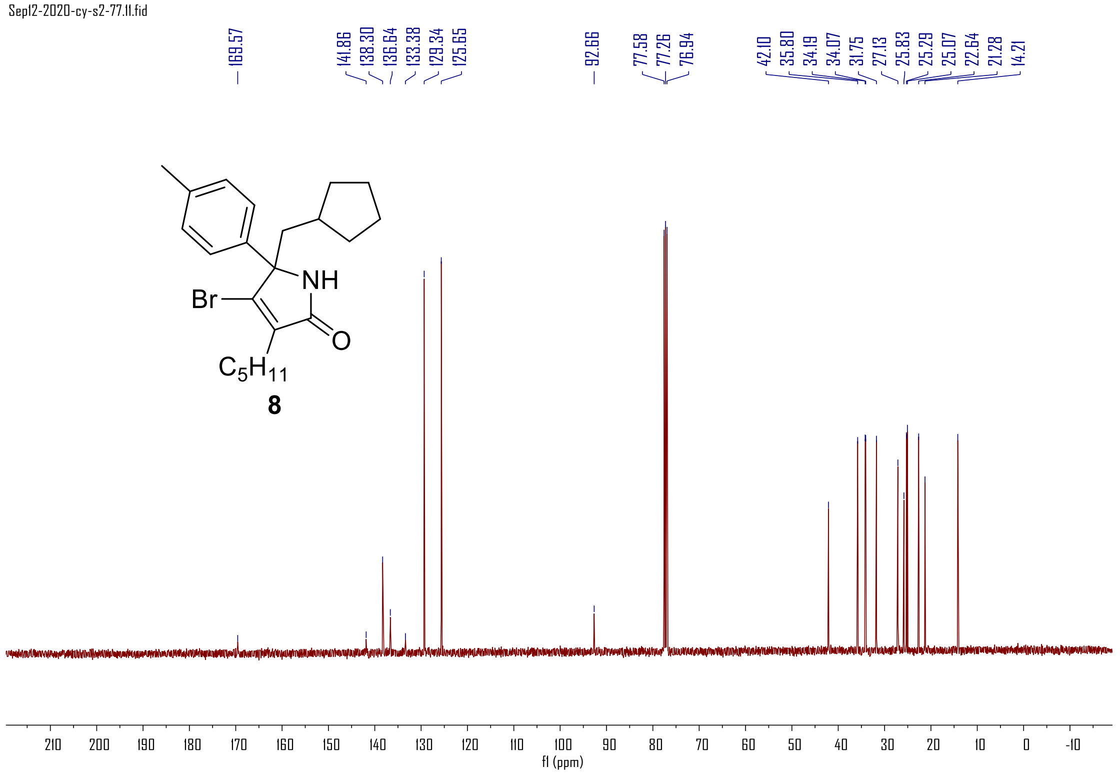
Sep12-2020-cy-s2-77.11.fid
169.57
141.86 138.30 136.64 133.38 129.34 125.65
92.66
77.58 77.26 76.94
42.10 35.80 34.19 34.07 31.75 27.13 25.83 25.29 25.07 22.64 21.28 14.21
Br
NH
O
C_5H_{11}
8
210 200 190 180 170 160 150 140 130 120 110 100 90 80 70 60 50 40 30 20 10 0 -10
f1 (ppm)

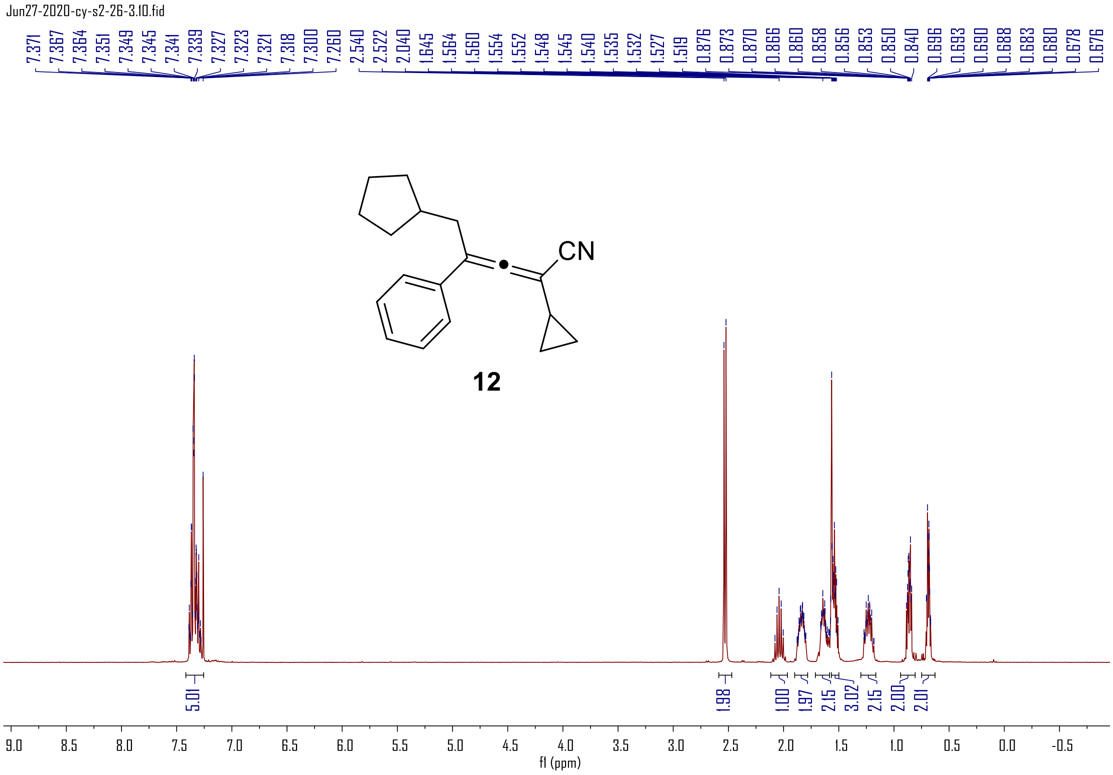
Jun27-2020-cy-s2-26-3.10.fid
7.371 7.367 7.364 7.351 7.349 7.345 7.341 7.339 7.327 7.323 7.321 7.318 7.300 7.260 2.540 2.522 2.040 1.645 1.564 1.560 1.554 1.552 1.548 1.545 1.540 1.535 1.532 1.527 1.519 0.876 0.873 0.870 0.866 0.860 0.858 0.856 0.853 0.850 0.840 0.696 0.693 0.690 0.688 0.683 0.680 0.678 0.676
CN
12
5.01
1.98
1.00
1.97
2.15
3.02
2.15
2.00
2.01
9.0 8.5 8.0 7.5 7.0 6.5 6.0 5.5 5.0 4.5 4.0 3.5 3.0 2.5 2.0 1.5 1.0 0.5 0.0 -0.5
f1 (ppm)

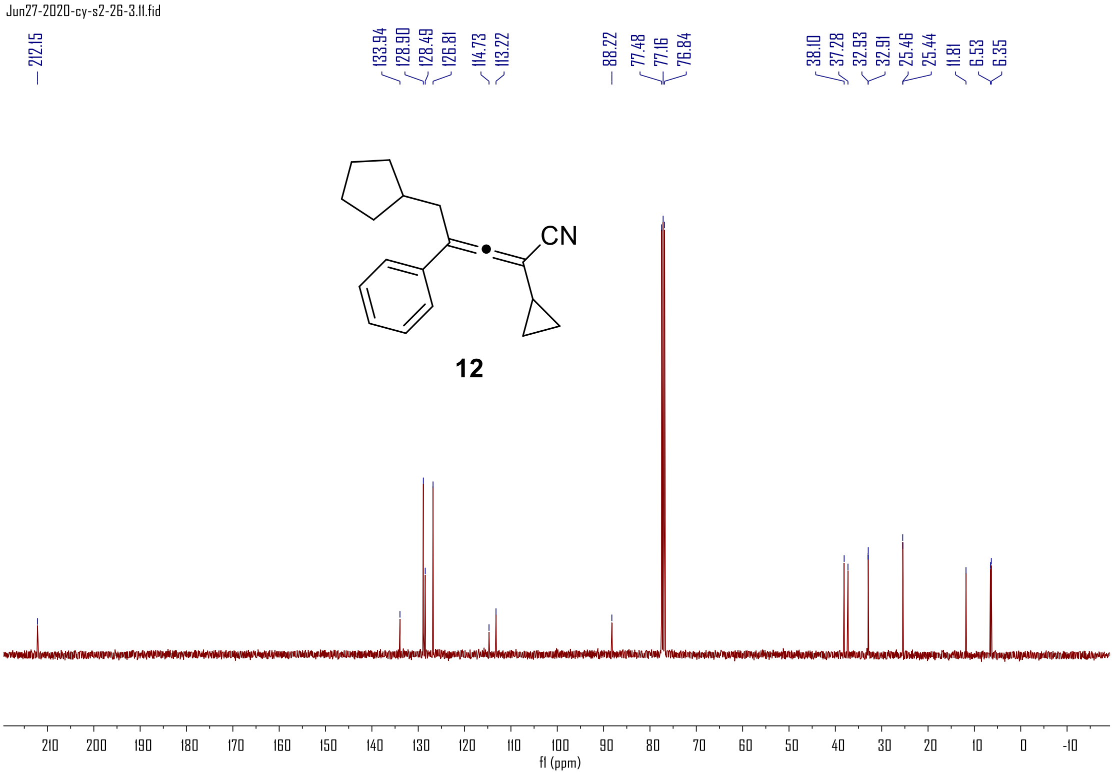
Jun27-2020-cy-s2-26-3.11.fid
212.15
133.94 128.90 128.49 126.81 114.73 113.22
88.22 77.48 77.16 76.84
38.10 37.28 32.93 32.91 25.46 25.44 11.81 6.53 6.35
CN
12
210 200 190 180 170 160 150 140 130 120 110 100 90 80 70 60 50 40 30 20 10 0 -10
f1 (ppm)

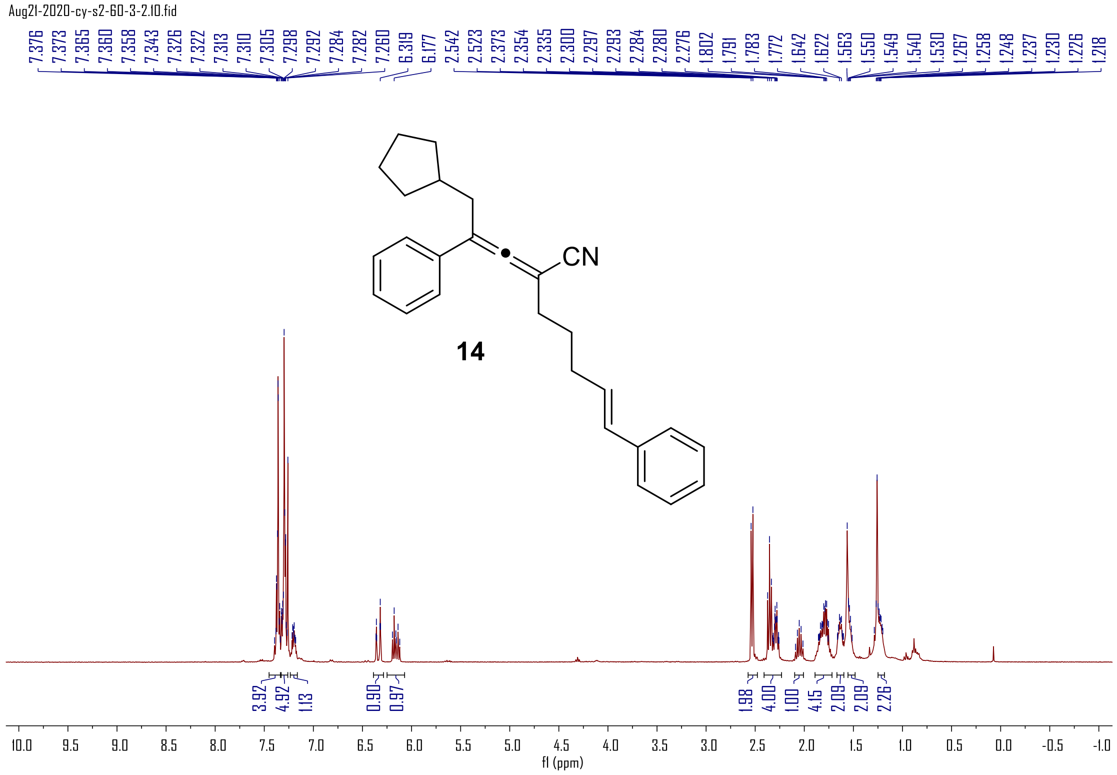
Aug21-2020-cy-s2-60-3-2.10.fid
7.376 7.373 7.365 7.360 7.358 7.343 7.326 7.322 7.313 7.310 7.305 7.298 7.292 7.284 7.282 7.260 6.319 6.177 2.542 2.523 2.373 2.354 2.335 2.300 2.297 2.293 2.284 2.280 2.276 1.802 1.791 1.783 1.772 1.642 1.622 1.563 1.550 1.549 1.540 1.530 1.267 1.258 1.248 1.237 1.230 1.226 1.218
CN
14
3.92 4.92 1.13 0.90 0.97 1.98 4.00 1.00 4.15 2.09 2.09 2.26
f1 (ppm)

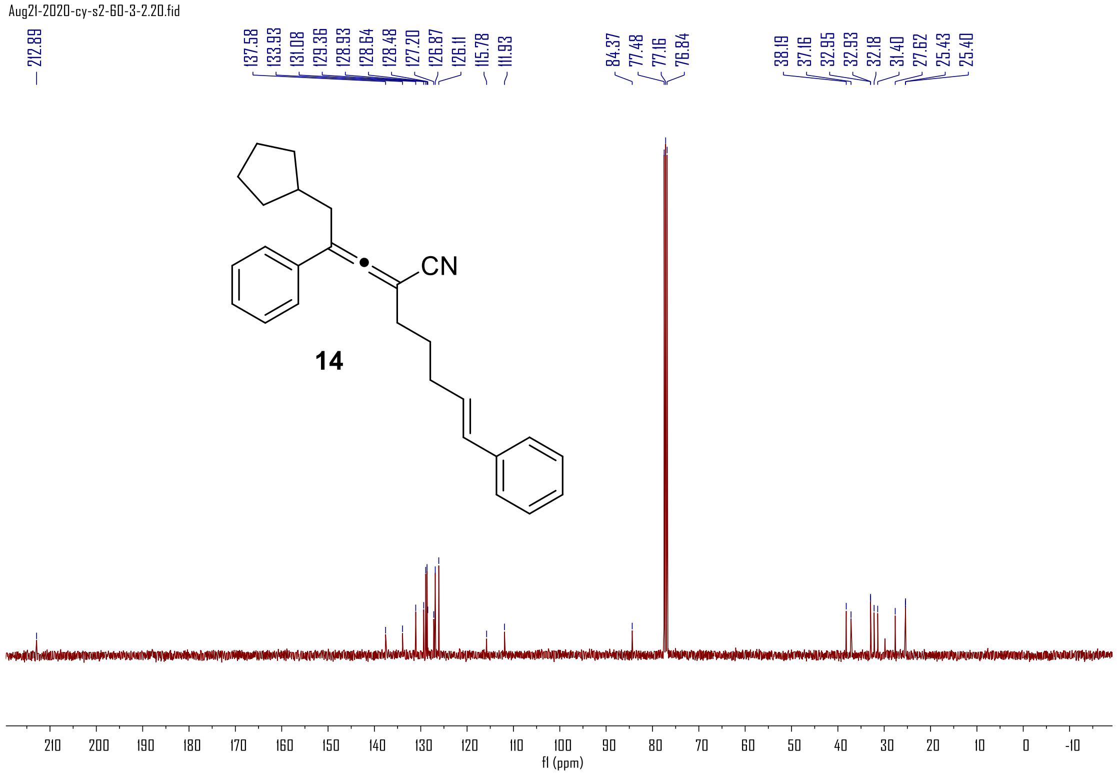
Aug21-2020-cy-s2-60-3-2.20.fid
212.89
137.58 133.93 131.08 129.36 128.93 128.64 128.48 127.20 126.87 126.11 115.78 111.93
84.37 77.48 77.16 76.84
38.19 37.16 32.95 32.93 32.18 31.40 27.62 25.43 25.40
CN
14
f1 (ppm)
